# Photoinduced cobaloxime catalysis enabled dehydrogenative C2-phosphinylation of bicyclo[1.1.0]butanes to access phosphorylated cyclobutenes†

**DOI:** 10.1039/d5ra03697f

**Published:** 2025-07-10

**Authors:** Yunong Chang, Fangyu Bian, Jiefei Guo, Miao-Miao Li, Wei Ding

**Affiliations:** a Division of Molecular Catalysis and Synthesis, Henan Institute of Advanced Technology, Zhengzhou University Zhengzhou 450001 P. R. China miaomiaoli116@zzu.edu.cn ding_w@zzu.edu.cn

## Abstract

A photoinduced ring-opening radical C2-phosphinylation of bicyclo[1.1.0]butanes with secondary phosphine oxides by cobaloxime catalysis is described. This reaction features mild conditions, wide substrate scope, and high site-selectivities, producing a diverse range of phosphorylated cyclobutenes in good yields with hydrogen evolution. The mechanism studies indicate that this reaction likely proceeds through a bicyclo[1.1.0]butane isomerization and dehydrogenative allylic phosphinylation process.

## Abstract 2

A ring-opening radical C2-phosphinylation of bicyclo[1.1.0]butanes with secondary phosphine oxides is developed by visible-light-induced cobaloxime catalysis.

## Graphical abstract

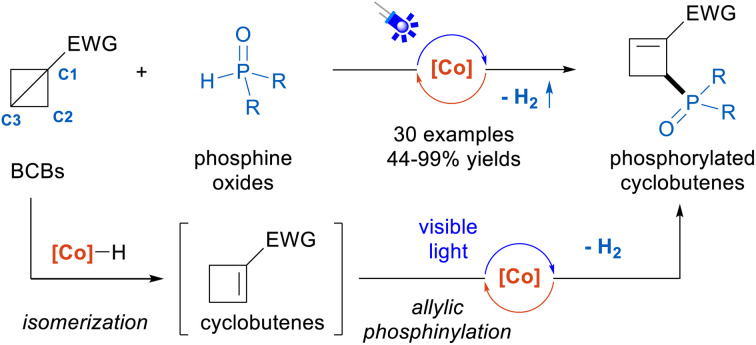

## Introduction

**Scheme 1:**
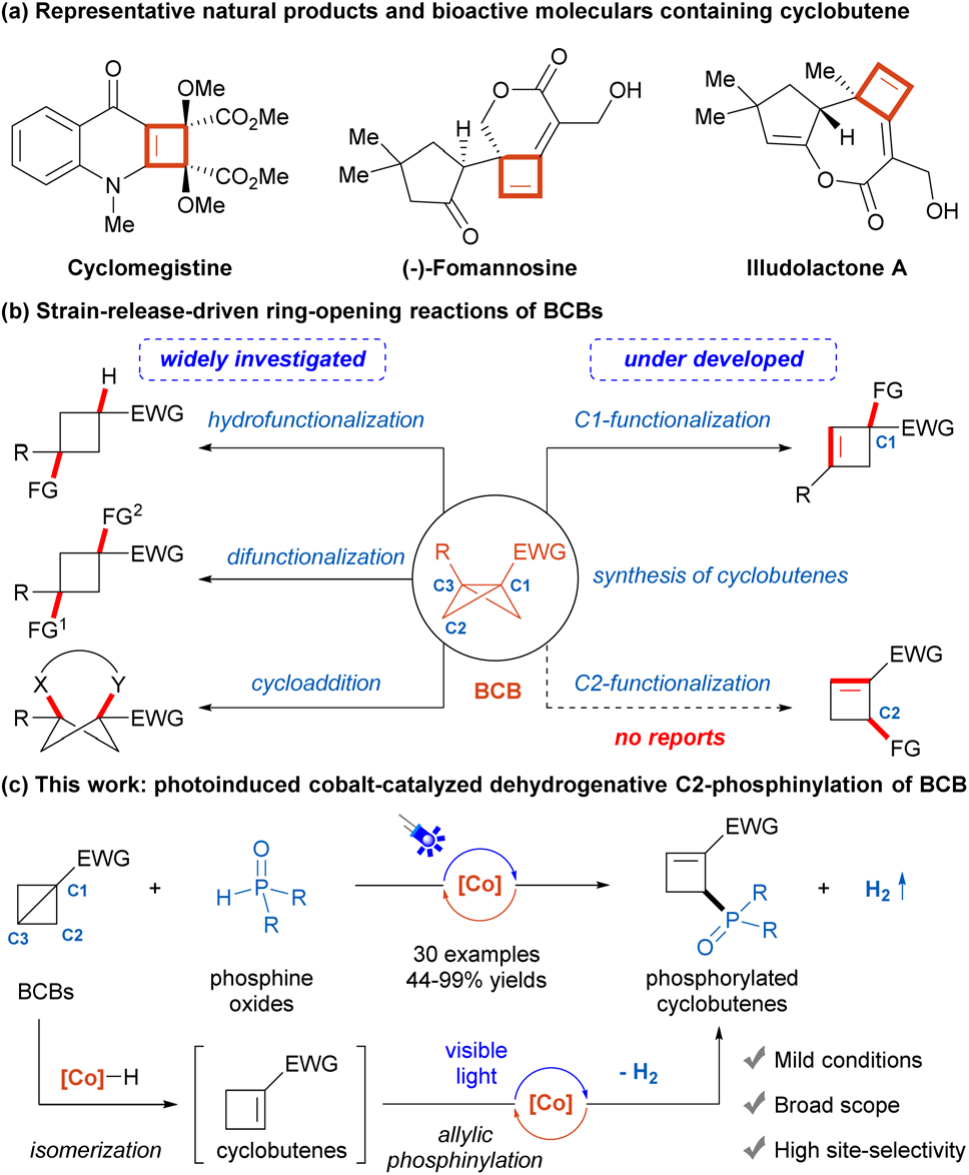
Ring-opening reactions of BCBs to synthesize cyclobutenes.

Cyclobutenes are important frameworks commonly found in natural products and pharmaceutical compounds with a wide range of biological properties (Scheme 1a),^1^ as well as versatile building blocks in organic synthesis.^2^ Consequently, the construction of these scaffolds has attracted substantial interest, and remarkable advancements have been made.^3^ However, the scope of the vast majority of known methods is limited to specialized starting materials. In recent years, bicyclo[1.1.0]butanes (BCBs), the smallest fused carbocycles, have gained increasing attention as highly reactive substrates due to their inherent significant ring strain (66 kcal mol^−1^) and high π-characters of the central C1–C3 σ bond.^4^ A series of ring-opening reactions of BCBs, including hydrofunctionalization,^5^ difunctionalization,^6^ and cycloaddition,^7^ are widely utilized for the concise and efficient synthesis of the functionalized cyclobutane derivatives and substituted aryl bioisosteres (Scheme 1b). In contrast, the selective preparations of cyclobutenes from BCBs have yet to be extensively developed and mainly afford C1-functionalized products.^8^ Particularly, to our knowledge, no reaction to synthesize C2-functionalized cyclobutenes from BCBs has been reported to date.^9^

Biomimetic excited-cobaloxime catalysis, initially developed to mimic the reaction of vitamin B_12_,^10^ has emerged as a powerful tool for organic synthesis. In 2019, Wu and co-workers originally developed a direct activation of H-phosphine oxide by photoinduced cobaloxime catalysis to afford a reactive phosphinoyl radical.^11*a*^ Since then, various dehydrogenative radical phosphorylation reactions of unsaturated compounds were realized to afford valuable alkenylphosphine oxides and phosphorylated heteroaromatics.^11^ Recently, our group has also used this strategy to achieve allylic phosphinylation of alkenes and allylamines with hydrogen evolution, in which the cobaloxime complex performed a double duty as both a photoredox catalyst and hydrogen evolution metal catalyst.^11*g*,*h*^ On the other hand, organocobalt(iii) complexs were able to catalyze the ring-opening isomerization of BCBs.^12^ Inspired by these remarkable works and our ongoing interest in photochemical synthesis,^13^ we envisaged that [Co^III^]–H species, conveniently accessible from photoinduced cobaloxime catalysis,^11^ could promote the regioselective isomerization of BCBs through a reversible addition–elimination process to access cyclobutene intermediates.^14^ The following photoinduced cobaloxime-catalyzed dehydrogenative allylic phosphinylation of cyclobutenes would afford C2-phosphorylated cyclobutenes (Scheme 1c). This reaction would feature mild reaction conditions, good site-selectivity and high atom economy with H_2_ as the byproduct. During the preparation of this manuscript, Deng and Liu reported an elegant similar transformation of 1,3-disubstituted BCBs, which gave different regioselectivities of products, compared with the present reaction.^15^

## Results and discussion

We initially chose BCB amide 1a and diphenylphosphine oxide 2a as model substrates to investigate the feasibility of our hypothesis. After extensive screening of the reaction conditions (Table 1; see Tables S1–S9 in the ESI†), we succeeded in the desired ring-opening C2-phosphinylation reaction in the presence of Co(dmgH)_2_(4-CO_2_Mepy)Cl (10 mol%) as catalyst and pyridine (1.0 equiv.) as base in DCE under irradiation of 40 W blue LEDs at room temperature, affording the phosphorylated cyclobutene product 3aa in 89% isolated yield with exclusive regioselectivity (entry 1). Moreover, the hydrogen gas was detected by GC-TCD during the reaction (see Fig. S4 in the ESI†). Control experiments indicated that cobalt catalyst and visible light were both essential for this reaction, while base significantly improved the reaction efficiency (entries 2−4). Then, a variety of cobaloxime catalysts were examined. The Co(iii) complexes were capable of catalyzing this transformation with comparable reaction efficiencies (entries 5–6, also see Table S1 in ESI†), particularly, the cobaloxime catalysts with different pyridine ligands had no dramatic effect on the product yields, which was probably caused by the ligand exchange process of cobaloxime complex with base pyridine. When using Co(dmgH)_2_(4-CO_2_Mepy)Cl as catalyst, the reaction gave a slightly increased yield. Therefore, Co(dmgH)_2_(4-CO_2_Mepy)Cl was chose as the optimized catalyst. However, the use of the Co(ii)-catalyst Co(dmgBF_2_)_2_(H_2_O)_2_ led to a significant drop in the yield of 3aa (entry 7). In addition, the use of other inorganic and organic bases instead of pyridine, such as K_2_CO_3_, Et_3_N and DBU, resulted in diminished yields (entries 8–10). Furthermore, no improvement in yield was observed upon alteration of solvents (entries 11–13).

**Table 1:** Optimization of reaction conditions^a^

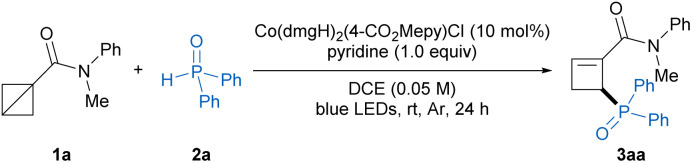		
Entry	Variation to standard conditions	Yield^b^ (%)
1	None	89
2	No Co(dmgH)_2_(4-CO_2_Mepy)Cl	0
3	No blue LEDs	0
4	No pyridine	43
5	Co(dmgH)(dmgH_2_)Cl_2_ as the catalyst	82
6	Co(dmgH)_2_pyCl as the catalyst	79
7	Co(dmgBF_2_)_2_(H_2_O)_2_ as the catalyst	15
8	K_2_CO_3_ instead of pyridine	40
9	Et_3_N instead of pyridine	23
10	DBU instead of pyridine	29
11	DCM instead of DCE	76
12	Toluene instead of DCE	54
13	MeCN instead of DCE	47

a Unless otherwise noted, the reaction conditions: 1a (0.4 mmol, 2.0 equiv), 2a (0.2 mmol), Co(dmgH)_2_(4-CO_2_Mepy)Cl (10 mol%), pyridine (1.0 equiv), DCE (4.0 mL), irradiation *via* a 40 W blue LEDs (450 nm) under Ar at room temperature for 24 h.

b Isolated yield.

Having established the optimal reaction conditions, we then explored the substrate scope of this photoinduced ring-opening phosphinylation reaction. As illustrated in Table 2, a wide variety of *N*-aryl-*N*-alkyl BCB amides with different substituents smoothly took part in the reaction to afford the desired products 3ba–3ja in 68−94% yields, which indicated that the electronic properties and steric hindrance of substrates had no much effect on the reaction efficiencies. The acyclic and cyclic *N*,*N*-dialkyl BCB amides were also amenable to this transformation to provide the corresponding products 3ka–3oa in good yields. Moreover, the relatively unstable Weinreb amide substituted BCB was well compatible with this reaction to deliver the product 3pa in 86% yield. Noteworthy, the reaction could tolerate other kinds of BCBs, including BCB esters and BCB phenyl sulfone, producing the desired products 3qa–3sa in excellent yields, whereas BCB ketones failed to participate in this protocol due to the competitive Michael addition of diphenylphosphine oxide to α,β-unsaturated ketone intermediate. Next, the scope of the secondary phosphine oxide was investigated with BCB 1a as the reaction partner. A variety of monosubstituted and disubstituted diarylphosphine oxides smoothly participated in the reaction to provide the phosphorylated cyclobutene products 3ab–3ah in 44−86% yields. The heterocycle-substituted phosphine oxides were also suitable substrates to deliver the corresponding products 3ai and 3aj in 75% and 82% yields, respectively. Furthermore, the asymmetric alkylphenylphosphine oxides could undergo this transformation smoothly to afford the desired products 3ak and 3al in good yields. Notably, the model reaction could be carried out on a 2 mmol scale with no decrease in the yield (89% yield), and the structure of product 3aa was unambiguously determined by X-ray crystallographic analysis (CCDC 2422929).

**Table 2:** Substrate scope^a^

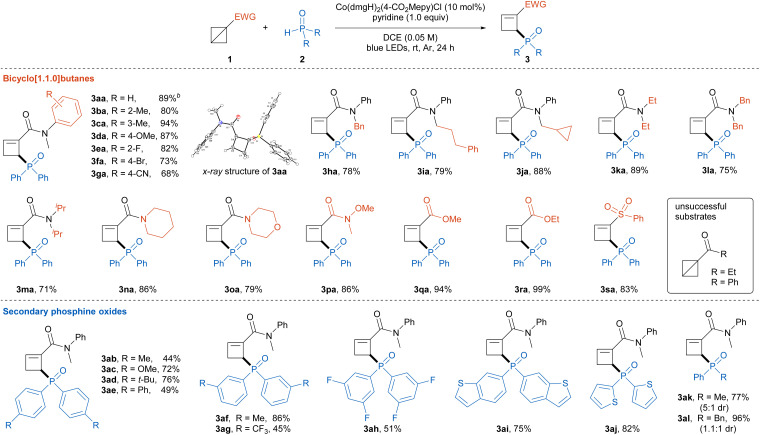

a Reaction conditions: 1 (0.4 mmol, 2.0 equiv.), 2 (0.2 mmol), Co(dmgH)_2_(4-CO_2_Mepy)Cl (10 mol%), pyridine (0.2 mmol, 1.0 equiv.), DCE (4.0 mL), irradiation *via* a 40 W blue LEDs (450 nm) under Ar at room temperature for 24 h.

b The reaction was performed on a 2.0 mmol scale for 72 h.

**Scheme 2:**
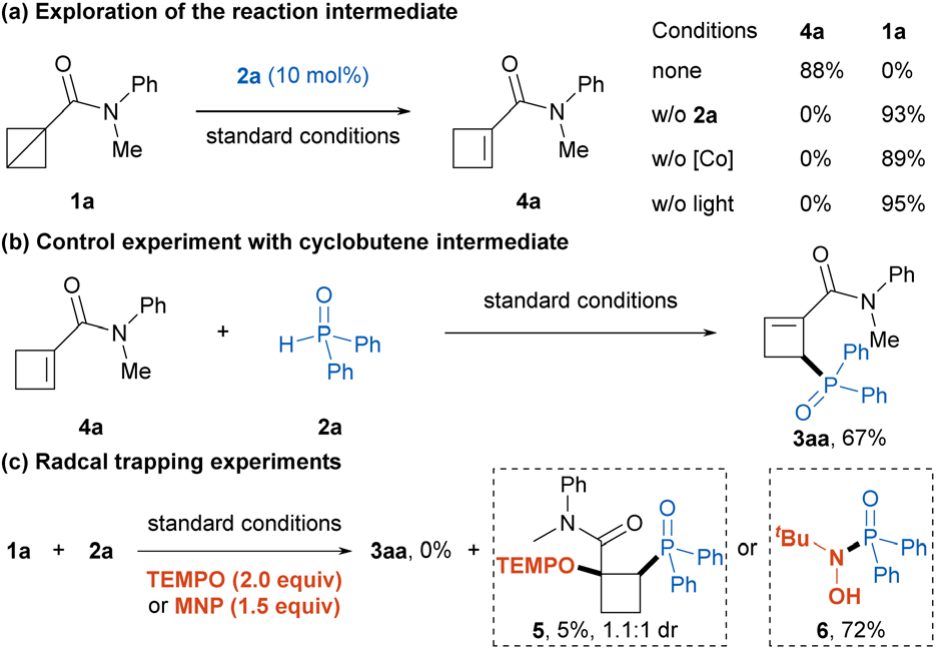
Mechanistic investigations. (a and b) Exploration of cyclobutene intermediate, (c) radical trapping experiments.

To gain some insights into the reaction mechanism, several mechanistic experiments were conducted (Scheme 2). First, we probed the reaction intermediates with 10 mol% of diphenylphosphine oxide 2a, in which the ring-opening isomerization product 4a of BCB 1a was isolated in 88% yield with exclusive site-selectivity (Scheme 2a). Control experiments showed that 2a, cobaloxime and visible light were indispensable in the conversion of BCB 1a to intermediate 4a. Meanwhile, the reaction of cyclobutene 4a and 2a under standard conditions afforded product 3aa in 67% yield, which suggested the cyclobutene 4a was probably the key intermediate of this reaction (Scheme 2b). In addition, the radical trapping experiments with 2,2,6,6-tetramethylpiperidin-1-oxyl (TEMPO) and 2-methyl-2-nitrosopropane (MNP) gave a complete suppression of the ring-opening phosphinylation reaction, while the radical adducts 5 and 6 were obtained in 5% and 72% yields, respectively, which indicated that the reaction might proceed through a phosphorus radical pathway (Scheme 2c).

**Scheme 3:**
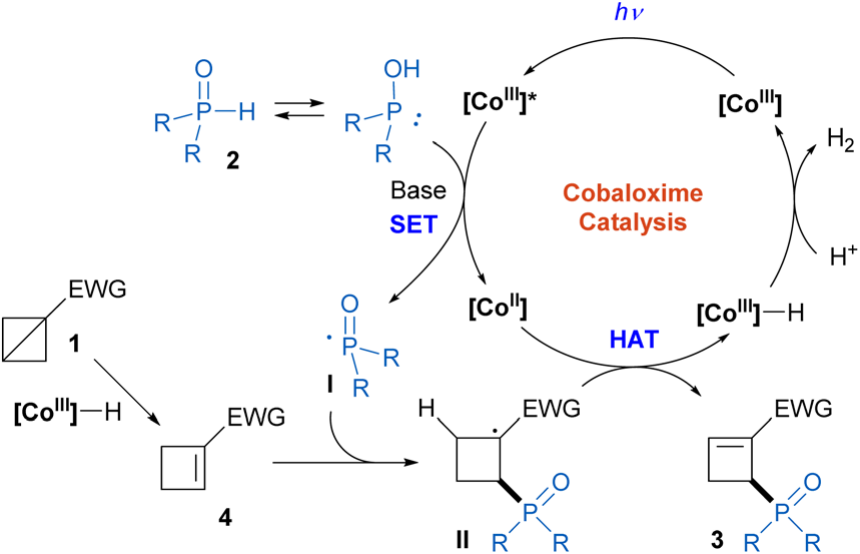
Proposed reaction mechanism.

Based on the above results and related literatures,^11–15^ we proposed a plausible mechanism for this transformation (Scheme 3). First, the [Co^III^]–H complex, which was conveniently generated in the initial photoinduced cobaloxime-catalyzed radical phosphinylation process (Scheme 2a),^13*d*^ could promote BCB isomerization to produce the cyclobutene intermediate 4. The regioselectivity of this step is distinct from Deng and Liu's work,^15^ which may be attributed to the different BCB substrates. Meanwhile, the photoexcited [Co^III^] complex (*E*(Co^III^*/Co^II^) = +2.2 V *vs.* SCE)^11*a*,*c*^ oxidized H-phosphine oxide 2 (*E*^ox^ = +1.22 V *vs.* SCE for 2a)^11*c*^ with the facilitation of a base to form the phosphinoyl radical I and [Co^II^] species. Then, the regioselective addition of radical I to cyclobutene 4 afforded carbon radical intermediate II. The subsequent hydrogen atom abstraction by the [Co^II^] species generated the phosphinylation product 3 and [Co^III^]–H complex. Finally, the [Co^III^]–H complex reacted with a proton to release H_2_ with the regeneration of the [Co^III^] catalyst.

**Scheme 4:**
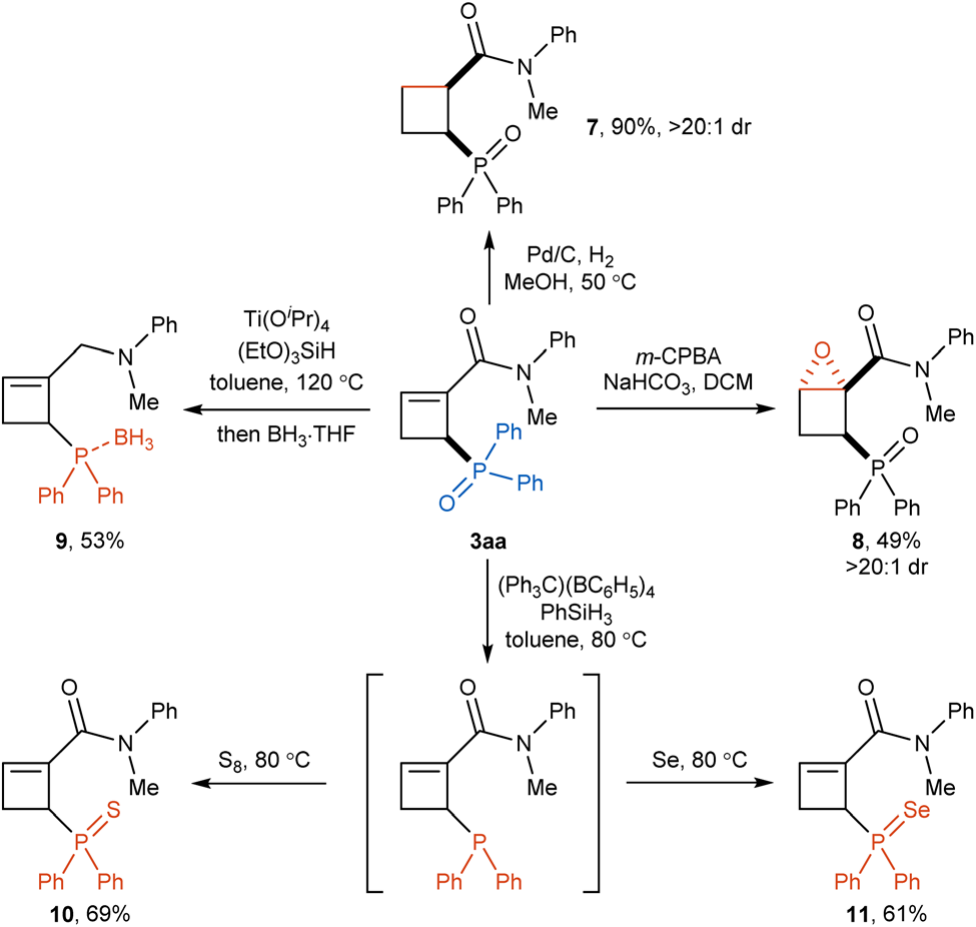
Synthetic applications.

The phosphorylated cyclobutene products obtained by the present reaction could be used as versatile building blocks for diverse further transformations (Scheme 4). The reduction of the C–C double bond of cyclobutene product 3aa with a catalytic amount of Pd/C under a hydrogen gas atmosphere afforded cyclobutane 7 in good yield with excellent diastereoselectivity. In addition, epoxidation of the olefin moiety in 3aa with *m*CPBA could be occurred smoothly to provide fused-cyclic compound 8 in 49% yield with exclusive diastereoselectivity. To our delight, simultaneous reduction of phosphoryl and amide groups in 3aa was achieved using Ti(O*i*-Pr)_4_ and (EtO)_3_SiH to afford phosphine compound 9 in 53% yield. Furthermore, the phosphine oxide group could be selectively reduced by PhSiH_3_ to give trivalent phosphine intermediate, following by the oxidation with elemental sulfur and selenium to obtain phosphine sulfide 10 and phosphine selenide 11 in 69% and 61% yields, respectively.

## Conclusions

In summary, we developed a ring-opening C2-phosphinylation of BCBs with secondary phosphine oxides by visible-light-induced cobaloxime catalysis. This reaction features a wide substrate scope to produce a diverse range of phosphorylated cyclobutenes in good yields with H_2_ as the byproduct. The preliminary mechanism studies revealed a BCB isomerization and dehydrogenative allylic phosphinylation process in this approach.

## Author contributions

W. Ding and M.-M. Li conceived the idea and supervised the project. Y. Chang, F. Bian and J. Guo designed and conducted all experiments and analysed the data. W. Ding and Y. Chang wrote the manuscript with the input from all authors.

## Conflicts of interest

There are no conflicts to declare.

## Supplementary Material

RA-015-D5RA03697F-s001

RA-015-D5RA03697F-s002

## Data Availability

The data supporting this article have been included as part of the ESI.†
