# Supplementary material for: Photoinduced cobaloxime catalysis enabled dehydrogenative C2-phosphinylation of bicyclo[1.1.0]butanes to access phosphorylated cyclobutenes
Source: RSC Adv. 2025 Jul 10;15(29):24053–7. doi: 10.1039/d5ra03697f (PMC12243002; doi:10.1039/d5ra03697f)
Supplement: RA-015-D5RA03697F-s001 [file RA-015-D5RA03697F-s001.pdf]

Supplementary Information (SI) for RSC Advances.
This journal is © The Royal Society of Chemistry 2025

Supporting Information

Photoinduced Cobaloxime Catalysis Enabled Dehydrogenative C2-Phosphinyayion of Bicyclo[1.1.0]butanes to Access Phosphorylated Cyclobutenes

Yunong Chang, Fangyu Bian, Jiefei Guo, Miao-Miao Li* and Wei Ding*

Division of Molecular Catalysis and Synthesis, Henan Institute of Advanced Technology, Zhengzhou University, Zhengzhou 450001, P. R. China

Email: miaomiaoli116@zzu.edu.cn; ding_w@zzu.edu.cn

Contents

1. General Information S2
2. Preparation of Starting Materials S4
3. Optimization of Reaction Conditions S5
4. Experimental Procedures and Product Characterization S11
5. Mechanistic Studies S29
6. Product Transformations S35
7. X-Ray Diffraction Analysis S40
8. References S42
9. NMR Spectra S43

1. General Information

General. All reactions dealing with air- or moisture-sensitive compound were performed by standard Schlenk techniques in oven-dried reaction vessels under argon atmosphere or in an argon-filled glove box. Analytical thin-layer chromatography (TLC) was performed on Merck 60 F254 silica gel plates. Flash chromatography was performed as described by Still et al., using 200-300 mesh silica gel. ^{1}H, ^{13}C, ^{19}F and ^{31}P nuclear magnetic resonance (NMR) spectra were recorded on Bruker AVANCE NEO 400 M NMR spectrometers in Zhengzhou University (North Campus). ^{1}H and ^{13}C NMR spectra are reported in parts per million (ppm) downfield from an internal standard, tetramethylsilane (0 ppm), $CDCl_3$ (77.0 ppm), and DMSO-d_6 (39.5 ppm), respectively. High-resolution mass spectra (HRMS) were obtained with an Agilent 6210 ESI/TOF mass spectrometer. Melting points were determined using a capillary melting point apparatus.

Materials. Unless otherwise noted, commercial reagents were purchased from Energy Chemical, Bidepharm, *J*&K Scientific or other commercial suppliers and were used as received. DCE, DCM and MeCN were distilled over CaH_2 and stored under Ar. THF and toluene were distilled over Na/benzophenone, and stored under Ar. Anhydrous DMF and DMSO were purchased from *J*&K Scientific. Cobaloxime complexs were prepared according to the literature procedures[1], and purified by recrystallization.

Photoreactor. The photoreactors used in this research were bought from GeAo Chem (Figure S1: blue LEDs). Two parallel LED lamps (total 40 W, λ_{max} = 450 nm) are placed perpendicular to the sidewall of the reaction vessels, so that the reaction vessels can be equally exposed to LEDs (about 5 W was distributed to each hole). 10 mL Schlenk tube bought from SYNTHWARE GLASS, was used as photoreaction vessel, which was positioned 2-3 cm from the blue LED lamp. During the reaction, a pinch fan at one end of the equipment keeps working, counteracting the heat generated by the LED lamp and stabilizing the reaction temperature.

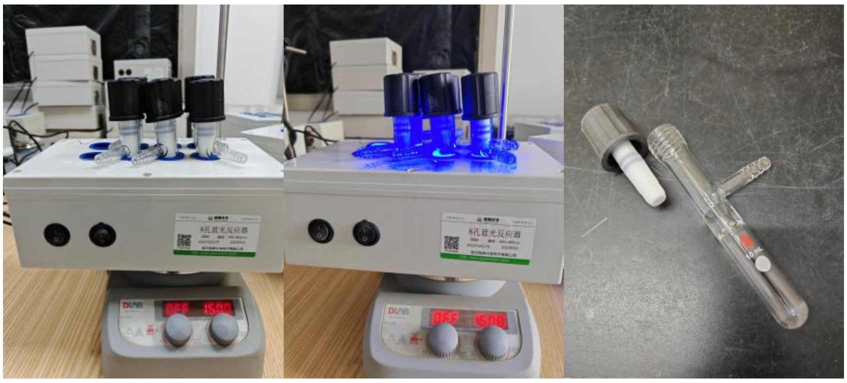

Figure S1. Photoreaction set-up and reaction vessel.

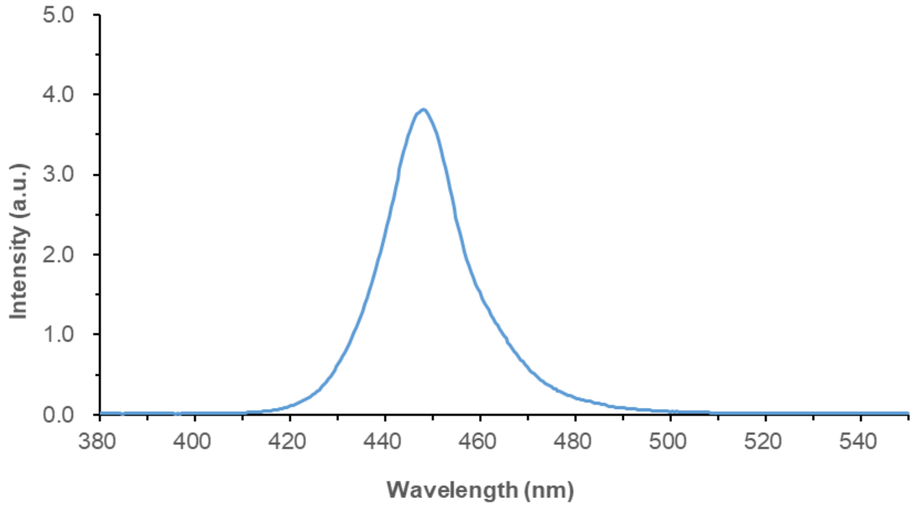

Figure S2. Light spectrum of the photon source: 40 W blue LEDs (λ_{max} = 450 nm).

2. Preparation of Starting Materials

All bicyclo[1.1.0]butanes were synthesized according to the literature procedures,[2-7] and purified by flash chromatography. Spectral data of the bicyclo[1.1.0]butanes **1a-1r**,[2] **1s**,[7] showed good agreement with the literature data. Except for some commercially available compounds, phosphine oxides were synthesized according to the literature procedures,[8-10] and purified by flash chromatography. Spectral data of the phosphine oxides **2d**,[8] **2e**,[9] **2i-2j**,[10] showed good agreement with the literature data.

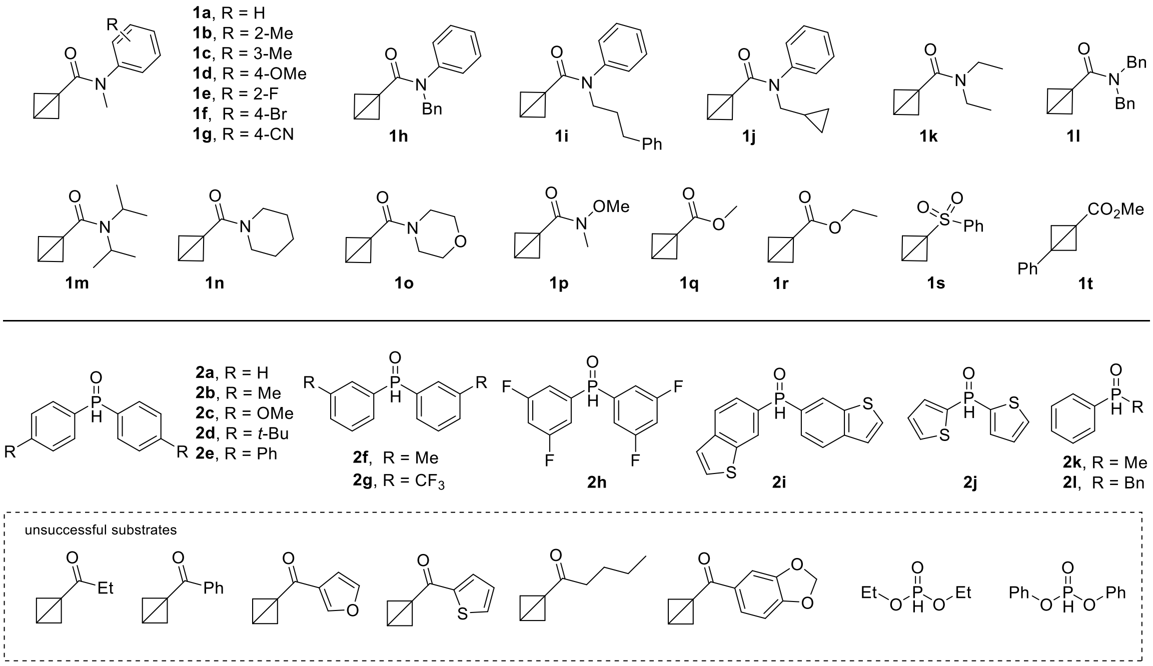

Figure S3. Bicyclo[1.1.0]butanes and secondary phosphine oxides in this study.

3. Optimization of Reaction Conditions

Table S1. Cobaloxime catalyst effect

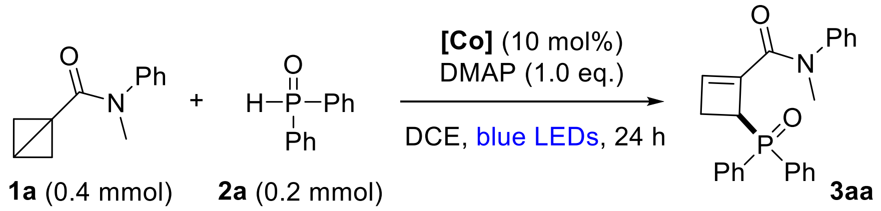

Entry	[Co]	Yield
1	$[Co(dmgH)(dmgH_2)Cl_2]$	82%
2	$[Co(dmgH)_2(4\text{-}CO_2Mepy)Cl]$	83%
3	$[Co(dmgH)_2(4\text{-}CNpy)Cl]$	83%
4	$[Co(dmgH)_2pyCl]$	79%
5	$[Co(dmgH)_2py_2]PF_6$	75%
6	$[Co(dmgH)_2(4\text{-}(DMAPpy)Cl]$	67%
7	$[Co(chgH)(chgH)Cl_2]$	56%
8	$[Co(chgH)_2(4\text{-}CO_2MePy)Cl]$	57%
9	$[Co(chgH)_2PyCl]$	63%
10	$Co(dmgBF_2)_2(H_2O)_2$	25%

Reaction condition: **1a** (0.4 mmol), **2a** (0.2 mmol), [Co] 10 mol% and DMAP (0.2 mmol) in DCE (4 mL) was irradiated with 40 W blue LEDs for 24 h under argon atmosphere at rt. Isolated yield.

Table S2. Base effect

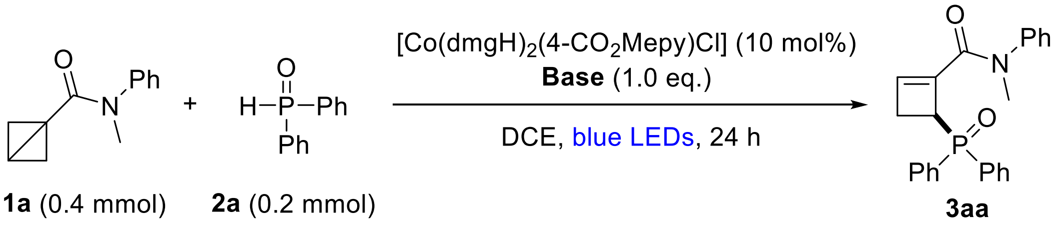

Entry	Base	Yield
1	DMAP[a]	77%
2	LiOH	5%
3	K_2CO_3	40%
4	$NaHCO_3$	64%
5	Cs_2CO_3	18%
6	DABCO	43%
7	K_2HPO_4	27%
8	pyridine	89%
9	TMEDA	35%
10	DIPEA	49%
11	CsF	trace
12	KO^{t}Bu	30%
13	Et_3N	23%
14	(^{i}Pr)$_2$NH	trace
15	DBU	29%
16	2,6-Lutidine	25%
17	2,6-di-tert-butylpyridine	67%
18	4-Cyanopyridine	trace
19	4-Aminopyridine	50%
20	2-Chloro-6-(trifluoromethyl)pyridine	48%

Reaction condition: **1a** (0.4 mmol), **2a** (0.2 mmol), [Co(dmgH)$_2$(4-CO$_2$Mepy)Cl] (10 mol%) and base (0.2 mmol) in DCE (4 mL) was irradiated with 40 W blue LEDs for 24 h under argon atmosphere at rt. Isolated yield. [a] DMAP (0.1 mmol) was used.

Table S3. Solvent effect

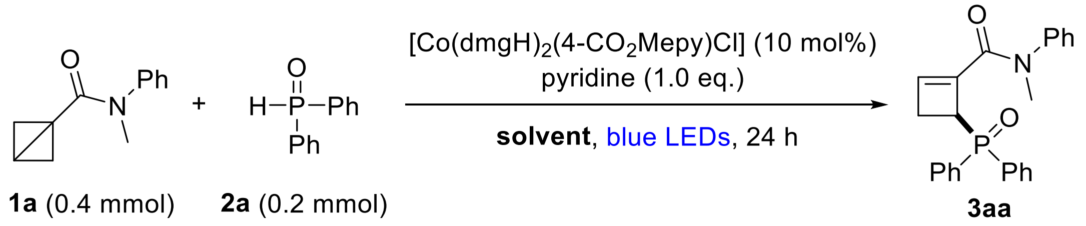

Entry	Solvent	Yield
1	DCE	89%
2	DCM	76%
3	PhCl	66%
4	1,2-Dichlorobenzene	57%
5	$CHCl_3$	58%
6	EtOAc	18%
7	THF	12%
8	EtOH	13%
9	Et_2O	14%
10	DMF	trace
11	DMSO	10%
12	MeCN	47%
13	Toluene	54%

Reaction condition: **1a** (0.4 mmol), **2a** (0.2 mmol), Co(dmgH)$_2$(4-CO$_2$Me)Cl (10 mol%) and pyridine (0.2 mmol) in solvent (4 mL) was irradiated with 40 W blue LEDs for 24 h under argon atmosphere at rt. Isolated yield.

Table S4. The effect of amount of cobaloxime

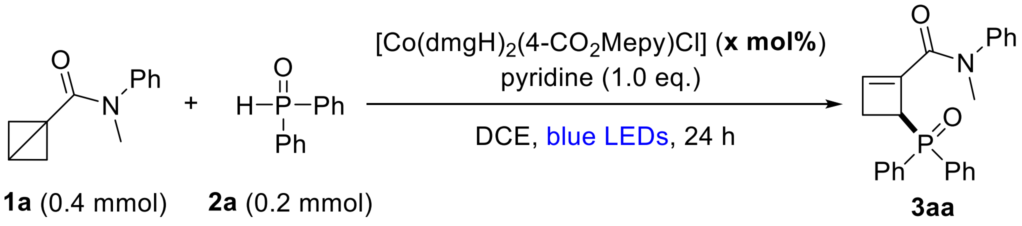

Entry	x mol%	Yield
1	2 mol%	24%
2	4 mol%	50%
3	6 mol%	55%
4	8 mol%	64%
5	10 mol%	89%
6	12 mol%	83%
7	15 mol%	80%

Reaction condition: **1a** (0.4 mmol), **2a** (0.2 mmol), Co(dmgH)$_2$(4-CO$_2$Me)pyCl (x mol%) and pyridine (0.2 mmol) in DCE (4 mL) was irradiated with 40 W blue LEDs for 24 h under argon atmosphere at rt. Isolated yield.

Table S5. The effect of amount of base

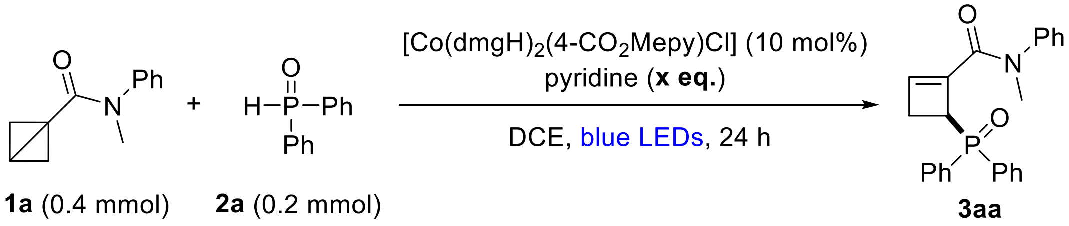

Entry	x eq.	Yield
1	0.25 eq.	74%
2	0.5 eq.	76%
3	0.75 eq.	78%
4	1.0 eq.	89%

Reaction condition: **1a** (0.4 mmol), **2a** (0.2 mmol), Co(dmgH)$_2$(4-CO$_2$Mepy)Cl (10 mol%) and pyridine (x mmol) in DCE (4 mL) was irradiated with 40 W blue LEDs for 24 h under argon atmosphere at rt. Isolated yield.

Table S6. The effect of molar ratio of **1a** and **2a**

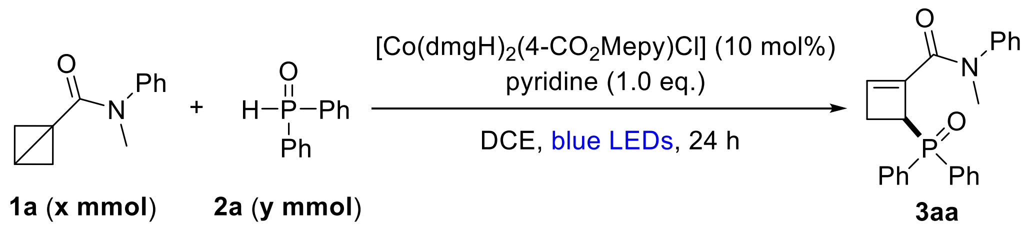

Entry	1a : 2a	Yield
1	0.20 mmol : 0.20 mmol	70%
2	0.26 mmol : 0.20 mmol	76%
3	0.30 mmol : 0.20 mmol	76%
4	0.34 mmol : 0.20 mmol	77%
5	0.38 mmol : 0.20 mmol	83%

Reaction condition: **1a** (x mmol), **2a** (y mmol), $Co(dmgH)_2(4\text{-}CO_2Me)pyCl$ (10 mol%) and pyridine (0.2 mmol) in DCE (4 mL) was irradiated with 40 W blue LEDs for 24 h under argon atmosphere at rt. Isolated yield.

Table S7. The effect of reaction concentration

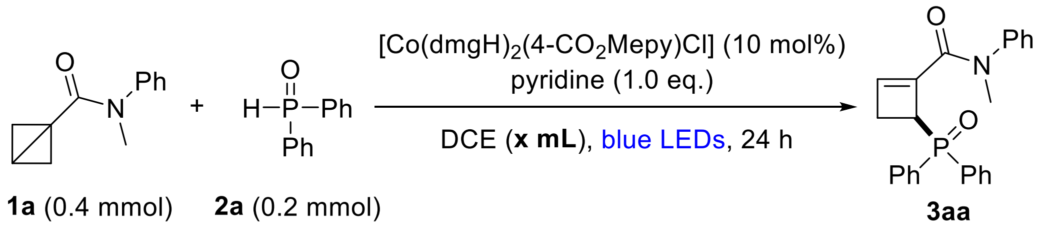

Entry	x mL	Yield
1	1.0	74%
2	2.0	86%
3	3.0	86%
4	4.0	89%

Reaction condition: **1a** (0.4 mmol), **2a** (0.2 mmol), $Co(dmgH)_2(4\text{-}CO_2Me)pyCl$ (10 mol%) and pyridine (0.2 mmol) in DCE (x mL) was irradiated with 40 W blue LEDs for 24 h under argon atmosphere at rt. Isolated yield.

Table S8. The effect of reaction time

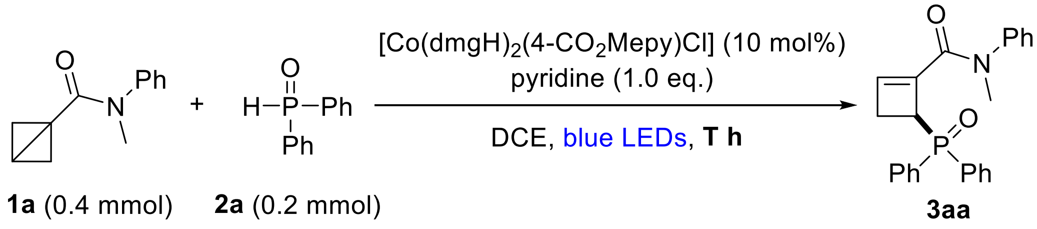

Entry	T h	Yield
1	6.0	50%
2	12.0	65%
3	24.0	89%

Reaction condition: **1a** (0.4 mmol), **2a** (0.2 mmol), Co(dmgH)$_2$(4-CO$_2$Me)pyCl (10 mol%) and pyridine (0.2 mmol) in DCE (4 mL) was irradiated with 40 W blue LEDs for T h under argon atmosphere at rt. Isolated yield.

Table S9. Control experiments

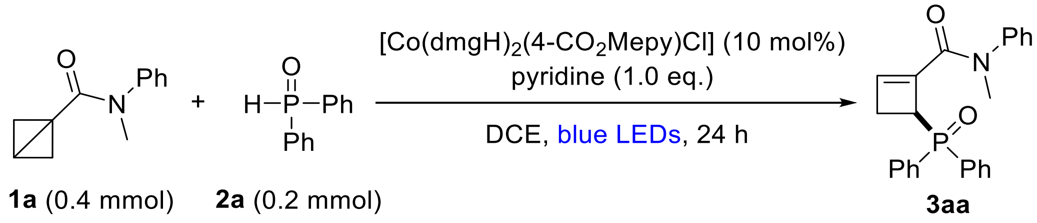

Entry	Variation to Standard Conditions	Yield
1	no visible light	0%
2	no pyridine	43%
3	no Co(dmgH)$_2$(4-CO$_2$Mepy)Cl	0%
4	none	89%

Reaction condition: **1a** (0.4 mmol), **2a** (0.2 mmol), Co(dmgH)$_2$(4-CO$_2$Me)pyCl (10 mol%) and pyridine (0.2 mmol) in DCE (4 mL) was irradiated with 40 W blue LEDs for 24 h under argon atmosphere at rt. Isolated yield.

4. Experimental Procedures and Product Characterization

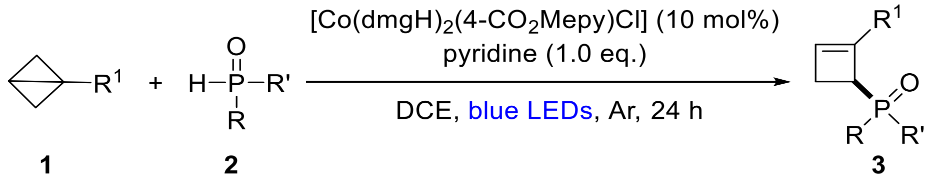

General procedure: Under an argon atmosphere, an oven-dried 10 mL Schlenk tube equipped with a magnetic stir bar was charged with Co(dmgH)$_2$(4-CO$_2$Mepy)Cl (9.2 mg, 0.02 mmol, 10 mol%) and bicyclo[1.1.0]butane **1** (0.40 mmol, 2.0 eq.). Then, the Schlenk tube was introduced in a glovebox, where it was charged with phosphine oxide compound **2** (0.20 mmol, 1.0 eq.). The tube was taken out of the glovebox and connected to a vacuum line through evacuating and back-filling with Ar for 3 times. After DCE (4 mL) and pyridine (0.20 mmol, 1.0 eq.) were added under Ar flow, the resulting mixture was degassed via 'freeze-pump-thaw' procedure (3 times) under Ar atmosphere and was stirred under blue LED (40 W) at room temperature for 24 h (monitored by TLC analysis). The reaction mixture was concentrated under reduced pressure and the crude residue was purified by column chromatography on silica gel (eluent: petroleum ether: ethyl acetate: ethanol= 30:1:1-10:1:1) to give the desired products **3**.

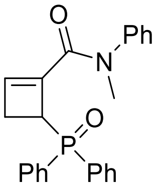

4-(Diphenylphosphoryl)-*N*-methyl-*N*-phenylcyclobut-1-ene-1-carboxamid (3aa): Prepared according to the general procedure from **1a** (0.40 mmol) and **2a** (0.20 mmol), and purified by column chromatography on silica gel with petroleum ether/ethyl acetate/ethanol (10/1/1) to provide the title compound **3aa** (69.3 mg, 89% yield) as a white solid; R_f 0.2 (petroleum ether/ethyl acetate/ethanol = 10/2/1); m.p. 170.2–172.4 °C; ^{1}H NMR (400 MHz, CDCl$_3$) δ 7.91–7.79 (m, 4H), 7.57–7.44 (m, 6H), 7.34–7.28 (m, 3H), 7.01–6.90 (m, 2H), 5.21 (s, 1H), 3.82 (s, 1H), 3.16 (s, 3H), 2.56–2.36 (m, 2H); ^{13}C NMR (100 MHz, CDCl$_3$) δ 161.9, 143.1, 142.8 (d, J_{C-P} = 12.5 Hz), 138.7 (d, J_{C-P} = 8.2 Hz), 133.1 (d, J_{C-P} = 97.5 Hz), 132.0 (d, J_{C-P} = 98.9 Hz), 131.7 (d, J_{C-P} = 3.2 Hz), 131.6 (d, J_{C-P} = 3.2 Hz), 131.5 (d, J_{C-P} = 9.4 Hz), 130.9 (d, J_{C-P} = 9.3 Hz), 129.3, 128.4 (d, J_{C-P} = 11.5 Hz), 128.1 (d, J_{C-P} = 11.7 Hz), 127.7, 127.4, 41.9 (d, J_{C-P} = 72.6 Hz), 37.5, 28.1 (d, J_{C-P} = 5.5 Hz); ^{31}P NMR (162 MHz, CDCl$_3$) δ 29.6; HRMS (ESI) Calcd for $C_{24}H_{22}NO_2PNa$ $[M + Na]^+$ 410.1280, found 410.1288.

Procedure for 2 mmol-scale reaction: Under an argon atmosphere, an oven-dried 100 mL Schlenk tube equipped with a magnetic stir bar was charged with Co(dmgH)$_2$(4-CO$_2$Mepy)Cl

(92.0 mg, 0.2 mmol, 10 mol%) and bicyclo[1.1.0]butane **1a** (748.0 mg, 4.0 mmol, 2.0 eq.). Then, the Schlenk tube was introduced in a glovebox, where it was charged with diphenylphosphine oxide **2a** (404.0 mg, 2.0 mmol, 1.0 eq.). The tube was taken out of the glovebox and connected to a vacuum line through evacuating and back-filled with Ar for 3 times. After dry DCE (40 mL) and pyridine (162.0 μL, 2.0 mmol, 1.0 eq.) were added under Ar flow, the resulting mixture was degassed via 'freeze-pump-thaw' procedure (3 times) under Ar atmosphere and was stirred under blue LED (40 W) at rt for 72 h (monitored by TLC analysis). The mixture was concentrated under reduced pressure and purified by flash column chromatography to give the product **3aa** (690.4 mg, 89% yield).

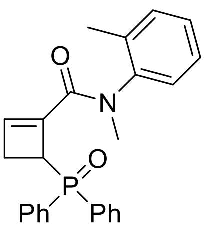

4-(Diphenylphosphoryl)-*N*-methyl-*N*-(*o*-tolyl)cyclobut-1-ene-1-carboxamide (3ba): Prepared according to the general procedure from **1b** (0.40 mmol) and **2a** (0.20 mmol), and purified by column chromatography on silica gel with petroleum ether/ethyl acetate/ethanol (10/1/1) to provide the title compound **3ba** (63.8 mg, 80% yield) as a white solid; R_f 0.3 (petroleum ether/ethyl acetate/ethanol = 10/2/1); m.p. 141.1–143.0 °C; please note that the *o*-methyl phenyl leads to the existence of atropisomers of the amide bond in **3ba**, so the HNMR data is the result of diastereoisomers mixture (isomer ratio = 1.2:1), ^{1}H NMR (400 MHz, $CDCl_3$) δ 7.94–7.78 (m, 8H), 7.54–7.41 (m, 12H), 7.26–7.12 (m, 6H), 6.99 (d, *J* = 7.6 Hz, 1H), 6.81 (d, *J* = 7.7 Hz, 1H), 4.82 (s, 1H), 4.66 (s, 1H), 4.11 (s, 1H), 3.98 (s, 1H), 3.10 (s, 3H), 3.08 (s, 3H), 2.58–2.42 (m, 2H), 2.41–2.35 (m, 2H), 2.18 (s, 3H), 2.15 (s, 3H); ^{13}C NMR (100 MHz, $CDCl_3$) δ 161.22, 161.2, 142.8 (d, $J_{C\text{-}P}$ = 12.7 Hz), 142.6 (d, $J_{C\text{-}P}$ = 12.7 Hz), 141.82, 141.80, 138.7 (d, $J_{C\text{-}P}$ = 8.4 Hz), 138.62 (d, $J_{C\text{-}P}$ = 8.7 Hz), 136.6, 135.5, 133.7 (d, $J_{C\text{-}P}$ = 97.4 Hz), 133.5 (d, $J_{C\text{-}P}$ = 98.0 Hz), 132.4 (d, $J_{C\text{-}P}$ = 99.1 Hz), 132.2 (d, $J_{C\text{-}P}$ = 99.3 Hz), 131.6 (d, $J_{C\text{-}P}$ = 5.8 Hz), 131.55 (d, $J_{C\text{-}P}$ = 9.3 Hz), 131.50 (d, $J_{C\text{-}P}$ = 7.3 Hz), 131.49 (d, $J_{C\text{-}P}$ = 9.7 Hz), 131.40 (d, $J_{C\text{-}P}$ = 5.4 Hz), 131.39 (d, $J_{C\text{-}P}$ = 5.1 Hz), 130.91 (d, $J_{C\text{-}P}$ = 9.0 Hz), 130.90, 130.7 (d, $J_{C\text{-}P}$ = 9.0 Hz), 128.8, 128.51 (d, $J_{C\text{-}P}$ = 2.2 Hz), 128.50 (d, $J_{C\text{-}P}$ = 13.5 Hz), 128.46 (d, $J_{C\text{-}P}$ = 12.3 Hz), 128.40 (d, $J_{C\text{-}P}$ = 1.7 Hz), 128.34 (d, $J_{C\text{-}P}$ = 11.5 Hz), 128.33 (d, $J_{C\text{-}P}$ = 10.8 Hz), 128.3 (d, $J_{C\text{-}P}$ = 2.1 Hz), 128.0 (d, $J_{C\text{-}P}$ = 1.8 Hz), 127.9, 127.4, 41.5 (d, $J_{C\text{-}P}$ = 72.9 Hz), 41.3 (d, $J_{C\text{-}P}$ = 72.8 Hz), 36.44, 36.40, 27.8 (d, $J_{C\text{-}P}$ = 5.4 Hz), 27.6 (d, $J_{C\text{-}P}$ = 5.6 Hz), 17.3, 17.2; ^{31}P NMR (162 MHz, $CDCl_3$) δ 30.0, 28.9; HRMS (ESI) Calcd for $C_{25}H_{24}NO_2PNa$ $[M + Na]^+$ 424.1437, found 424.1447.

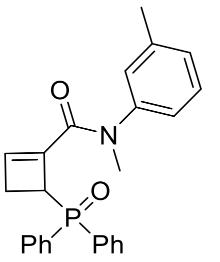

4-(Diphenylphosphoryl)-*N*-methyl-*N*-(*m*-tolyl)cyclobut-1-ene-1-carboxamide (3ca): Prepared according to the general procedure from **1c** (0.40 mmol) and **2a** (0.20 mmol), and purified by column chromatography on silica gel with petroleum ether/ethyl acetate/ethanol (10/1/1) to provide the title compound **3ca** (75.5 mg, 94% yield) as a white solid; R*f* 0.2 (petroleum ether/ethyl acetate/ethanol = 10/2/1); m.p. 127.3–129.3 °C; ^{1}H NMR (400 MHz, $CDCl_3$) δ 7.86 (q, *J* = 10.4, 5.3 Hz, 4H), 7.62–7.43 (m, 6H), 7.17 (dd, *J* = 7.7, 3.6 Hz, 1H), 7.08 (t, *J* = 5.5 Hz, 1H), 6.74 (d, *J* = 7.6 Hz, 1H), 6.65 (s, 1H), 5.24 (s, 1H), 3.76 (s, 1H), 3.12 (s, 3H), 2.58–2.38 (m, 2H), 2.30 (s, 3H); ^{13}C NMR (100 MHz, $CDCl_3$) δ 161.9, 142.9, 142.8 (d, $J_{C\text{-}P}$ = 12.4 Hz), 139.3, 138.9 (d, $J_{C\text{-}P}$ = 8.3 Hz), 133.2 (d, $J_{C\text{-}P}$ = 97.8 Hz), 131.7 (d, $J_{C\text{-}P}$ = 98.2 Hz), 131.6 (d, $J_{C\text{-}P}$ = 3.2 Hz), 131.56 (d, $J_{C\text{-}P}$ = 9.2 Hz), 131.55 (d, $J_{C\text{-}P}$ = 3.2 Hz), 130.9 (d, $J_{C\text{-}P}$ = 9.3 Hz), 129.0, 128.5, 128.4 (d, $J_{C\text{-}P}$ = 11.7 Hz), 128.1 (d, $J_{C\text{-}P}$ = 11.8 Hz), 127.7, 124.4, 42.1 (d, $J_{C\text{-}P}$ = 72.7 Hz), 37.4, 28.1 (d, $J_{C\text{-}P}$ = 5.4 Hz), 21.2; ^{31}P NMR (162 MHz, $CDCl_3$) δ 29.4; HRMS (ESI) Calcd for $C_{25}H_{25}NO_2P$ $[M + H]^+$ 402.1617, found 402.1628.

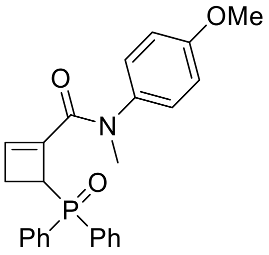

4-(Diphenylphosphoryl)-*N*-(4-methoxyphenyl)-*N*-methylcyclobut-1-ene-1-carboxamide (3da): Prepared according to the general procedure from **1d** (0.40 mmol) and **2a** (0.20 mmol), and purified by column chromatography on silica gel with petroleum ether/ethyl acetate/ethanol (10/1/1) to provide the title compound **3da** (72.3 mg, 87% yield) as a white solid; R*f* 0.2 (petroleum ether/ethyl acetate/ethanol = 10/2/1); m.p. 171.0–172.1 °C; ^{1}H NMR (400 MHz, $CDCl_3$) δ 7.86 (q, *J* = 9.7 Hz, 4H), 7.62–7.38 (m, 6H), 6.96–6.73 (m, 4H), 5.19 (s, 1H), 3.85 (s, 1H), 3.79 (s, 3H), 3.11 (s, 3H), 2.61–2.35 (m, 2H); ^{13}C NMR (100 MHz, $CDCl_3$) δ 162.0, 158.9, 142.6 (d, $J_{C\text{-}P}$ = 12.6 Hz), 138.9 (d, $J_{C\text{-}P}$ = 8.0 Hz), 135.9, 133.2 (d, $J_{C\text{-}P}$ = 97.4 Hz), 131.9 (d, $J_{C\text{-}P}$ = 99.3 Hz), 131.53 (d, $J_{C\text{-}P}$ = 2.2 Hz), 131.50 (d, $J_{C\text{-}P}$ = 2.5 Hz), 131.4 (d, $J_{C\text{-}P}$ = 9.4 Hz), 130.9 (d, $J_{C\text{-}P}$ = 9.2 Hz), 128.5, 128.4 (d, $J_{C\text{-}P}$ = 11.6 Hz), 128.1 (d, $J_{C\text{-}P}$ = 11.9 Hz), 114.4, 55.4, 41.9 (d, $J_{C\text{-}P}$ = 72.9 Hz), 37.6, 28.1 (d, $J_{C\text{-}P}$ = 5.5 Hz); ^{31}P NMR (162 MHz, $CDCl_3$) δ 29.3;

HRMS (ESI) Calcd for $C_{25}H_{24}NO_3PNa$ $[M+Na]^+$ 440.1386, found 440.1396.

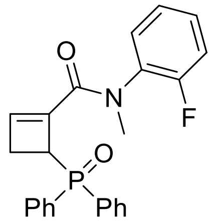

4-(Diphenylphosphoryl)-*N*-(2-fluorophenyl)-*N*-methylcyclobut-1-ene-1-carboxamide (3ea): Prepared according to the general procedure from **1e** (0.40 mmol) and **2a** (0.20 mmol), and purified by column chromatography on silica gel with petroleum ether/ethyl acetate/ethanol (10/1/1) to provide the title compound **3ea** (66.5 mg, 82% yield) as a white solid; R*f* 0.2 (petroleum ether/ethyl acetate/ethanol = 10/2/1); m.p. 136.9–138.7 °C; ^{1}H NMR (400 MHz, $CDCl_3$) δ 7.95–7.80 (m, 4H), 7.55–7.41 (m, 6H), 7.35–7.28 (m, 1H), 7.21–6.84 (m, 3H), 5.07 (s, 1H), 4.12 (s, 1H), 3.12 (s, 3H), 2.56–2.40 (m, 2H); ^{13}C NMR (100 MHz, $CDCl_3$) δ 161.6, 159.4 (d, $J_{C\text{-}P}$ = 12.3 Hz), 158.0 (d, $J_{C\text{-}F}$ = 247.5 Hz), 156.8 (d, $J_{C\text{-}P}$ = 5.2 Hz), 142.2 (d, $J_{C\text{-}P}$ = 3.4 Hz), 142.1 (d, $J_{C\text{-}P}$ = 3.6 Hz), 133.3 (d, $J_{C\text{-}P}$ = 95.6 Hz), 131.5 (d, $J_{C\text{-}F}$ = 9.4 Hz), 131.46, 131.1 (d, $J_{C\text{-}P}$ = 93.5 Hz), 130.7 (d, $J_{C\text{-}F}$ = 10.7 Hz), 129.8 (d, $J_{C\text{-}F}$ = 8.2 Hz), 129.7 (d, $J_{C\text{-}F}$ = 8.1 Hz), 128.4 (d, $J_{C\text{-}P}$ = 11.7 Hz), 128.0 (d, $J_{C\text{-}P}$ = 11.8 Hz), 116.6 (d, $J_{C\text{-}F}$ = 20.4 Hz), 116.2 (d, $J_{C\text{-}F}$ = 21.0 Hz), 41.3 (d, $J_{C\text{-}P}$ = 75.0 Hz), 36.5 (d, $J_{C\text{-}F}$ = 6.5 Hz), 27.8 (d, $J_{C\text{-}P}$ = 5.0 Hz); ^{19}F NMR (376 MHz, $CDCl_3$) δ -120.4, -122.0; ^{31}P NMR (162 MHz, $CDCl_3$) δ 29.0; HRMS (ESI) Calcd for $C_{24}H_{22}FNO_2P$ $[M + H]^+$ 406.1367, found 406.1375.

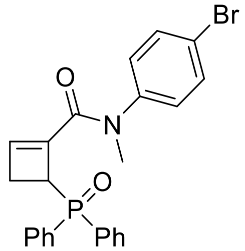

***N*-(4-BromoPhenyl)-4-(diphenylphosphoryl)-*N*-methylcyclobut-1-ene-1-carboxamide (3fa):** Prepared according to the general procedure from **1f** (0.40 mmol) and **2a** (0.20 mmol), and purified by column chromatography on silica gel with petroleum ether/ethyl acetate/ethanol (10/1/1) to provide the title compound **3fa** (67.4 mg, 73% yield) as a white solid; R*f* 0.2 (petroleum ether/ethyl acetate/ethanol = 10/2/1); m.p. 162.0–163.9 °C; ^{1}H NMR (400 MHz, $CDCl_3$) δ 7.90–7.80 (m, 4H), 7.61–7.44 (m, 8H), 6.90 (d, J = 8.3 Hz, 2H), 5.30 (s, 1H), 3.98 (s, 1H), 3.15 (s, 3H), 2.61–2.43 (m, 2H); ^{13}C NMR (100 MHz, $CDCl_3$) δ 161.8, 143.0 (d, $J_{C\text{-}P}$ = 12.6 Hz), 142.2, 138.3 (d, $J_{C\text{-}P}$ = 8.0 Hz), 132.9 (d, $J_{C\text{-}P}$ = 97.4 Hz), 132.5, 132.1 (d, $J_{C\text{-}P}$ = 98.8 Hz), 131.73 (d, $J_{C\text{-}P}$ = 3.1 Hz), 131.70 (d, $J_{C\text{-}P}$ = 3.0 Hz), 131.5 (d, $J_{C\text{-}P}$ = 9.5 Hz), 130.9 (d, $J_{C\text{-}}$

P = 9.1 Hz), 129.1, 128.5 (d, $J_{C\text{-}P}$ = 11.6 Hz), 128.3 (d, $J_{C\text{-}P}$ = 11.9 Hz), 121.4, 41.9 (d, $J_{C\text{-}P}$ = 72.7 Hz), 37.5, 28.3 (d, $J_{C\text{-}P}$ = 5.6 Hz); ^{31}P NMR (162 MHz, $CDCl_3$) δ 29.4; HRMS (ESI) Calcd for $C_{24}H_{22}BrNO_2P$ $[M + H]^+$ 466.0566, found 466.0575.

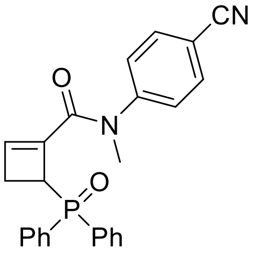

***N*-(4-Cyanophenyl)-4-(diphenylphosphoryl)-*N*-methylcyclobut-1-ene-1-carboxamide (3ga):** Prepared according to the general procedure from **1g** (0.40 mmol) and **2a** (0.20 mmol), and purified by column chromatography on silica gel with petroleum ether/ethyl acetate/ethanol (10/1/1) to provide the title compound **3ga** (56.2 mg, 68% yield) as a white solid; R_f 0.2 (petroleum ether/ethyl acetate/ethanol = 10/2/1); m.p. 158.4–160.4 °C; ^{1}H NMR (400 MHz, $CDCl_3$) δ 7.94–7.82 (m, 2H), 7.81–7.72 (m, 2H), 7.64 (d, J = 8.1 Hz, 2H), 7.58–7.42 (m, 6H), 7.25 (d, J = 8.7 Hz, 2H), 5.50 (s, 1H), 4.05 (s, 1H), 3.23 (s, 3H), 2.68–2.46 (m, 2H); ^{13}C NMR (100 MHz, $CDCl_3$) δ 161.8, 147.2, 142.8 (d, $J_{C\text{-}P}$ = 12.7 Hz), 137.9 (d, $J_{C\text{-}P}$ = 7.5 Hz), 133.1, 132.9 (d, $J_{C\text{-}P}$ = 97.4 Hz), 132.0 (d, $J_{C\text{-}P}$ = 98.7 Hz), 131.91 (d, $J_{C\text{-}P}$ = 4.7 Hz), 131.90 (d, $J_{C\text{-}P}$ = 4.6 Hz), 131.3 (d, $J_{C\text{-}P}$ = 9.7 Hz), 130.7 (d, $J_{C\text{-}P}$ = 9.1 Hz), 128.5 (d, $J_{C\text{-}P}$ = 11.7 Hz), 128.4 (d, $J_{C\text{-}P}$ = 11.9 Hz), 127.8, 118.1, 110.9, 41.9 (d, $J_{C\text{-}P}$ = 72.5 Hz), 37.3, 28.4 (d, $J_{C\text{-}P}$ = 6.2 Hz); ^{31}P NMR (162 MHz, $CDCl_3$) δ 29.5; HRMS (ESI) Calcd for $C_{25}H_{21}N_2O_2PNa$ $[M + Na]^+$ 435.1233, found 435.1240.

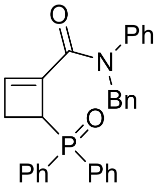

***N*-Benzyl-4-(diphenylphosphoryl)-*N*-phenylcyclobut-1-ene-1-carboxamide (3ha):** Prepared according to the general procedure from **1h** (0.40 mmol) and **2a** (0.20 mmol), and purified by column chromatography on silica gel with petroleum ether/ethyl acetate/ethanol (10/1/1) to provide the title compound **3ha** (72.4 mg, 78% yield) as a white solid; R_f 0.3 (petroleum ether/ethyl acetate/ethanol 10/2/1); m.p. 138.9–140.8 °C; ^{1}H NMR (400 MHz, $CDCl_3$) δ 7.97–7.80 (m, 4H), 7.54–7.42 (m, 6H), 7.25–7.16 (m, 6H), 7.09–6.98 (m, 2H), 6.86–6.76 (m, 2H), 5.16–4.92 (m, 2H), 4.49 (d, J = 14.4 Hz, 1H), 3.99 (s, 1H), 2.65–2.38 (m, 2H); ^{13}C NMR (100 MHz, $CDCl_3$) δ 161.2, 142.8 (d, $J_{C\text{-}P}$ = 13.5 Hz), 141.1, 138.2 (d, $J_{C\text{-}P}$ = 7.3 Hz), 136.6, 132.8 (d, $J_{C\text{-}P}$ = 98.0 Hz), 131.9 (d, $J_{C\text{-}P}$ = 96.0 Hz), 131.35 (d, $J_{C\text{-}P}$ = 2.9 Hz), 131.31 (d,

J_{C-P} = 3.0 Hz), 131.2, 130.7 (d, J_{C-P} = 9.2 Hz), 128.8, 128.4, 128.2 (d, J_{C-P} = 11.6 Hz), 128.0, 127.9 (d, J_{C-P} = 11.7 Hz), 127.7, 127.0, 52.8, 41.7 (d, J_{C-P} = 72.6 Hz), 27.8 (d, J_{C-P} = 5.7 Hz); ^{31}P NMR (162 MHz, $CDCl_3$) δ 29.3; HRMS (ESI) Calcd for $C_{30}H_{26}NO_2PNa$ $[M + Na]^+$ 486.1593, found 486.1603.

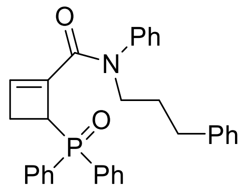

4-(Diphenylphosphoryl)-*N*-phenyl-*N*-(3-phenylpropyl)cyclobut-1-ene-1-carboxamide (3ia): Prepared according to the general procedure from **1i** (0.40 mmol) and **2a** (0.20 mmol), and purified by column chromatography on silica gel with petroleum ether/ethyl acetate/ethanol (10/1/1) to provide the title compound **3ia** (77.3 mg, 79% yield) as a colorless oil; R_f 0.2 (petroleum ether/ethyl acetate/ethanol = 10/2/1); ^{1}H NMR (400 MHz, $CDCl_3$) δ 7.89–7.81 (m, 4H), 7.52–7.42 (m, 6H), 7.35–7.29 (m, 3H), 7.25–7.19 (m, 2H), 7.17–7.10 (m, 1H), 7.07 (d, *J* = 7.5 Hz, 2H), 6.97 (d, *J* = 7.2 Hz, 2H), 5.08 (s, 1H), 4.03–3.87 (m, 2H), 3.38–3.26 (m, 1H), 2.56–2.40 (m, 4H), 1.74–1.60 (m, 2H); ^{13}C NMR (100 MHz, $CDCl_3$) δ 161.4, 142.3 (d, J_{C-P} = 11.6 Hz), 141.4, 138.6 (d, J_{C-P} = 8.1 Hz), 133.1 (d, J_{C-P} = 97.6 Hz), 131.9 (d, J_{C-P} = 95.9 Hz), 131.52 (d, J_{C-P} = 2.8 Hz), 131.50 (d, J_{C-P} = 3.2 Hz), 131.4 (d, J_{C-P} = 9.4 Hz), 130.8 (d, J_{C-P} = 9.3 Hz), 129.2, 128.5, 128.4 (d, J_{C-P} = 11.6 Hz), 128.2, 128.0 (d, J_{C-P} = 11.8 Hz), 127.9, 125.7, 48.9, 41.9 (d, J_{C-P} = 72.9 Hz), 32.8, 28.9, 27.9 (d, J_{C-P} = 5.6 Hz); ^{31}P NMR (162 MHz, $CDCl_3$) δ 29.6; HRMS (ESI) Calcd for $C_{32}H_{31}NO_2P$ $[M + H]^+$ 492.2087, found 492.2093.

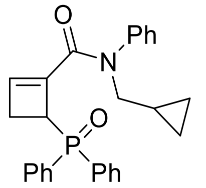

***N*-(Cyclopropylmethyl)-4-(diphenylphosphoryl)-*N*-phenylcyclobut-1-ene-1-carboxamide (3ja):** Prepared according to the general procedure from **1j** (0.40 mmol) and **2a** (0.20 mmol), and purified by column chromatography on silica gel with petroleum ether/ethyl acetate/ethanol (10/1/1) to provide the title compound **3ja** (75.2 mg, 88% yield) as a white solid; R_f 0.2 (petroleum ether/ethyl acetate/ethanol = 10/2/1); m.p. 111.1–112.2 °C; ^{1}H NMR (400 MHz, $CDCl_3$) δ 7.90–7.77 (m, 4H), 7.49–7.37 (m, 6H), 7.28–7.18 (m, 3H), 7.03–6.85 (m, 2H), 5.00 (s, 1H), 3.86 (s, 1H), 3.63 (dd, *J* = 13.8, 7.4 Hz, 1H), 3.17 (dd, *J* = 13.8, 7.0 Hz, 1H), 2.58–2.42 (m, 1H), 2.41–2.31 (m, 1H), 0.83–0.73 (m, 1H), 0.34–0.25 (m, 2H), 0.09–-0.11 (m, 2H); ^{13}C NMR (100 MHz, $CDCl_3$) δ 161.3, 142.4 (d, J_{C-P} = 13.0 Hz), 141.8, 138.8 (d, J_{C-P} = 8.0 Hz),

133.1 (d, J_{C-P} = 97.5 Hz), 132.0 (d, J_{C-P} = 98.5 Hz), 131.52 (d, J_{C-P} = 9.6 Hz), 131.50 (d, J_{C-P} = 3.4 Hz), 131.4 (d, J_{C-P} = 2.9 Hz), 130.9 (d, J_{C-P} = 9.2 Hz), 129.0, 128.7, 128.3 (d, J_{C-P} = 11.5 Hz), 127.9 (d, J_{C-P} = 11.7 Hz), 127.8, 53.5, 41.9 (d, J_{C-P} = 72.6 Hz), 27.8 (d, J_{C-P} = 5.7 Hz), 9.4, 3.7, 3.2; ^{31}P NMR (162 MHz, $CDCl_3$) δ 29.3; HRMS (ESI) Calcd for $C_{27}H_{26}NO_2PNa$ [M + Na]$^+$ 450.1593 found 450.1600.

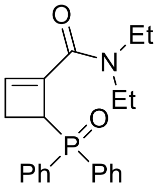

4-(Diphenylphosphoryl)-*N*,*N*-diethylcyclobut-1-ene-1-carboxamide (3ka): Prepared according to the general procedure from **1k** (0.40 mmol) and **2a** (0.20 mmol), and purified by column chromatography on silica gel with petroleum ether/ethyl acetate/ethanol (10/1/1) to provide the title compound **3ka** (62.5 mg, 89% yield) as a white solid; R_f 0.2 (petroleum ether/ethyl acetate/ethanol = 10/2/1); m.p. 138.5–139.5 °C; ^{1}H NMR (400 MHz, $CDCl_3$) δ 7.90–7.71 (m, 4H), 7.59–7.37 (m, 6H), 6.27 (s, 1H), 4.32 (d, *J* = 4.5 Hz, 1H), 3.77 (dd, *J* = 14.7, 7.3 Hz, 1H), 3.40 (dd, *J* = 13.7, 7.0 Hz, 1H), 3.09 (dd, *J* = 14.6, 7.2 Hz, 1H), 3.02–2.84 (m, 2H), 2.68 (dt, *J* = 14.7, 4.3 Hz, 1H), 1.11 (t, *J* = 7.1 Hz, 3H), 0.82 (t, *J* = 7.1 Hz, 3H); ^{13}C NMR (100 MHz, $CDCl_3$) δ 163.4, 138.4 (d, J_{C-P} = 8.0 Hz), 136.1 (d, J_{C-P} = 13.0 Hz), 132.9 (d, J_{C-P} = 97.5 Hz), 131.8 (d, J_{C-P} = 98.5 Hz), 131.72 (d, J_{C-P} = 3.2 Hz), 131.70 (d, J_{C-P} = 3.3 Hz), 131.2 (d, J_{C-P} = 9.6 Hz), 130.6 (d, J_{C-P} = 9.4 Hz), 128.6 (d, J_{C-P} = 11.5 Hz), 128.3 (d, J_{C-P} = 11.8 Hz), 42.4, 42.36 (d, J_{C-P} = 72.6 Hz), 39.2, 27.9 (d, J_{C-P} = 5.7 Hz), 14.8, 12.3; ^{31}P NMR (162 MHz, $CDCl_3$) δ 28.3; HRMS (ESI) Calcd for $C_{21}H_{25}NO_2P$ [M + H]$^+$ 354.1617, found 354.1627.

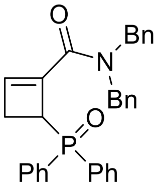

***N*,*N*-Dibenzyl-4-(diphenylphosphoryl)cyclobut-1-ene-1-carboxamide (3la):** Prepared according to the general procedure from **1l** (0.40 mmol) and **2a** (0.20 mmol), and purified by column chromatography on silica gel with petroleum ether/ethyl acetate/ethanol (20/1/1) to provide the title compound **3la** (71.8 mg, 75% yield) as a white solid; R_f 0.3 (petroleum ether/ethyl acetate/ethanol = 10/2/1); m.p. 112.6–114.9 °C; ^{1}H NMR (400 MHz, $CDCl_3$) δ 7.93–7.75 (m, 4H), 7.56–7.38 (m, 6H), 7.33 (t, *J* = 7.4 Hz, 2H), 7.28 (s, 1H), 7.24–7.09 (m, 5H), 6.91–6.78 (m, 2H), 6.10 (s, 1H), 5.15 (d, *J* = 14.7 Hz, 1H), 4.94 (d, *J* = 17.2 Hz, 1H), 4.43 (dd, *J* = 4.8, 1.9 Hz, 1H), 4.13 (d, *J* = 17.2 Hz, 1H), 3.76 (d, *J* = 14.7 Hz, 1H), 2.84–2.73 (m, 1H),

2.70–2.59 (m, 1H); ^{13}C NMR (100 MHz, $CDCl_3$) δ 164.3, 138.8 (d, $J_{C\text{-}P}$ = 12.7 Hz), 137.5 (d, $J_{C\text{-}P}$ = 8.0 Hz), 136.9, 136.3, 132.9 (d, $J_{C\text{-}P}$ = 97.7 Hz), 131.81 (d, $J_{C\text{-}P}$ = 98.8 Hz), 131.80 (d, $J_{C\text{-}P}$ = 2.8Hz), 131.72 (d, $J_{C\text{-}P}$ = 2.9 Hz), 131.70 (d, $J_{C\text{-}P}$ = 9.5 Hz), 130.4 (d, $J_{C\text{-}P}$ = 9.4 Hz), 128.8, 128.6 (d, $J_{C\text{-}P}$ = 11.6 Hz), 128.44, 128.40 (d, $J_{C\text{-}P}$ = 12.4 Hz), 128.2, 127.3, 127.2, 126.3, 50.4, 47.5, 41.9 (d, $J_{C\text{-}P}$ = 72.5 Hz), 27.8 (d, $J_{C\text{-}P}$ = 5.5 Hz); ^{31}P NMR (162 MHz, $CDCl_3$) δ 29.1; HRMS (ESI) Calcd for $C_{31}H_{29}NO_2P$ $[M + H]^+$ 478.1930, found 478.1925.

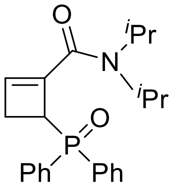

4-(Diphenylphosphoryl)-*N*,*N*-diisopropylcyclobut-1-ene-1-carboxamide (3ma): Prepared according to the general procedure from **1m** (0.40 mmol) and **2a** (0.20 mmol), and purified by column chromatography on silica gel with petroleum ether/ethyl acetate/ethanol (20/1/1) to provide the title compound **3ma** (54.1 mg, 71% yield) as a white solid; R_f 0.3 (petroleum ether/ethyl acetate/ethanol = 10/2/1); m.p. 181.3–183.4 °C; ^{1}H NMR (400 MHz, $CDCl_3$) δ 7.85 (dd, J = 11.5, 7.4 Hz, 2H), 7.74 (dd, J = 11.4, 7.5 Hz, 2H), 7.46 (m, 6H), 6.14 (s, 1H), 4.71–4.57 (m, 1H), 4.32 (d, J = 4.5 Hz, 1H), 3.33–3.16 (m, 1H), 2.87 (dd, J = 14.5, 7.1 Hz, 1H), 2.64 (dt, J = 14.5, 4.5 Hz, 1H), 1.32 (d, J = 6.8 Hz, 3H), 1.11 (d, J = 6.7 Hz, 3H), 1.03–0.89 (m, 6H); ^{13}C NMR (100 MHz, $CDCl_3$) δ 163.6, 139.3 (d, $J_{C\text{-}P}$ = 8.1 Hz), 134.2 (d, $J_{C\text{-}P}$ = 13.2 Hz), 133.0 (d, $J_{C\text{-}P}$ = 97.0 Hz), 131.8 (d, $J_{C\text{-}P}$ = 98.3 Hz), 131.7 (d, $J_{C\text{-}P}$ = 2.8 Hz), 131.6 (d, $J_{C\text{-}P}$ = 2.7 Hz), 131.4 (d, $J_{C\text{-}P}$ = 9.6 Hz), 130.5 (d, $J_{C\text{-}P}$ = 9.4 Hz), 128.6 (d, $J_{C\text{-}P}$ = 11.5 Hz), 128.4 (d, $J_{C\text{-}P}$ = 11.7 Hz), 49.9, 45.5, 42.1 (d, $J_{C\text{-}P}$ = 72.7 Hz), 27.7 (d, $J_{C\text{-}P}$ = 5.4 Hz), 21.3, 20.4, 20.1, 20.0; ^{31}P NMR (162 MHz, $CDCl_3$) δ 27.9; HRMS (ESI) Calcd for $C_{23}H_{28}NO_2PNa$ $[M + Na]^+$ 404.1750, found 404.1756.

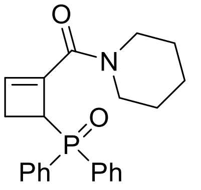

(4-(Diphenylphosphoryl)cyclobut-1-en-1-yl)(piperidin-1-yl)methanone (3na): Prepared according to the general procedure from **1n** (0.40 mmol) and **2a** (0.20 mmol), and purified by column chromatography on silica gel with petroleum ether/ethyl acetate/ethanol (10/1/1) to provide the title compound **3na** (62.8 mg, 86% yield) as a white solid; R_f 0.2 (petroleum ether/ethyl acetate/ethanol = 10/2/1); m.p. 182.6–184.4 °C; ^{1}H NMR (400 MHz, $CDCl_3$) δ 7.83 (dd, J = 11.6, 7.4 Hz, 2H), 7.76 (dd, J = 11.5, 7.4 Hz, 2H), 7.58–7.39 (m, 6H), 6.21 (s, 1H),

4.30 (d, J = 4.5 Hz, 1H), 3.62–3.45 (m, 2H), 3.41–3.32 (m, 1H), 3.30–3.19 (m, 1H), 2.95–2.84 (m, 1H), 2.74–2.63 (m, 1H), 1.57–1.59 (m, 2H), 1.48–1.34 (m, 3H), 1.26–1.16 (m, 1H); ^{13}C NMR (100 MHz, $CDCl_3$) δ 162.4, 138.0 (d, $J_{C\text{-}P}$ = 6.5 Hz), 136.9 (d, $J_{C\text{-}P}$ = 13.1 Hz), 132.8 (d, $J_{C\text{-}P}$ = 97.5 Hz), 131.8 (d, $J_{C\text{-}P}$ = 2.8 Hz), 131.7 (d, $J_{C\text{-}P}$ = 2.8 Hz), 131.6 (d, $J_{C\text{-}P}$ = 98.8 Hz), 131.3 (d, $J_{C\text{-}P}$ = 9.4 Hz), 130.6 (d, $J_{C\text{-}P}$ = 9.4 Hz), 128.6 (d, $J_{C\text{-}P}$ = 11.5 Hz), 128.3 (d, $J_{C\text{-}P}$=11.8 Hz), 47.5, 42.4 (d, $J_{C\text{-}P}$ = 72.7 Hz), 42.2, 28.2 (d, $J_{C\text{-}P}$ = 5.5 Hz), 26.3, 25.2, 24.3; ^{31}P NMR (162 MHz, $CDCl_3$) δ 28.4; HRMS (ESI) Calcd for $C_{22}H_{24}NO_2PNa$ $[M + Na]^+$ 388.1437, found 388.1447.

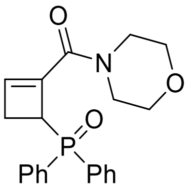

(4-(Diphenylphosphoryl)cyclobut-1-en-1-yl)(morpholino)methanone (3oa): Prepared according to the general procedure from **1o** (0.40 mmol) and **2a** (0.20 mmol), and purified by column chromatography on silica gel with petroleum ether/ethyl acetate/ethanol (10/1/1) to provide the title compound **3oa** (58.1 mg, 79% yield) as a white solid; R*f* 0.1 (petroleum ether/ethyl acetate/ethanol = 10/2/1); m.p. 173.1–175.3 °C; ^{1}H NMR (400 MHz, $CDCl_3$) δ 7.84 (dd, J = 11.5, 7.4 Hz, 2H), 7.73 (dd, J = 11.4, 7.4 Hz, 2H), 7.58–7.44 (m, 6H), 6.26 (s, 1H), 4.32 (d, J = 4.5 Hz, 1H), 3.80 (d, J = 12.9 Hz, 1H), 3.62–3.56 (m, 2H), 3.54–3.46 (m, 1H), 3.34 (t, J = 10.1 Hz, 1H), 3.16 (d, J = 9.7 Hz, 2H), 2.92 (dd, J = 14.7, 6.7 Hz, 1H), 2.76–2.63 (m, 2H); ^{13}C NMR (100 MHz, $CDCl_3$) δ 162.7, 137.7 (d, $J_{C\text{-}P}$ = 12.8 Hz), 137.2 (d, $J_{C\text{-}P}$ = 8.3 Hz), 132.5 (d, $J_{C\text{-}P}$ = 92.2 Hz), 132.0 (d, $J_{C\text{-}P}$ = 3.2 Hz), 131.6 (d, $J_{C\text{-}P}$ = 91.7 Hz), 131.9 (d, $J_{C\text{-}P}$ = 2.8 Hz), 131.2 (d, $J_{C\text{-}P}$ = 9.3 Hz), 130.5 (d, $J_{C\text{-}P}$ = 9.2 Hz), 128.7 (d, $J_{C\text{-}P}$ = 11.4 Hz), 128.4 (d, $J_{C\text{-}P}$ = 11.7 Hz), 66.7, 66.3, 46.9, 42.3 (d, $J_{C\text{-}P}$ = 72.3 Hz), 41.4, 28.3 (d, $J_{C\text{-}P}$ = 5.0 Hz); ^{31}P NMR (162 MHz, $CDCl_3$) δ 28.4; HRMS (ESI) Calcd for $C_{21}H_{22}NO_3PNa$ $[M + Na]^+$ 390.1230, found 390.1240.

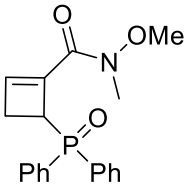

4-(Diphenylphosphoryl)-*N*-methoxy-*N*-methylcyclobut-1-ene-1-carboxamide (3pa): Prepared according to the general procedure from **1p** (0.40 mmol) and **2a** (0.20 mmol), and purified by column chromatography on silica gel with petroleum ether/ethyl acetate/ethanol (5/1/1) to provide the title compound **3pa** (58.30 mg, 86% yield) as a white solid; R*f* 0.1

(petroleum ether/ethyl acetate/ethanol = 10/2/1); m.p. 142.2–143.5 °C; ^{1}H NMR (400 MHz, $CDCl_3$) δ 7.91–7.71 (m, 4H), 7.54–7.37 (m, 6H), 6.60 (s, 1H), 4.20 (s, 1H), 3.56 (s, 3H), 3.08 (s, 3H), 2.86–2.62 (m, 2H); ^{13}C NMR (100 MHz, $CDCl_3$) δ 161.2, 144.1 (d, J_{C-P} = 12.0 Hz), 136.8 (d, J_{C-P} = 8.0 Hz), 133.2 (d, J_{C-P} = 98.0 Hz), 131.9 (d, J_{C-P} = 96.0 Hz), 131.5 (d, J_{C-P} = 2.2 Hz), 131.4 (d, J_{C-P} = 2.1 Hz), 131.3 (d, J_{C-P} = 9.0 Hz), 130.7 (d, J_{C-P} = 9.3 Hz), 128.3 (d, J_{C-P} = 11.6 Hz), 127.9 (d, J_{C-P} = 11.8 Hz), 61.0, 41.5 (d, J_{C-P} = 72.7 Hz), 32.5, 28.7 (d, J_{C-P} = 5.5 Hz); ^{31}P NMR (162 MHz, $CDCl_3$) δ 29.3; HRMS (ESI) Calcd for $C_{19}H_{20}NO_3PNa$ $[M + Na]^+$ 364.1073, found 364.1082.

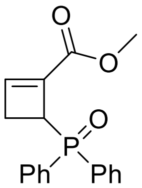

Methyl 4-(diphenylphosphoryl)cyclobut-1-ene-1-carboxylate (3qa): Prepared according to the general procedure from **1q** (0.40 mmol) and **2a** (0.20 mmol), and purified by column chromatography on silica gel with petroleum ether/ethyl acetate/ethanol (10/1/1) to provide the title compound **3qa** (58.8 mg, 94% yield) as a white solid; R*f* 0.2 (petroleum ether/ethyl acetate/ethanol = 10/2/1); m.p. 136.3–138.2 °C; ^{1}H NMR (400 MHz, $CDCl_3$) δ 7.89–7.73 (m, 4H), 7.56–7.43 (m, 6H), 6.93 (s, 1H), 4.18–4.05 (m, 1H), 3.41 (s, 3H), 2.87–2.65 (m, 2H); ^{13}C NMR (100 MHz, $CDCl_3$) δ 161.2, 148.3 (d, J_{C-P} = 12.8 Hz), 135.4 (d, J_{C-P} = 8.5 Hz), 133.0 (d, J_{C-P} = 98.3 Hz), 131.8 (d, J_{C-P} = 2.8 Hz), 131.7 (d, J_{C-P} = 2.8 Hz), 131.6 (d, J_{C-P} = 99.8 Hz), 131.4 (d, J_{C-P} = 9.3 Hz), 130.7 (d, J_{C-P} = 9.2 Hz),128.6 (d, J_{C-P} = 11.5 Hz), 128.1 (d, J_{C-P} = 11.9 Hz), 51.2, 41.1 (d, J_{C-P} = 73.0 Hz), 28.9 (d, J_{C-P} = 5.3 Hz); ^{31}P NMR (162 MHz, $CDCl_3$) δ 27.7; HRMS (ESI) Calcd for $C_{18}H_{17}O_3PNa$ $[M + Na]^+$ 335.0808, found 335.0816.

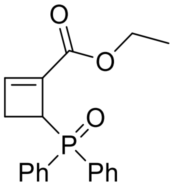

Ethyl 4-(diphenylphosphoryl)cyclobut-1-ene-1-carboxylate (3ra): Prepared according to the general procedure from **1r** (0.40 mmol) and **2a** (0.20 mmol), and purified by column chromatography on silica gel with petroleum ether/ethyl acetate/ethanol (10/1/1) to provide the title compound **3ra** (64.2 mg, 99% yield) as a white solid; R*f* 0.2 (petroleum ether/ethyl acetate/ethanol = 10/2/1); m.p. 144.6–146.6 °C; ^{1}H NMR (400 MHz, $CDCl_3$) δ 7.86–7.75 (m, 4H), 7.54–7.43 (m, 6H), 6.92 (s, 1H), 4.13 (s, 1H), 4.02–3.94 (m, 1H), 3.91–3.80 (m, 1H), 2.83–2.64 (m, 2H), 0.98 (t, J = 7.1 Hz, 3H); ^{13}C NMR (100 MHz, $CDCl_3$) δ 160.8, 148.0 (d, J_{C-P} =

12.9 Hz), 135.8 (d, $J_{C\text{-}P}$ = 8.8 Hz), 133.2 (d, $J_{C\text{-}P}$ = 98.2 Hz), 131.69 (d, $J_{C\text{-}P}$ = 99.7 Hz), 131.65 (d, $J_{C\text{-}P}$ = 2.2 Hz), 131.63 (d, $J_{C\text{-}P}$ = 2.3 Hz), 131.4 (d, $J_{C\text{-}P}$ = 9.3 Hz), 130.7 (d, $J_{C\text{-}P}$ = 9.2 Hz), 128.5 (d, $J_{C\text{-}P}$ = 11.4 Hz), 128.1 (d, $J_{C\text{-}P}$ = 11.8 Hz), 60.2, 40.8 (d, $J_{C\text{-}P}$ = 73.3 Hz), 28.8 (d, $J_{C\text{-}P}$ = 5.1 Hz), 13.8; ^{31}P NMR (162 MHz, $CDCl_3$) δ 28.0; HRMS (ESI) Calcd for $C_{19}H_{19}O_3PNa$ [M + Na]$^+$ 349.0964, found 349.0971.

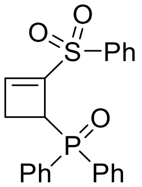

Diphenyl(2-(phenylsulfonyl)cyclobut-2-en-1-yl)phosphine oxide (3sa): Prepared according to the general procedure from **1s** (0.40 mmol) and **2a** (0.20 mmol), and purified by column chromatography on silica gel with petroleum ether/ethyl acetate/ethanol (10/1/1) to provide the title compound **3sa** (65.3 mg, 83% yield) as a white solid; R_f 0.2 (petroleum ether/ethyl acetate/ethanol = 10/2/1); m.p. 120.6–122.6 °C; ^{1}H NMR (400 MHz, $CDCl_3$) δ 7.74 (d, J = 7.8 Hz, 2H), 7.70–7.58 (m, 5H), 7.57–7.49 (m, 4H), 7.48–7.40 (m, 4H), 6.71 (s, 1H), 3.86–3.78 (m, 1H), 3.12 (dt, J = 13.9, 4.2 Hz, 1H), 2.81 (dd, J = 13.8, 7.1 Hz, 1H); ^{13}C NMR (100 MHz, $CDCl_3$) δ 146.0 (d, $J_{C\text{-}P}$ = 15.5 Hz), 140.7 (d, $J_{C\text{-}P}$ = 7.6 Hz), 138.2, 133.8, 132.43 (d, $J_{C\text{-}P}$ = 2.5 Hz), 132.41 (d, $J_{C\text{-}P}$ = 2.5 Hz), 131.1 (d, $J_{C\text{-}P}$ = 100.5 Hz), 131.0 (d, $J_{C\text{-}P}$ = 9.2 Hz), 130.8 (d, $J_{C\text{-}P}$ = 9.4 Hz), 130.2 (d, $J_{C\text{-}P}$ = 100.7 Hz), 129.3, 128.8 (d, $J_{C\text{-}P}$ = 7.3 Hz), 128.7 (d, $J_{C\text{-}P}$ = 7.3 Hz), 127.9, 39.2 (d, $J_{C\text{-}P}$ = 70.4 Hz), 31.3 (d, $J_{C\text{-}P}$ = 4.2 Hz); ^{31}P NMR (162 MHz, $CDCl_3$) δ 29.3; HRMS (ESI) Calcd for $C_{22}H_{19}O_3PSNa$ [M + Na]$^+$ 417.0685, found 417.0691.

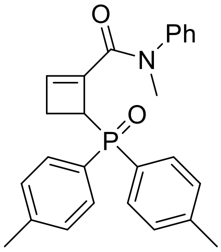

4-(Di-*p*-tolylphosphoryl)-*N*-methyl-*N*-phenylcyclobut-1-ene-1-carboxamide (3ab): Prepared according to the general procedure from **1a** (0.40 mmol) and **2b** (0.20 mmol), and purified by column chromatography on silica gel with petroleum ether/ethyl acetate/ethanol (10/1/1) to provide the title compound **3ab** (36.7 mg, 44% yield) as a white solid; R_f 0.2 (petroleum ether/ethyl acetate/ethanol = 10/2/1); m.p. 124.5–126.2 °C; ^{1}H NMR (400 MHz, $CDCl_3$) δ 7.78–7.65 (m, 4H), 7.38–7.27 (m, 5H), 7.24 (s, 2H), 6.98 (d, J = 7.4 Hz, 2H), 5.22 (s, 1H), 3.80 (s, 1H), 3.16 (s, 3H), 2.42 (s, 2H), 2.40 (s, 3H), 2.38 (s, 3H); ^{13}C NMR (100 MHz, $CDCl_3$) δ 162.0, 142.3 (d, $J_{C\text{-}P}$ = 14.2 Hz), 143.2, 141.8 (d, $J_{C\text{-}P}$ = 2.1 Hz), 138.9 (d, $J_{C\text{-}P}$ = 8.2

Hz), 131.5 (d, J_{C-P} = 9.7 Hz), 130.9 (d, J_{C-P} = 9.5 Hz), 130.1 (d, J_{C-P} = 99.5 Hz), 129.2, 129.1 (d, J_{C-P} = 11.9 Hz), 128.9 (d, J_{C-P} = 101.2 Hz), 128.8 (d, J_{C-P} = 12.1 Hz), 127.7, 127.4, 42.1 (d, J_{C-P} = 72.7 Hz), 37.5, 28.2 (d, J_{C-P} = 5.5 Hz), 21.6, 21.5; ^{31}P NMR (162 MHz, $CDCl_3$) δ 29.4; HRMS (ESI) Calcd for $C_{26}H_{26}NO_2PNa$ $[M + Na]^+$ 438.1593, found 438.1601.

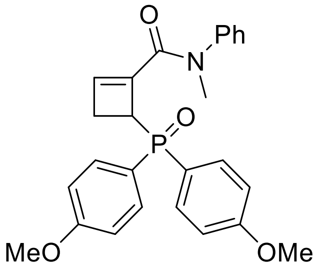

4-(Bis(4-methoxyphenyl)phosphoryl)-*N*-methyl-*N*-phenylcyclobut-1-ene-1-carboxamide (3ac): Prepared according to the general procedure from **1a** (0.40 mmol) and **2c** (0.20 mmol), and purified by column chromatography on silica gel with petroleum ether/ethyl acetate/ethanol (10/1/1) to provide the title compound **3ac** (64.2 mg, 72% yield) as a white solid; R_f 0.2 (petroleum ether/ethyl acetate/ethanol = 10/2/1); m.p. 122.3–123.9 °C; ^{1}H NMR (400 MHz, $CDCl_3$) δ 7.76–7.63 (m, 4H), 7.25 (app. s, 2H), 7.09–6.87 (m, 7H), 4.62 (s, 1H), 4.28 (s, 1H), 3.85 (s, 3H), 3.83 (s, 3H), 3.30 (s, 3H), 2.50–2.39 (m, 1H), 2.28 (s, 1H); ^{13}C NMR (100 MHz, $CDCl_3$) δ 162.6, 161.4, 143.4 (d, J_{C-P} = 11.1 Hz), 142.6, 138.9 (d, J_{C-P} = 8.7 Hz), 134.0 (d, J_{C-P} = 10.0 Hz), 132.3 (d, J_{C-P} = 98.0 Hz), 133.2 (d, J_{C-P} = 10.9 Hz), 130.7 (d, J_{C-P} = 98.2 Hz), 129.50 (d, J_{C-P} = 6.9 Hz), 129.45 (d, J_{C-P} = 6.1 Hz), 128.4, 127.6, 114.2 (d, J_{C-P} = 12.8 Hz), 113.9 (d, J_{C-P} = 12.7 Hz), 55.4, 55.3, 42.4 (d, J_{C-P} = 72.5 Hz), 38.4, 28.9 (d, J_{C-P} = 3.2 Hz); ^{31}P NMR (162 MHz, $CDCl_3$) δ 35.3; HRMS (ESI) Calcd for $C_{26}H_{27}NO_4P$ $[M + H]^+$ 448.1672, found 448.1681.

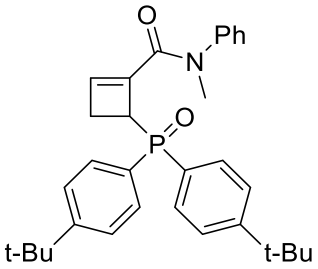

4-(Bis(4-(*tert*-butyl)phenyl)phosphoryl)-*N*-methyl-*N*-phenylcyclobut-1-ene-1-carboxamide (3ad): Prepared according to the general procedure from **1a** (0.40 mmol) and **2d** (0.20 mmol), and purified by column chromatography on silica gel with petroleum ether/ethyl acetate/ethanol (20/1/1) to provide the title compound **3ad** (76.3 mg, 76% yield) as a white solid; R_f 0.3 (petroleum ether/ethyl acetate/ethanol = 10/2/1); m.p. 92.3–94.3 °C; ^{1}H NMR (400 MHz, $CDCl_3$) δ 7.78 (dd, J = 11.2, 8.0 Hz, 4H), 7.52–7.47 (m, 4H), 7.30–7.26 (m, 3H), 6.84–6.77 (m, 2H), 5.34 (s, 1H), 3.68 (s, 1H), 3.15 (s, 3H), 2.62–2.39 (m, 2H), 1.34 (s, 9H), 1.33 (s, 9H); ^{13}C NMR (100 MHz, $CDCl_3$) δ 162.3, 154.8, 143.1, 142.5 (d, J_{C-P} = 14.6 Hz),

139.3 (d, $J_{C\text{-}P}$ = 7.5 Hz), 131.5 (d, $J_{C\text{-}P}$ = 9.6 Hz), 131.0 (d, $J_{C\text{-}P}$ = 9.5 Hz), 130.0 (d, $J_{C\text{-}P}$ = 99.8 Hz), 129.2, 128.8 (d, $J_{C\text{-}P}$ = 99.0 Hz), 127.6, 127.3 (d, $J_{C\text{-}P}$ = 3.7 Hz), 127.2 (d, $J_{C\text{-}P}$ = 3.9 Hz), 125.4 (d, $J_{C\text{-}P}$ = 11.9 Hz), 125.1 (d, $J_{C\text{-}P}$ = 11.7 Hz), 42.6 (d, $J_{C\text{-}P}$ = 72.6 Hz), 37.4, 35.0, 31.2, 31.1, 28.3 (d, $J_{C\text{-}P}$ = 5.4 Hz); ^{31}P NMR (162 MHz, $CDCl_3$) δ 29.4; HRMS (ESI) Calcd for $C_{32}H_{38}NO_2PNa$ $[M + Na]^+$ 522.2532, found 522.2541.

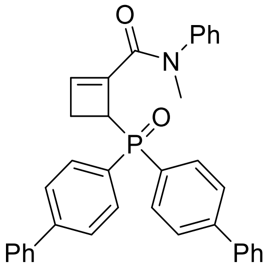

4-(Di([1,1'-biphenyl]-4-yl)phosphoryl)-*N*-methyl-*N*-phenylcyclobut-1-ene-1-carboxamide (3ae): Prepared according to the general procedure from **1a** (0.40 mmol) and **2e** (0.20 mmol), and purified by column chromatography on silica gel with petroleum ether/ethyl acetate/ethanol (10/1/1) to provide the title compound **3ae** (52.6mg, 49% yield) as a white solid; R_f 0.2 (petroleum ether/ethyl acetate/ethanol = 10/2/1); m.p. 121.5–123.5 °C; ^{1}H NMR (400 MHz, $CDCl_3$) δ 8.01–7.92 (m, 4H), 7.75–7.69 (m, 4H), 7.65–7.56 (m, 4H), 7.48–7.43 (m, 4H), 7.41–7.34 (m, 2H), 7.33–7.27 (m, 3H), 6.98 (d, J = 6.8 Hz, 2H), 5.21 (s, 1H), 3.91 (s, 1H), 3.18 (s, 3H), 2.62–2.46 (m, 2H); ^{13}C NMR (100 MHz, $CDCl_3$) δ 162.0, 144.4 (d, $J_{C\text{-}P}$ = 2.8 Hz), 143.1, 140.1 (d, $J_{C\text{-}P}$ = 10.3 Hz), 138.9 (d, $J_{C\text{-}P}$ = 8.0 Hz), 132.1 (d, $J_{C\text{-}P}$ = 9.7 Hz), 131.8 (d, $J_{C\text{-}P}$ = 98.7 Hz), 131.5 (d, $J_{C\text{-}P}$ = 9.6 Hz), 130.6 (d, $J_{C\text{-}P}$ = 99.8 Hz), 129.3, 128.9, 128.0, 127.9, 127.8, 127.4, 127.21 (d, $J_{C\text{-}P}$ = 2.2 Hz), 127.20, (d, $J_{C\text{-}P}$ = 12.4 Hz), 126.9 (d, $J_{C\text{-}P}$ = 12.1 Hz), 42.0 (d, $J_{C\text{-}P}$ = 72.8 Hz), 37.5, 28.2 (d, $J_{C\text{-}P}$ = 5.5 Hz); ^{31}P NMR (162 MHz, $CDCl_3$) δ 29.6; HRMS (ESI) Calcd for $C_{36}H_{31}NO_2P$ $[M + H]^+$ 540.2087, found 540.2092.

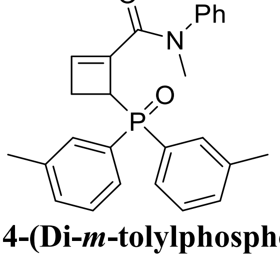

4-(Di-*m*-tolylphosphoryl)-*N*-methyl-*N*-phenylcyclobut-1-ene-1-carboxamide (3af): Prepared according to the general procedure from **1a** (0.40 mmol) and **2f** (0.20 mmol), and purified by column chromatography on silica gel with petroleum ether/ethyl acetate/ethanol (10/1/1) to provide the title compound **3af** (71.5mg, 86% yield) as a white solid; R_f 0.2 (petroleum ether/ethyl acetate/ethanol = 10/2/1); m.p. 128.7–130.8 °C; ^{1}H NMR (400 MHz, $CDCl_3$) δ 7.74–7.58 (m, 4H), 7.41–7.26 (m, 7H), 6.97–6.84 (m, 2H), 5.25 (s, 1H), 3.78 (s, 1H),

3.16 (s, 3H), 2.57–2.42 (m, 2H), 2.40 (s, 3H), 2.38 (s, 3H); ^{13}C NMR (100 MHz,) δ 162.0, 143.2, 142.5 (d, J_{C-P} = 13.6 Hz), 138.9 (d, J_{C-P} = 7.9 Hz), 138.2 (d, J_{C-P} = 11.4 Hz), 137.9 (d, J_{C-P} = 11.6 Hz), 133.0 (d, J_{C-P} = 96.9 Hz), 132.3 (d, J_{C-P} = 2.6 Hz), 132.2 (d, J_{C-P} = 2.9 Hz), 132.0 (d, J_{C-P} = 8.9 Hz), 131.9 (d, J_{C-P} = 95.6 Hz), 131.5 (d, J_{C-P} = 8.6 Hz), 129.2, 128.5 (d, J_{C-P} = 9.8 Hz), 128.2 (d, J_{C-P} = 12.3 Hz), 127.9 (d, J_{C-P} = 12.5 Hz), 127.8 (d, J_{C-P} = 9.7 Hz), 127.6, 127.3, 42.1 (d, J_{C-P} = 72.7 Hz), 37.4, 28.2 (d, J_{C-P} = 5.6 Hz), 21.4; ^{31}P NMR (162 MHz, $CDCl_3$) δ 29.2; HRMS (ESI) Calcd for $C_{26}H_{26}NO_2PNa$ $[M + Na]^+$ 438.1593, found 438.1601.

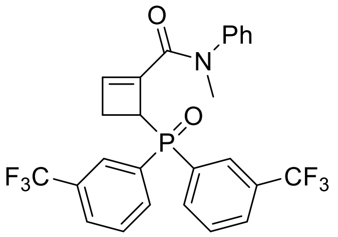

4-(Bis(3-(trifluoromethyl)phenyl)phosphoryl)-*N*-methyl-*N*-phenylcyclobut-1-ene-1-carboxamide (3ag): Prepared according to the general procedure from **1a** (0.40 mmol) and **2g** (0.20 mmol), and purified by column chromatography on silica gel with petroleum ether/ethyl acetate/ethanol (10/1/1) to provide the title compound **3ag** (47.1mg, 45% yield) as a white solid; R_f 0.3 (petroleum ether/ethyl acetate/ethanol = 10/2/1); m.p. 108.2–109.1 °C; ^{1}H NMR (400 MHz, $CDCl_3$) δ 8.22–8.05 (m, 4H), 7.85–7.79 (m, 2H), 7.69–7.63 (m, 2H), 7.40–7.32 (m, 3H), 7.03–6.97 (m, 2H), 4.94 (s, 1H), 4.02 (s, 1H), 3.16 (s, 3H), 2.54–2.43 (m, 2H); ^{13}C NMR (100 MHz, $CDCl_3$) δ 160.1, 143.2 (d, J_{C-P} = 15.5 Hz), 142.9, 138.0 (d, J_{C-P} = 8.0 Hz), 134.7 (dd, J_{C-F} = 9.2 Hz, J_{C-P} = 2.3 Hz), 134.1 (d, J_{C-P} = 98.0 Hz), 134.0 (dd, J_{C-F} = 9.4 Hz, J_{C-P} = 2.4 Hz), 132.9 (d, J_{C-P} = 98.5 Hz), 131.3 (d, J_{C-P} = 12.2 Hz), 130.6 (d, J_{C-P} = 12.9 Hz), 129.5, 129.2 (dd, J_{C-F} = 16.6 Hz, J_{C-P} = 11.5 Hz), 128.7 (dd, J_{C-F} = 16.8 Hz, J_{C-P} = 11.8 Hz), 128.60 (d, J_{C-P} = 7.3 Hz), 128.56 (d, J_{C-P} = 7.3 Hz), 128.3 (dd, J_{C-F} = 10.2 Hz, J_{C-P} = 4.1 Hz), 128.1, 127.8 (dd, J_{C-F} = 9.4 Hz, J_{C-P} = 3.9 Hz), 127.4, 123.5 (q, J_{C-F} = 271.2 Hz), 123.3 (q, J_{C-F} = 272.5 Hz), 41.3 (d, J_{C-P} = 74.0 Hz), 37.5, 27.5 (d, J_{C-P} = 5.7 Hz); ^{31}P NMR (162 MHz, $CDCl_3$) δ 28.6; ^{19}F NMR (376 MHz, $CDCl_3$) δ -62.78, -62.84; HRMS (ESI) Calcd for $C_{26}H_{21}F_6NO_2P$ $[M + H]^+$ 524.1209, found 524.1212.

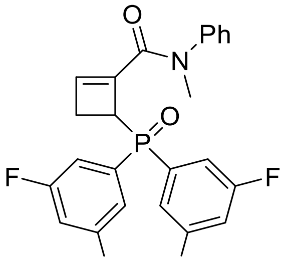

4-(Bis(3,5-difluorophenyl)phosphoryl)-*N*-methyl-*N*-phenylcyclobut-1-ene-1-

carboxamide (3ah): Prepared according to the general procedure from **1a** (0.40 mmol) and **2h** (0.20 mmol), and purified by column chromatography on silica gel with petroleum ether/ethyl acetate/ethanol (10/1/1) to provide the title compound **3ah** (46.5mg, 51% yield) as a white solid; R_f 0.3 (petroleum ether/ethyl acetate/ethanol = 10/2/1); m.p. 161.2–163.1 °C; ^{1}H NMR (400 MHz, $CDCl_3$) δ 7.41 (d, J = 7.4 Hz, 3H), 7.17–6.95 (m, 8H), 5.42 (s, 1H), 3.41 (s, 1H), 3.27 (s, 3H), 2.68 (d, J = 14.3 Hz, 1H), 2.40–2.27 (m, 1H); ^{13}C NMR (100 MHz, $CDCl_3$) δ 164.4 (dd, J_{C-F} = 253.3 Hz, J_{C-P} = 11.1 Hz), 164.2 (dd, J_{C-F} = 253.2 Hz, J_{C-P} = 11.1 Hz), 161.8 (dd, J_{C-F} = 253.3 Hz, J_{C-P} = 11.0 Hz), 161.6 (dd, J_{C-F} = 253.2 Hz, J_{C-P} = 11.0 Hz), 160.8, 144.4 (d, J_{C-P} = 15.5 Hz), 142.8, 136.6 (d, J_{C-P} = 8.8 Hz), 135.0 (d, J_{C-P} = 97.0 Hz, J_{C-F} = 6.8 Hz), 134.3 (d, J_{C-P} = 96.7 Hz, J_{C-F} = 6.7 Hz), 129.7, 128.6, 127.8, 114.1 (d, J_{C-P} = 17.7 Hz, J_{C-F} = 9.2 Hz), 114.0 (d, J_{C-P} = 17.7 Hz, J_{C-F} = 9.2 Hz), 113.8 (d, J_{C-P} = 16.9 Hz, J_{C-F} = 8.9 Hz), 113.7 (d, J_{C-P} = 17.9 Hz, J_{C-F} = 9.9 Hz), 108.3 (dt, J_{C-F} = 24.7 Hz, J_{C-P} = 5.4 Hz), 108.1 (dt, J_{C-F} = 25.1 Hz, J_{C-P} = 5.6 Hz), 38.3 (d, J_{C-P} = 74.5 Hz), 37.7, 31.7 (d, J_{C-P} = 5.1 Hz); ^{19}F NMR (376 MHz, $CDCl_3$) δ -106.00, -106.03, -106.05; ^{31}P NMR (162 MHz, $CDCl_3$) δ 27.7; HRMS (ESI) Calcd for $C_{24}H_{19}F_4NO_2P$ $[M + H]^+$ 460.1084, found 460.1084.

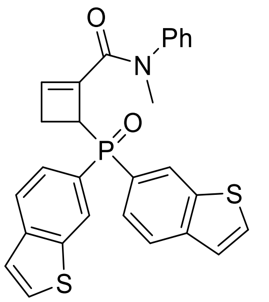

4-(Bis(benzo[*b*]thiophen-6-yl)phosphoryl)-*N*-methyl-*N*-phenylcyclobut-1-ene-1-carboxamide (3ai): Prepared according to the general procedure from **1a** (0.40 mmol) and **2i** (0.20 mmol), and purified by column chromatography on silica gel with petroleum ether/ethyl acetate/ethanol (20/1/1) to provide the title compound **3ai** (74.9 mg, 75% yield) as a white solid; R_f 0.3 (petroleum ether/ethyl acetate/ethanol = 10/2/1); m.p. 94.8–96.8 °C; ^{1}H NMR (400 MHz, $CDCl_3$) δ 8.42 (dd, J = 12.6, 5.5 Hz, 2H), 7.98 (t, J = 7.1 Hz, 2H), 7.78 (q, J = 8.6 Hz, 2H), 7.51 (d, J = 5.4 Hz, 2H), 7.42 (t, J = 5.3 Hz, 2H), 7.26–7.19 (m, 3H), 6.94–6.76 (m, 2H), 5.17 (s, 1H), 4.01 (s, 1H), 3.11 (s, 3H), 2.58–2.37 (m, 2H); ^{13}C NMR (100 MHz, $CDCl_3$) δ 161.9, 143.03 (d, J_{C-P} = 2.9 Hz), 143.02 (d, J_{C-P} = 13.7 Hz), 143.0 (d, J_{C-P} = 2.9 Hz), 139.3 (d, J_{C-P} = 12.0 Hz), 139.2 (d, J_{C-P} = 12.4 Hz), 138.9 (d, J_{C-P} = 8.1 Hz), 130.1 (d, J_{C-P} = 94.2 Hz), 129.3, 128.9 (d, J_{C-P} = 94.6 Hz), 127.8, 127.7 (d, J_{C-P} = 9.8 Hz), 127.5, 127.4, 127.3, 127.2 (d, J_{C-P} = 9.9 Hz), 126.2 (d, J_{C-P} = 11.2 Hz), 125.6 (d, J_{C-P} = 10.9 Hz), 124.3, 122.7 (d, J_{C-P} = 12.6 Hz), 122.4 (d, J_{C-P} = 13.0 Hz), 42.2 (d, J_{C-P} = 73.1 Hz), 37.5, 28.3 (d, J_{C-P} = 5.5 Hz); ^{31}P NMR (162 MHz,

$CDCl_3$) δ 31.3; HRMS (ESI) Calcd for $C_{28}H_{22}NO_2PS_2Na$ $[M + Na]^+$ 522.0722, found 522.0727.

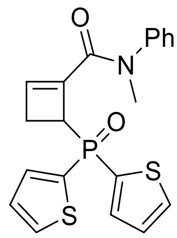

4-(Di(thiophen-2-yl)phosphoryl)-*N*-methyl-*N*-phenylcyclobut-1-ene-1-carboxamide (3aj): Prepared according to the general procedure from **1a** (0.40 mmol) and **2j** (0.20 mmol), and purified by column chromatography on silica gel with petroleum ether/ethyl acetate/ethanol (10/1/1) to provide the title compound **3aj** (65.4mg, 82% yield) as a white solid; R_f 0.3 (petroleum ether/ethyl acetate/ethanol = 10/2/1); m.p. 99.7–101.5 °C; ^{1}H NMR (400 MHz, $CDCl_3$) δ 7.88–7.57 (m, 4H), 7.42–7.28 (m, 3H), 7.25–7.17 (m, 2H), 7.04 (d, J = 7.4 Hz, 2H), 5.30 (s, 1H), 3.69 (s, 1H), 3.21 (s, 3H), 2.54–2.42 (m, 2H); ^{13}C NMR (100 MHz, $CDCl_3$) δ 161.8, 143.2, 143.1 (d, $J_{C\text{-}P}$ = 14.6 Hz), 138.7 (d, $J_{C\text{-}P}$ = 8.3 Hz), 137.5 (d, $J_{C\text{-}P}$ = 64.7 Hz), 136.7 (d, $J_{C\text{-}P}$ = 62.5 Hz), 136.0 (d, $J_{C\text{-}P}$ = 10.1 Hz), 135.5 (d, $J_{C\text{-}P}$ = 9.9 Hz), 133.2 (d, $J_{C\text{-}P}$ = 4.5 Hz), 132.9 (d, $J_{C\text{-}P}$ = 4.8 Hz), 129.4, 128.2 (d, $J_{C\text{-}P}$ = 13.6 Hz), 128.0 (d, $J_{C\text{-}P}$ = 14.2 Hz), 127.9, 127.5, 45.1 (d, $J_{C\text{-}P}$ = 82.6 Hz), 37.5, 28.0 (d, $J_{C\text{-}P}$ = 14.6 Hz); ^{31}P NMR (162 MHz, $CDCl_3$) δ 20.2; HRMS (ESI) Calcd for $C_{20}H_{18}NO_2PS_2Na$ $[M + Na]^+$ 422.0409, found 422.0416.

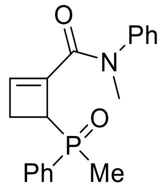

***N*-Methyl-4-(methyl(phenyl)phosphoryl)-*N*-phenylcyclobut-1-ene-1-carboxamide (3ak):** Prepared according to the general procedure from **1a** (0.40 mmol) and **2k** (0.20 mmol), and purified by column chromatography on silica gel with petroleum ether/ethyl acetate/ethanol (10/1/1) to provide the title compound **3ak** in 5:1 dr value, the major isomer (50.0 mg, 77% yield) was isolated as a white solid; R_f 0.15 (petroleum ether/ethyl acetate/ethanol = 10/2/1); m.p. 121.5–123.5 °C; ^{1}H NMR (400 MHz, $CDCl_3$) δ 7.71 (dd, J = 11.1, 7.0 Hz, 2H), 7.56–7.44 (m, 3H), 7.43–7.30 (m, 3H), 7.23 (d, J = 7.5 Hz, 2H), 4.97 (s, 1H), 3.40 (d, J = 4.8 Hz, 1H), 3.32 (s, 3H), 2.38–2.29 (m, 1H), 2.18–2.07 (m, 1H), 1.98 (d, J = 12.0 Hz, 3H); ^{13}C NMR (100 MHz, $CDCl_3$) δ 161.8, 143.5 (d, $J_{C\text{-}P}$ = 12.7 Hz), 143.3, 138.4 (d, $J_{C\text{-}P}$ = 5.3 Hz), 134.1 (d, $J_{C\text{-}P}$ = 93.8 Hz), 131.4 (d, $J_{C\text{-}P}$ = 2.5 Hz), 129.9 (d, $J_{C\text{-}P}$ = 9.2 Hz), 129.6, 128.5 (d, $J_{C\text{-}P}$ = 11.2 Hz), 128.2, 127.8, 43.2 (d, $J_{C\text{-}P}$ = 71.3 Hz), 37.8, 26.9 (d, $J_{C\text{-}P}$ = 5.6 Hz), 16.1 (d, $J_{C\text{-}P}$ = 69.8 Hz); ^{31}P NMR (162 MHz, $CDCl_3$) δ 34.1; HRMS (ESI) Calcd for $C_{19}H_{20}NO_2PNa$ $[M + Na]^+$ 348.1124, found 348.1132.

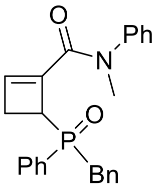

4-(Benzyl(phenyl)phosphoryl)-*N*-methyl-*N*-phenylcyclobut-1-ene-1-carboxamide (3al): Prepared according to the general procedure from **1a** (0.40 mmol) and **2l** (0.20 mmol), and purified by column chromatography on silica gel with petroleum ether/ethyl acetate/ethanol (10/1/1) to provide the title compound **3al** (76.6 mg, 96% yield) in 1.1:1 dr value, and the isomers could be separated by chromatography.

Major isomer (40.1 mg): a white solid; R_f 0.2 (petroleum ether/ethyl acetate/ethanol = 10/2/1); m.p. 157.2–159.1 °C; ^{1}H NMR (400 MHz, $CDCl_3$) δ 7.80 (dd, J = 10.5, 7.7 Hz, 2H), 7.57 (t, J = 7.4 Hz, 1H), 7.53–7.44 (m, 4H), 7.29–7.26 (d, J = 8.4 Hz, 5H), 7.21 (t, J = 7.5 Hz, 1H), 6.99–6.84 (m, 2H), 4.56–4.29 (m, 1H), 4.08 (t, J = 13.2 Hz, 1H), 3.62 (t, J = 14.7 Hz, 2H), 3.33 (s, 3H), 2.38–2.17 (m, 2H); ^{13}C NMR (100 MHz, $CDCl_3$) δ 161.8, 143.9 (d, $J_{C\text{-}P}$ = 13.0 Hz), 143.1, 139.2 (d, $J_{C\text{-}P}$ = 6.5 Hz), 131.9 (d, $J_{C\text{-}P}$ = 8.4 Hz), 131.6 (d, $J_{C\text{-}P}$ = 2.7 Hz), 131.52 (d, $J_{C\text{-}P}$ = 8.4 Hz), 130.50(d, $J_{C\text{-}P}$ = 5.1 Hz), 129.9 (d, $J_{C\text{-}P}$ = 92.7 Hz), 129.6, 128.4 (d, $J_{C\text{-}P}$ = 2.6 Hz), 128.3, 128.0 (d, $J_{C\text{-}P}$ = 11.2 Hz), 127.5, 126.6 (d, $J_{C\text{-}P}$ = 3.0 Hz), 40.6 (d, $J_{C\text{-}P}$ = 69.8 Hz), 38.0, 36.9 (d, $J_{C\text{-}P}$ = 62.0 Hz), 26.9 (d, $J_{C\text{-}P}$ = 6.2 Hz); ^{31}P NMR (162 MHz, $CDCl_3$) δ 37.9; HRMS (ESI) Calcd for $C_{25}H_{25}NO_2P$ $[M + H]^+$ 402.1617, found 402.1627.

Minor isomer (36.5 mg): a white solid; R_f 0.15 (petroleum ether/ethyl acetate/ethanol = 10/2/1); m.p. 119.5–121.1 °C; ^{1}H NMR (400 MHz, $CDCl_3$) δ 7.40 (t, J = 7.0 Hz, 3H), 7.36–7.28 (m, 7H), 7.16–7.11 (m, 5H), 4.83 (s, 1H), 3.94 (s, 1H), 3.75 (s, 1H), 3.69–3.60 (m, 1H), 3.39 (s, 3H), 2.13 (d, J = 6.1 Hz, 2H); ^{13}C NMR (100 MHz, $CDCl_3$) δ 161.9, 143.7 (d, $J_{C\text{-}P}$ = 12.9 Hz), 143.4, 138.0 (d, $J_{C\text{-}P}$ = 9.0 Hz), 132.5 (d, $J_{C\text{-}P}$ = 7.4 Hz), 131.4 (d, $J_{C\text{-}P}$ = 92.2 Hz), 131.3 (d, $J_{C\text{-}P}$ = 2.8 Hz), 130.6 (d, $J_{C\text{-}P}$ = 8.1 Hz), 130.4 (d, $J_{C\text{-}P}$ = 5.2 Hz), 130.0 (d, $J_{C\text{-}P}$ = 5.4 Hz), 129.6, 128.2, 128.1 (d, $J_{C\text{-}P}$ = 11.3 Hz), 127.9, 126.3 (d, $J_{C\text{-}P}$ = 3.5 Hz), 39.9 (d, $J_{C\text{-}P}$ = 69.5 Hz), 38.4 (d, $J_{C\text{-}P}$ = 60.0 Hz), 38.1, 27.3 (d, $J_{C\text{-}P}$ = 5.7 Hz); ^{31}P NMR (162 MHz, $CDCl_3$) δ 35.3; HRMS (ESI) Calcd for $C_{25}H_{25}NO_2P$ $[M + H]^+$ 402.1617, found 402.1624.

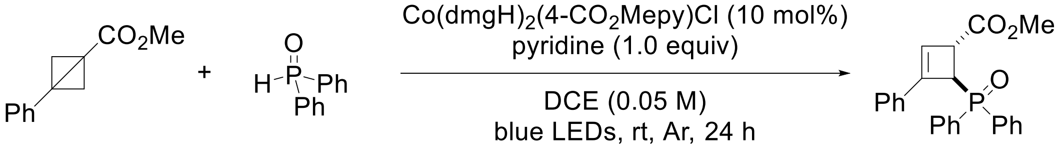

1t **2a** **3ta**, 90%

***trans*-Methyl-4-(diphenylphosphoryl)-3-phenylcyclobut-2-ene-1-carboxylate (3ta):**

Prepared according to the general procedure from **1t** (0.40 mmol) and **2a** (0.20 mmol), and purified by column chromatography on silica gel with petroleum ether/ethyl acetate/ethanol (30/1/1) to provide the title compound **3ta** (70.0 mg, 90% yield) as a white solid; R_f 0.4 (petroleum ether/ethyl acetate/ethanol = 10/2/1); ^{1}H NMR (400 MHz, $CDCl_3$) δ 7.86–7.70 (m, 4H), 7.56–7.48 (m, 2H), 7.47–7.33 (m, 4H), 7.20–7.14 (m, 1H), 7.13–6.90 (m, 4H), 6.32 (s, 1H), 4.54 (s, 1H), 3.73 (d, J = 7.1 Hz, 1H), 3.68 (s, J = 2.3 Hz, 3H); ^{13}C NMR: (100 MHz, $CDCl_3$) δ 171.8, 146.9 (d, J_{C-P} = 8.3 Hz), 132.2, 131.91 (d, J_{C-P} = 3.1 Hz), 131.90 (d, J_{C-P} = 3.0 Hz), 131.8 (d, J_{C-P} = 97.9 Hz), 131.6 (d, J_{C-P} = 98.1 Hz), 131.3 (d, J_{C-P} = 9.3 Hz), 131.1 (d, J_{C-P} = 9.1 Hz), 128.5 (d, J_{C-P} = 4.5 Hz), 128.47, 128.42 (d, J_{C-P} = 4.3 Hz), 128.0, 126.7 (d, J_{C-P} = 13.2 Hz), 125.8, 52.2, 45.4 (d, J_{C-P} = 69.3 Hz), 43.7 (d, J_{C-P} = 3.3 Hz); ^{31}P NMR (162 MHz, $CDCl_3$) δ 27.9.

5. Mechanistic Studies

(a) Detection of hydrogen gas H_2.

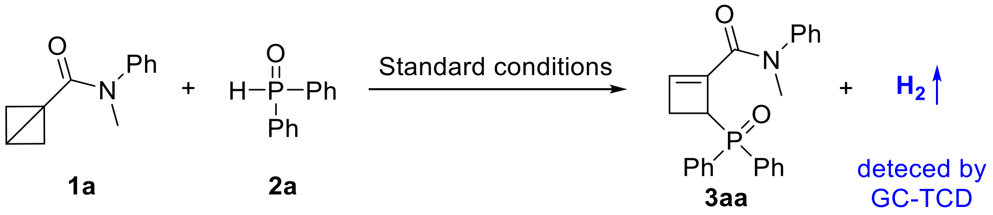

Under an argon atmosphere, an oven-dried 10 mL Schlenk tube equipped with a magnetic stir bar was charged with Co(dmgH)$_2$(4-CO$_2$Mepy)Cl (9.2 mg, 0.02 mmol, 10 mol%) and bicyclo[1.1.0]butane **1a** (0.40 mmol, 2.0 eq.). Then, the Schlenk tube was introduced in a glovebox, where it was charged with diphenylphosphine oxide **2a** (0.20 mmol, 1.0 eq.). The tube was taken out of the glovebox and connected to a vacuum line through evacuating and back-filling with Ar for 3 times. After dry DCE (4 mL) and pyridine (0.20 mmol, 1.0 eq.) were added under Ar flow, the resulting mixture was degassed via 'freeze-pump-thaw' procedure (3 times) under Ar atmosphere and was stirred under blue LED (40 W) at room temperature for 24 h. After completion of the reaction, 1000 μL of gas was extracted from the reaction system and detected by GC-TCD. According to the spectrum (Figure S4), the only peak stands for the generation of hydrogen gas.

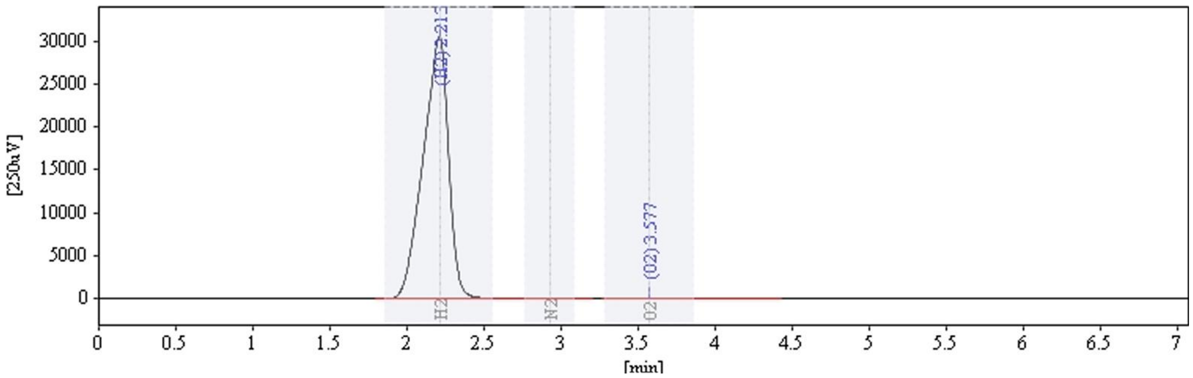

Figure S4. Hydrogen detected by GC-TCD.

(b) Separation of intermediate from model reaction with 10 mol% 2a.

Table 10. Control experiments.

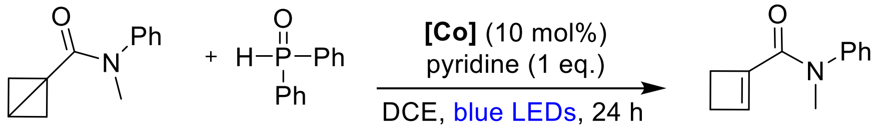

Entry	Variation to standard conditions	Yield[a]
1	none	88%
2	no pyridine	83%
3[b]	no **2a**	0%
4[c]	no **[Co]**	0%
5[d]	no visible light	0%

Reaction condition: **1a** (0.4 mmol), **2a** (0.04 mmol), [Co] 10 mol% and pyridine (0.2 mmol) in DCE (4 mL) was irradiated with 40 W blue LEDs for 24 h under argon atmosphere at rt. [a] isolation yield. [b] 93% recovery of **1a**. [c] 89% recovery of **1a**. [d] 95% recovery of **1a**.

Under an argon atmosphere, an oven-dried 10 mL Schlenk tube equipped with a magnetic stir bar was charged with Co(dmgH)$_2$(4-CO$_2$Mepy)Cl (0.04 mmol, 10 mol%) and bicyclo[1.1.0]butane **1a** (0.40 mmol, 1.0 eq.). Then, the Schlenk tube was introduced in a glovebox, where it was charged with diphenylphosphine oxide **2a** (0.04 mmol, 10 mol%). The tube was taken out of the glovebox and connected to a vacuum line through evacuating and back-filling with Ar for 3 times. After dry DCE (4 mL) and pyridine (0.40 mmol, 1.0 eq.) were added under Ar flow, the resulting mixture was degassed via ‘freeze-pump-thaw’ procedure (3 times) under Ar atmosphere and was stirred under blue LED (40 W) at room temperature for 24 h (monitored by TLC analysis). The reaction mixture was concentrated under reduced pressure and the crude residue was purified by column chromatography on silica gel (eluent: petroleum ether: ethyl acetate = 10:1) to give the intermediate **4a** (66.3 mg, 88% yield). The reaction without pyridine afforded the intermediate **4a** in 83% yield (62.2 mg), whereas no reaction occurred without **2a**, cobalt catalyst or visible light, and bicyclo[1.1.0]butane substrate **1a** was recovered.

***N*-Methyl-*N*-phenylcyclobut-1-ene-1-carboxamide** (**4a**): white solid; m.p. 62.7-64.1 °C; ^{1}H NMR (400 MHz, CDCl$_3$) δ 7.43–7.33 (m, 3H), 7.24–7.19 (m, 2H), 5.69 (s, 1H), 3.30 (s, 3H), 2.22 (s, 2H), 2.16 (s, 2H); ^{13}C NMR (100 MHz, CDCl$_3$) δ 162.5, 150.4, 143.5, 142.9, 141.1, 129.2, 127.7, 37.5, 30.7, 26.6; HRMS (ESI) Calcd for C$_{12}$H$_{14}$NO [M + H]$^+$ 188.1070, found 188.1079.

(c) Control experiment with intermediate 4a.

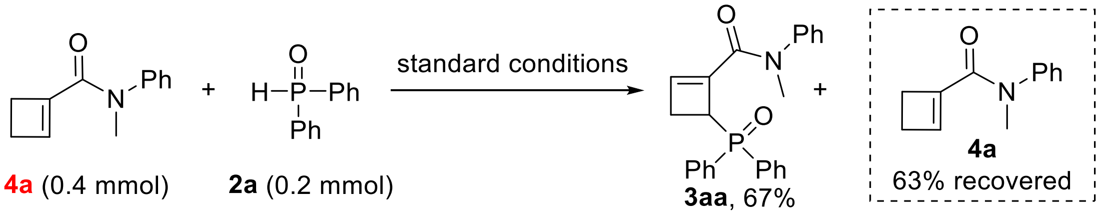

Under an argon atmosphere, an oven-dried 10 mL Schlenk tube equipped with a magnetic stir bar was charged with Co(dmgH)$_2$(4-CO$_2$Mepy)Cl (0.02 mmol, 10 mol%) and cyclobutene **4a** (0.40 mmol, 2.0 eq.). Then, the Schlenk tube was introduced in a glovebox, where it was charged with diphenylphosphine oxide **2a** (0.20 mmol, 1.0 eq.). The tube was taken out of the glovebox and connected to a vacuum line through evacuating and back-filling with Ar for 3 times. After dry DCE (4 mL) and pyridine (0.20 mmol, 1.0 eq.) were added under Ar flow, the resulting mixture was degassed via 'freeze-pump-thaw' procedure (3 times) under Ar atmosphere and was stirred under blue LED (40 W) at room temperature for 24 h (monitored by TLC analysis). The reaction mixture was concentrated under reduced pressure and the crude residue was purified by column chromatography on silica gel (eluent: petroleum ether: ethyl acetate: ethanol = 30:1:1-10:1:1) to give the **3aa** (52.0 mg, 67% yield) and to recover intermediate **4a** (47.5 mg, 63% yield).

(d) Radical trapping experiment.

Following the standard procedure of the model reaction, when 2.0 equiv. radical inhibitor 2,2,6,6-tetramethylpiperidine-1-oxy (TEMPO) was added to the reaction mixture, the formation of the desired product **3aa** was completely inhibited, and the radical adduct **5** was obtained in 5.2% yield.

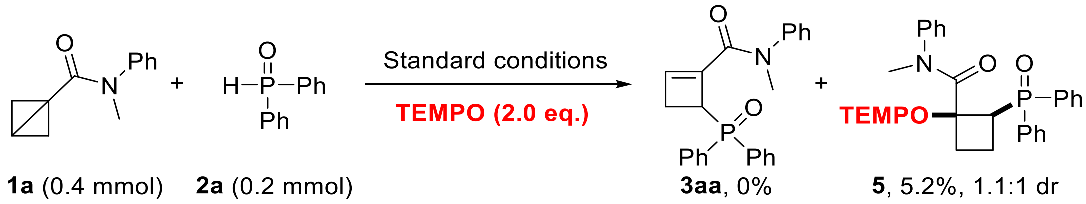

2-(Diphenylphosphoryl)-*N*-methyl-*N*-phenyl-1-(((2,2,6,6-tetramethylpiperidin-1-yl)oxy)methyl)cyclobutane-1-carboxamide (5): white solid; m.p. 183.5–185.3 °C; ^{1}H NMR (400 MHz, CDCl$_3$) δ 7.67 (dd, *J* = 22.8, 13.9 Hz, 8H), 7.50 (dd, *J* = 20.2, 7.0 Hz, 12H), 7.31 (q, *J* = 5.8, 3.8 Hz, 5H), 7.25–7.14 (m, 3H), 7.05 (d, *J* = 7.8 Hz, 2H), 3.33 (s, 7H), 3.08 (s, 3H), 2.90 (d, *J* = 42.7 Hz, 4H), 1.53 (d, *J* = 27.5 Hz, 11H), 1.44–1.28 (m, 5H), 1.27–1.10 (m, 7H), 1.04 (d, *J* = 19.2 Hz, 10H), 0.91–0.63 (m, 4H); ^{31}P NMR (162 MHz, CDCl$_3$) δ 35.4, 34.7;

HRMS (ESI) Calcd for $C_{33}H_{41}N_2O_3Na$ $[M + Na]^+$, 567.2747 found 567.2755.

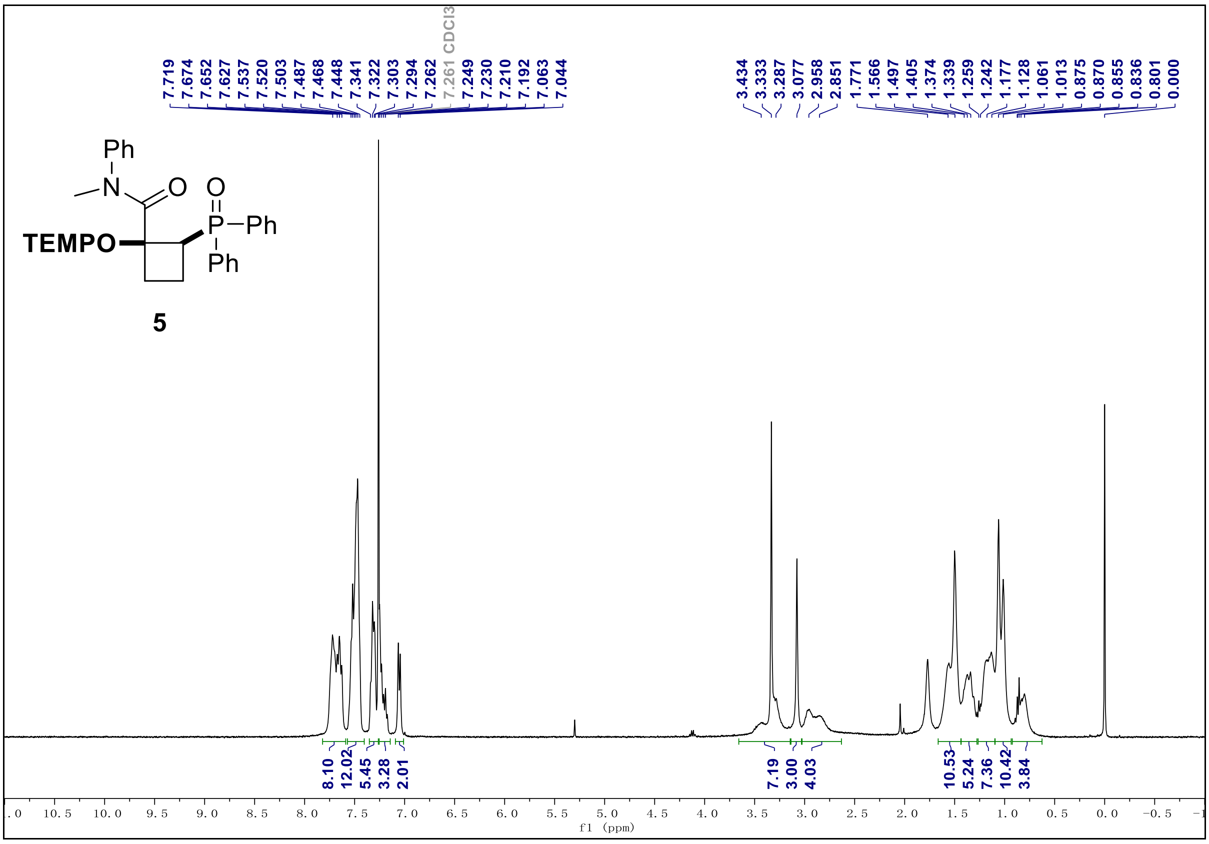

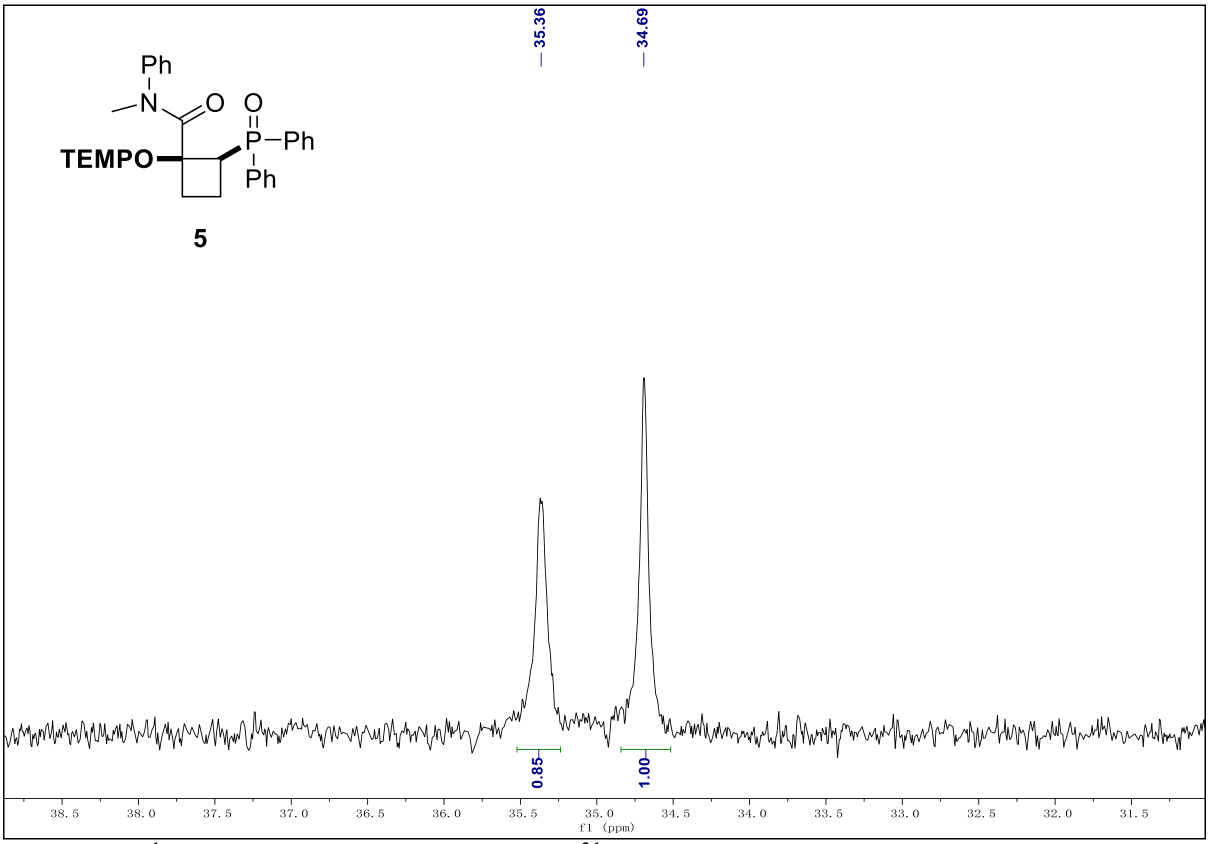

Figure S5. ^{1}H NMR (400 MHz, $CDCl_3$) and ^{31}P NMR (162 MHz, $CDCl_3$) spectrum of **5**.

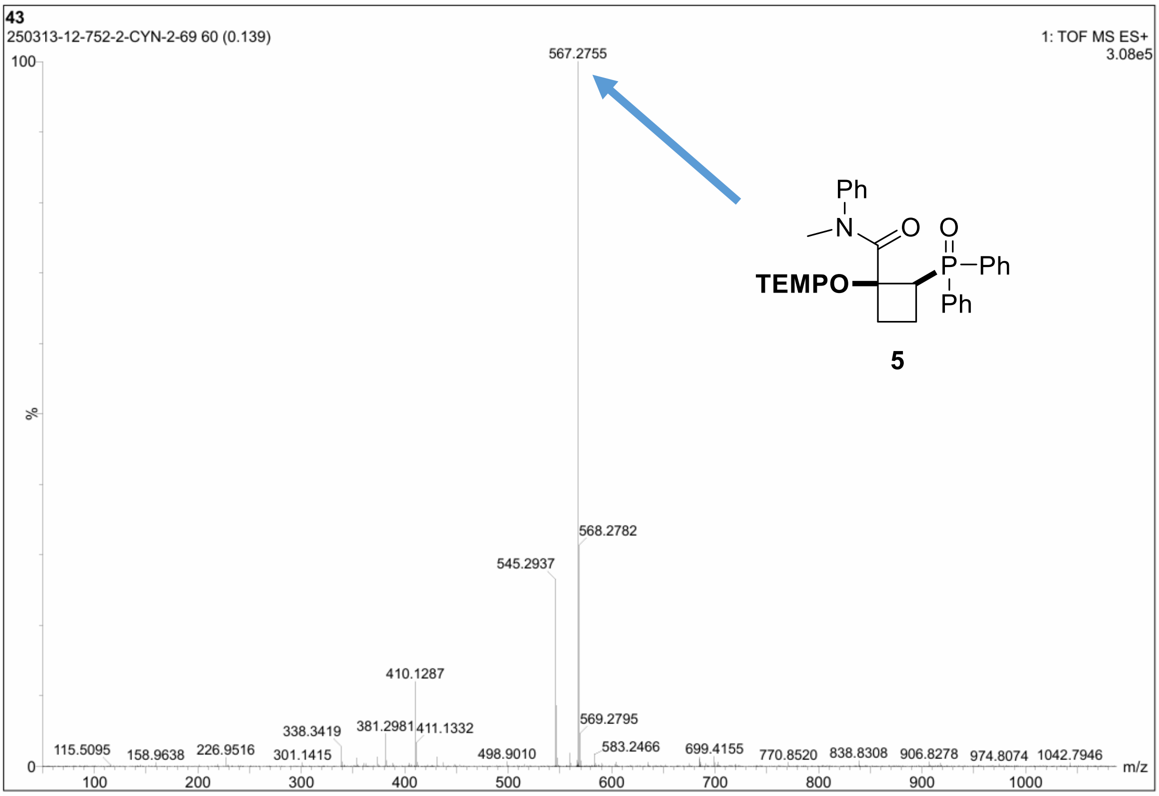

Figure S6. High-resolution mass spectrum of **5**.

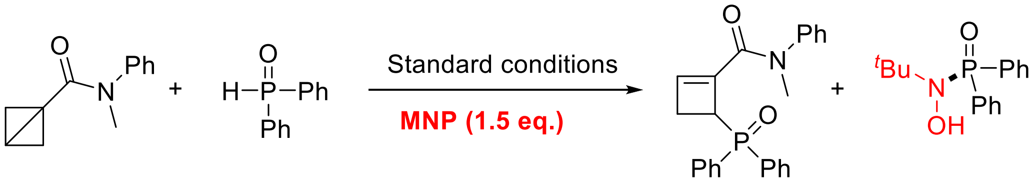

Following the standard procedure of the model reaction, when 1.5 equiv. radical inhibitor 2-methyl-2-nitrosopropane dimer (MNP) was added to the reaction mixture, the formation of the desired product **3aa** was completely inhibited, and the radical adduct **6** was obtained in 72% yields.[10] ^{1}H NMR (400 MHz, $CDCl_3$) δ 7.96–7.79 (m, 4H), 7.55–7.41 (m, 6H), 5.74 (s, 1H), 1.11 (s, 9H); ^{31}P NMR (162 MHz, $CDCl_3$) δ 34.1.

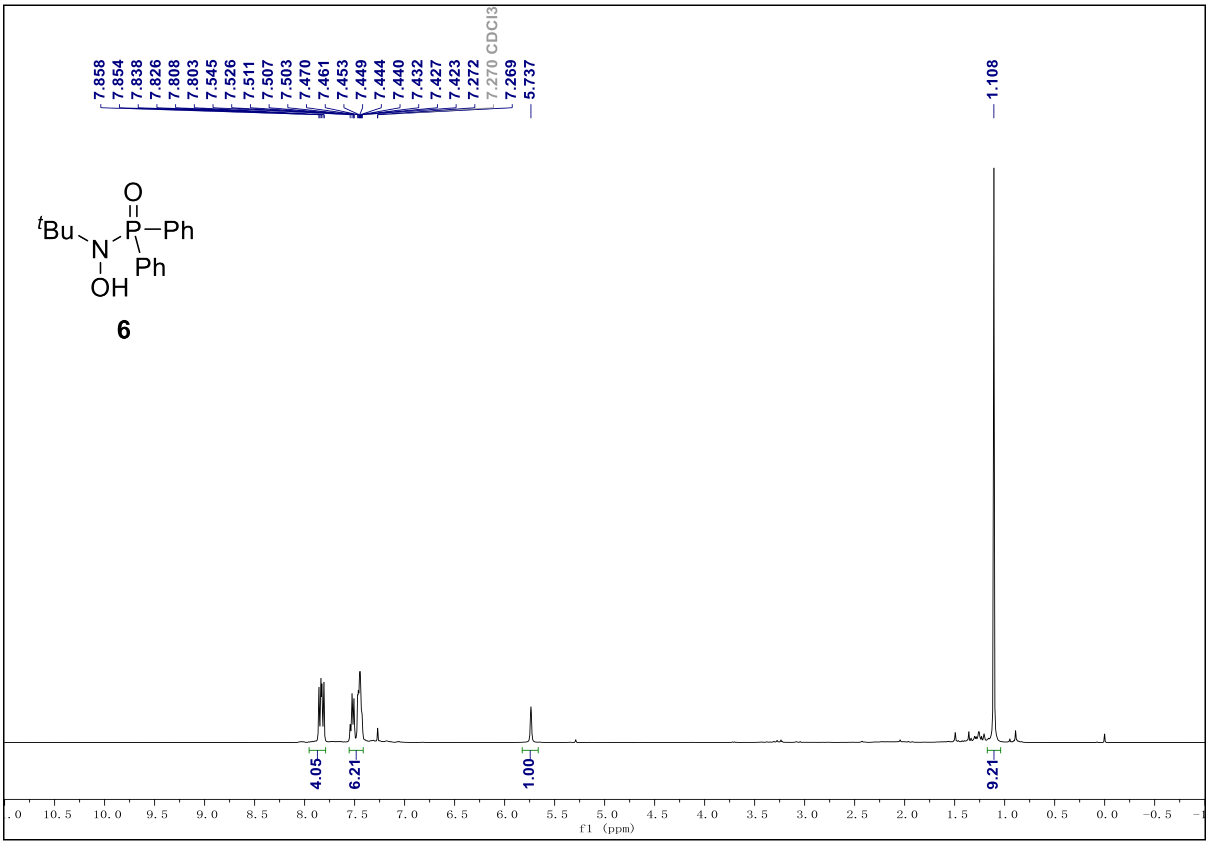

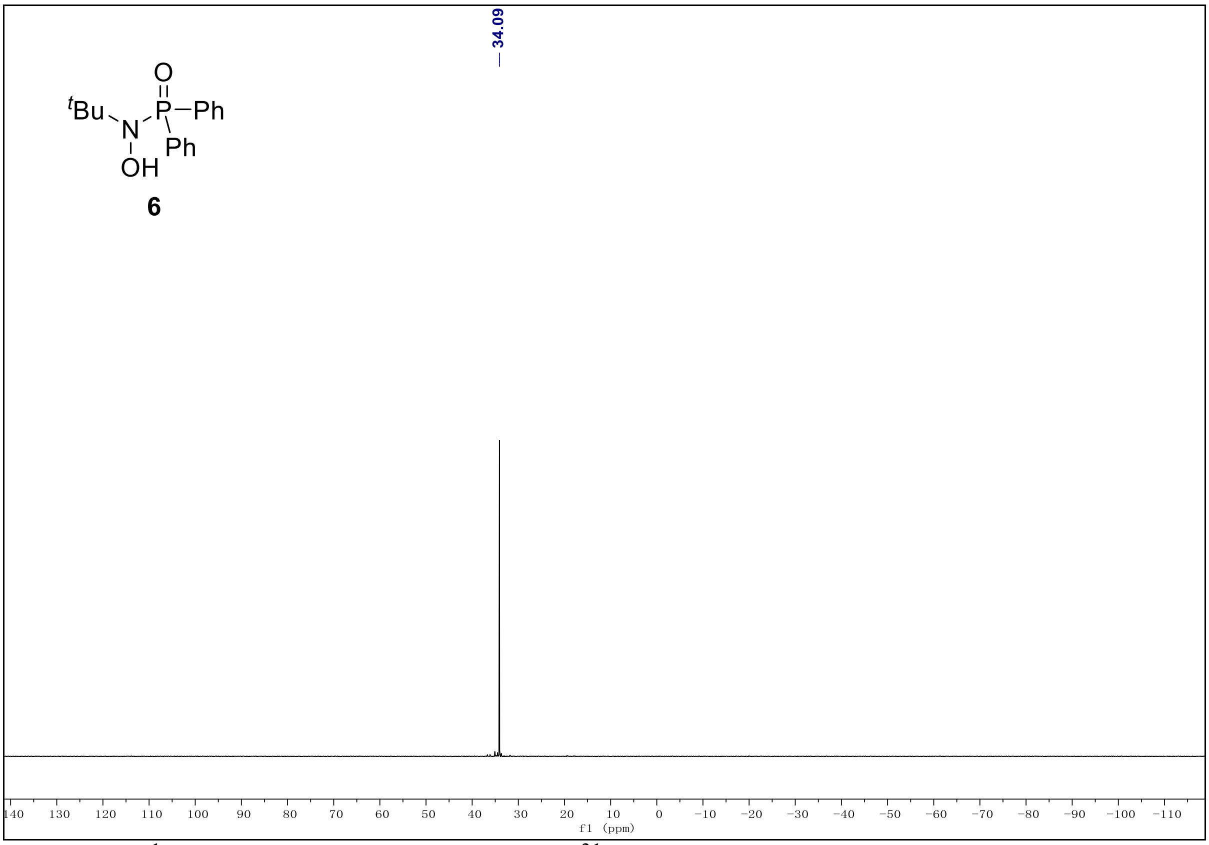

Figure S7. ^{1}H NMR (400 MHz, $CDCl_3$) and ^{31}P NMR (162 MHz, $CDCl_3$) spectrum of **6**.

6. Product Transformations

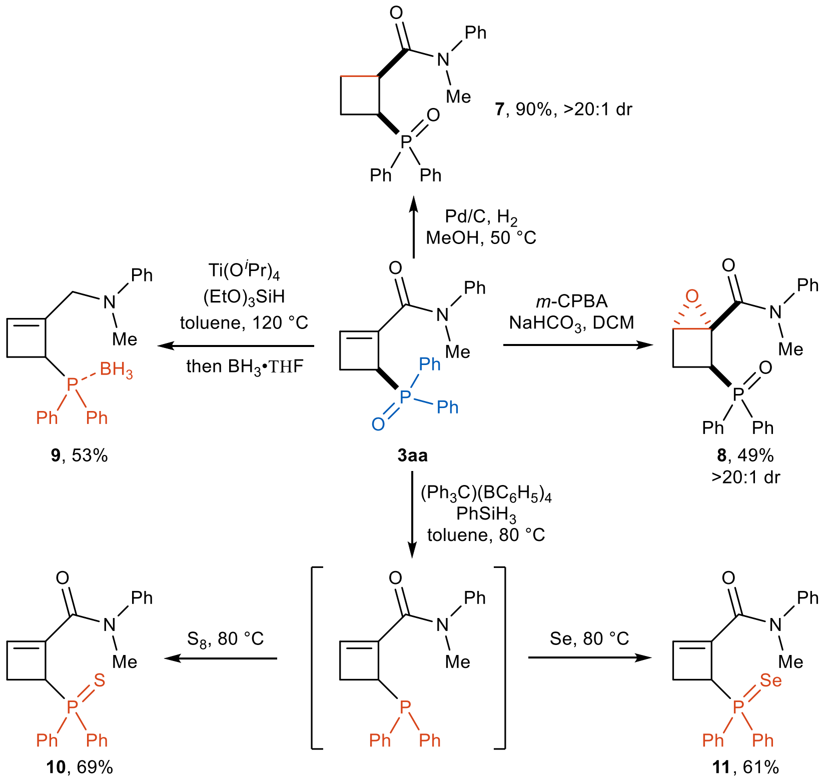

Figure S8. Synthetic applications.

Synthesis of compound 7:

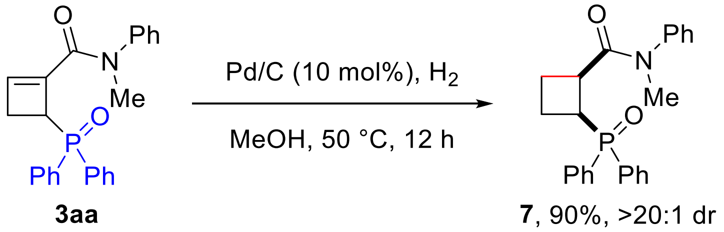

A 10 mL over-dried Schlenk tube equipped with a stir bar was charged with product **3aa** (77.4 mg, 0.20 mmol) under Ar atmosphere. After Pd/C (5% moisture content) (0.02 mmol, 10 mol%) and MeOH (4.0 mL) were added, the mixture was bubbled with H_2 balloon for three times. The resulting mixture was stirred with a H_2 balloon at 50 °C in oil bath for 12 h (monitored by TLC). After the reaction was cooled down to room temperature, the reaction solution was filtered by silica gel and the filtrate was concentrated under reduced pressure. The residue was purified by recrystallization to afford the compound **7** (69.8 mg, 90% yield, >20:1 dr) as a white solid. The relative configuration of **7** was established by the 2D NOESY data; m.p. 172.5–174.3 °C; ^{1}H NMR (400 MHz, $CDCl_3$) δ 7.80–7.68 (m, 4H), 7.51–7.40 (m, 6H), 7.37–7.27 (m, 3H), 7.09 (d, J = 7.4 Hz, 2H), 3.54–3.42 (m, 1H), 3.30–3.20 (m, 1H), 3.11 (s, 3H), 2.50–2.38 (m, 1H), 2.35–

2.24 (m, 1H), 2.17–2.06 (m, 1H), 1.75–1.69 (m, 1H); ^{13}C NMR (100 MHz, $CDCl_3$) δ 170.8 (d, J_{C-P} = 5.3 Hz), 144.0, 134.6 (d, J_{C-P} = 97.8 Hz), 133.4 (d, J_{C-P} = 97.5 Hz), 131.6 (d, J_{C-P} = 8.8 Hz), 131.2 (d, J_{C-P} = 3.5 Hz), 131.1 (d, J_{C-P} = 3.4 Hz), 130.8 (d, J_{C-P} = 8.9 Hz), 129.4, 128.3 (d, J_{C-P} = 11.6 Hz), 128.1 (d, J_{C-P} = 11.4 Hz), 127.4, 127.3, 39.4 (d, J_{C-P} = 6.3 Hz), 37.6, 37.0 (d, J_{C-P} = 70.4 Hz), 24.3 (d, J_{C-P} = 6.1 Hz), 21.0 (d, J_{C-P} = 4.2 Hz); ^{31}P NMR (162 MHz, $CDCl_3$) δ 30.5; HRMS (ESI) Calcd for $C_{24}H_{24}NO_2PNa$ $[M + Na]^+$ 412.1437, found 412.1445.

Synthesis of compound 8:

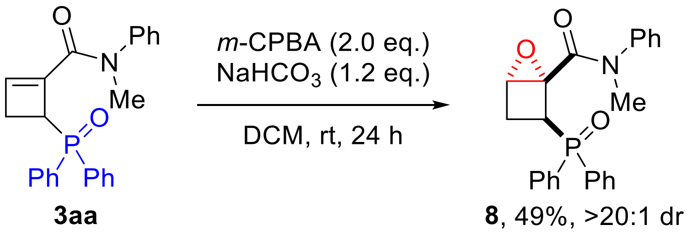

A 50 mL over-dried Schlenk tube equipped with a stir bar was charged with product **3aa** (77.4 mg, 0.20 mmol) under Ar atmosphere. After *m*-CPBA (62.0 mg, 0.4 mmol), $NaHCO_3$ (20.2 mg, 0.24 mmol) and dry DCM (10 mL) were added, the resulting mixture was stirred at room temperature for 24 h (monitored by TLC). Then the reaction solution was filtered by silica gel and the filtrate was concentrated under reduced pressure. The residue was purified by flash column chromatography on silica gel with petroleum ether/ethyl acetate/ethanol (20:1:1) to afford the compound **8** (39.6 mg, 49% yield) as a white solid; R_f 0.5 (petroleum ether/ethyl acetate/ethanol = 10:2:1); m.p. 155.5–157.3 °C; ^1H NMR (400 MHz, $CDCl_3$) δ 7.89 (dd, J = 12.0, 7.1 Hz, 2H), 7.70 (dd, J = 11.3, 7.4 Hz, 2H), 7.55–7.43 (m, 6H), 7.37–7.30 (m, 3H), 7.23–7.15 (m, 2H), 3.18–3.10 (s, 4H), 3.08–2.96 (s, 1H), 2.37–2.24 (m, 1H), 1.81–1.71 (m, 1H); ^{13}C NMR (100 MHz, $CDCl_3$) δ 163.7, 141.8, 132.5, 131.9 (d, J_{C-P} = 6.2 Hz), 131.8 (d, J_{C-P} = 6.8 Hz), 131.2 (d, J_{C-P} = 9.6 Hz), 130.7 (d, J_{C-P} = 9.3 Hz), 130.0 (d, J_{C-P} = 96.9 Hz), 129.5 (d, J_{C-P} = 96.8 Hz), 129.0, 128.7 (d, J_{C-P} = 11.8 Hz), 128.4 (d, J_{C-P} = 12.1 Hz), 128.0, 63.2, 61.8, 41.7 (d, J_{C-P} = 72.8 Hz), 38.0, 31.4, 30.2, 29.1; ^{31}P NMR (162 MHz, $CDCl_3$) δ 30.2; HRMS (ESI) Calcd for $C_{24}H_{22}NO_3PNa$ $[M + Na]^+$ 426.1230, found 426.1228.

Synthesis of compound 9:

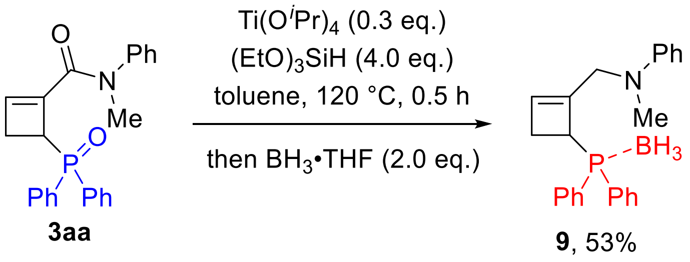

A 10 mL over-dried Schlenk tube equipped with a stir bar was charged with product **3aa** (77.40 mg, 0.20 mmol) under Ar atmosphere. After toluene (2.0 mL), $(EtO)_3SiH$ (0.80 mmol, 4.0 eq.) and Ti(O^{i}Pr)$_4$ (0.06 mmol, 0.3 eq.) were added sequentially via syringe, the resulting mixture was stirred at 120 °C in oil bath for 0.5 h. Then the reaction mixture was cooled down to room temperature, and dry BH_3·THF (1 M in THF, 2.0 eq.) was added under Ar atmosphere. The reaction mixture was stirred at room temperature for 3 h (monitored by TLC), then CH_2Cl_2 (2.0 mL) was added and concentrated under reduced pressure. The residue was purified by flash column chromatography on silica gel with petroleum ether/ethyl acetate/ethanol (100:1:1) to afford the compound **9** (39.3 mg, 53% yield) as a white solid; R_f 0.7 (petroleum ether/ethyl acetate/ethanol = 20:1:1); m.p. 169.0-171.2 ºC; ^{1}H NMR (400 MHz, $CDCl_3$) δ 7.80–7.75 (m, 2H), 7.67–7.60 (m, 2H), 7.49–7.36 (m, 6H), 7.19–7.12 (m, 2H), 6.71–6.65 (m, 1H), 6.62–6.54 (m, 2H), 5.84 (s, 1H), 3.94–3.85 (m, 1H), 3.71–3.53 (m, 2H), 2.81 (s, 3H), 2.74–2.57 (m, 2H); ^{13}C NMR (100 MHz, $CDCl_3$) δ 149.4, 143.6 (d, $J_{C\text{-}P}$ = 4.0 Hz), 132.4 (d, $J_{C\text{-}P}$ = 8.8 Hz), 131.9 (d, $J_{C\text{-}P}$ = 8.7 Hz), 131.8 (d, $J_{C\text{-}P}$ = 13.7 Hz), 131.4 (d, $J_{C\text{-}P}$ = 2.4 Hz), 131.1 (d, $J_{C\text{-}P}$ = 2.5 Hz), 129.5 (d, $J_{C\text{-}P}$ = 53.5 Hz), 129.4 (d, $J_{C\text{-}P}$ = 53.0 Hz), 128.9, 128.8 (d, $J_{C\text{-}P}$ = 10.0 Hz), 128.7 (d, $J_{C\text{-}P}$ = 9.8 Hz), 116.5, 112.4, 52.7, 38.5, 38.1 (d, $J_{C\text{-}P}$ = 34.0 Hz), 29.8 (d, $J_{C\text{-}P}$ = 2.7 Hz); ^{31}P NMR (162 MHz, $CDCl_3$) δ 16.6, 16.1; HRMS (ESI) Calcd for $C_{24}H_{28}BNP$ $[M + H]^+$ 372.2047, found 372.2055.

Synthesis of Compound 10.

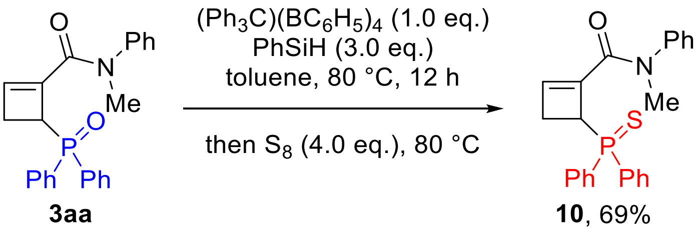

A 10 mL over-dried Schlenk tube equipped with a stir bar was charged with product **3aa** (38.7 mg, 0.10 mmol) under Ar atmosphere. After $(Ph_3C)(BC_6H_5)_4$ (0.10 mmol, 1.0 eq.), toluene (1.5

mL) and $PhSiH_3$ (0.30 mmol, 3.0 eq.) were added, the resulting mixture was stirred at 80 °C in oil bath for 12 h. The reaction solution was cooled down to room temperature, and S_8 (0.40 mmol, 4.0 eq.) was added under Ar atmosphere. The reaction mixture was stirred at 80 °C in oil bath for additional 2 h (monitored by TLC), then saturated aq. NH_4Cl (2.0 mL) was added and extracted with CH_2Cl_2 (6.0 mL), dried over Na_2SO_4, and concentrated under reduced pressure. The residue was purified by flash column chromatography on silica gel with petroleum ether/ethyl acetate/ethanol (100:1:1) to afford the compound **10** (27.9 mg, 69% yield) as a light yellow solid; R_f 0.7 (petroleum ether/ethyl acetate/ethanol = 20:1:1); m.p. 175.5–176.3 °C; ^{1}H NMR (400 MHz, $CDCl_3$) δ 8.09–7.94 (m, 2H), 7.89–7.74 (m, 2H), 7.52–7.42 (m, 6H), 7.38–7.29 (m, 3H), 7.20 (d, J = 7.5 Hz, 2H), 5.14 (s, 1H), 4.26 (s, 1H), 3.19 (s, 3H), 2.31 (s, 2H); ^{13}C NMR (100 MHz, $CDCl_3$) δ 161.6, 143.20, 142.7 (d, $J_{C\text{-}P}$ = 11.6 Hz), 139.0 (d, $J_{C\text{-}P}$ = 7.8 Hz), 133.2 (d, $J_{C\text{-}P}$ = 81.0 Hz), 132.4 (d, $J_{C\text{-}P}$ = 81.7 Hz), 132.0 (d, $J_{C\text{-}P}$ = 10.3 Hz), 131.4 (d, $J_{C\text{-}P}$ = 2.9 Hz), 131.2 (d, $J_{C\text{-}P}$ = 2.9 Hz), 131.0 (d, $J_{C\text{-}P}$ = 9.8 Hz), 129.3, 128.4 (d, $J_{C\text{-}P}$ = 11.7 Hz), 128.1 (d, $J_{C\text{-}P}$ = 12.2 Hz), 127.8, 127.7, 42.3 (d, $J_{C\text{-}P}$ = 54.8 Hz), 37.6, 28.9 (d, $J_{C\text{-}P}$ = 3.9 Hz); ^{31}P NMR (162 MHz, $CDCl_3$) δ 44.3; HRMS (ESI) Calcd for $C_{24}H_{22}NOPSNa$ $[M + Na]^+$ 426.1052, found 426.1058.

Synthesis of Compound 11.

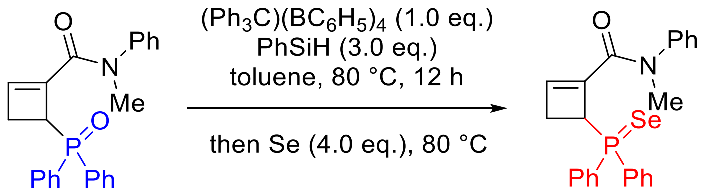

3aa **11**, 61%

A 10 mL over-dried Schlenk tube equipped with a stir bar was charged with product **3aa** (38.7 mg, 0.10 mmol) under Ar atmosphere. After $(Ph_3C)(BC_6H_5)_4$ (0.10 mmol, 1.0 eq.), toluene (1.5 mL) and $PhSiH_3$ (0.30 mmol, 3.0 eq.) were added, the resulting mixture was stirred at 80 °C in oil bath for 12 h. The reaction solution was cooled down to room temperature, and S_8 (0.40 mmol, 4.0 eq.) was added under Ar atmosphere. The reaction mixture was stirred at 80 °C in oil bath for additional 2 h (monitored by TLC), then saturated aq. NH_4Cl (2.0 mL) was added and extracted with CH_2Cl_2 (6.0 mL), dried over Na_2SO_4, and concentrated under reduced pressure. The residue was purified by flash column chromatography on silica gel with petroleum ether/ethyl acetate/ethanol (100:1:1) to afford the compound **11** (27.5 mg, 61% yield) as a white solid; R_f 0.6 (petroleum ether/ethyl acetate/ethanol = 20:1:1); m.p. 187.2–189.3 °C; ^{1}H NMR (400 MHz, $CDCl_3$) δ 8.04 (dd, J = 13.6, 7.0 Hz, 2H), 7.83 (dd, J = 12.9, 7.1 Hz, 2H),

7.51–7.40 (m, 6H), 7.37–7.28 (m, 3H), 7.26–7.20 (m, 2H), 5.12 (s, 1H), 4.34 (s, 1H), 3.19 (s, 3H), 2.27 (s, 2H); ^{13}C NMR (100 MHz, $CDCl_3$) δ 161.4, 143.2, 142.8 (d, J_{C-P} = 13.9 Hz), 139.5 (d, J_{C-P} = 7.4 Hz), 132.6 (d, J_{C-P} = 10.6 Hz), 132.1 (d, J_{C-P} = 90.5 Hz), 131.5 (d, J_{C-P} = 10.0 Hz), 131.41 (d, J_{C-P} = 90.3 Hz), 131.40 (d, J_{C-P} = 3.9 Hz), 131.3 (d, J_{C-P} = 3.0 Hz), 129.3, 128.5 (d, J_{C-P} = 11.9 Hz), 128.2 (d, J_{C-P} = 12.3 Hz), 127.9, 127.8, 41.6 (d, J_{C-P} = 47.3 Hz), 37.7, 29.7 (d, J_{C-P} = 3.5 Hz); ^{31}P NMR (162 MHz, $CDCl_3$) δ 38.9; HRMS (ESI) Calcd for $C_{24}H_{22}NOPSe$ $[M + H]^+$ 452.0677, found 452.0678.

7. X-Ray Diffraction Analysis

Recrystallization from PE/EA/DCM afforded single crystals suitable for X-ray diffraction analysis, which unambiguously confirmed the molecular structure of **3aa** (Figure S9). A suitable crystal was selected and measured on a XtaLAB Synergy R, DW system, HyPix diffractometer. The crystal was kept at 100.00(10) K during data collection. Using Olex2, the structure was solved with the SHELXS structure solution program using Direct Methods and refined with the SHELXL refinement package using Least Squares minimisation. These data can be obtained free of charge from the Cambridge Crystallographic Data Centre.

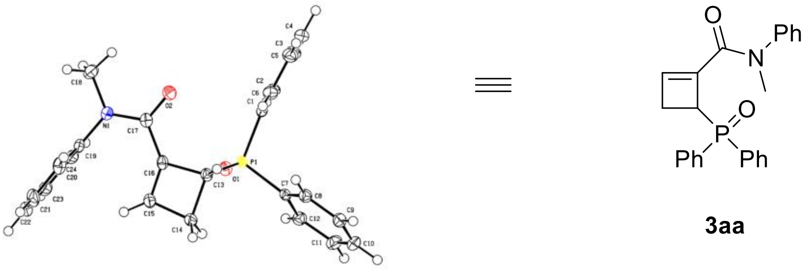

3aa

Figure S9. X-ray structure of **3aa** (CCDC: 2388351) (The thermal ellipsoid was drawn at the 50% probability level).

Table S11 Crystal data and structure refinement for **3aa**.

Identification code	**3aa**
Empirical formula	$C_{24}H_{22}NO_2P$
Formula weight	387.39
Temperature/K	100.00(10)
Crystal system	monoclinic
Space group	$P2_1/n$
a/Å	11.53630(10)
b/Å	12.06750(10)
c/Å	14.55360(10)
α/°	90
β/°	102.1150(10)
γ/°	90
Volume/Å^3	1980.94(3)
Z	4
ρ_{calc}g/cm^3	1.299

μ/mm^{-1}	1.380
F(000)	816.0
Crystal size/mm^3	0.15 × 0.13 × 0.12
Radiation	Cu Kα (λ = 1.54184)
2Θ range for data collection/°	8.924 to 146.474
Index ranges	-14 ≤ h ≤ 10, -14 ≤ k ≤ 5, -17 ≤ l ≤ 17
Reflections collected	13272
Independent reflections	3870 [R_{int} = 0.0264, R_{sigma} = 0.0236]
Data/restraints/parameters	3870/0/255
Goodness-of-fit on F^2	1.036
Final R indexes [I>=2σ (I)]	R_1 = 0.0330, wR_2 = 0.0857
Final R indexes [all data]	R_1 = 0.0351, wR_2 = 0.0874
Largest diff. peak/hole / e Å^{-3}	0.41/-0.34

8. References

(1) Sahoo, M. K.; Balaraman, E. *Green Chem.* **2019**, *21*, 2119-2128.

(2) Bai, D.-C.; Guo, X.-L.; Wang, X.-H.; Xu, W.-J.; Cheng, R.-S.; Wei, D.-H.; Lan, Y.; Chang, J.-B. *Nat. Commun.* **2024***,* 15*,* 2833.

(3) Wang, H.-M.; Shao, H.-L; Das, A.; Dutta, S.; Chan, H. T.; Daniliuc, C.; Houk, K. N.; Glorius, F. *Science* **2023**, *381*, 75-81.

(4) Guo, R.-Y.; Chang, Y.-C.; Herter, L.; Salome, C.; Braley, S. E.; Fessard, T. C.; Brown, M. K. *J. Am. Chem. Soc.* **2022**, *144*, 7988–7994.

(5) Yang, L.-L.; Wang, H.-Y.; Lang, M.; Wang, J.; Peng, S.-Y. *Org. Lett.* **2024**, 26, 4104–4110.

(6) Zhu, S.-J.; Tian, X.; Li, S.-W. *Org. Lett.* **2024**, 26, 6309–6313.

(7) Ociepa, M.; Wierzba, A. J.; Turkowska, J.; Gryko, D. *J. Am. Chem. Soc*. **2020**, *142*, 5355−5361.

(8) Jiao, R.-C.; Li, M.; Chen, X.; Zhang, Z.; Gong, X.-P.; Yue, H.; Liu, X.-Y.; Liang, Y.-M. *Org. Lett.* **2024**, *26*, 1387–1392.

(9) Tang, J.; Li, Z.-Y.; Meng, Q.; Liu, L.; Huang, T.-Z.; Li, C.-Y.; Li, Q.; Chen, T.-Q. *J. Org. Chem.* **2024**, *89*, 7848–7858.

(10) Liu, W.-Q.; Lei, T.; Zhou, S.; Yang, X.-L.; Li, J.; Chen, B.; Sivaguru, J.; Tung, C.-H.; Wu, L.-Z. *J. Am. Chem. Soc*. **2019**, *141*, 13941–13947.

9. NMR Spectra

^{1}H NMR (400 MHz, CDCl$_3$), ^{13}C NMR (100 MHz, CDCl$_3$) and ^{31}P NMR (162 MHz, CDCl$_3$) spectrum of 3aa.

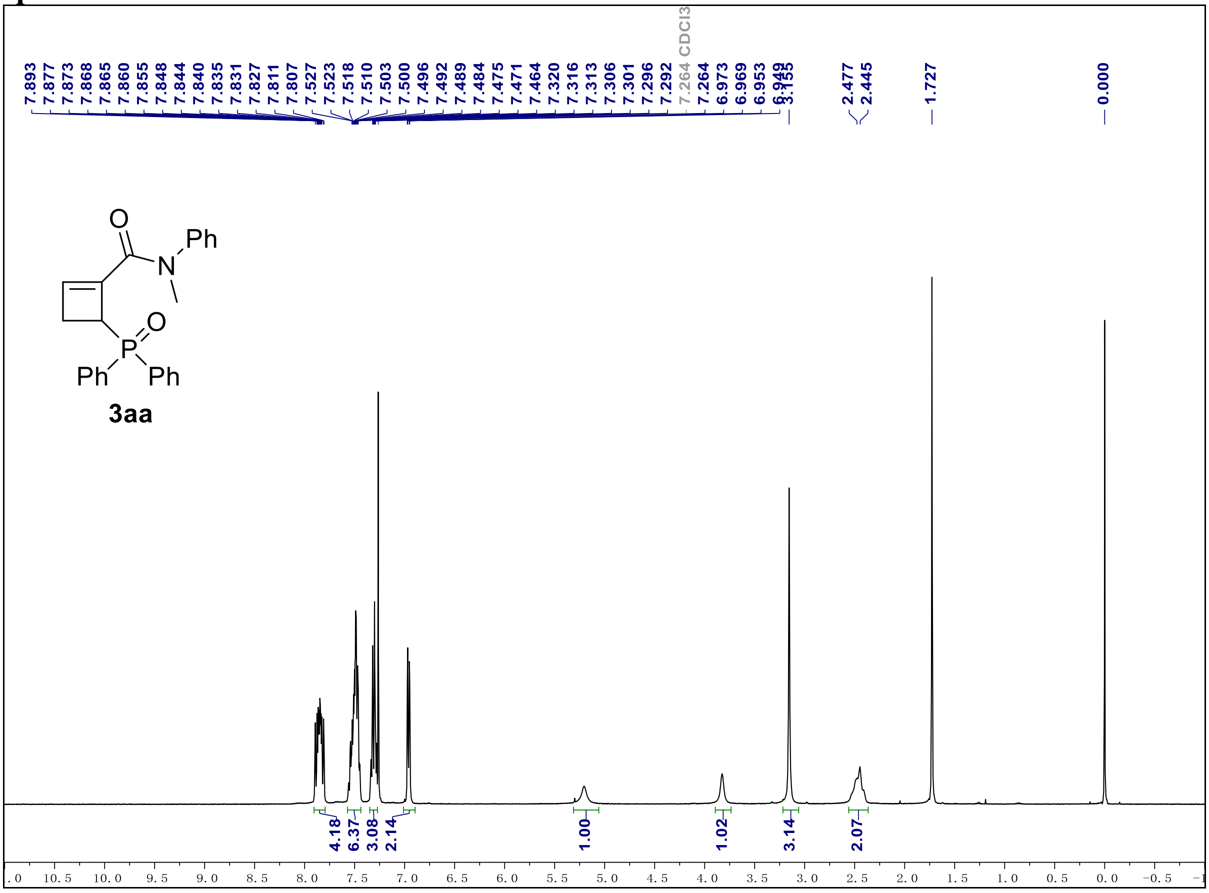

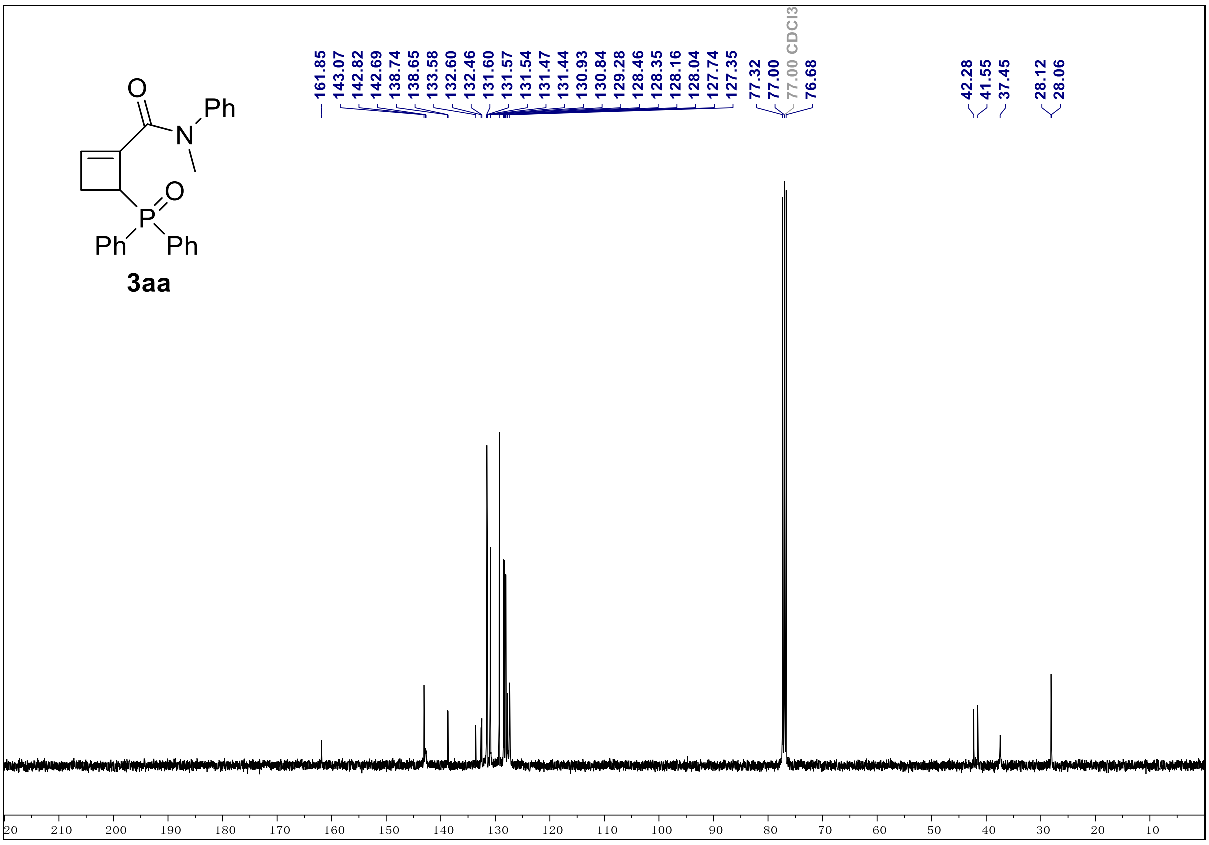

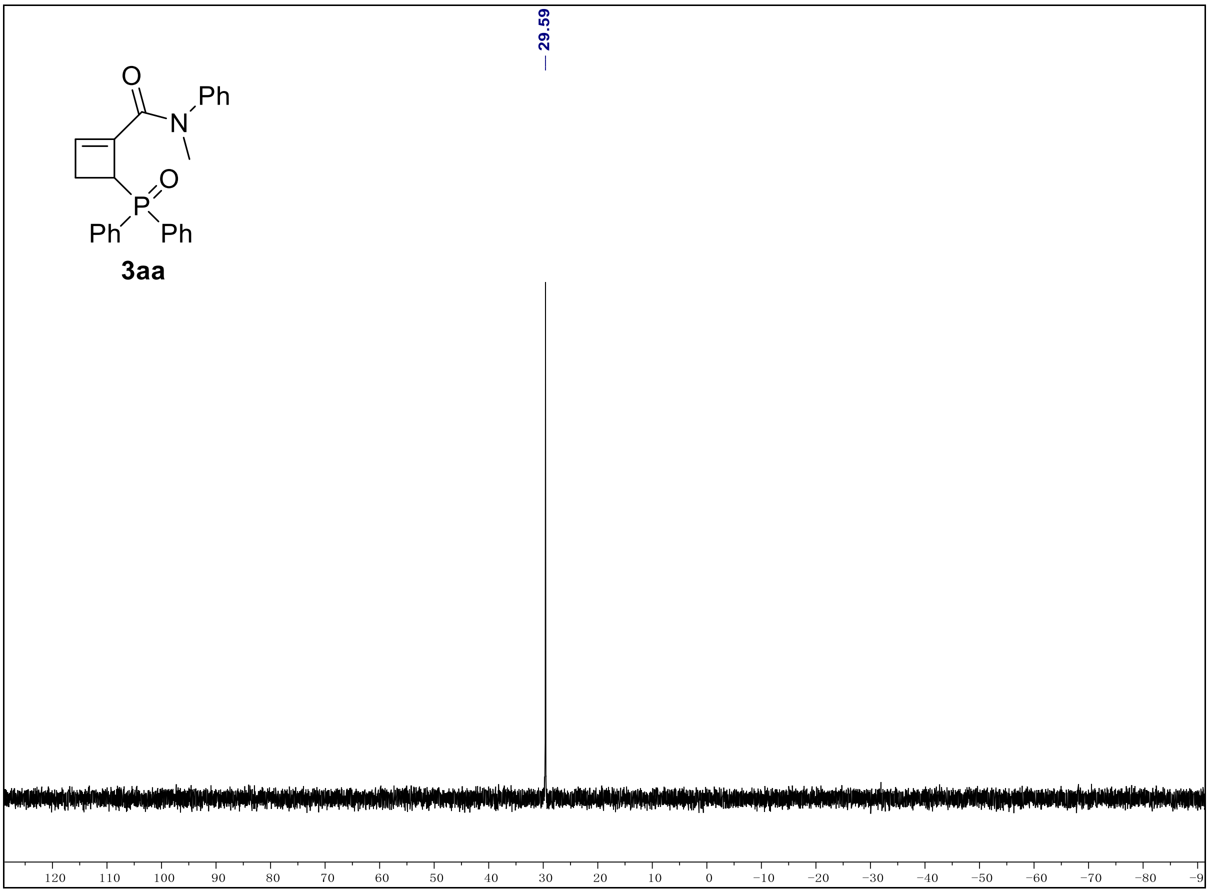
29.59
3aa
120
110
100
90
80
70
60
50
40
30
20
10
0
−10
−20
−30
−40
−50
−60
−70
−80

^{1}H NMR (400 MHz, $CDCl_3$), ^{13}C NMR (100 MHz, $CDCl_3$) and ^{31}P NMR (162 MHz, $CDCl_3$) spectrum of 3ba.

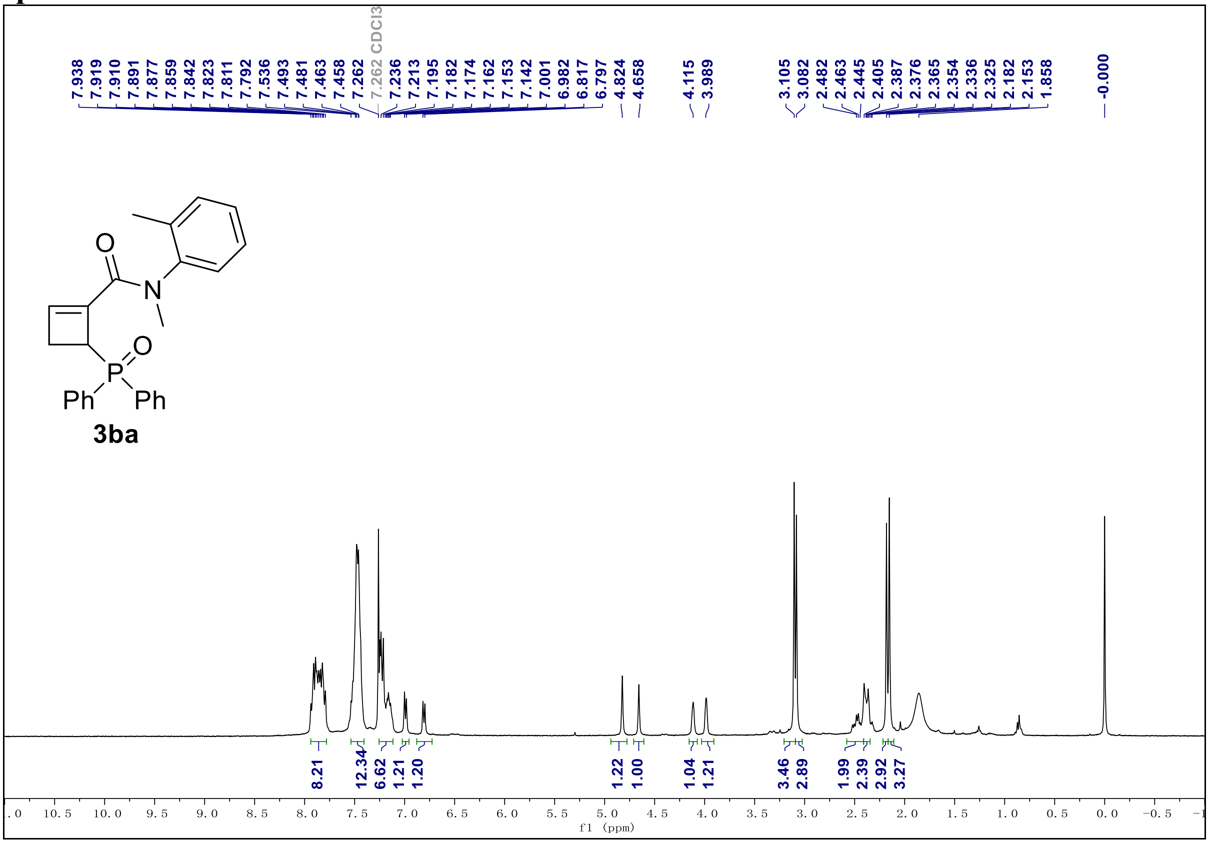

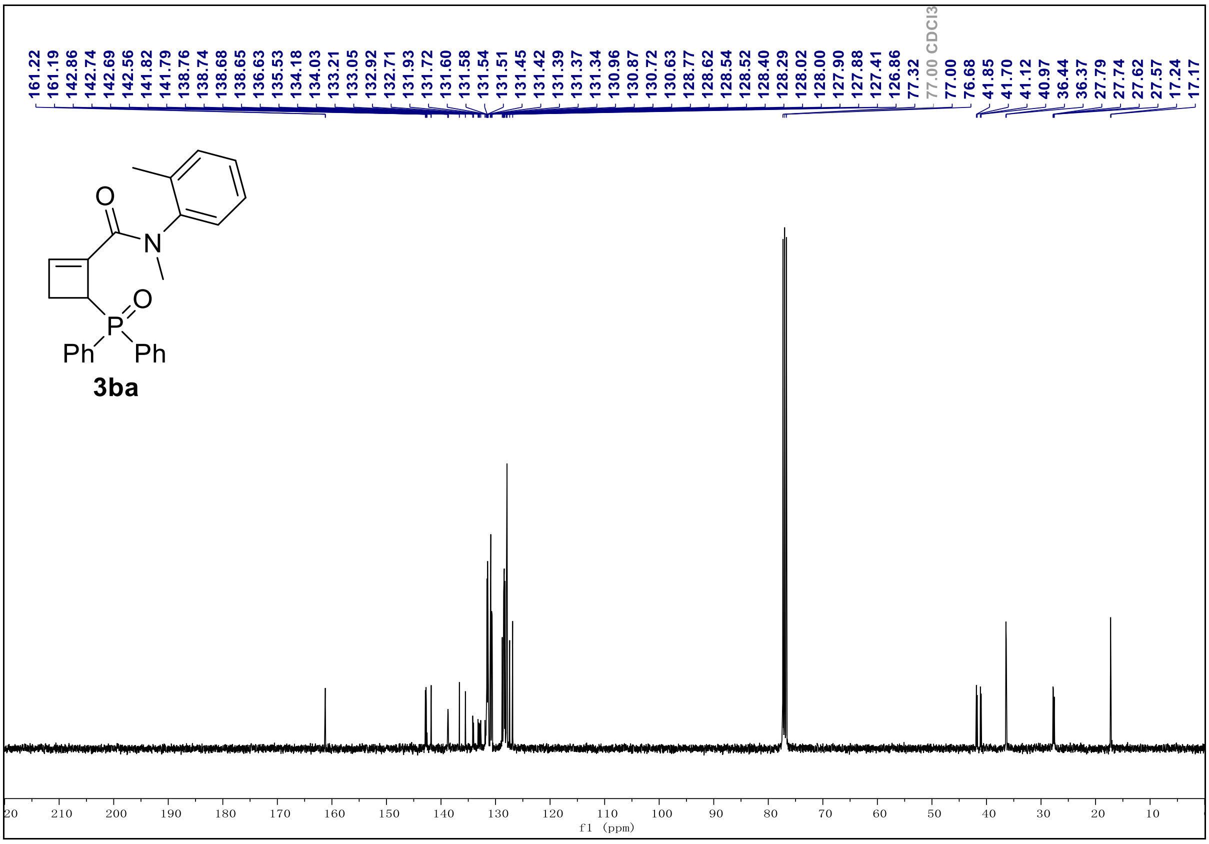

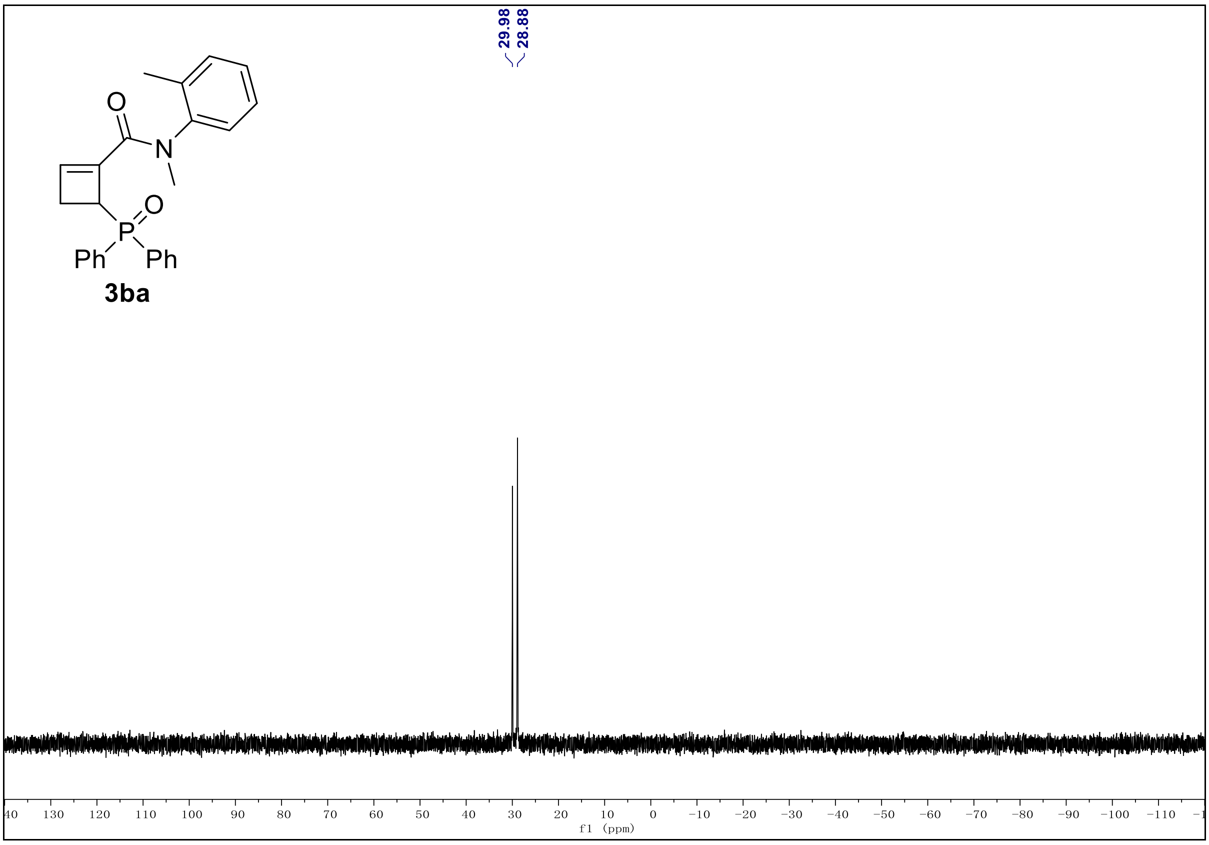
29.98
28.88
O
N
O
P
Ph
Ph
3ba
f1 (ppm)

^{1}H NMR (400 MHz, CDCl$_3$), ^{13}C NMR (100 MHz, CDCl$_3$) and ^{31}P NMR (162 MHz, CDCl$_3$) spectrum of 3ca.

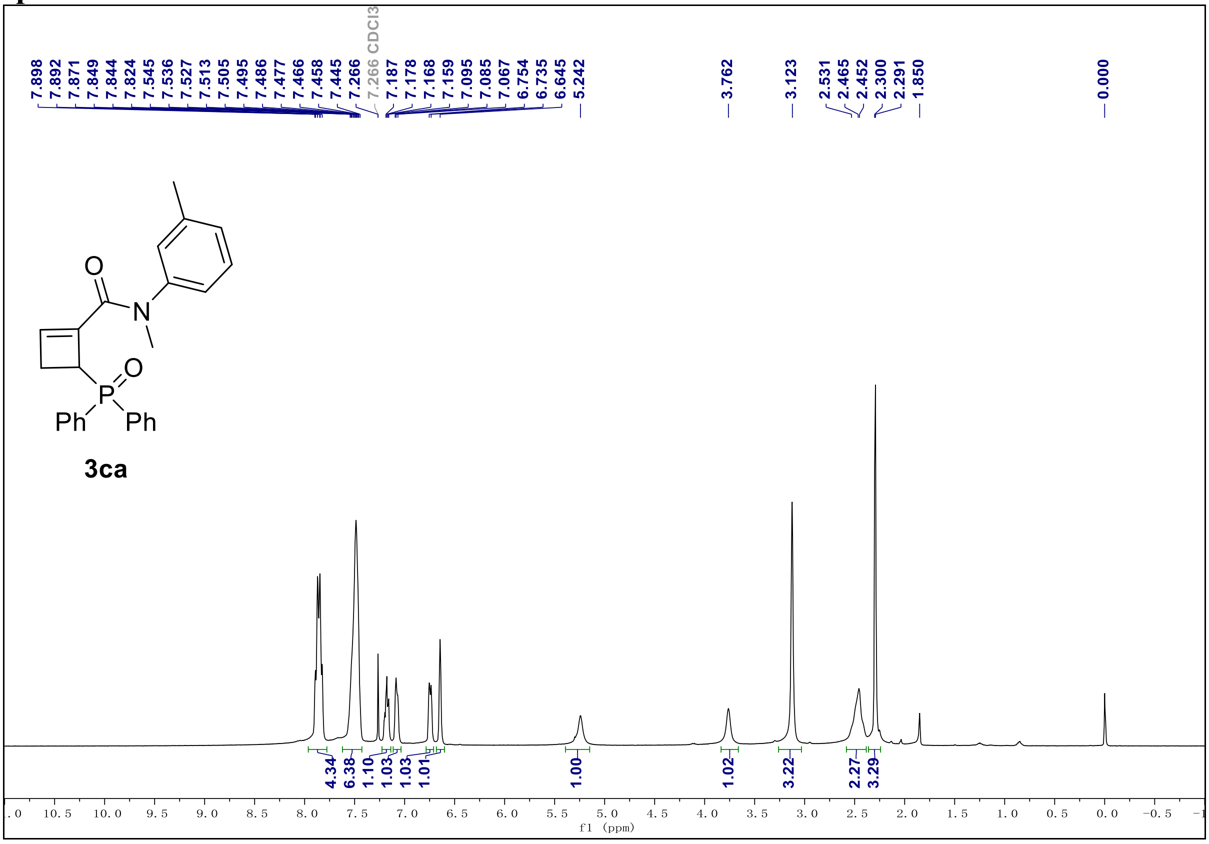

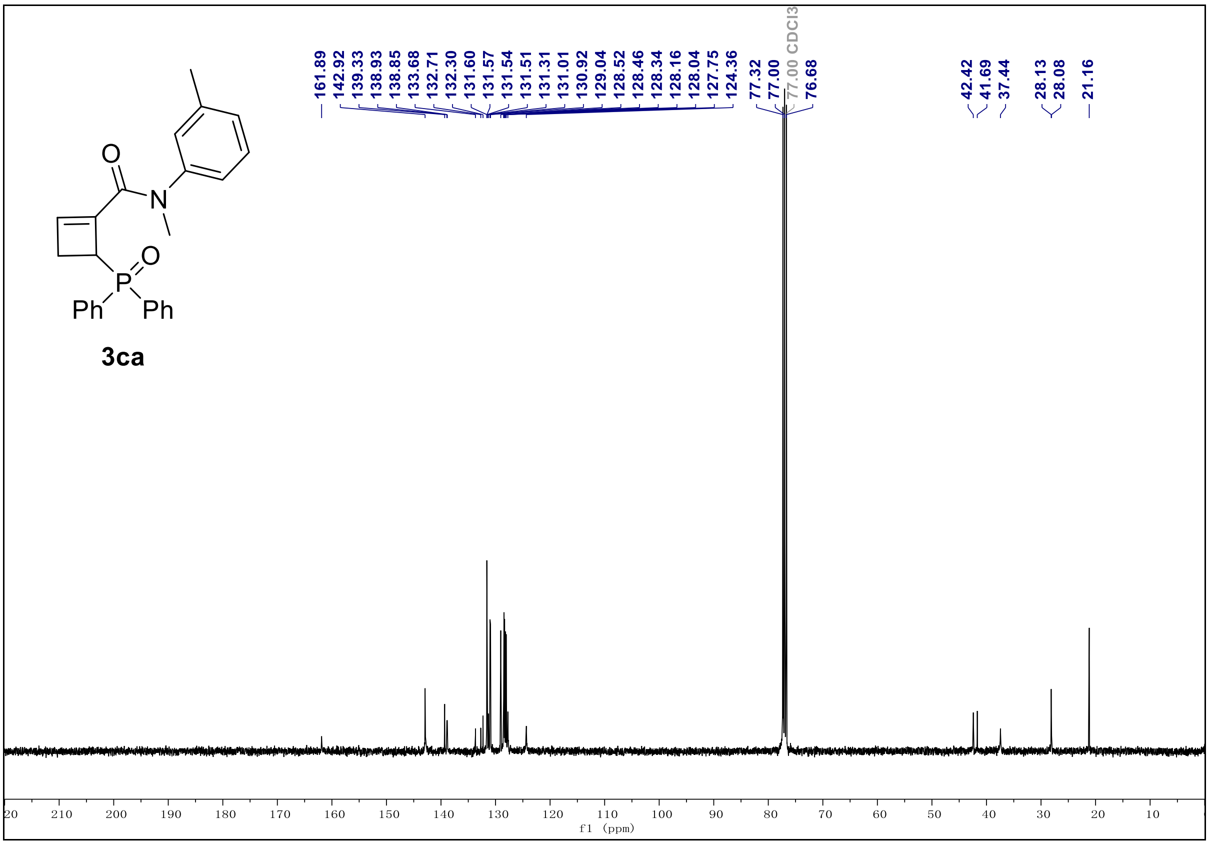

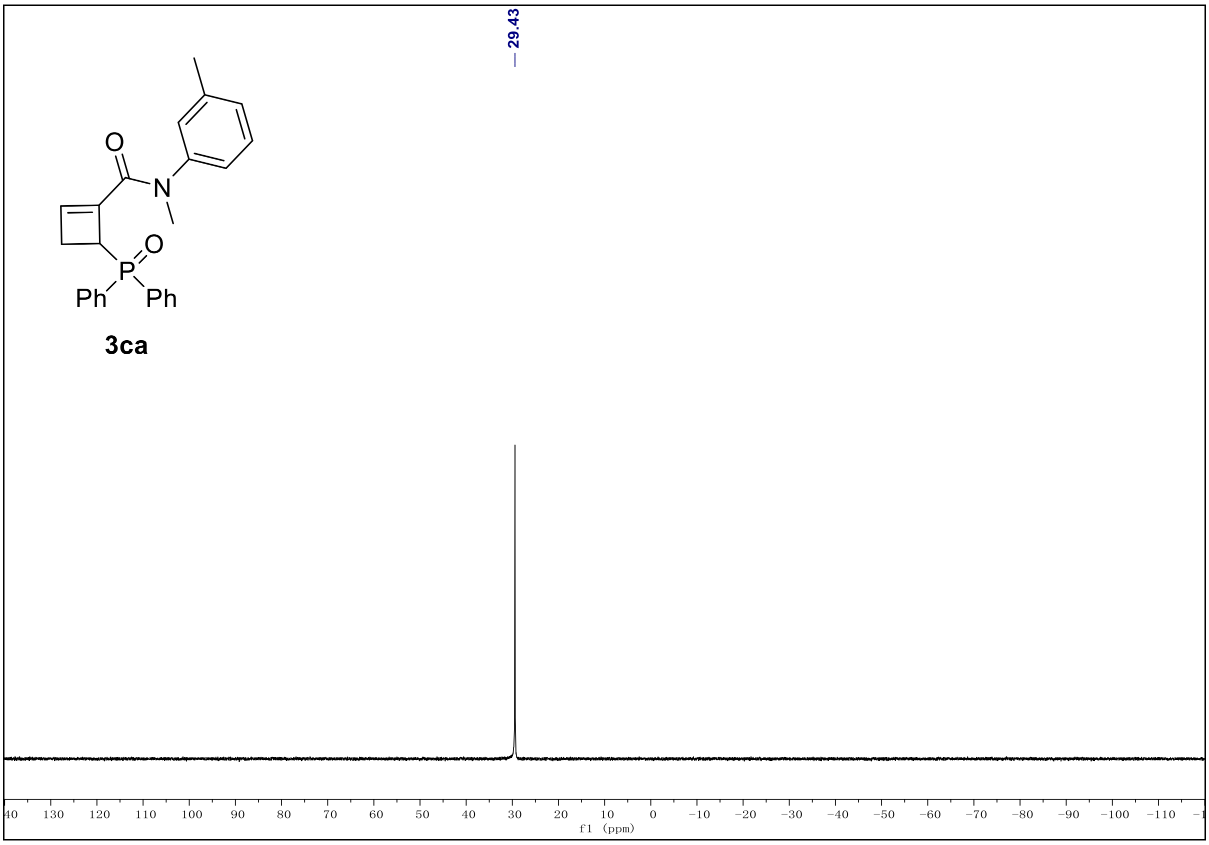
29.43
O
N
O
P
Ph
Ph
3ca
f1 (ppm)

^{1}H NMR (400 MHz, CDCl$_3$), ^{13}C NMR (100 MHz, CDCl$_3$) and ^{31}P NMR (162 MHz, CDCl$_3$) spectrum of 3da.

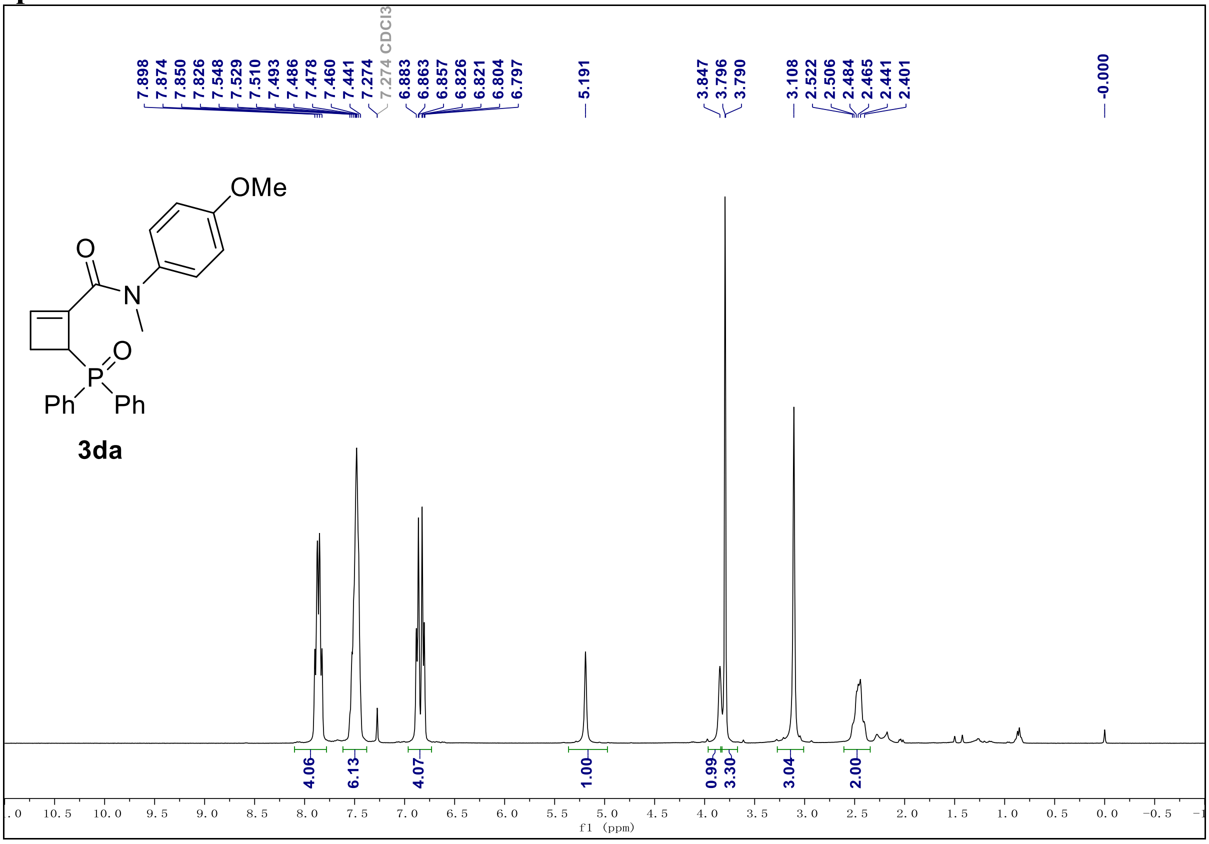

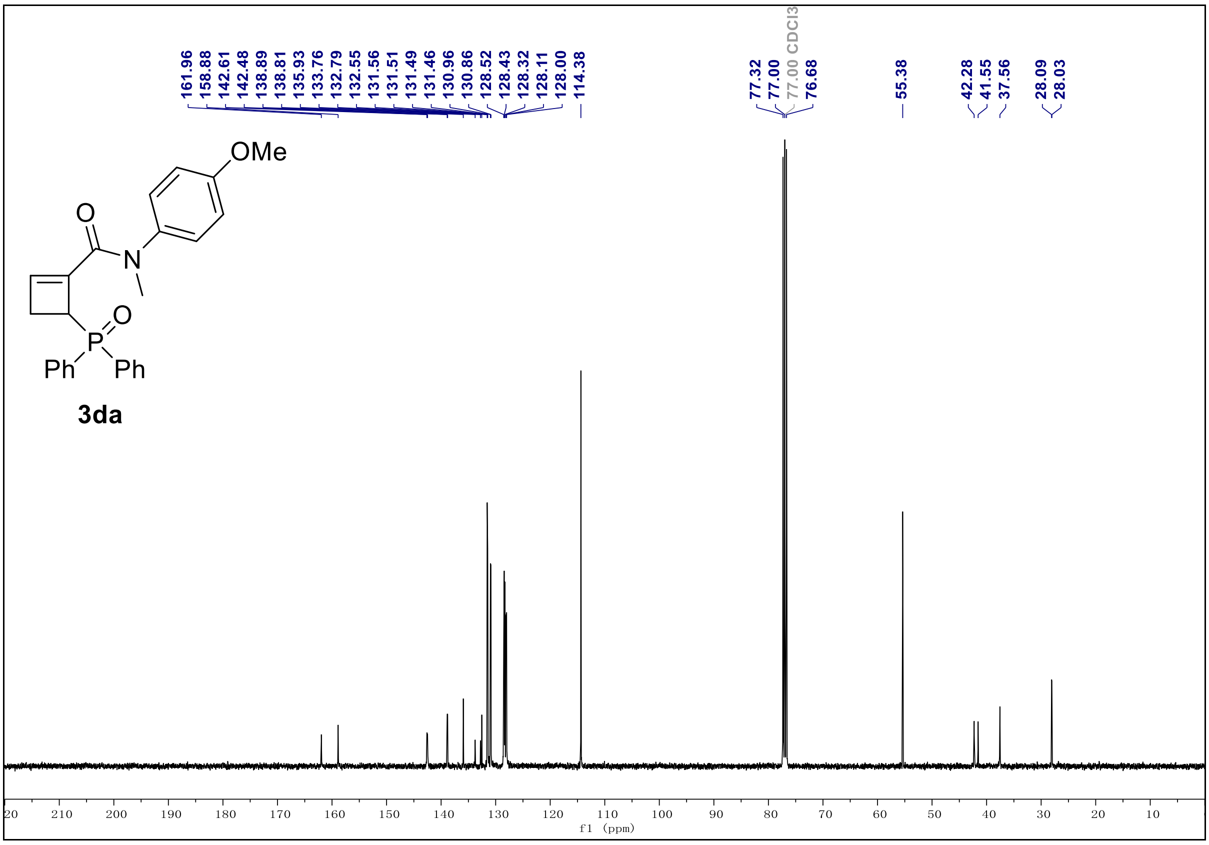

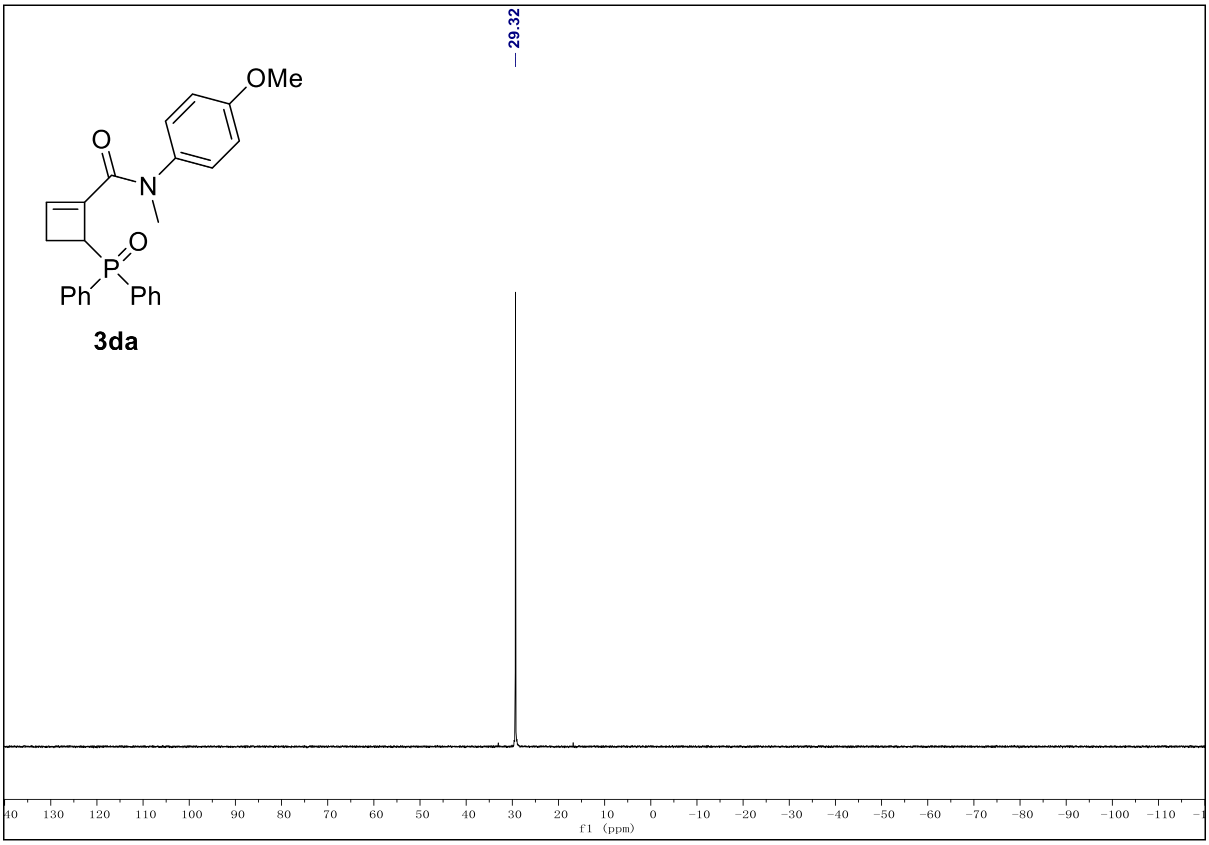

29.32
OMe
O
N
O
P
Ph
Ph
3da
140
130
120
110
100
90
80
70
60
50
40
30
20
10
0
-10
-20
-30
-40
-50
-60
-70
-80
-90
-100
-110
f1 (ppm)

^{1}H NMR (400 MHz, CDCl3), ^{13}C NMR (100 MHz, CDCl3), ^{19}F NMR (376 MHz, CDCl3) and ^{31}P NMR (162 MHz, CDCl3) spectrum of 3ea.

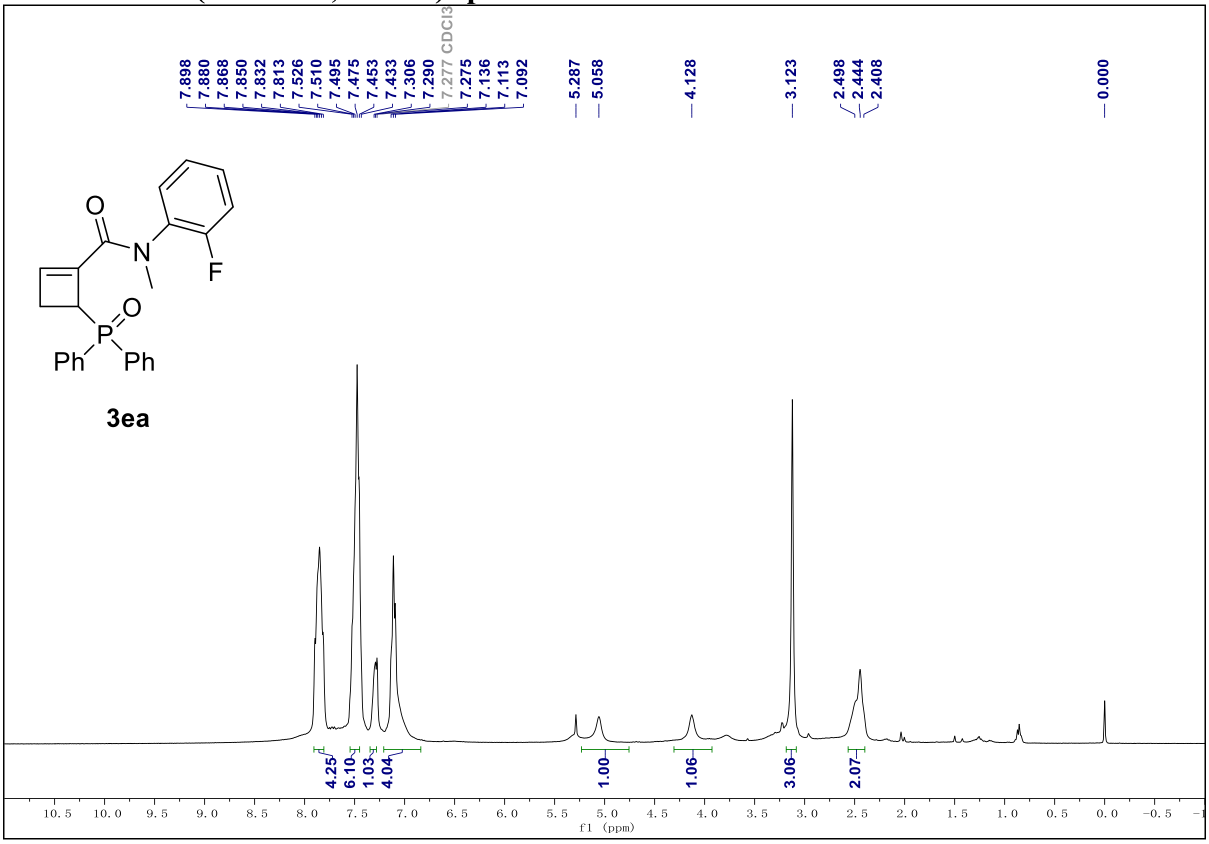

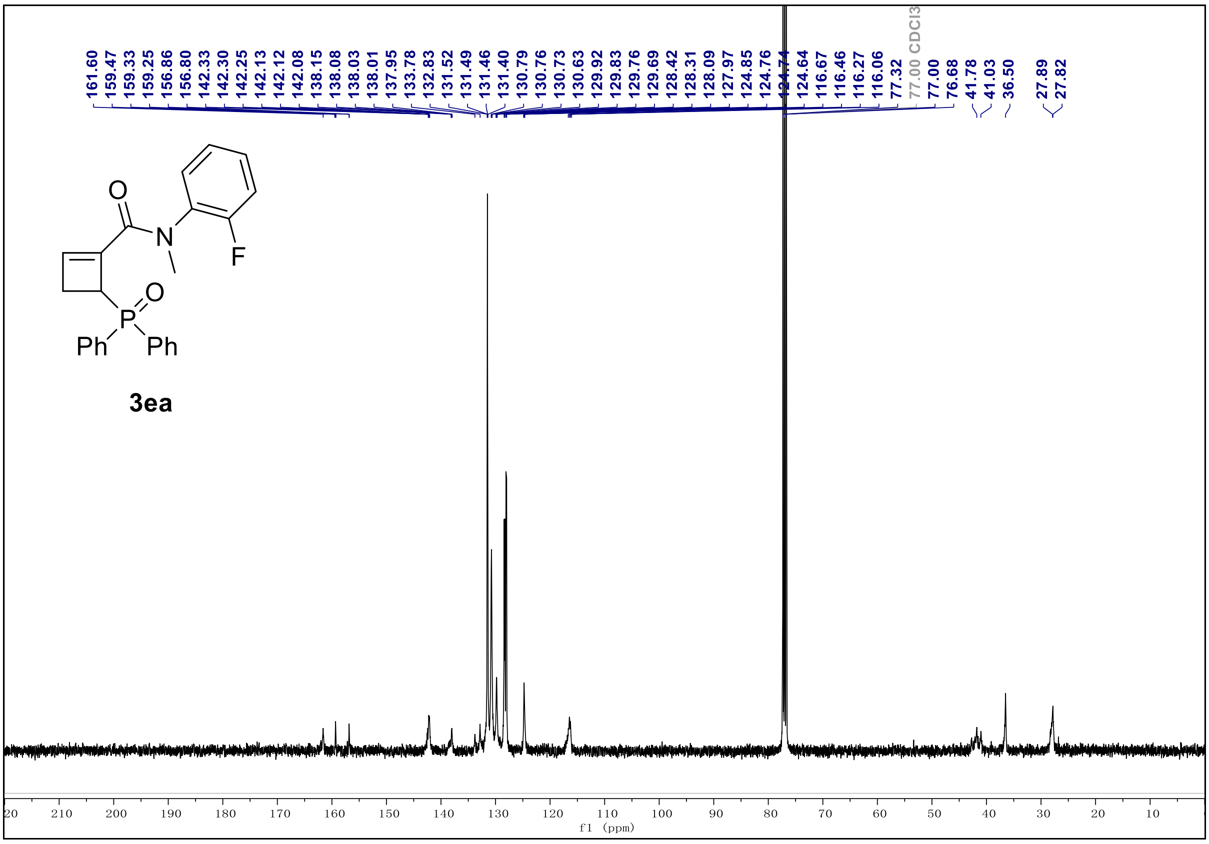

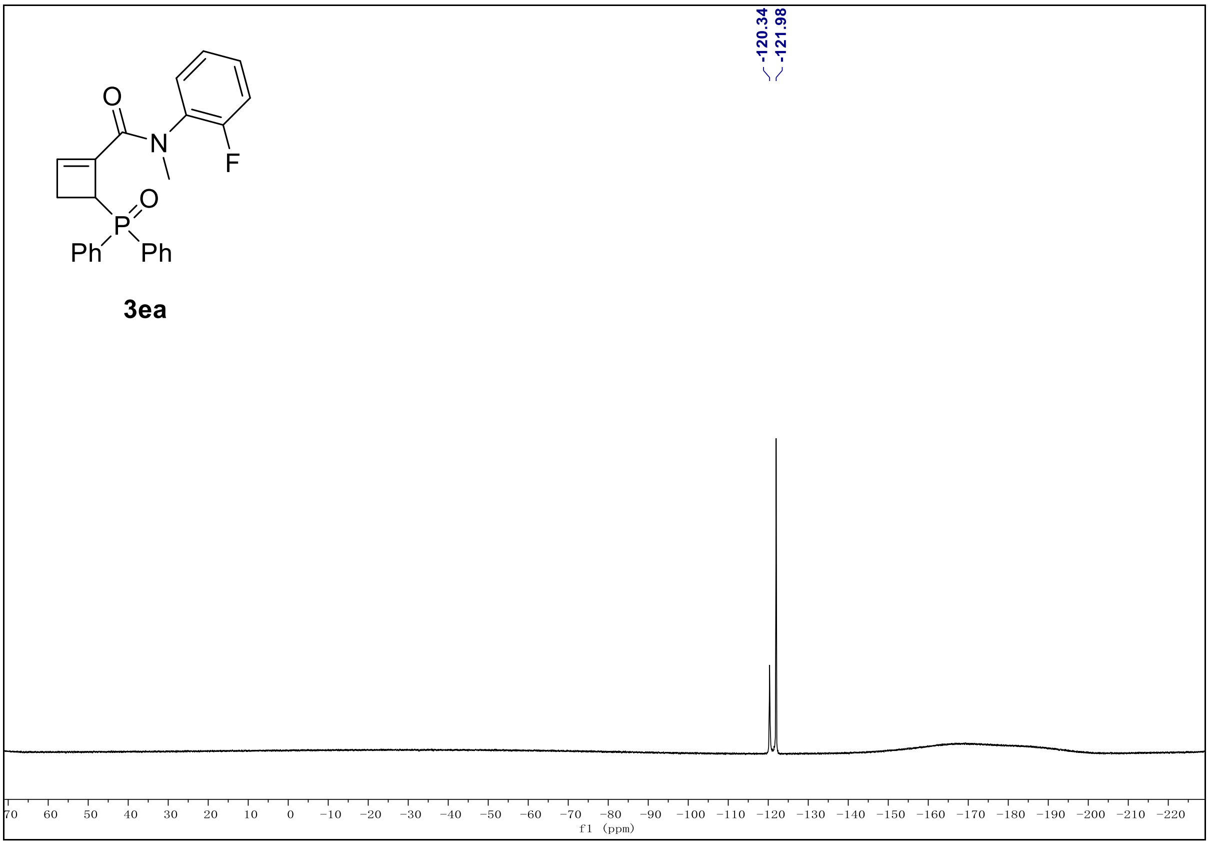
-120.34
-121.98
O
N
F
O
P
Ph
Ph
3ea
70 60 50 40 30 20 10 0 −10 −20 −30 −40 −50 −60 −70 −80 −90 −100 −110 −120 −130 −140 −150 −160 −170 −180 −190 −200 −210 −220
f1 (ppm)

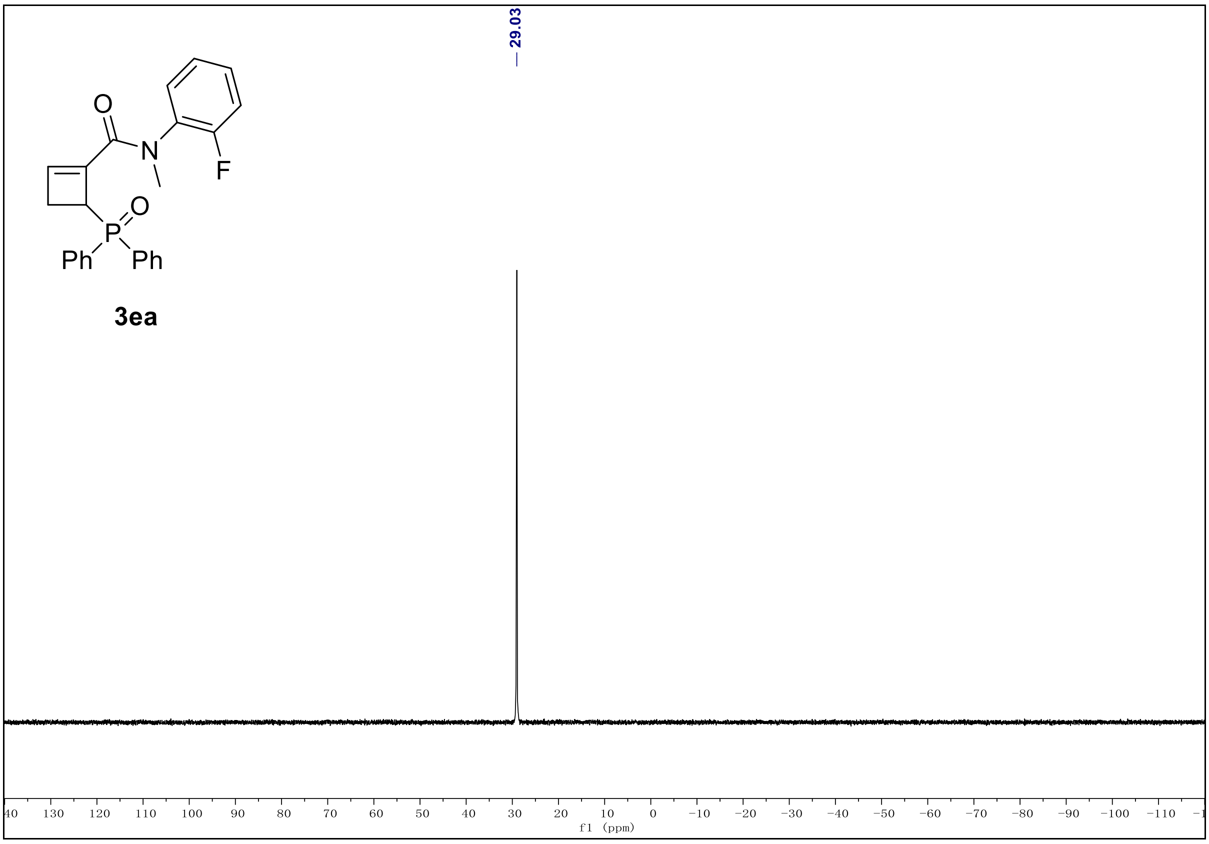
29.03
O
N
F
O
P
Ph
Ph
3ea
140 130 120 110 100 90 80 70 60 50 40 30 20 10 0 −10 −20 −30 −40 −50 −60 −70 −80 −90 −100 −110
f1 (ppm)

^{1}H NMR (400 MHz, $CDCl_3$), ^{13}C NMR (100 MHz, $CDCl_3$) and ^{31}P NMR (162 MHz, $CDCl_3$) spectrum of 3fa.

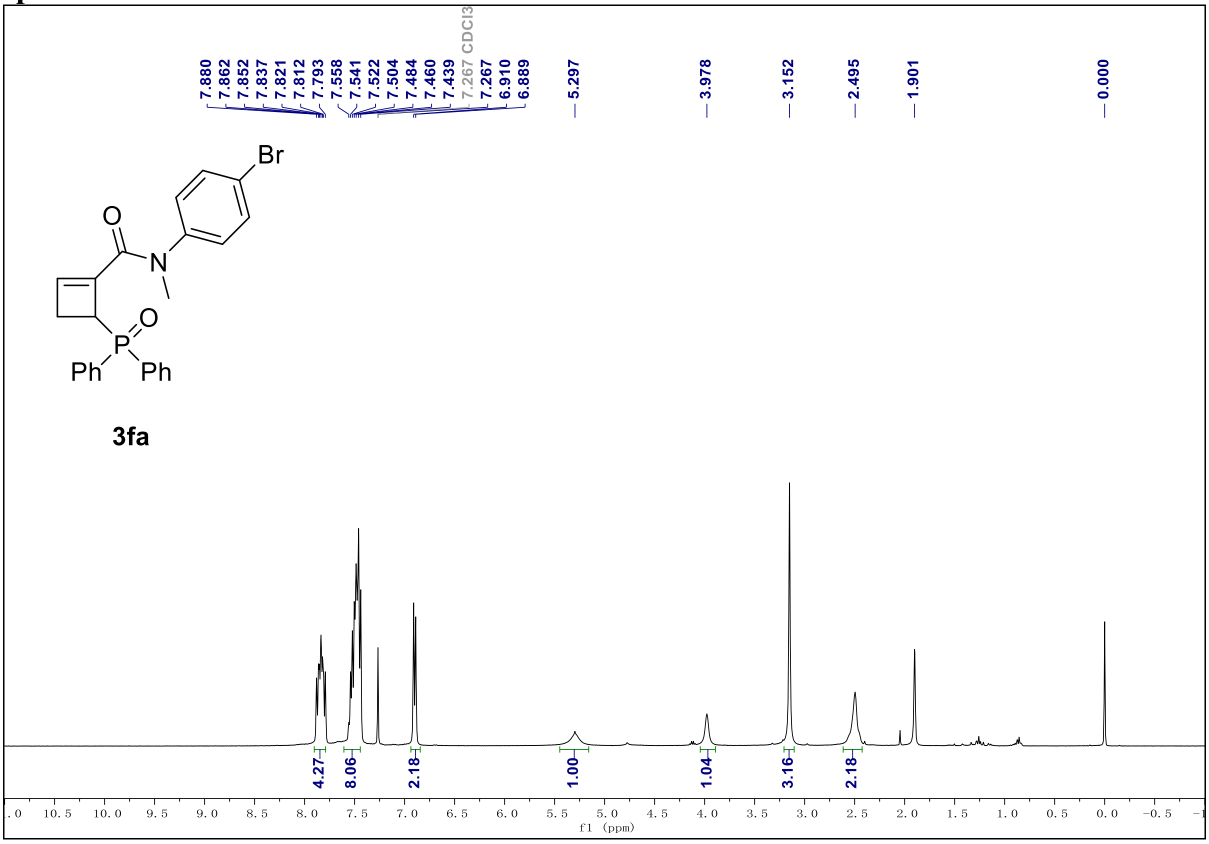

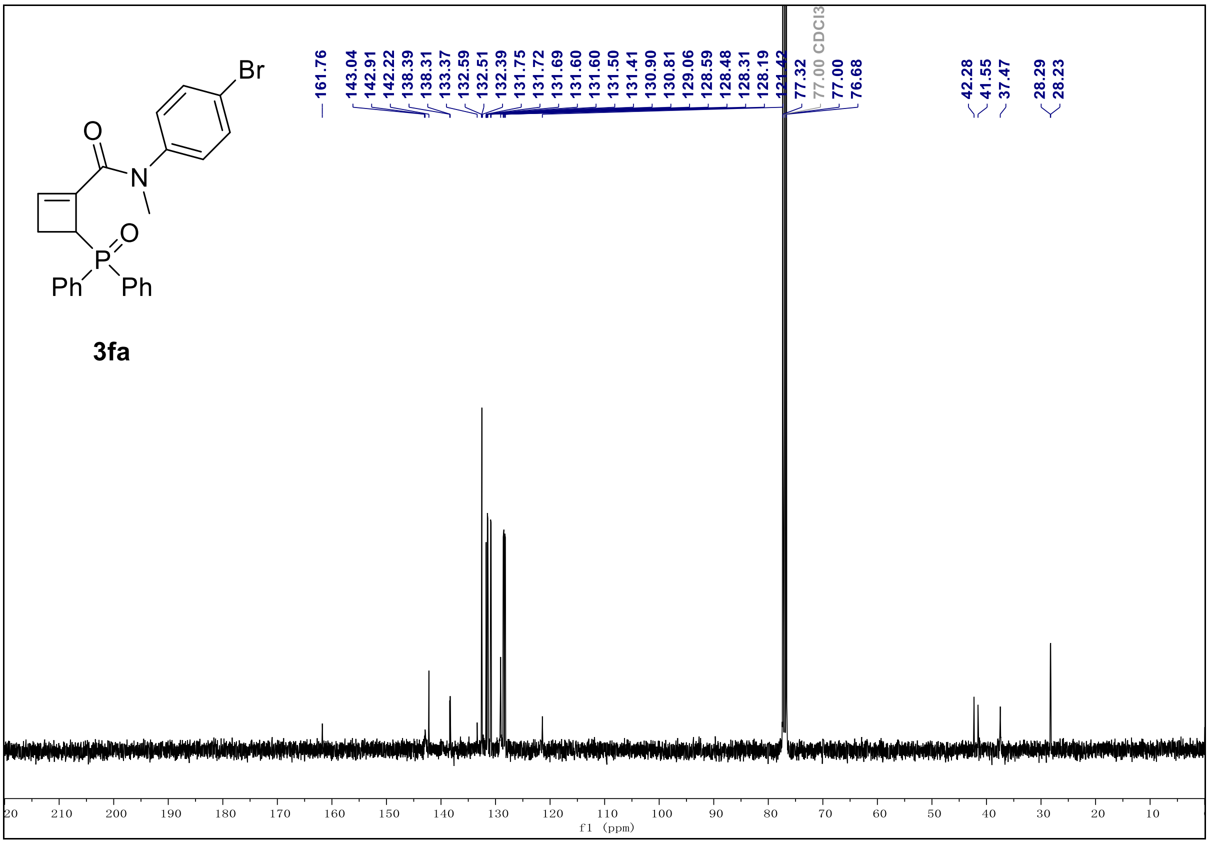

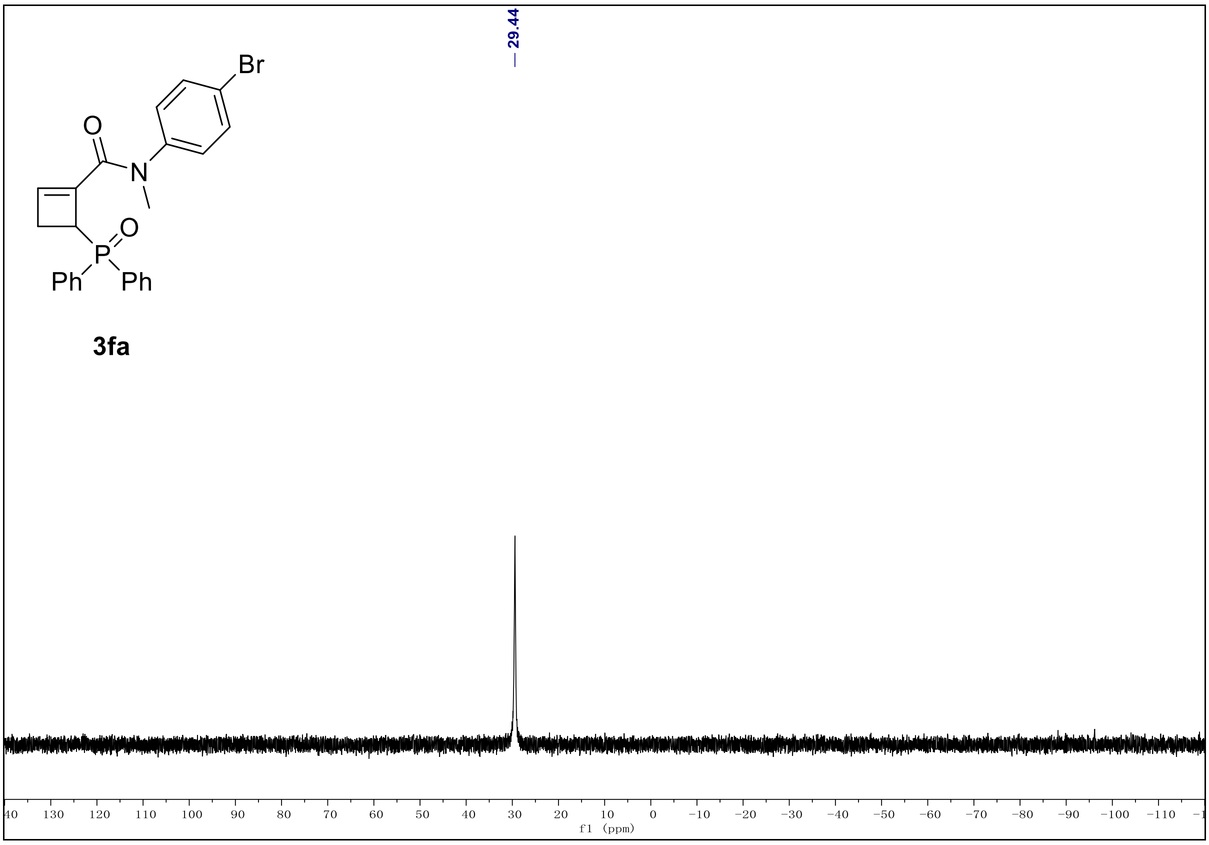

29.44
Br
O
N
O
P
Ph
Ph
3fa
f1 (ppm)

^{1}H NMR (400 MHz, CDCl3), ^{13}C NMR (100 MHz, CDCl3) and ^{31}P NMR (162 MHz, CDCl3) spectrum of 3ga.

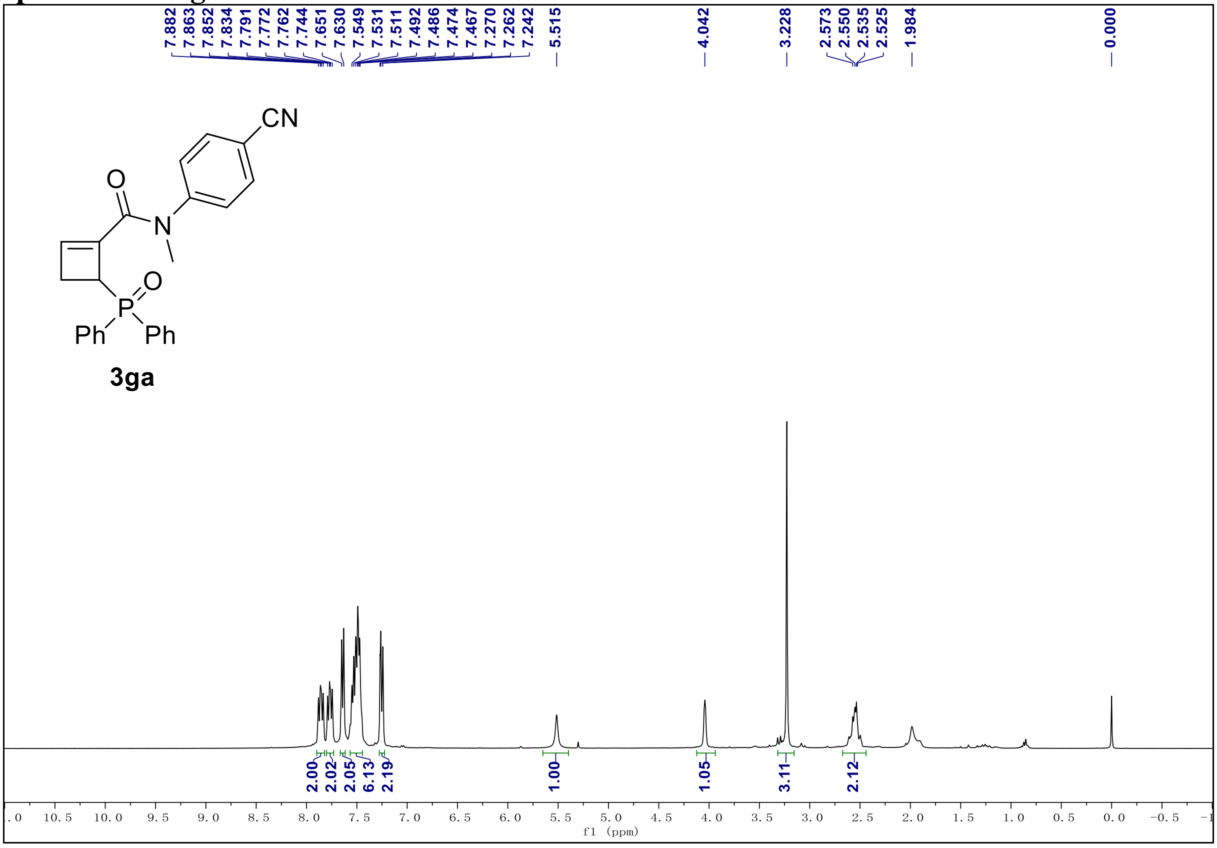

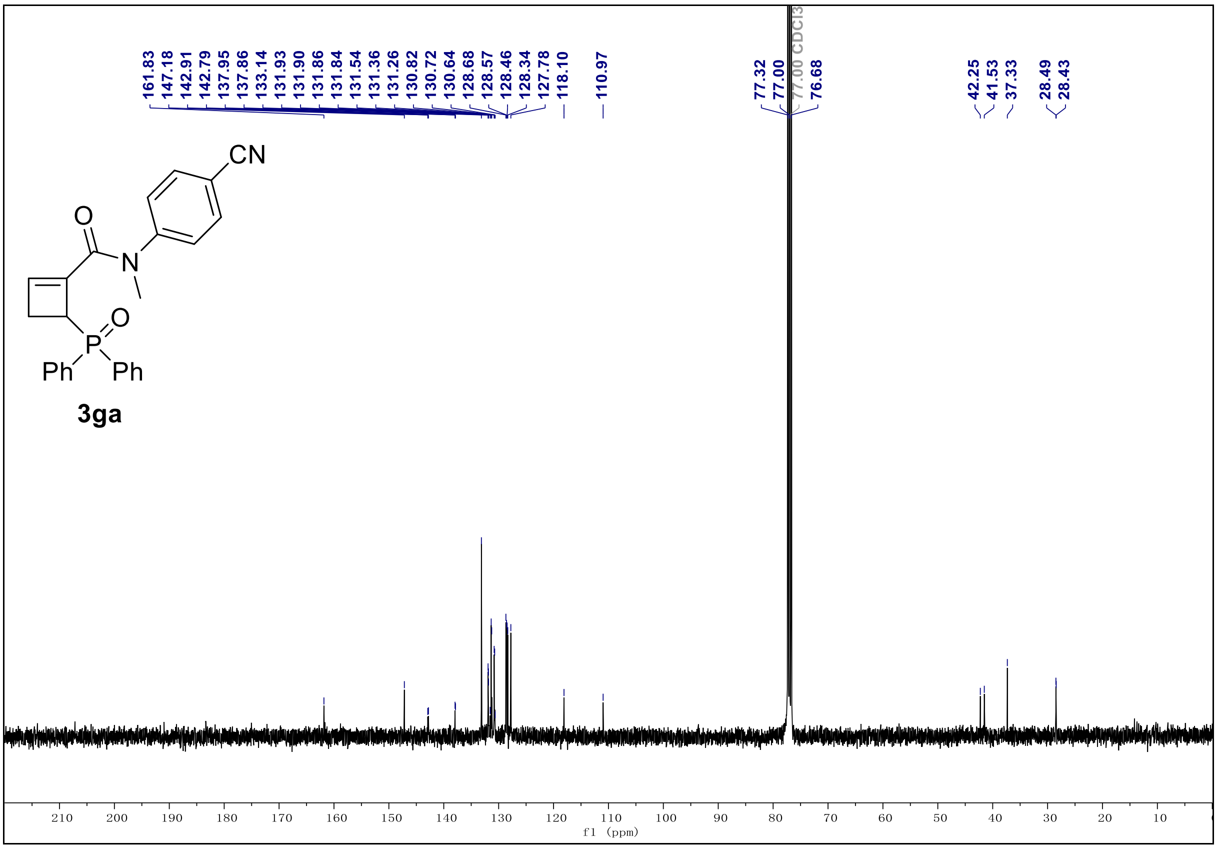

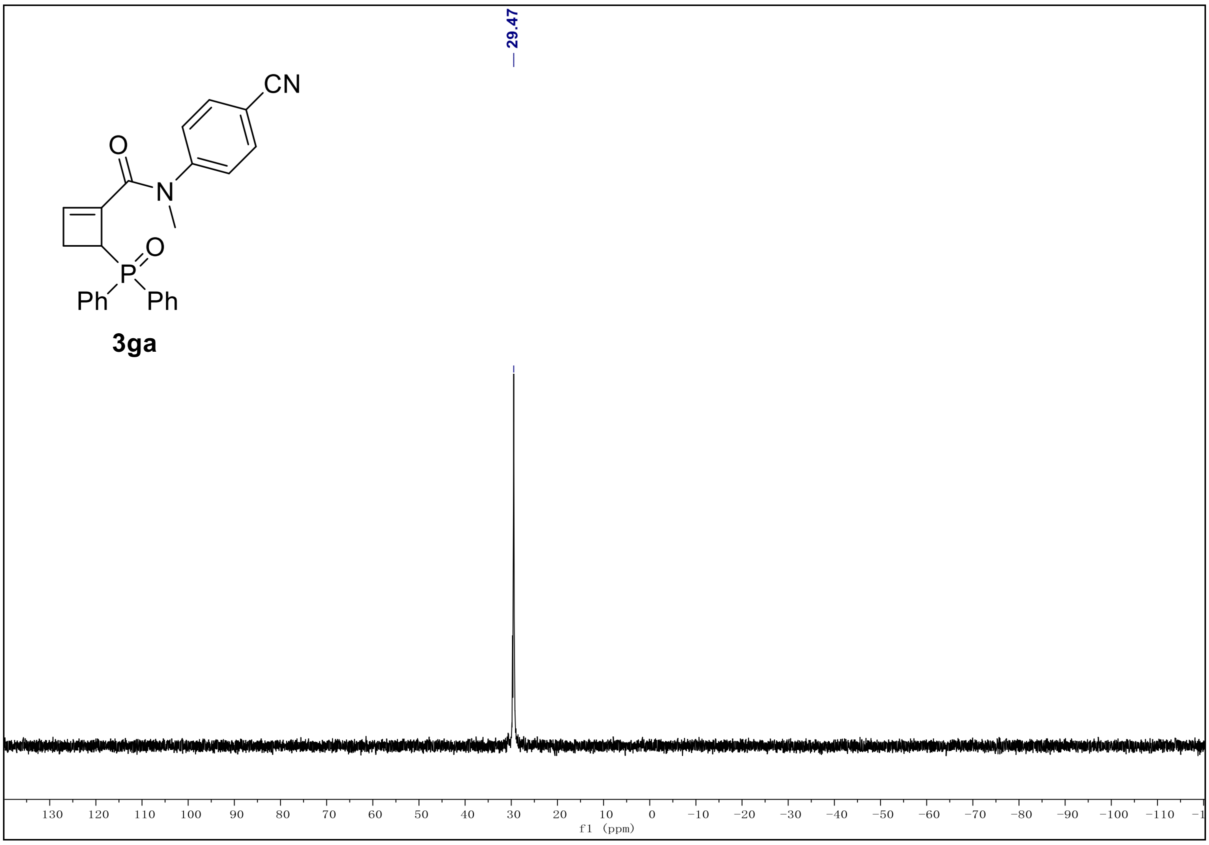
29.47
CN
O
N
P
O
Ph
Ph
3ga
130 120 110 100 90 80 70 60 50 40 30 20 10 0 −10 −20 −30 −40 −50 −60 −70 −80 −90 −100 −110
f1 (ppm)

^{1}H NMR (400 MHz, CDCl3), ^{13}C NMR (100 MHz, CDCl3) and ^{31}P NMR (162 MHz, CDCl3) spectrum of 3ha.

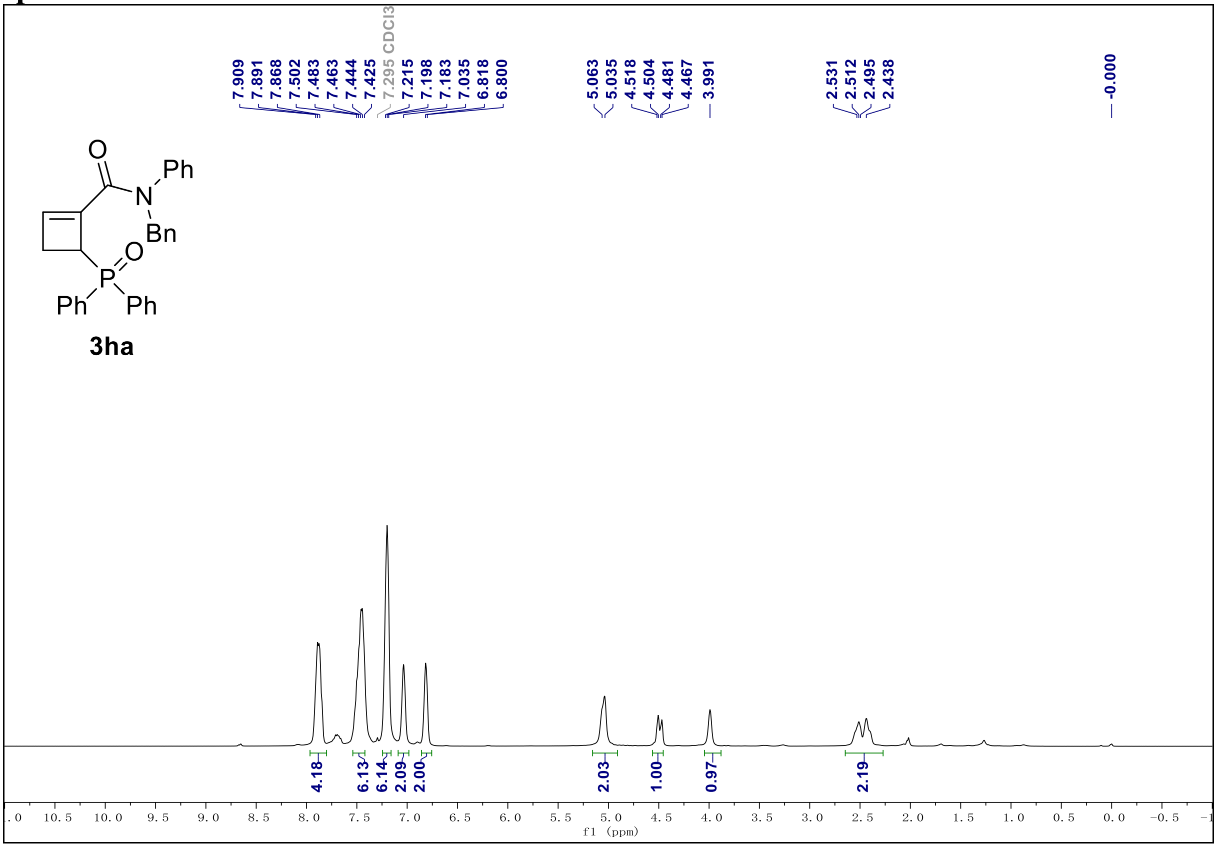

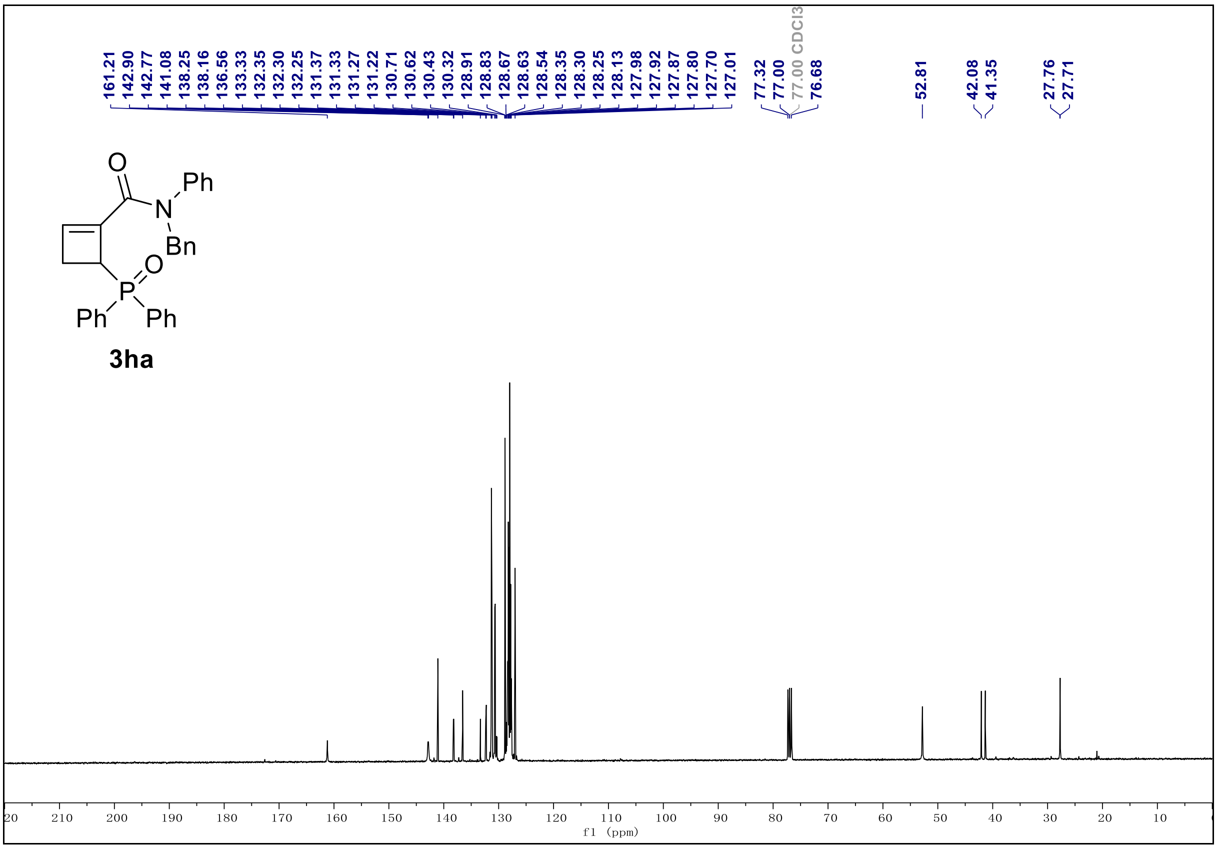

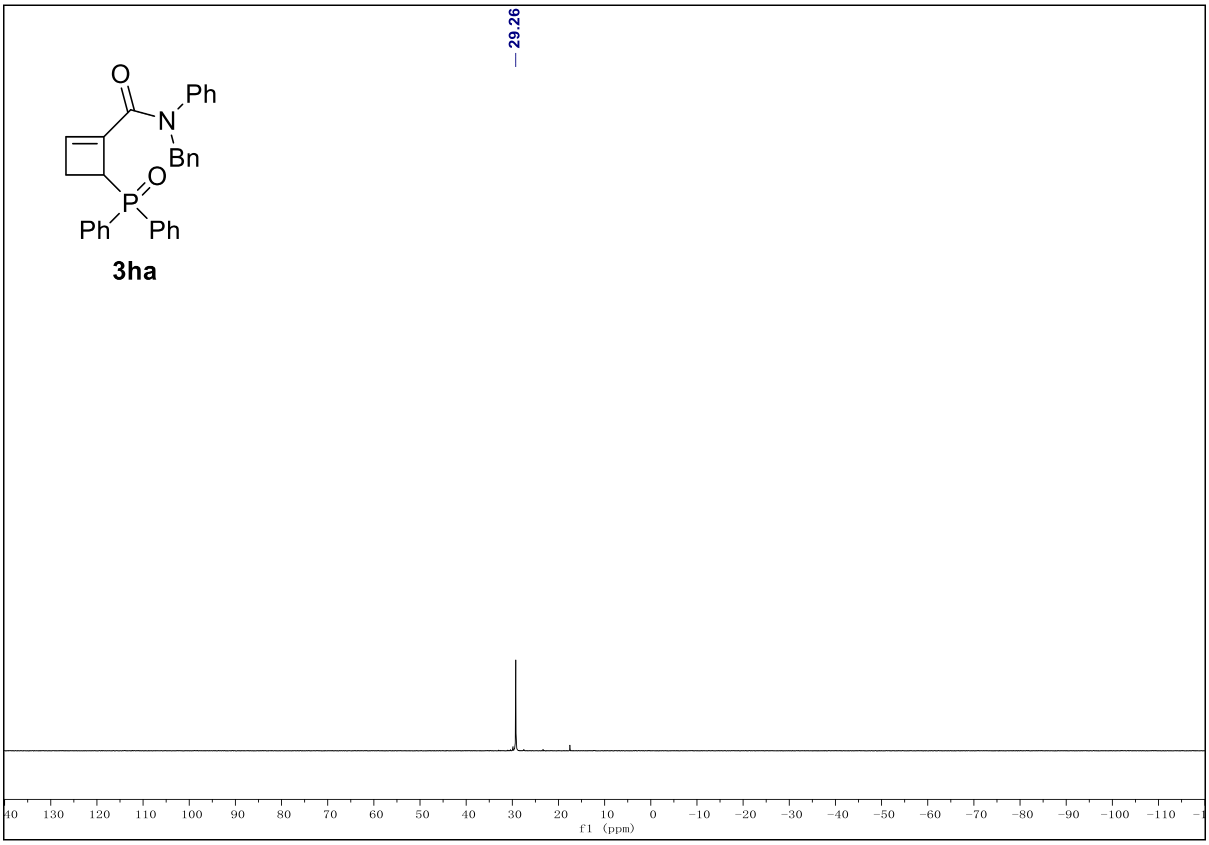
29.26
3ha
f1 (ppm)

^{1}H NMR (400 MHz, CDCl$_3$), ^{13}C NMR (100 MHz, CDCl$_3$) and ^{31}P NMR (162 MHz, CDCl$_3$) spectrum of 3ia.

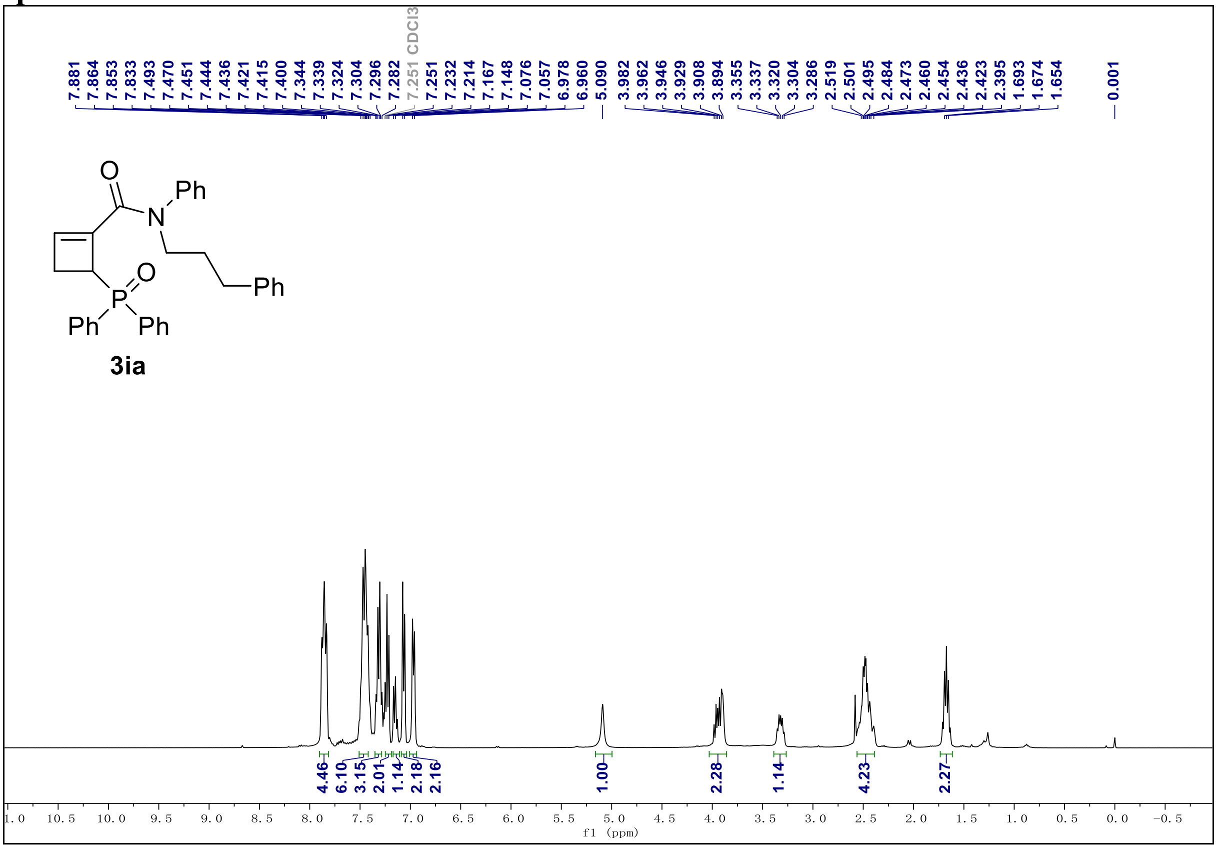

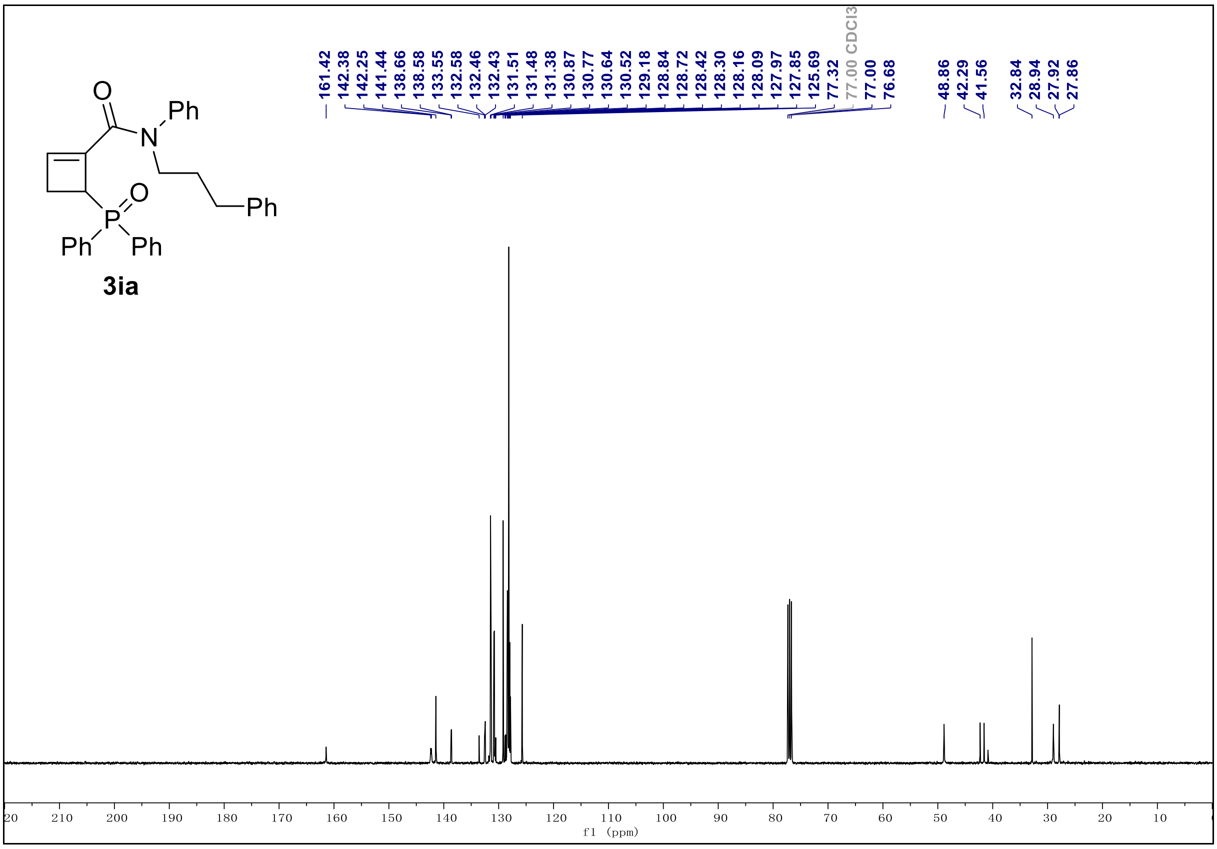

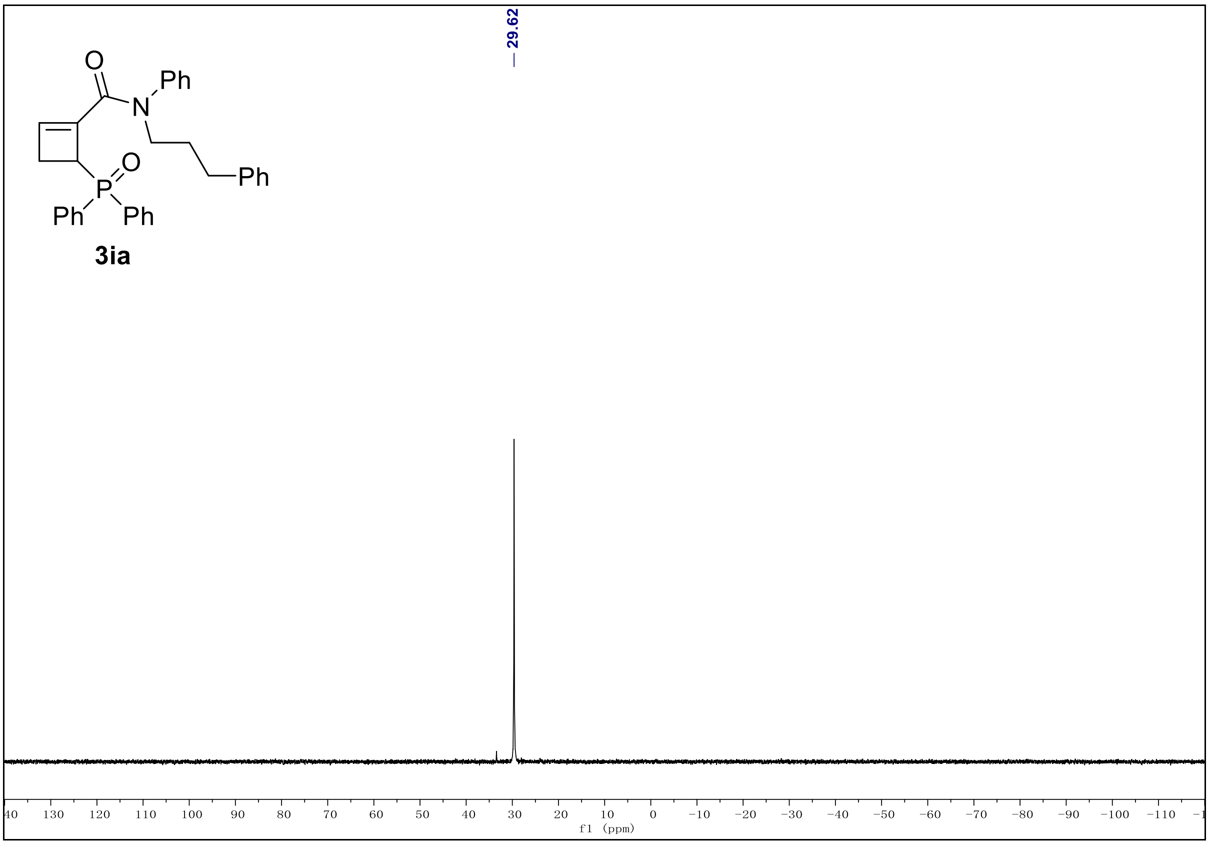
29.62
O
Ph
N
O
P
Ph
Ph
Ph
3ia
40
130
120
110
100
90
80
70
60
50
40
30
20
10
0
−10
−20
−30
−40
−50
−60
−70
−80
−90
−100
−110
f1 (ppm)

^{1}H NMR (400 MHz, $CDCl_3$), ^{13}C NMR (100 MHz, $CDCl_3$) and ^{31}P NMR (162 MHz, $CDCl_3$) spectrum of 3ja.

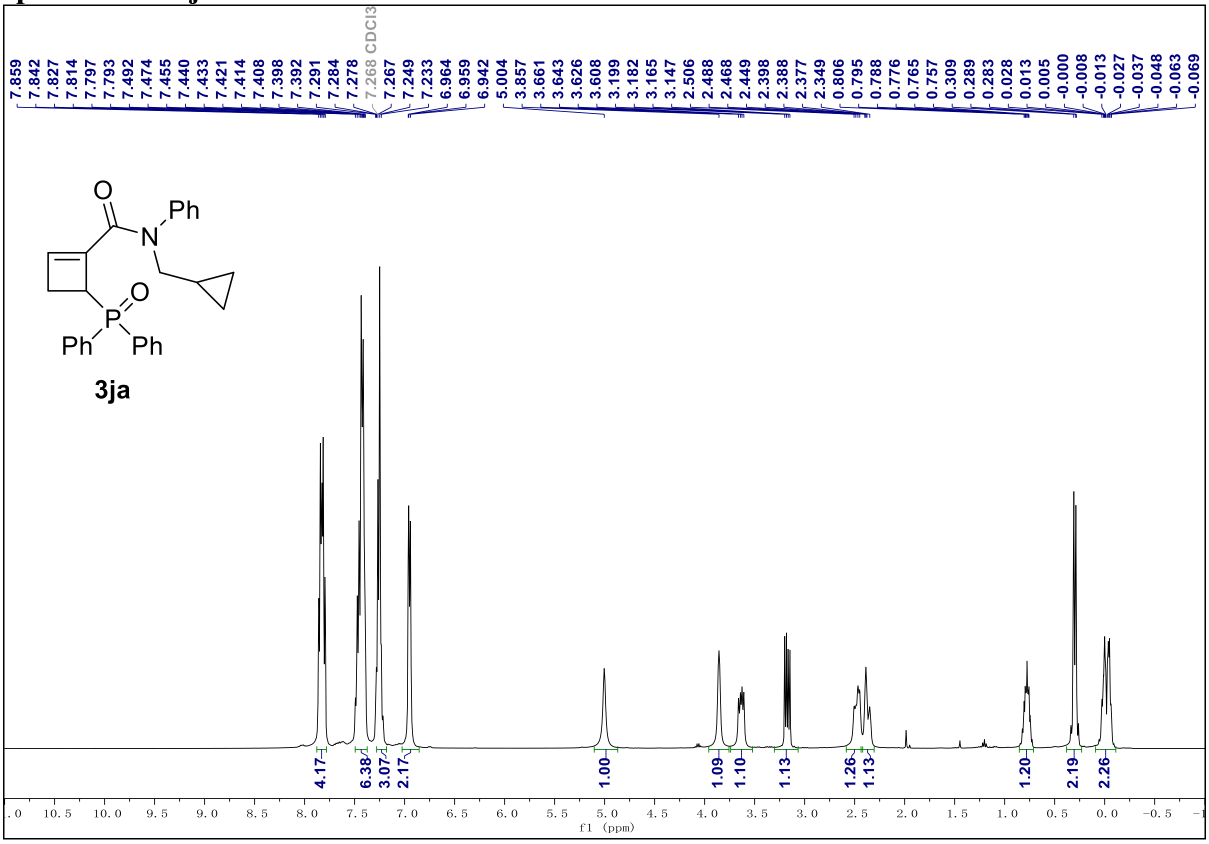

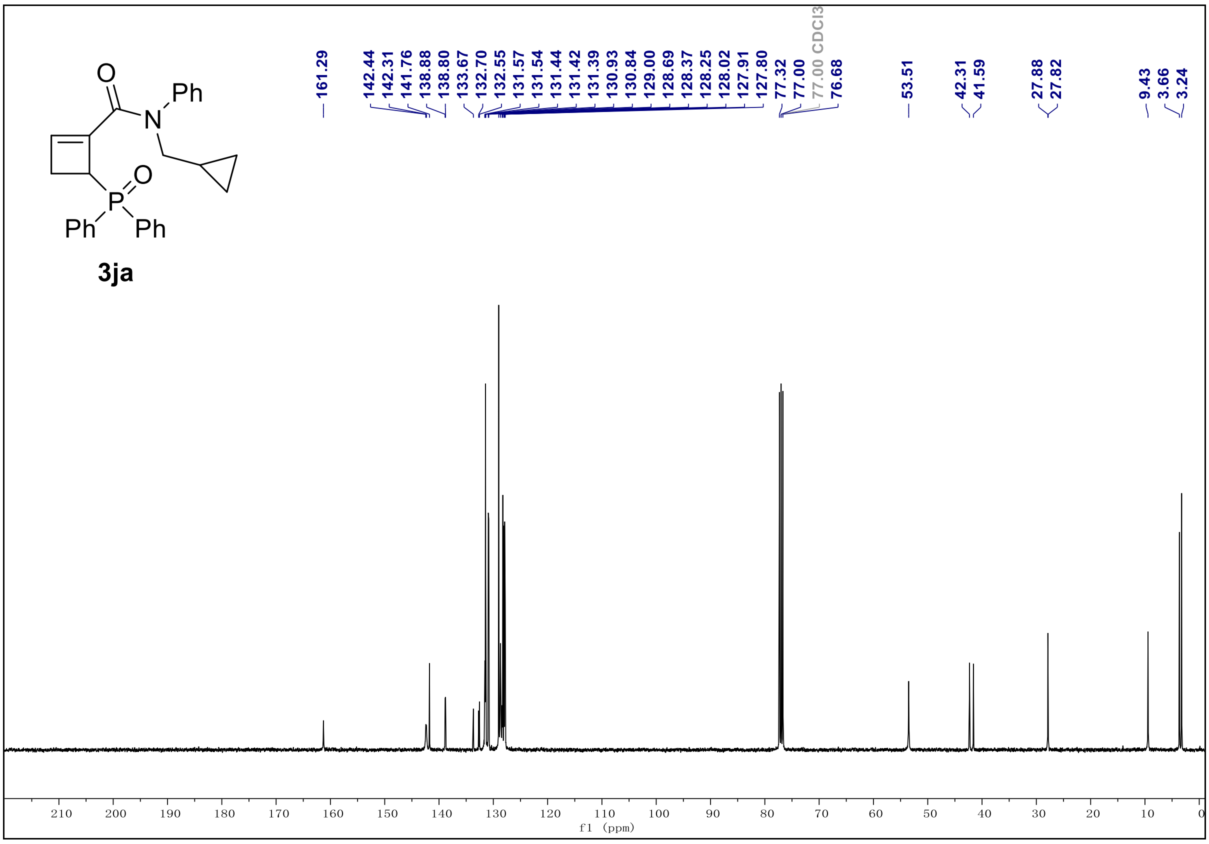

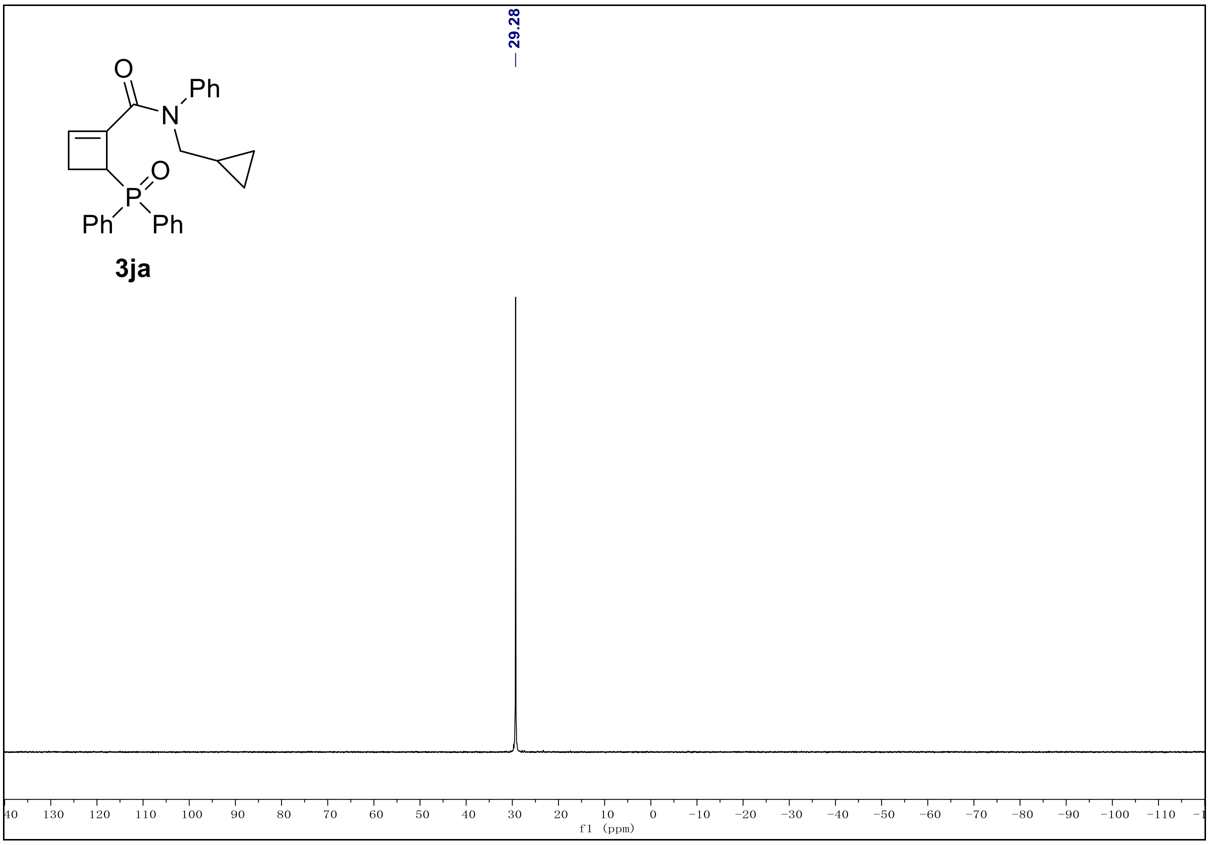
29.28
O
Ph
N
O
P
Ph
Ph
3ja
f1 (ppm)

^{1}H NMR (400 MHz, CDCl$_3$), ^{13}C NMR (100 MHz, CDCl$_3$) and ^{31}P NMR (162 MHz, CDCl$_3$) spectrum of 3ka.

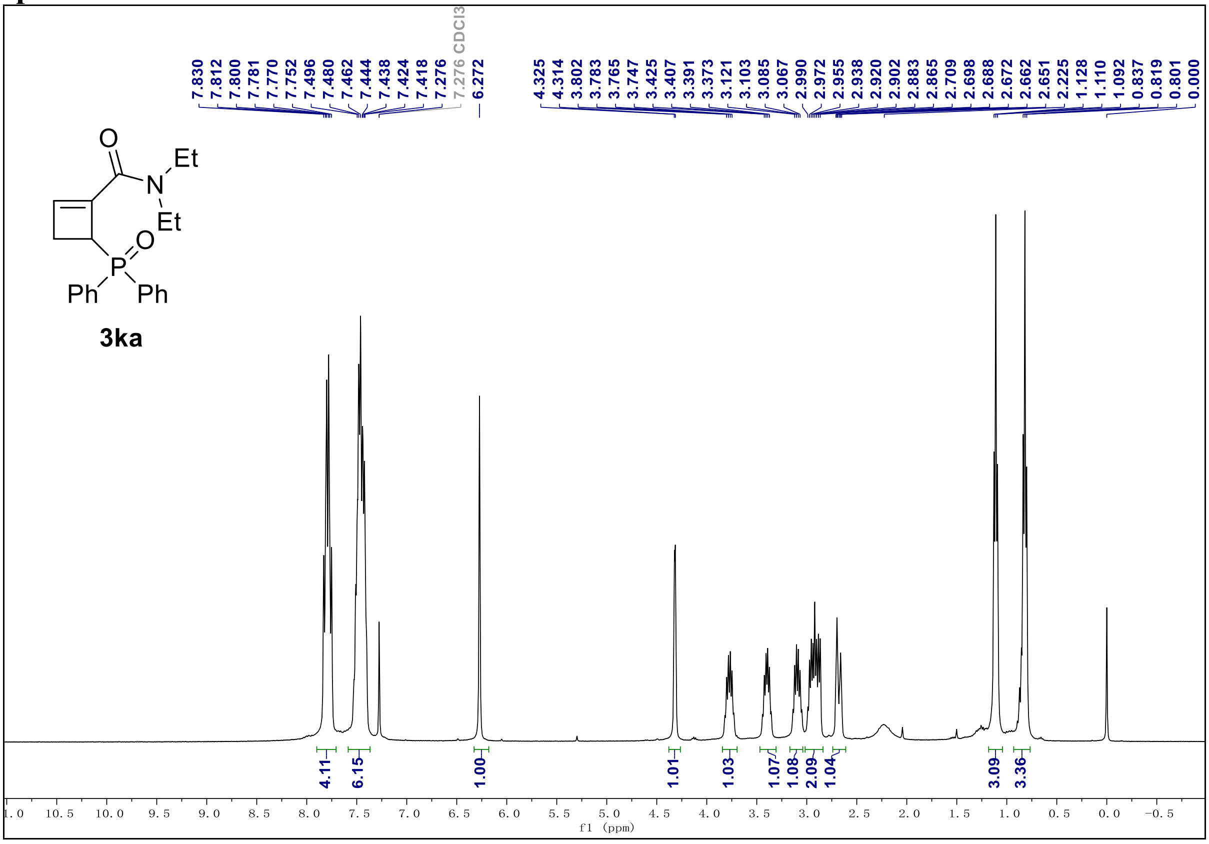

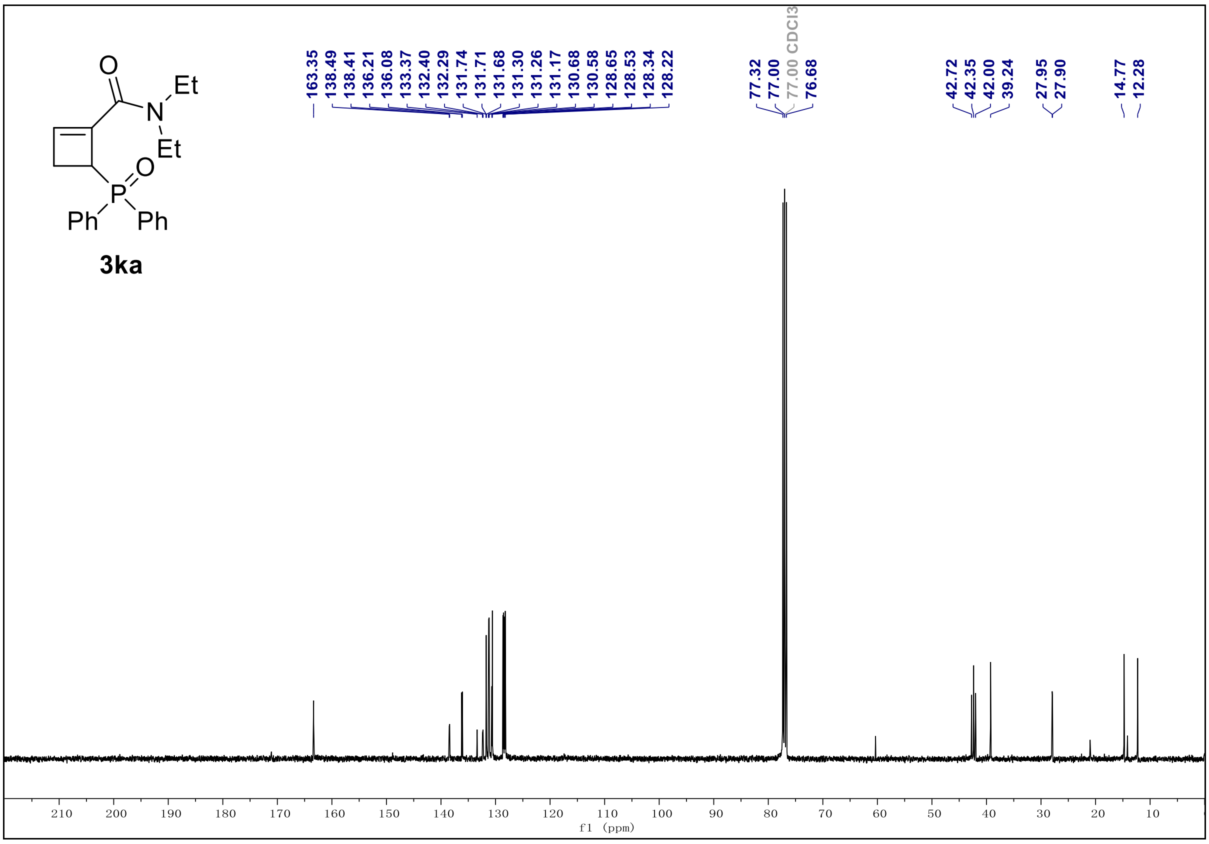

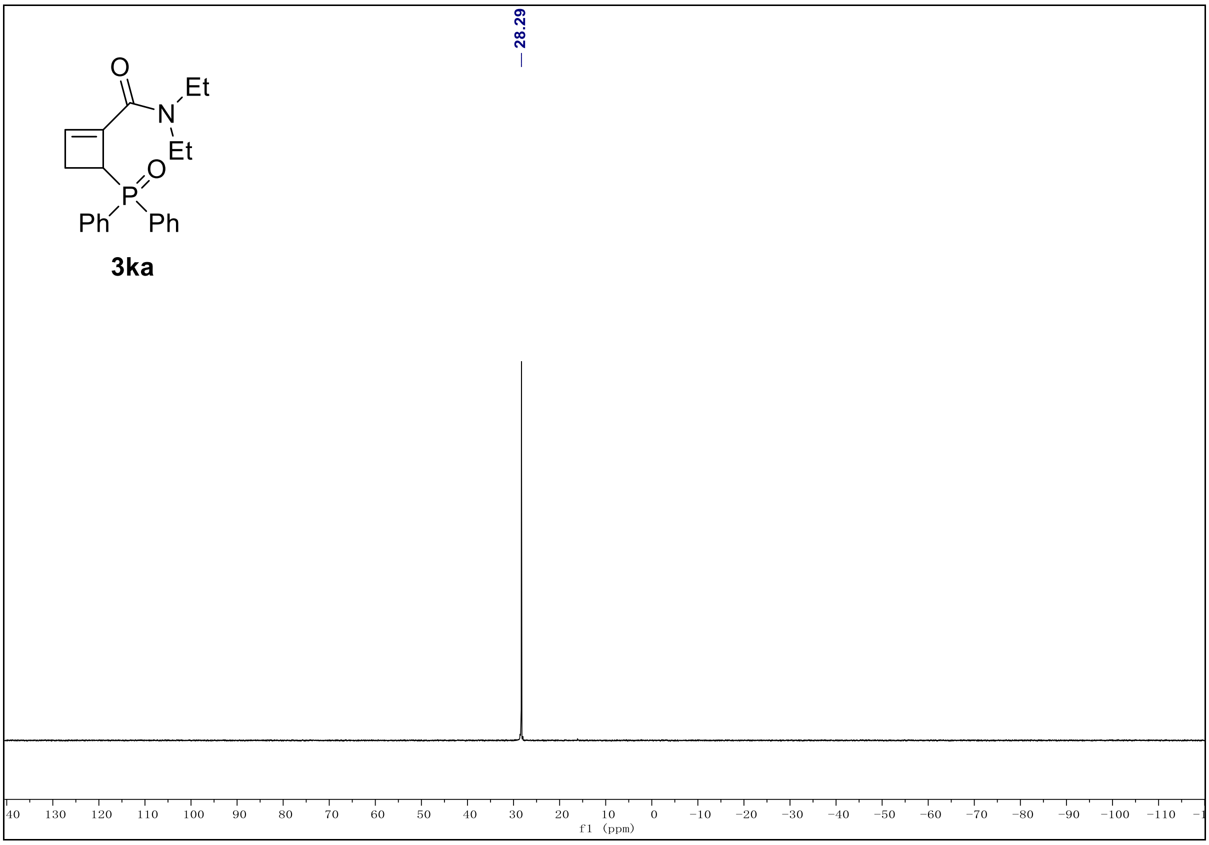

3ka
Et
N
O
Et
O
P
Ph
Ph
28.29
40 130 120 110 100 90 80 70 60 50 40 30 20 10 0 −10 −20 −30 −40 −50 −60 −70 −80 −90 −100 −110
f1 (ppm)

^{1}H NMR (400 MHz, CDCl$_3$), ^{13}C NMR (100 MHz, CDCl$_3$) and ^{31}P NMR (162 MHz, CDCl$_3$) spectrum of 3la.

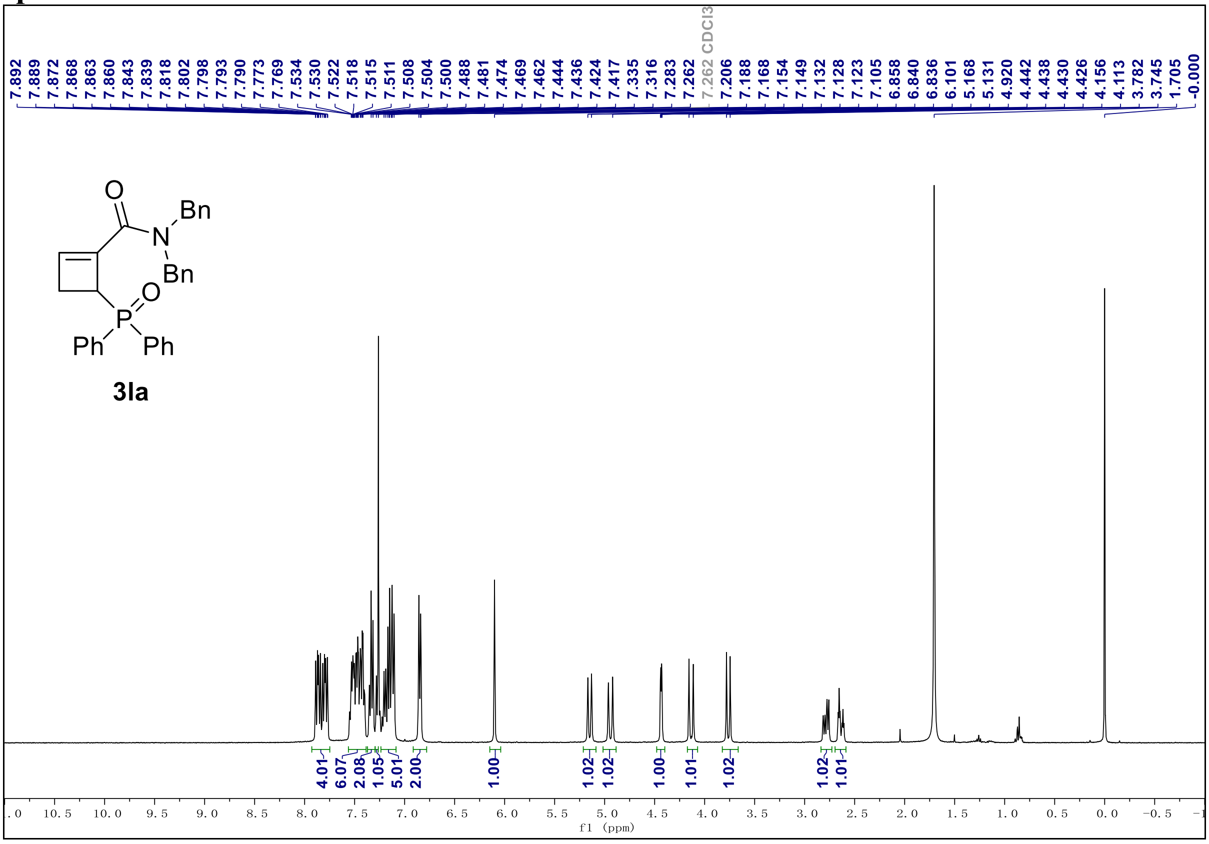

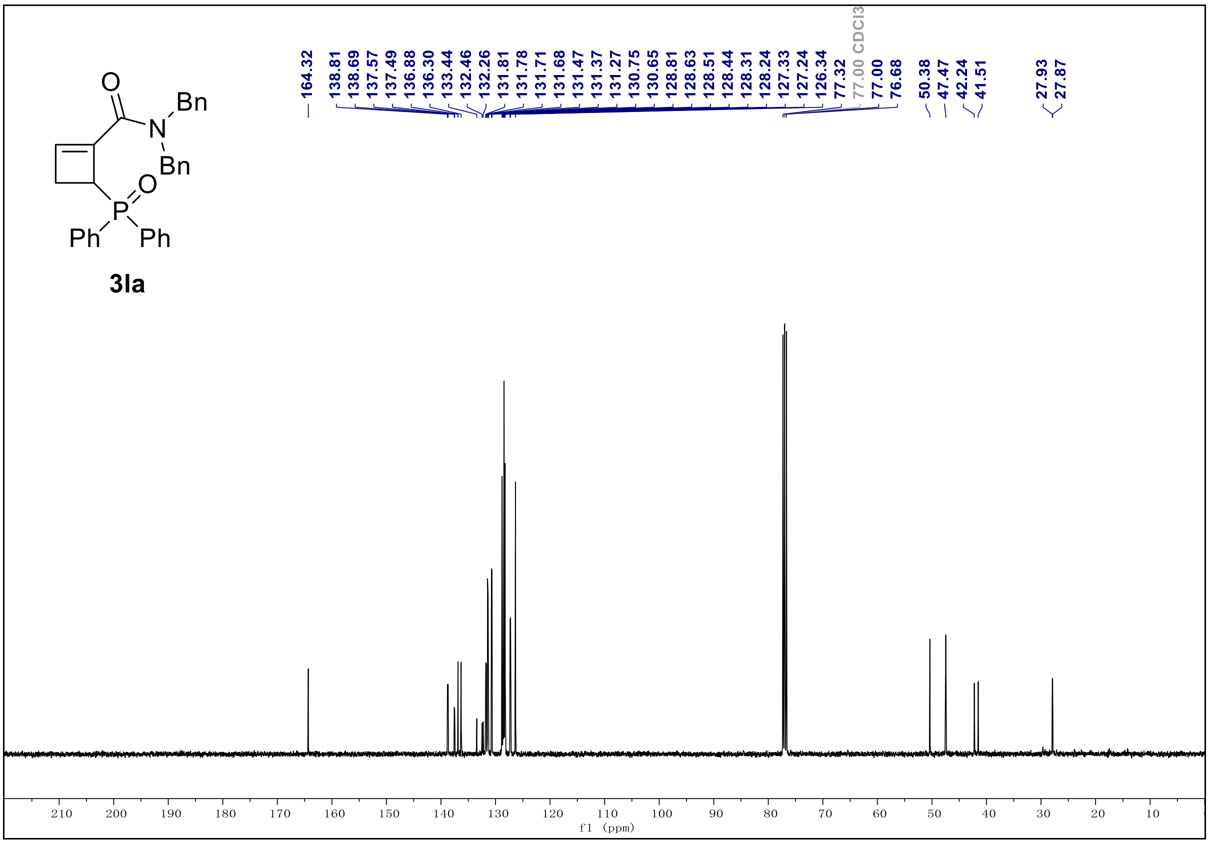

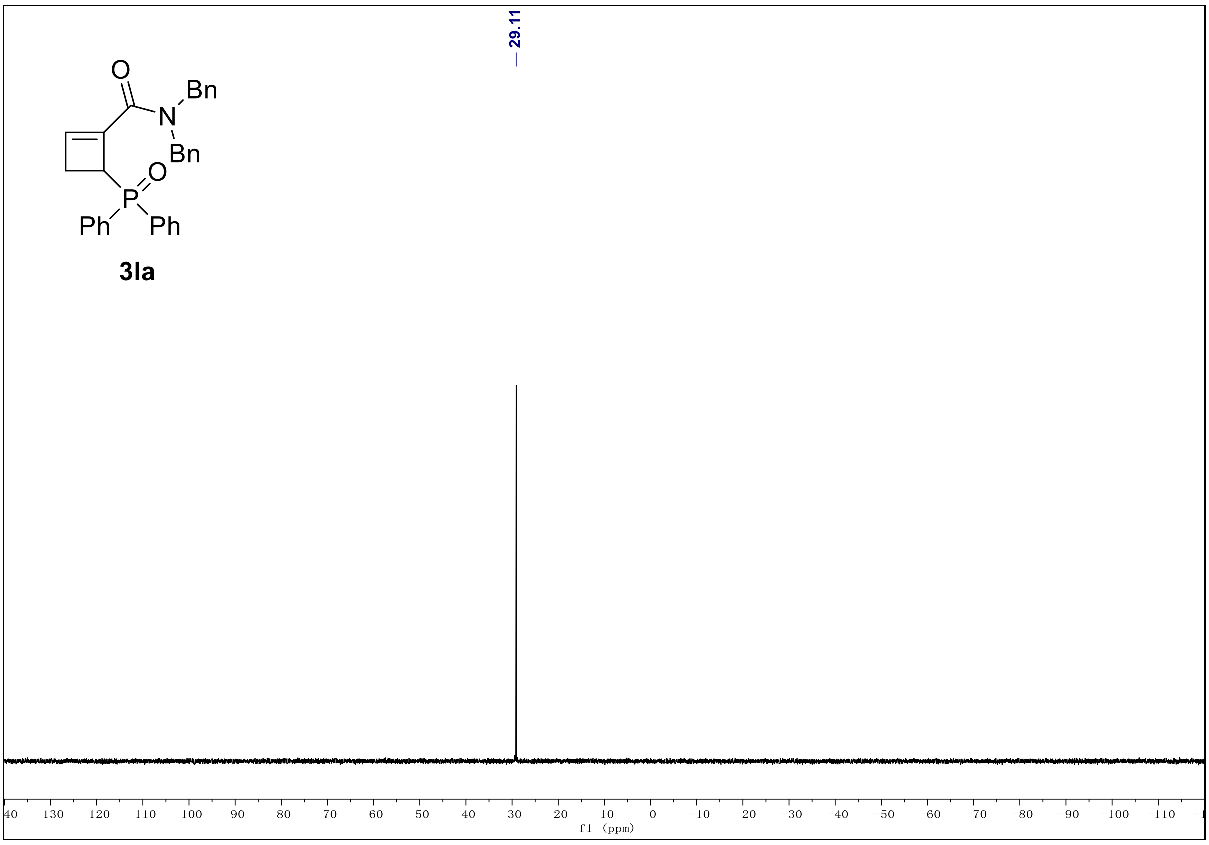
29.11
O
N
Bn
Bn
P
O
Ph
Ph
3la
f1 (ppm)

^{1}H NMR (400 MHz, CDCl$_3$), ^{13}C NMR (100 MHz, CDCl$_3$) and ^{31}P NMR (162 MHz, CDCl$_3$) spectrum of 3ma.

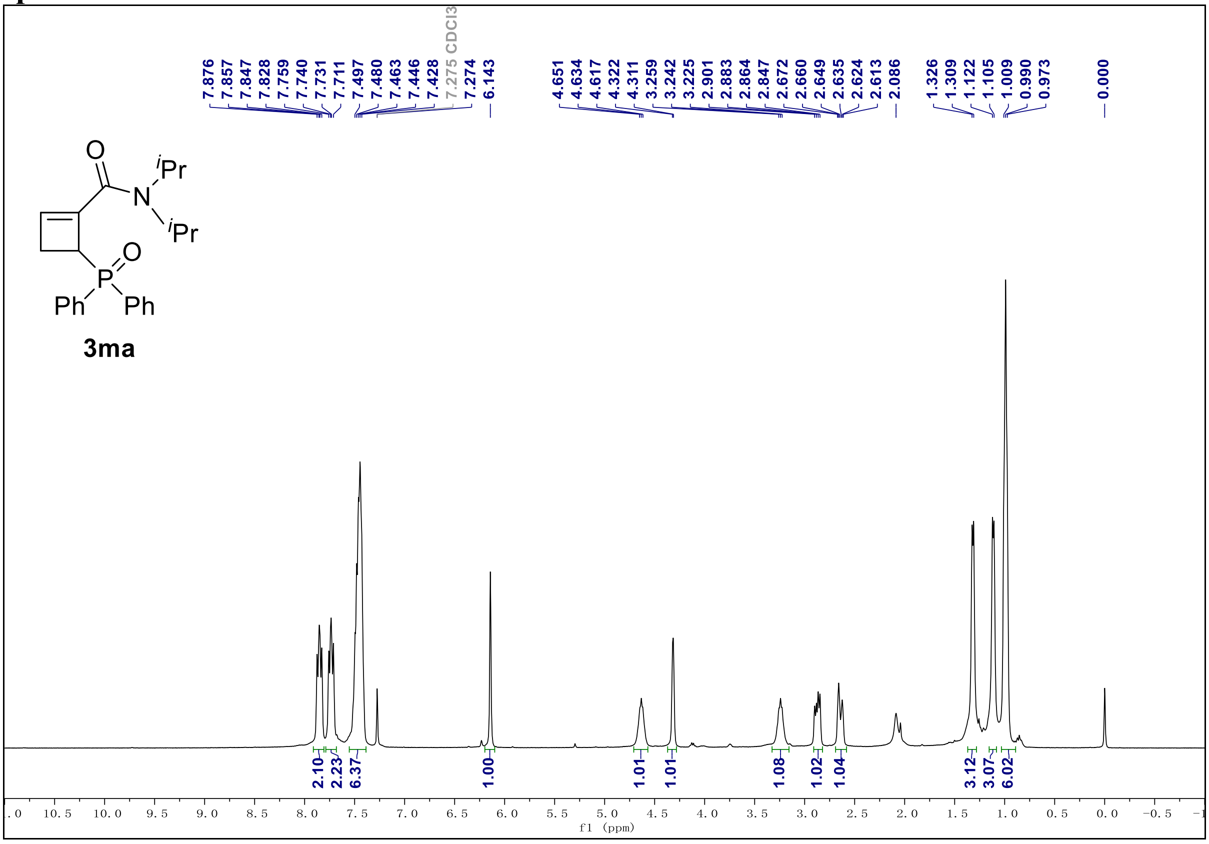

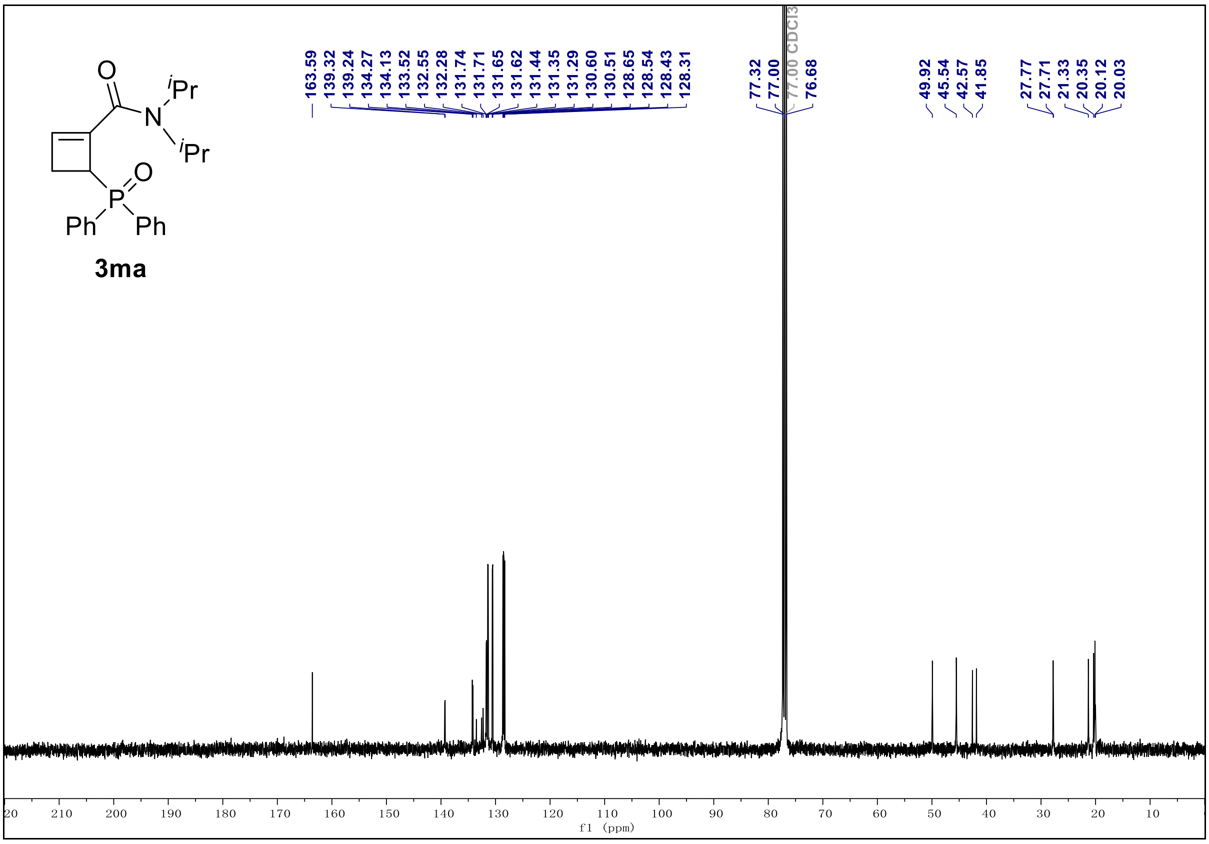

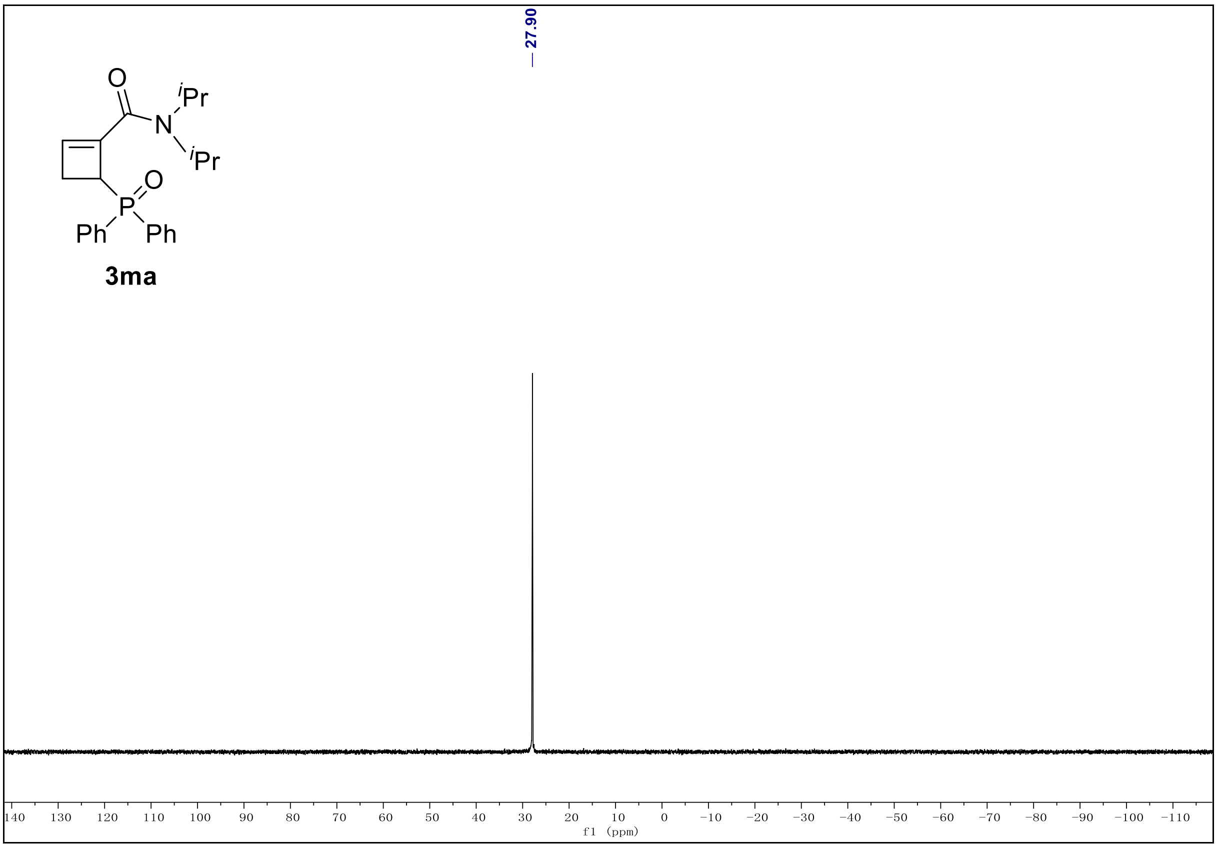
27.90
3ma
f1 (ppm)

^{1}H NMR (400 MHz, $CDCl_3$), ^{13}C NMR (100 MHz, $CDCl_3$) and ^{31}P NMR (162 MHz, $CDCl_3$) spectrum of 3na.

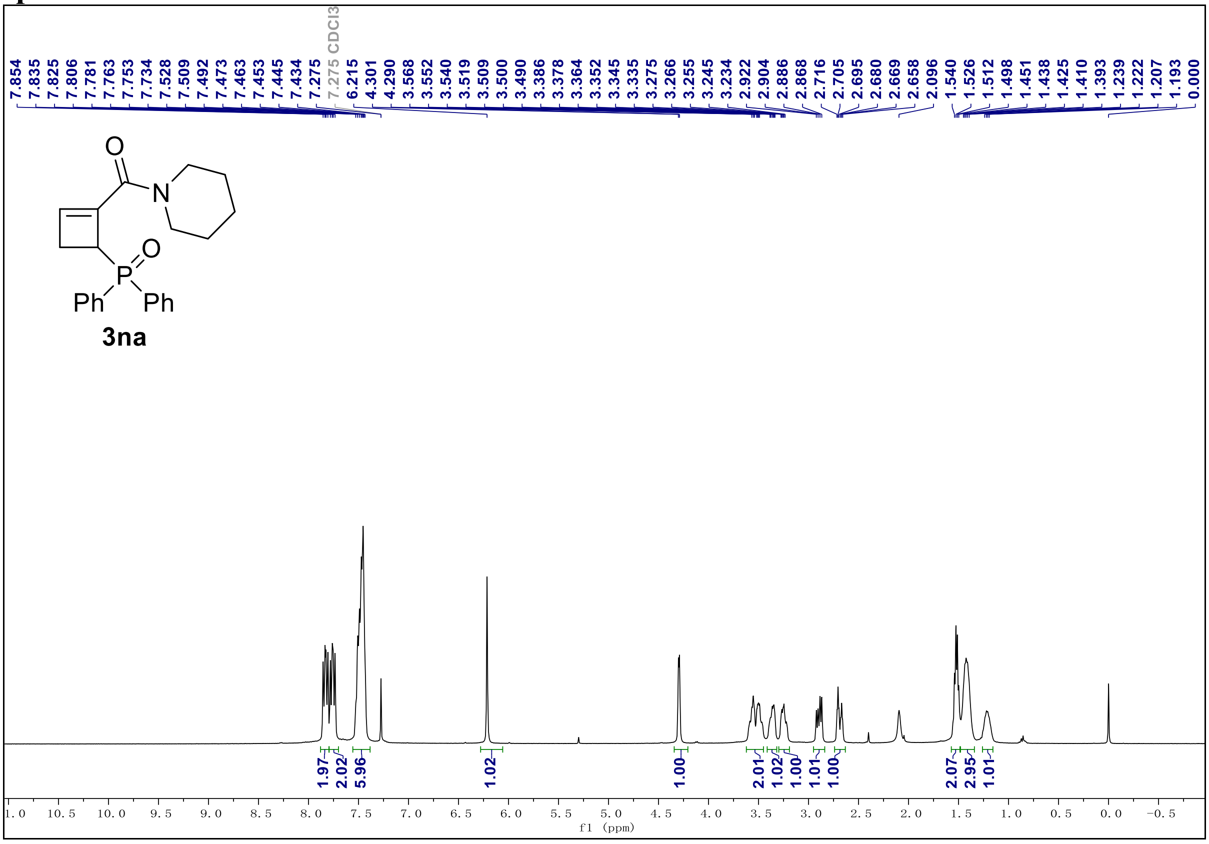

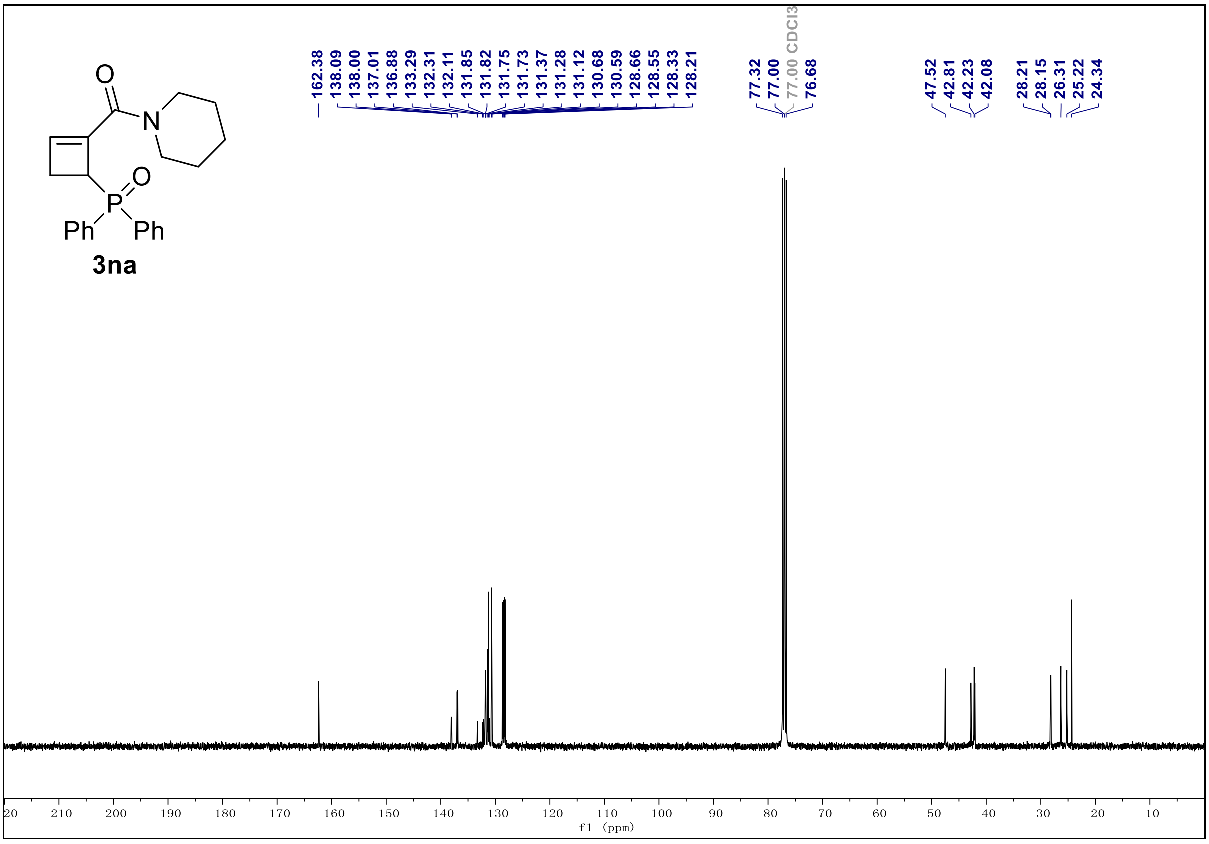

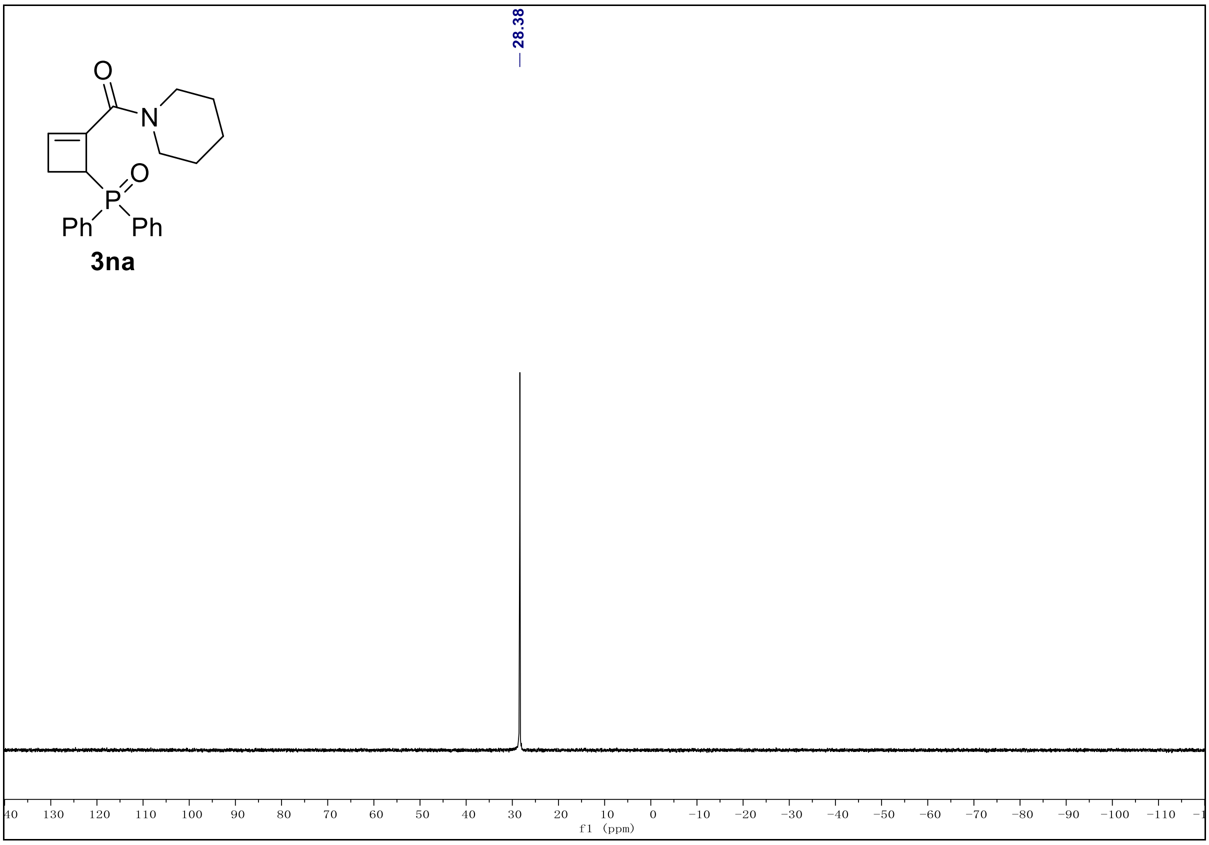
28.38
3na
f1 (ppm)

^{1}H NMR (400 MHz, $CDCl_3$), ^{13}C NMR (100 MHz, $CDCl_3$) and ^{31}P NMR (162 MHz, $CDCl_3$) spectrum of 3oa.

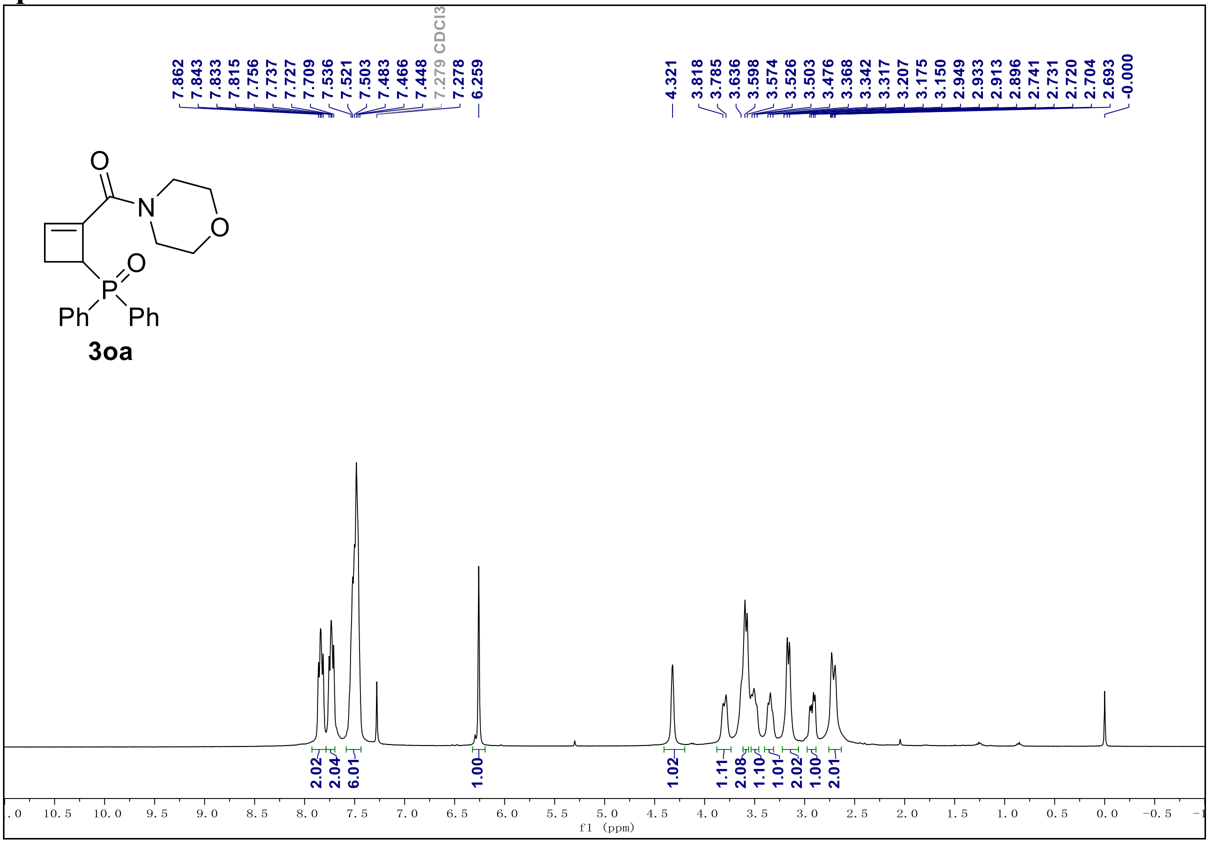

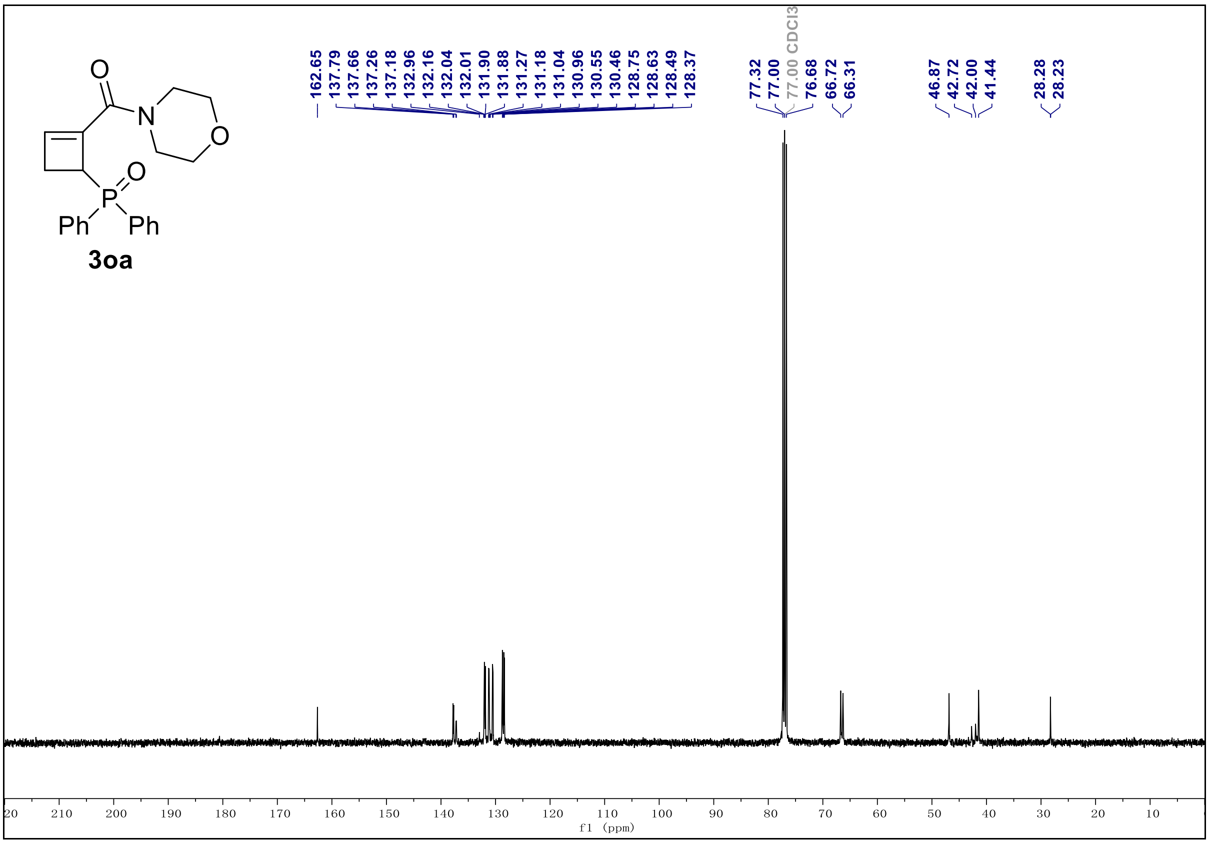

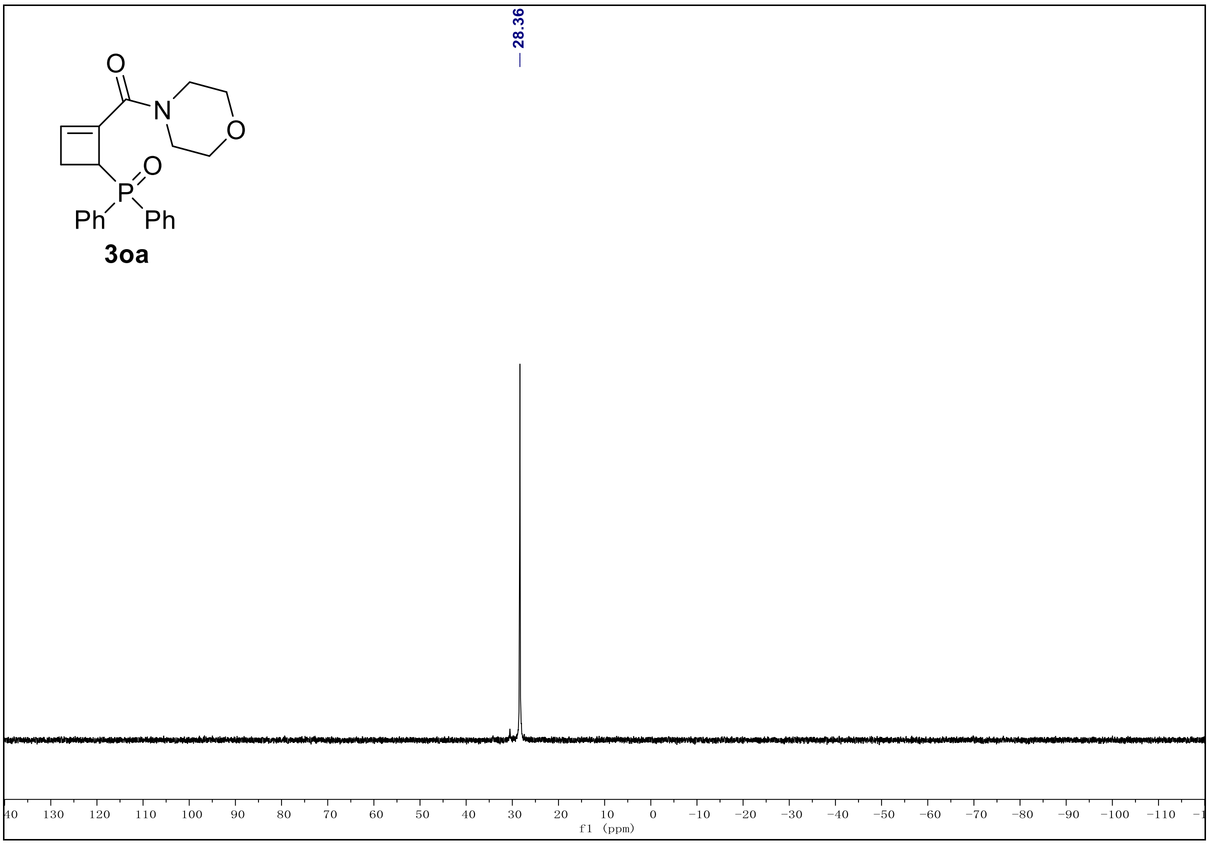
28.36
Ph
Ph
3oa
f1 (ppm)

^{1}H NMR (400 MHz, CDCl3), ^{13}C NMR (100 MHz, CDCl3) and ^{31}P NMR (162 MHz, CDCl3) spectrum of 3pa.

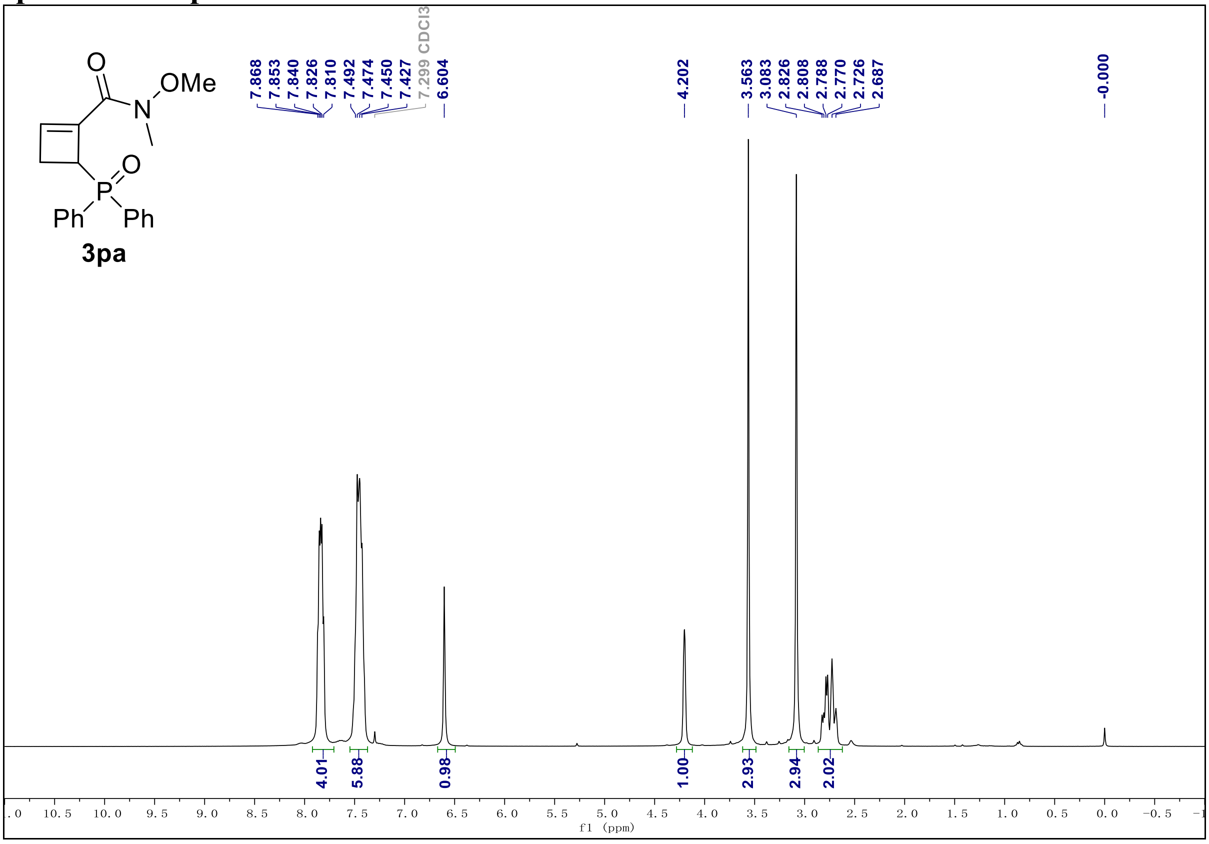

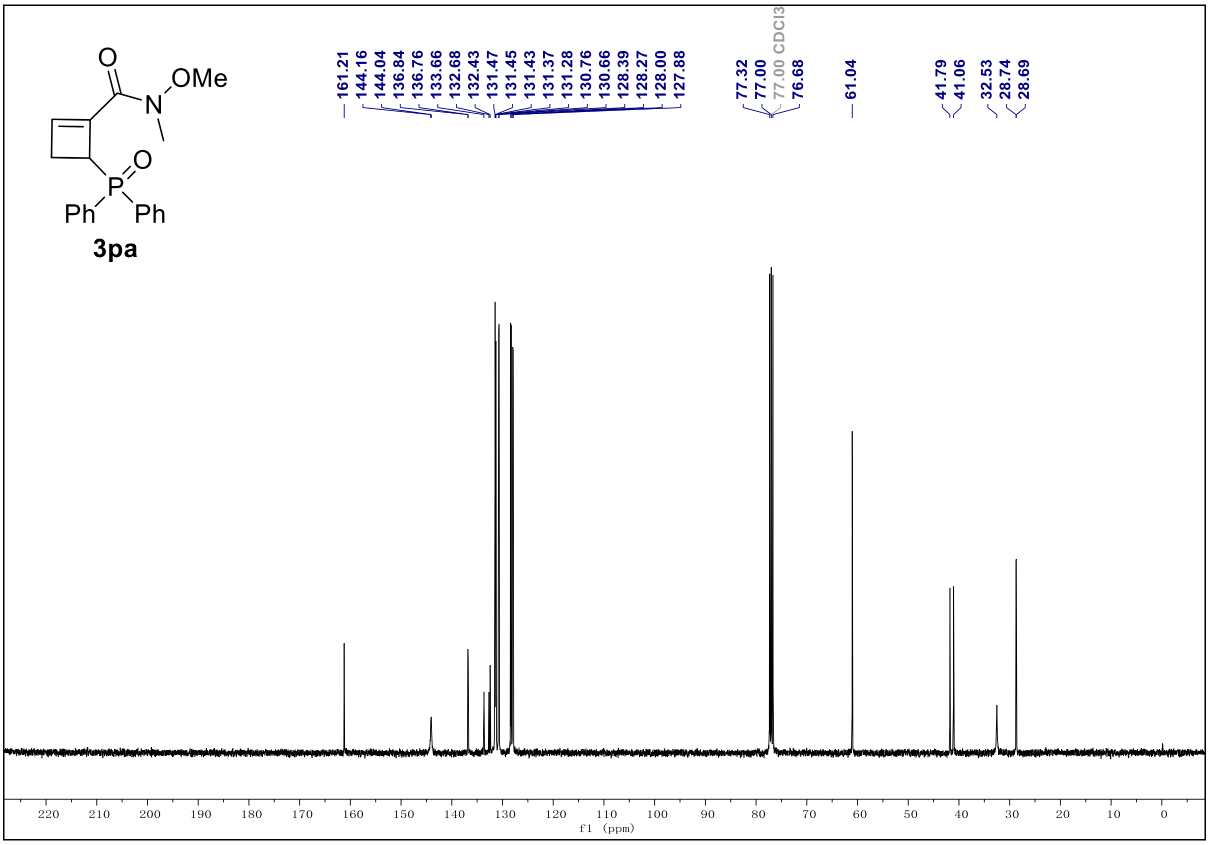

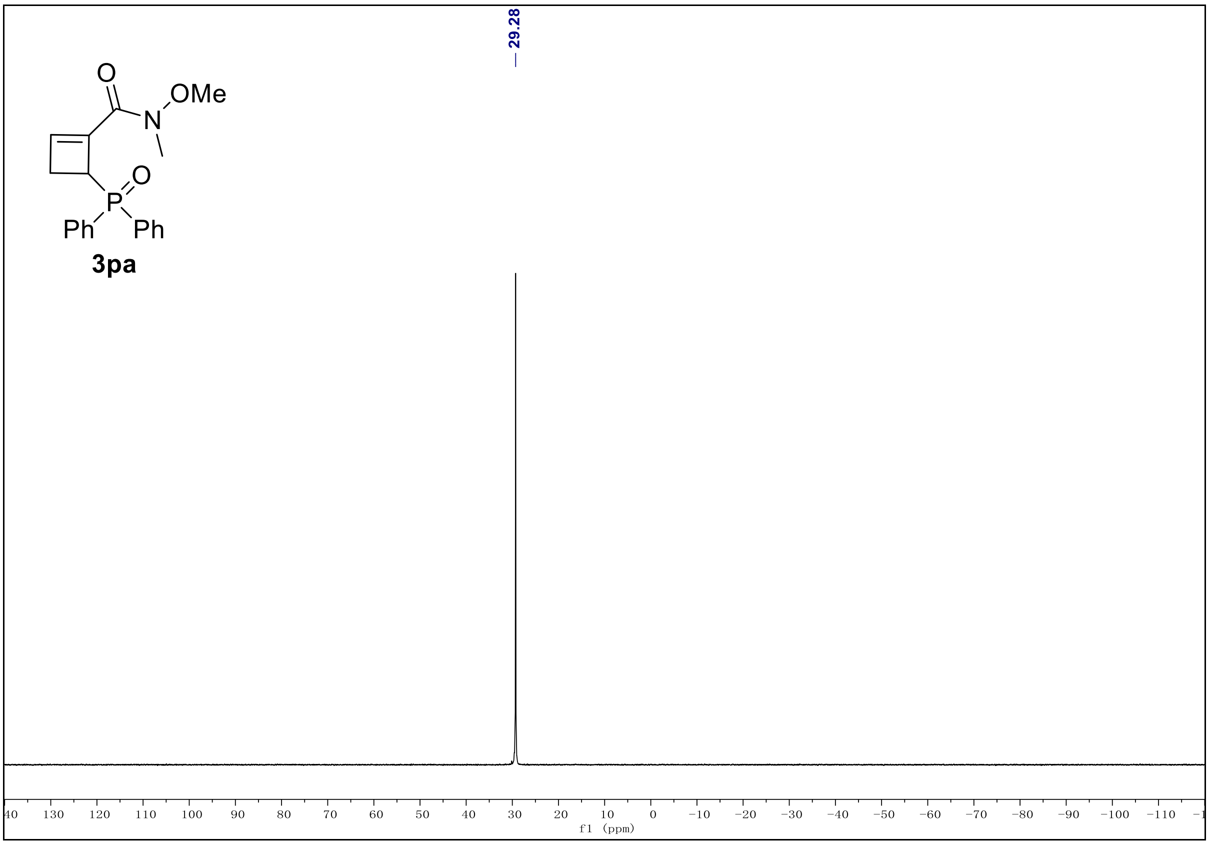

29.28
O
OMe
N
O
P
Ph
Ph
3pa
130 120 110 100 90 80 70 60 50 40 30 20 10 0 −10 −20 −30 −40 −50 −60 −70 −80 −90 −100 −110
f1 (ppm)

^{1}H NMR (400 MHz, $CDCl_3$), ^{13}C NMR (100 MHz, $CDCl_3$) and ^{31}P NMR (162 MHz, $CDCl_3$) spectrum of 3qa.

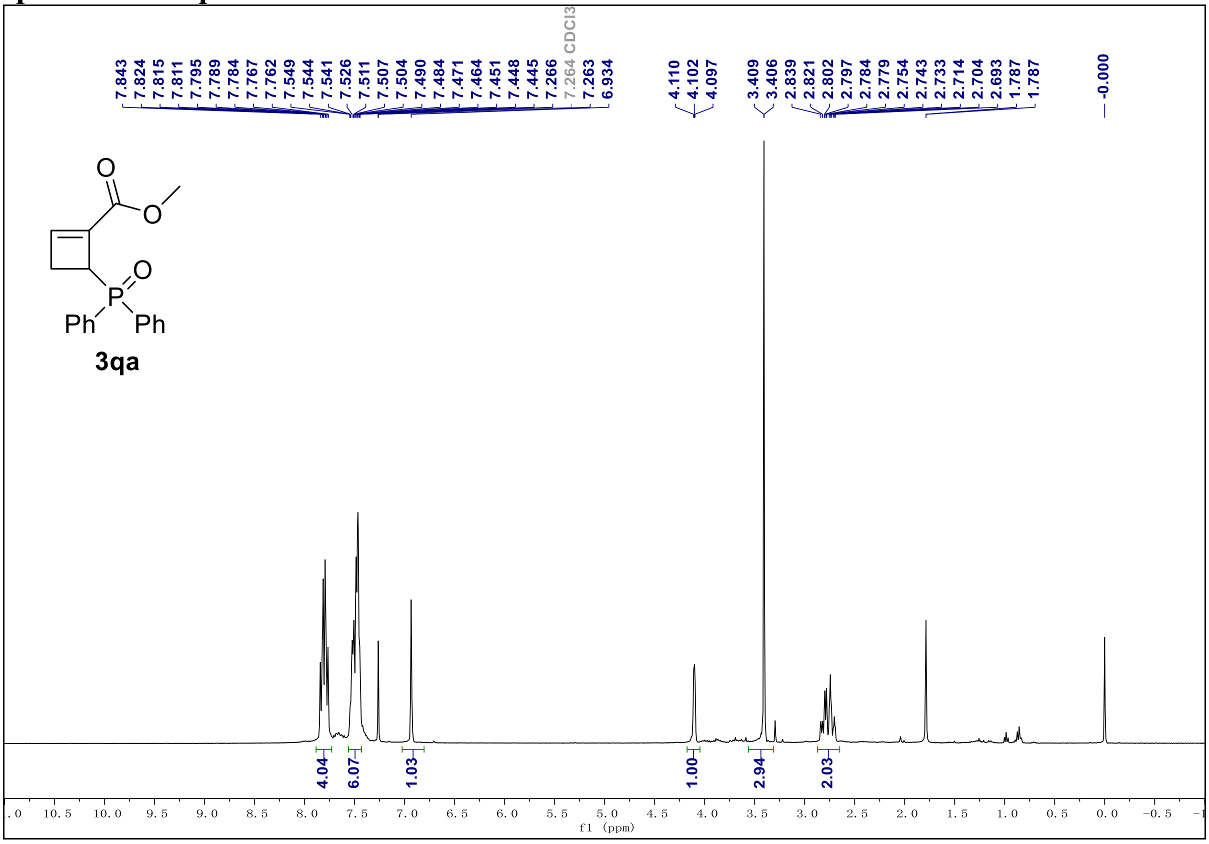

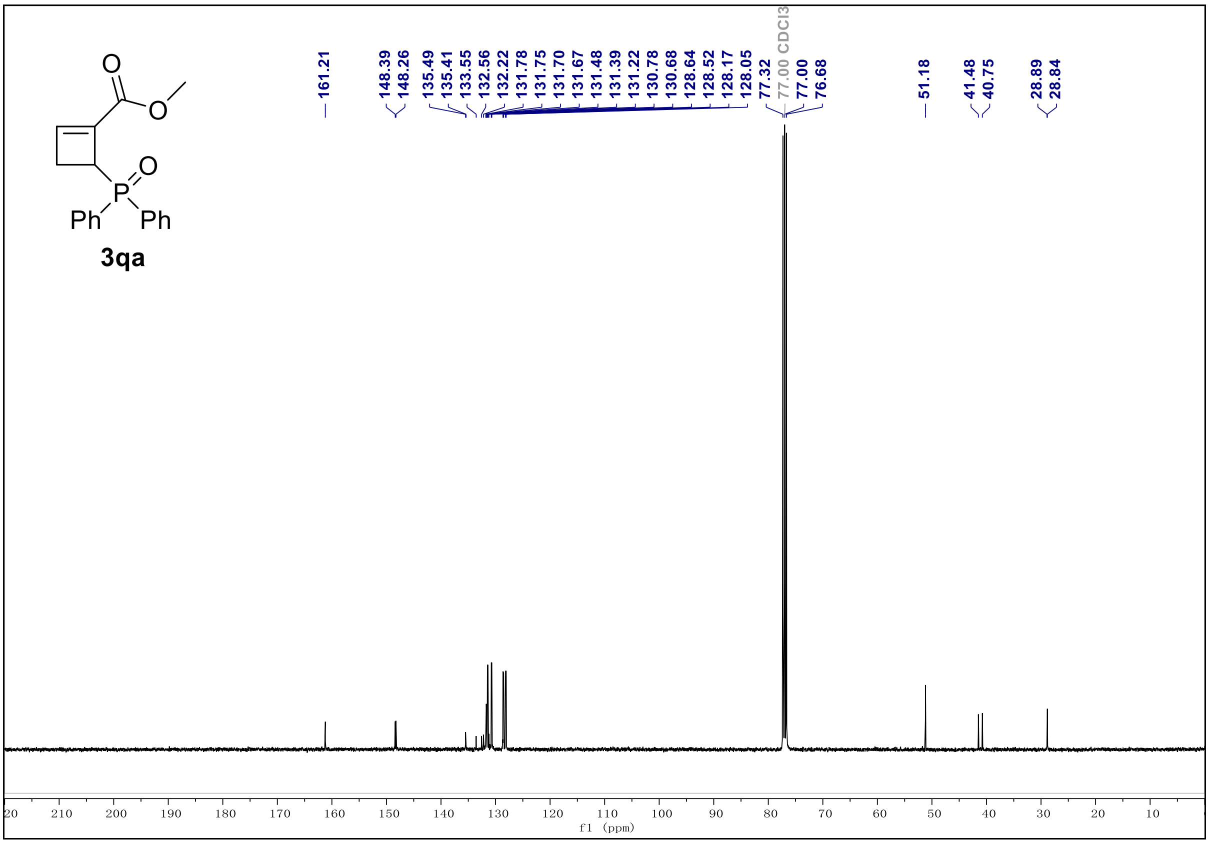

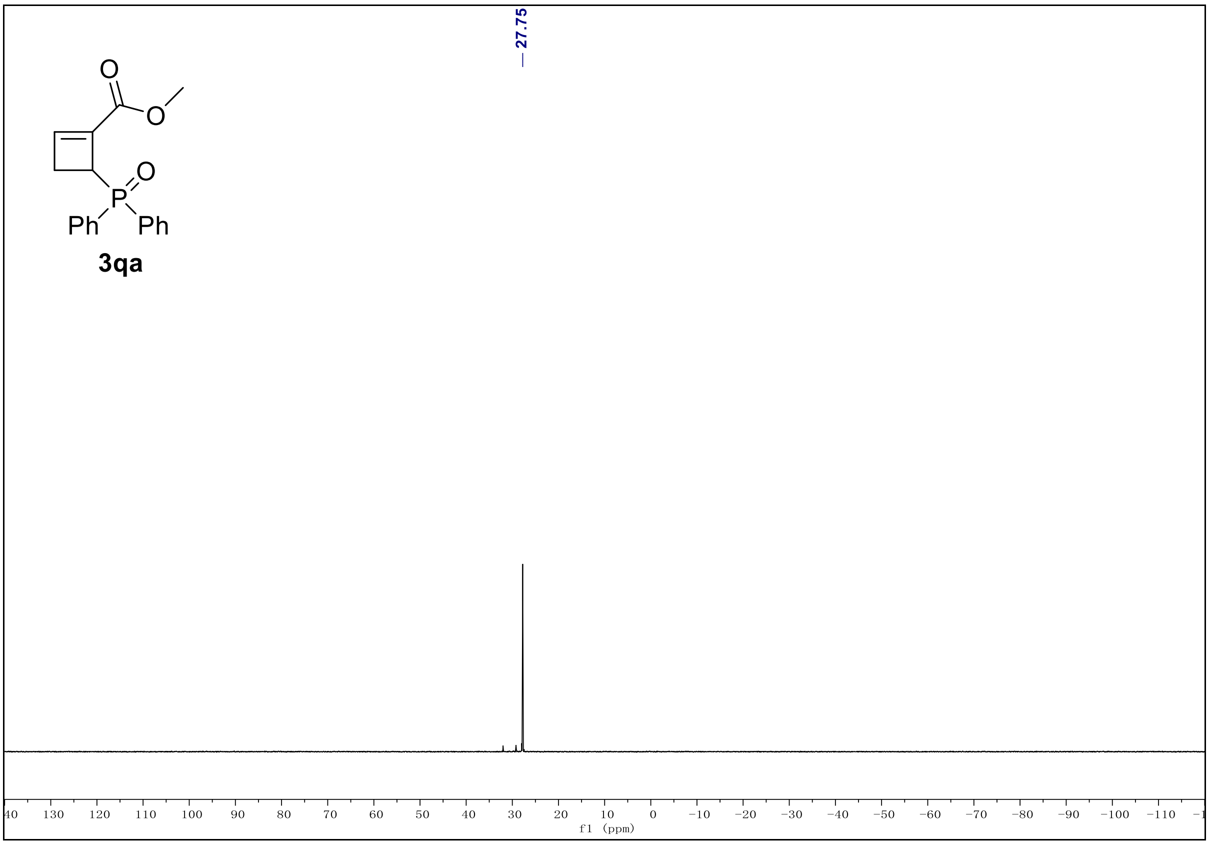
27.75
O
O
O
P
Ph
Ph
3qa
f1 (ppm)

^{1}H NMR (400 MHz, CDCl$_3$), ^{13}C NMR (100 MHz, CDCl$_3$) and ^{31}P NMR (162 MHz, CDCl$_3$) spectrum of 3ra.

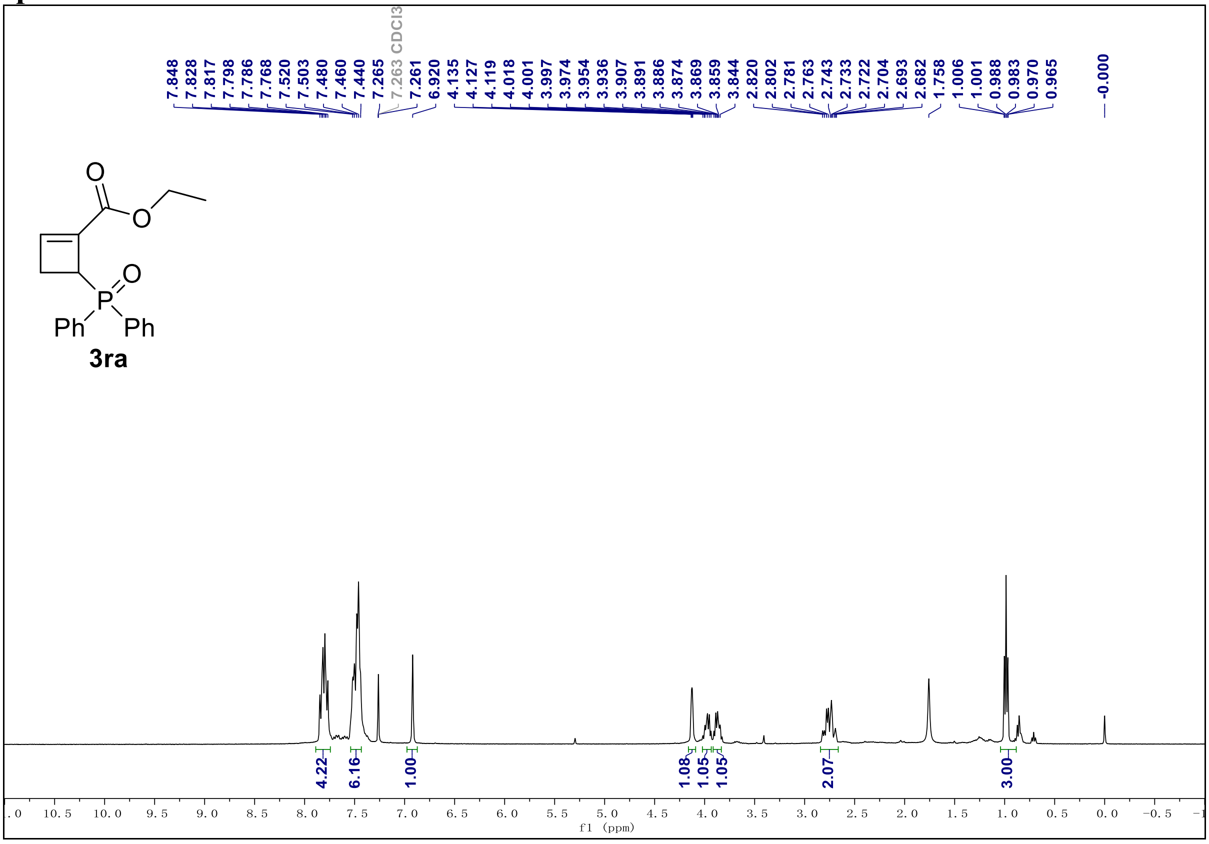

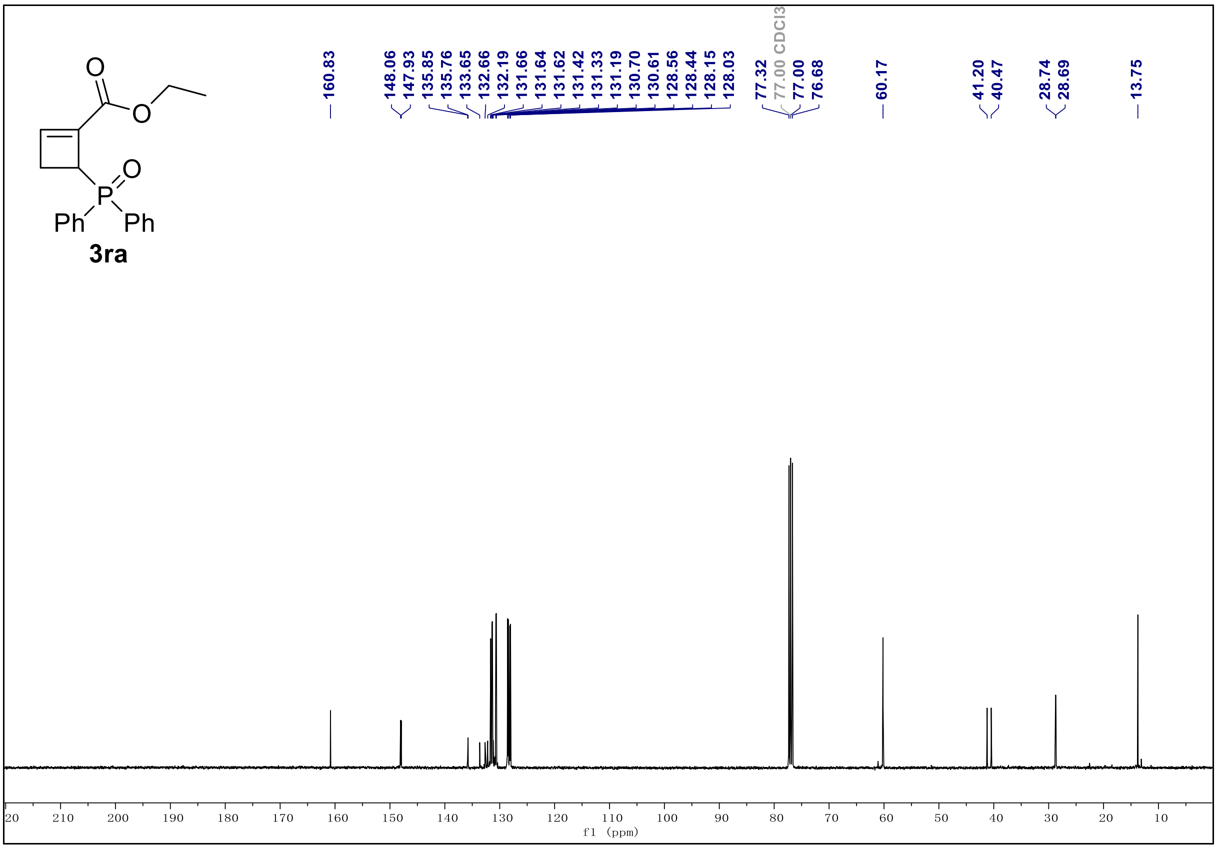

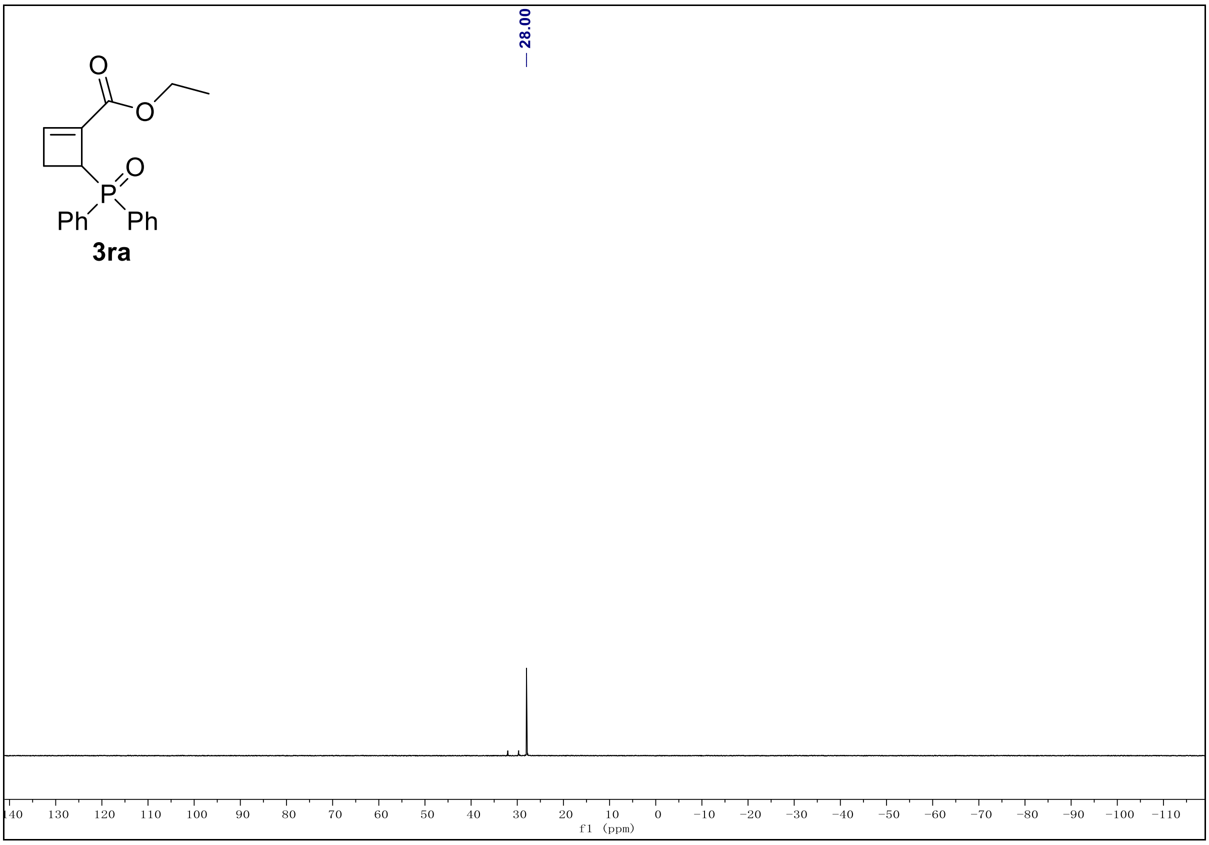

28.00
3ra
140
130
120
110
100
90
80
70
60
50
40
30
20
10
0
−10
−20
−30
−40
−50
−60
−70
−80
−90
−100
−110
f1 (ppm)

^{1}H NMR (400 MHz, $CDCl_3$), ^{13}C NMR (100 MHz, $CDCl_3$) and ^{31}P NMR (162 MHz, $CDCl_3$) spectrum of 3sa.

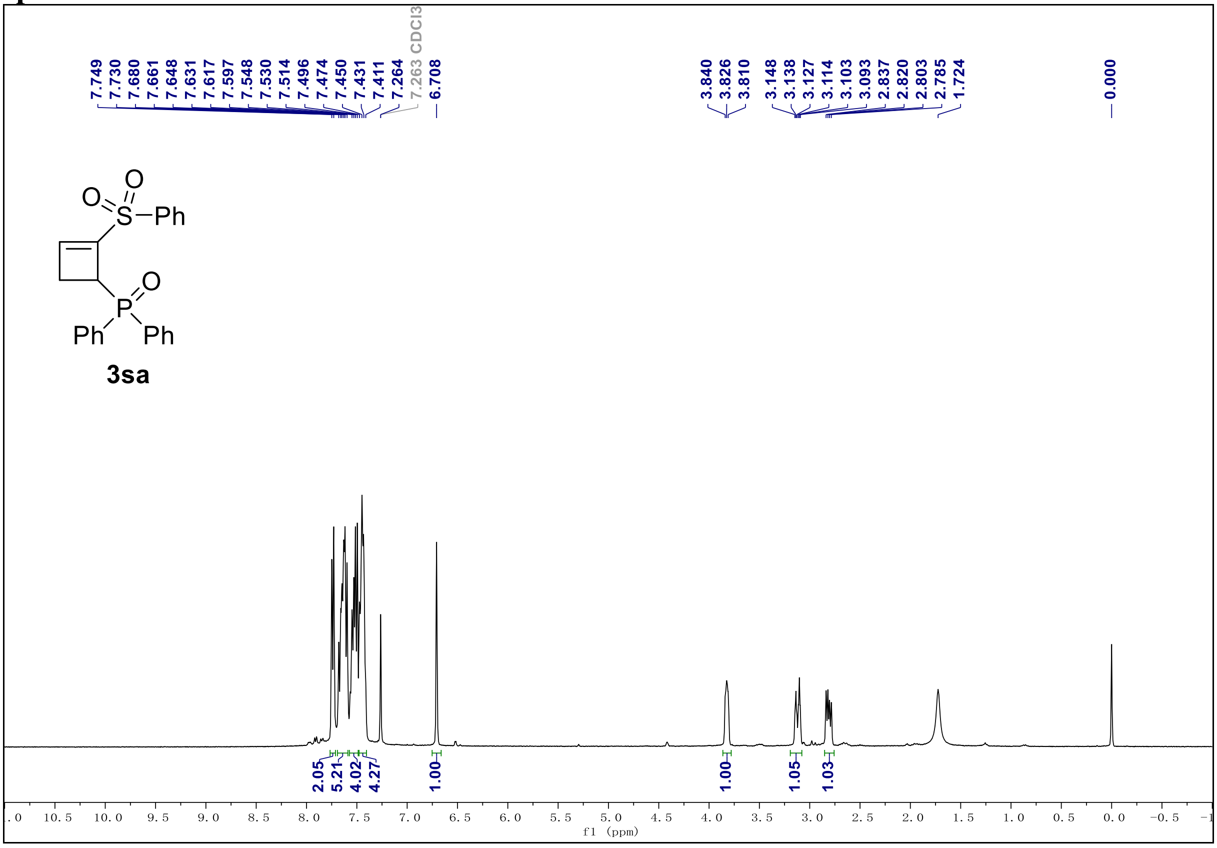

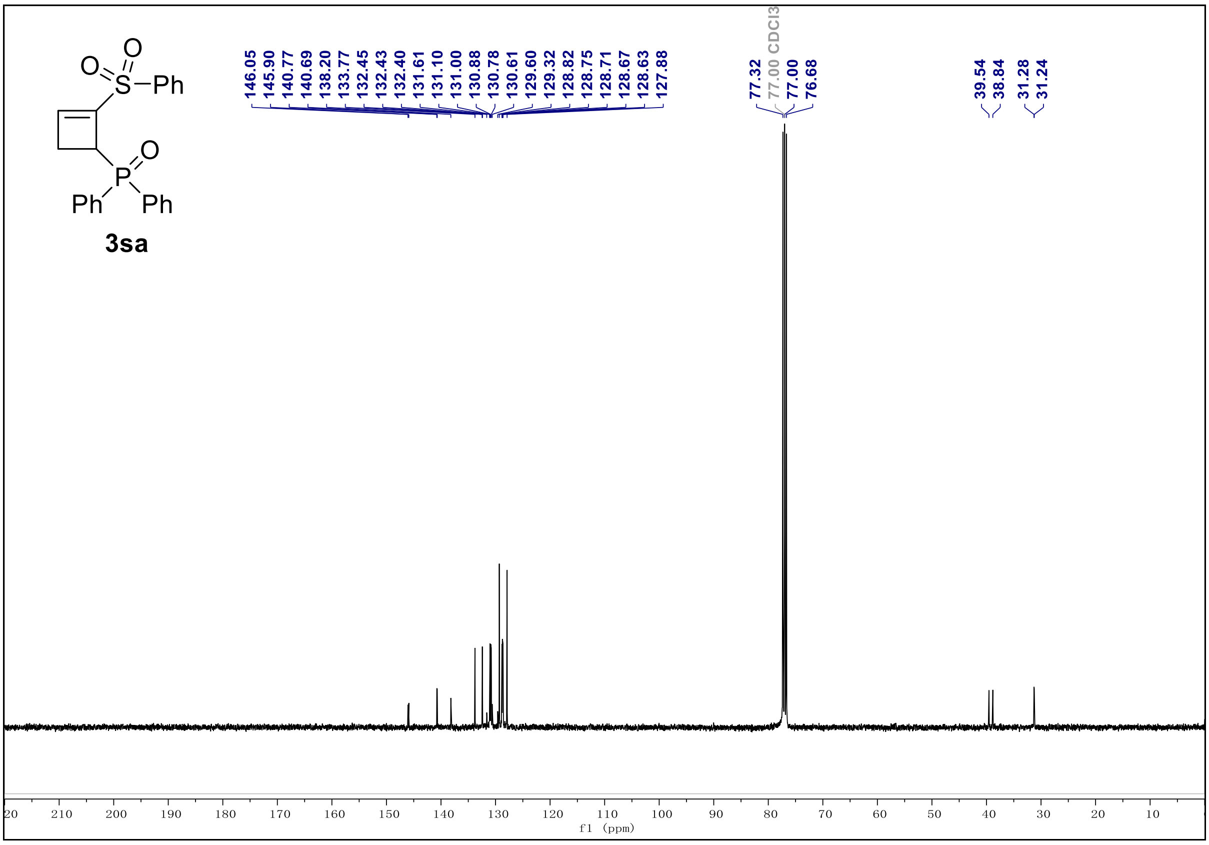

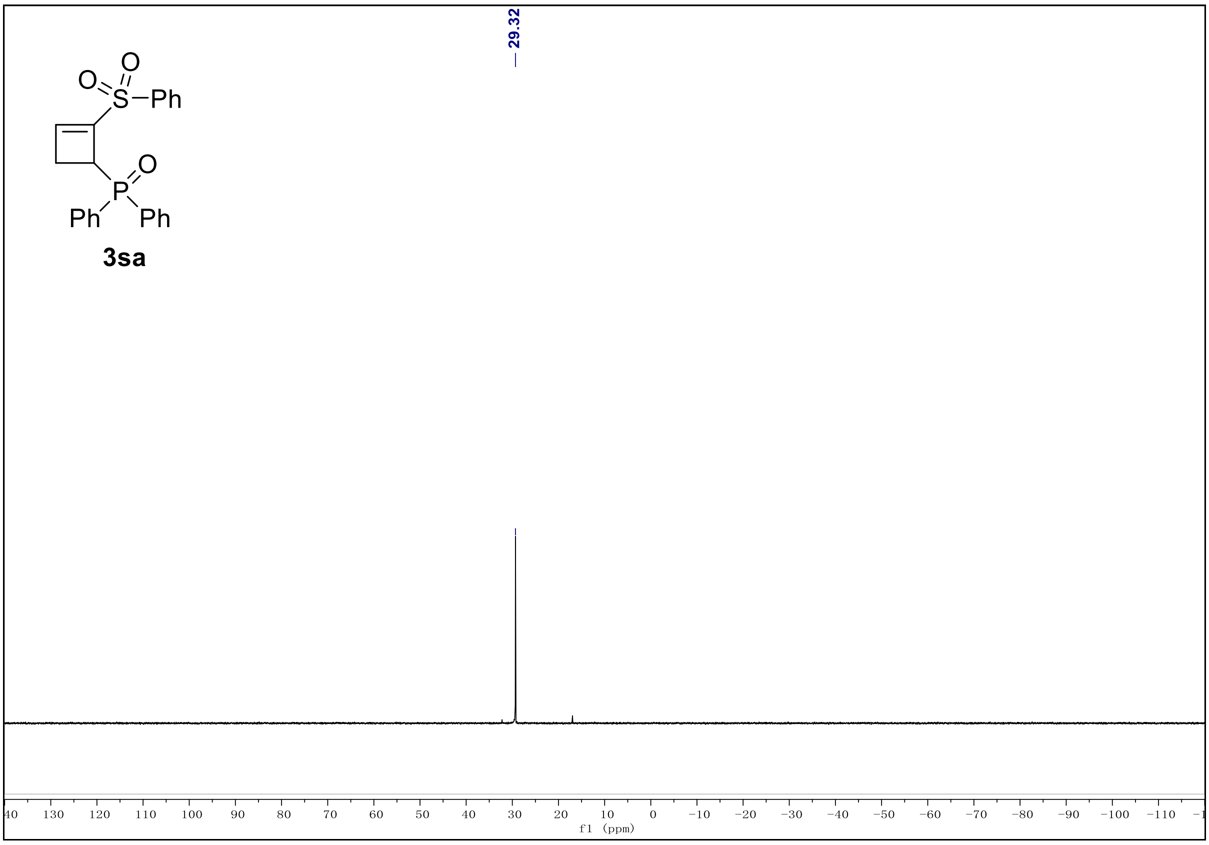
O
O
S
Ph
P
O
Ph
Ph
3sa
29.32
f1 (ppm)

^{1}H NMR (400 MHz, $CDCl_3$), ^{13}C NMR (100 MHz, $CDCl_3$) and ^{31}P NMR (162 MHz, $CDCl_3$) spectrum of 3ab.

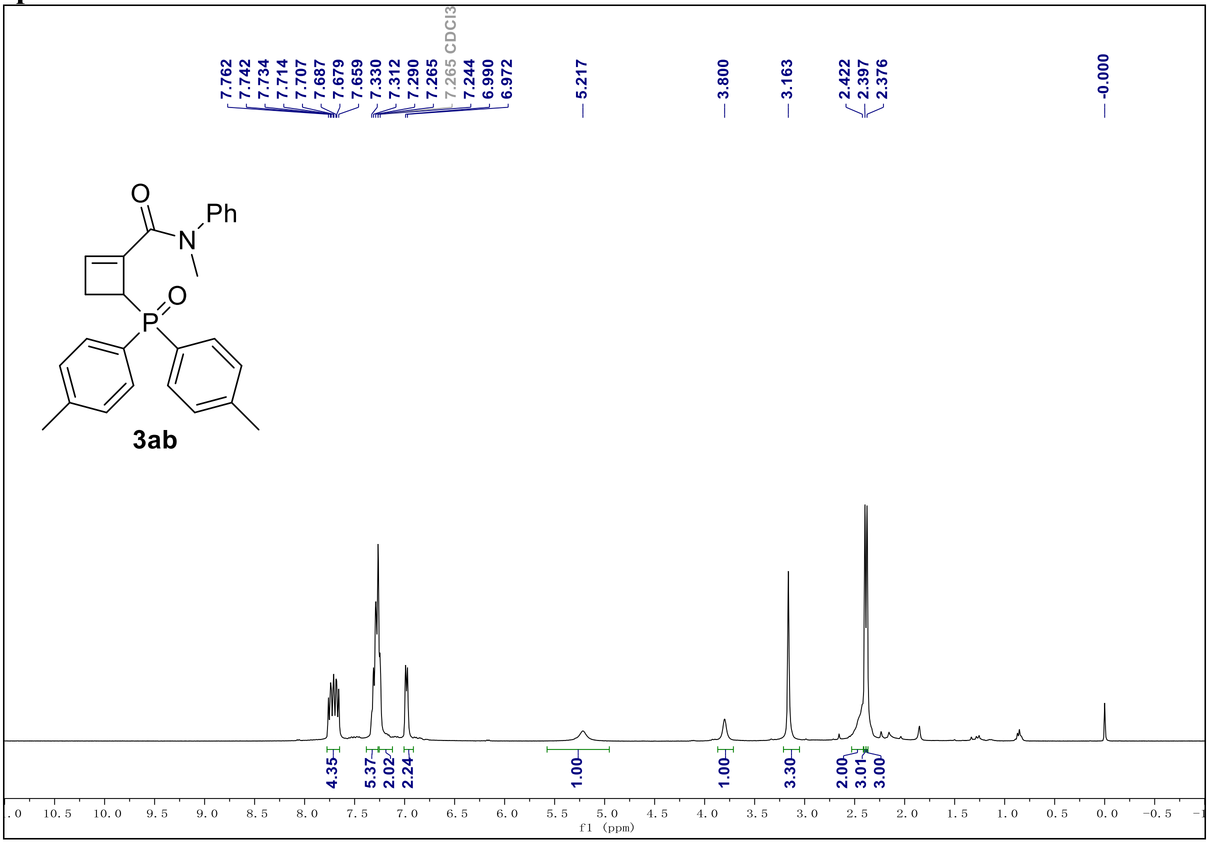

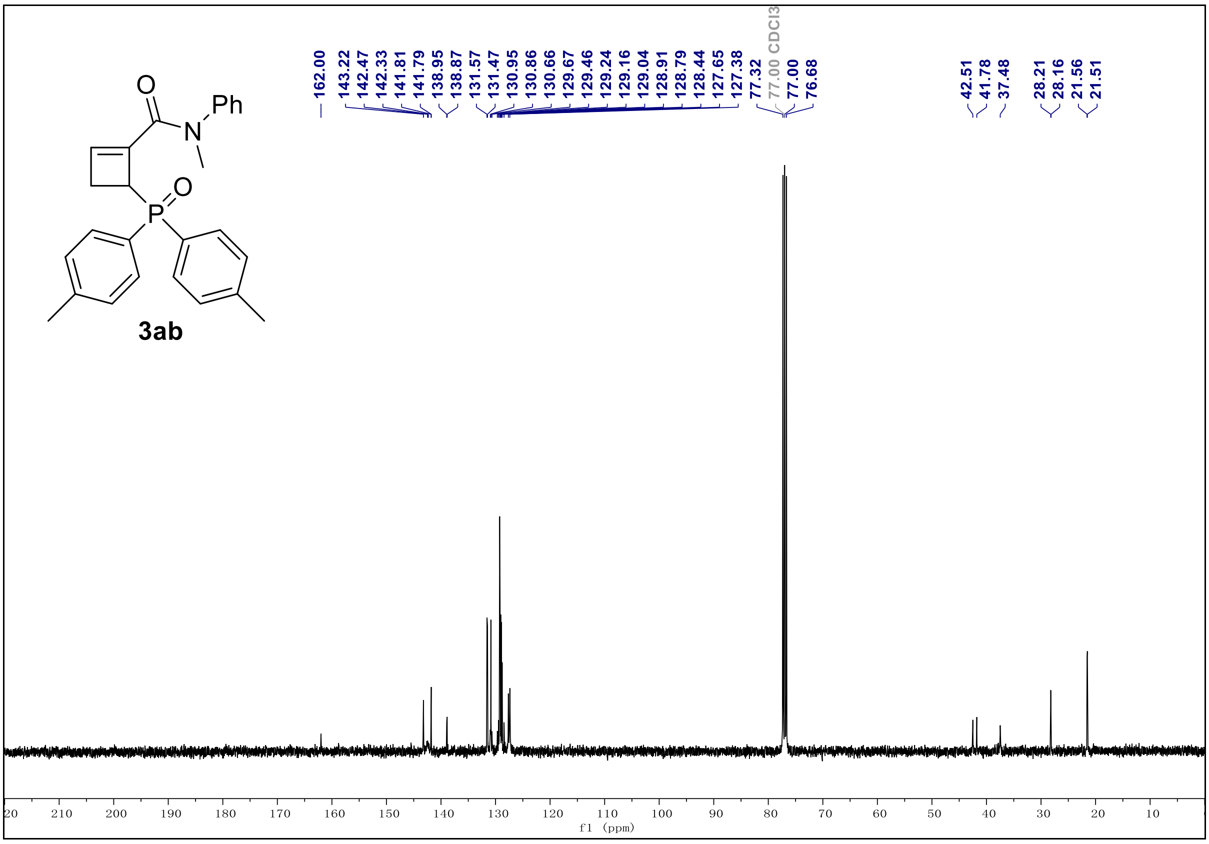

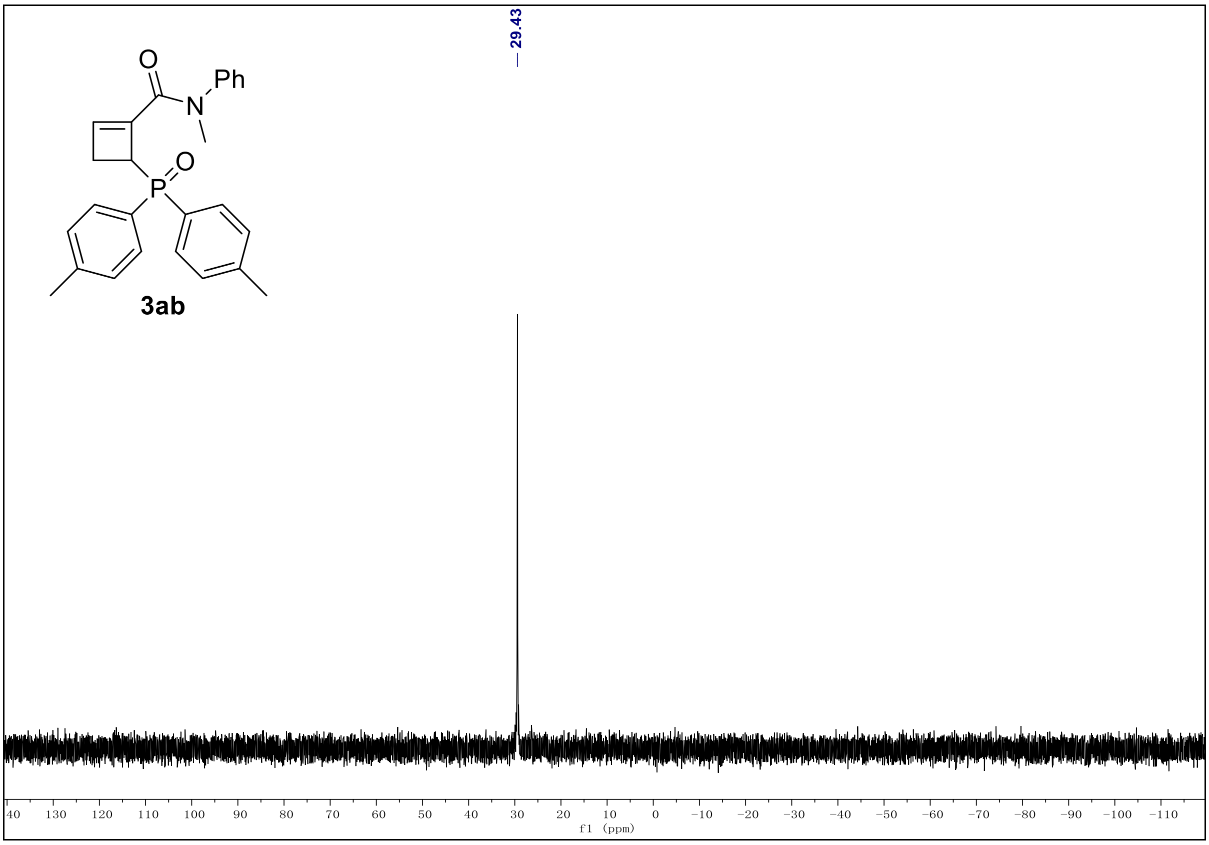

29.43
O
Ph
N
P
O
3ab
40
130
120
110
100
90
80
70
60
50
40
30
20
10
0
−10
−20
−30
−40
−50
−60
−70
−80
−90
−100
−110
f1 (ppm)

^{1}H NMR (400 MHz, $CDCl_3$), ^{13}C NMR (100 MHz, $CDCl_3$) and ^{31}P NMR (162 MHz, $CDCl_3$) spectrum of 3ac.

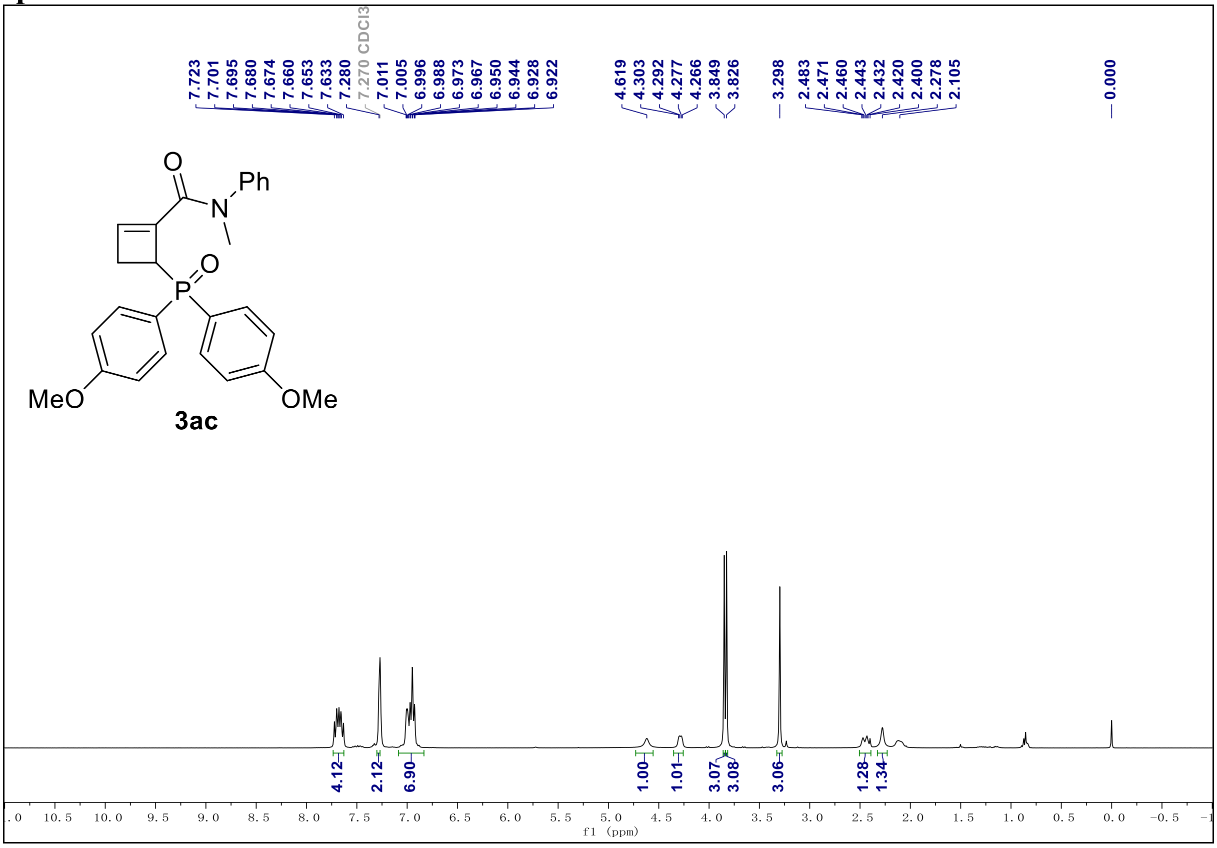

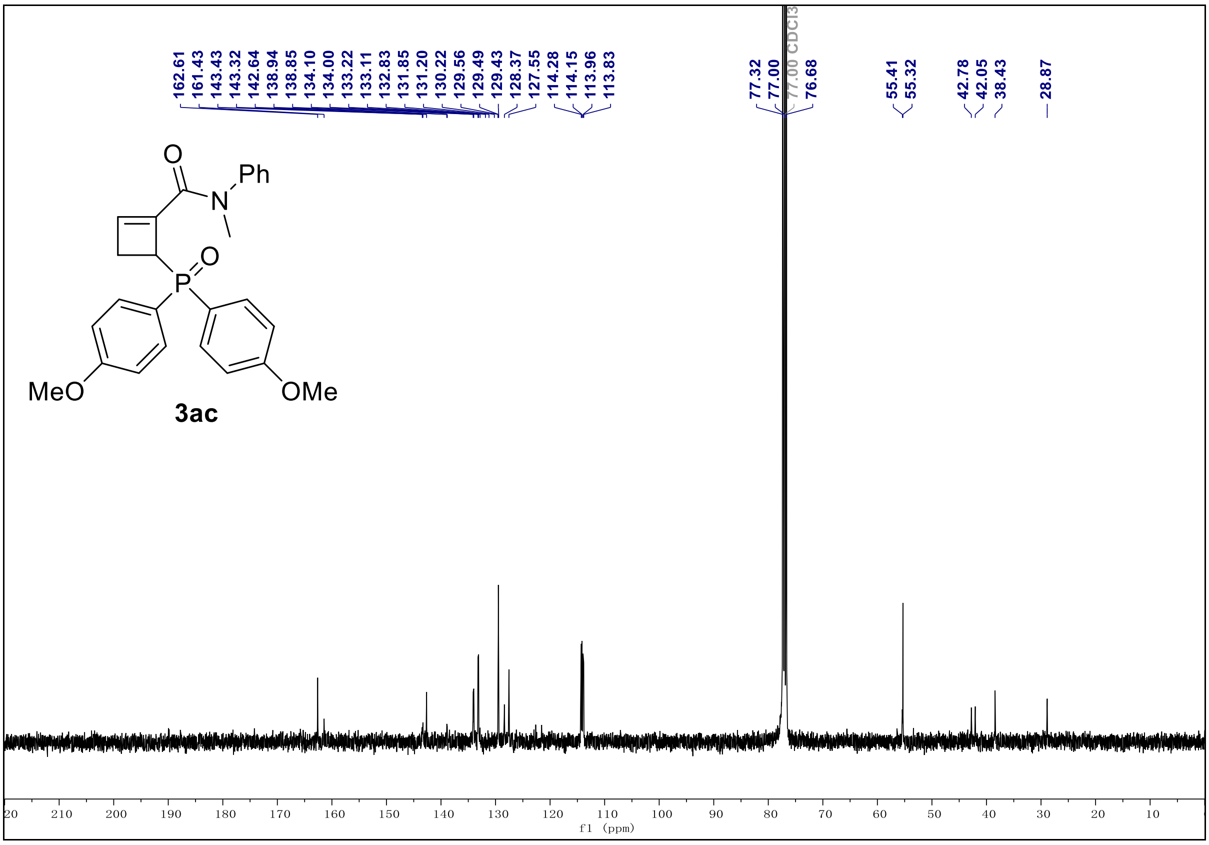

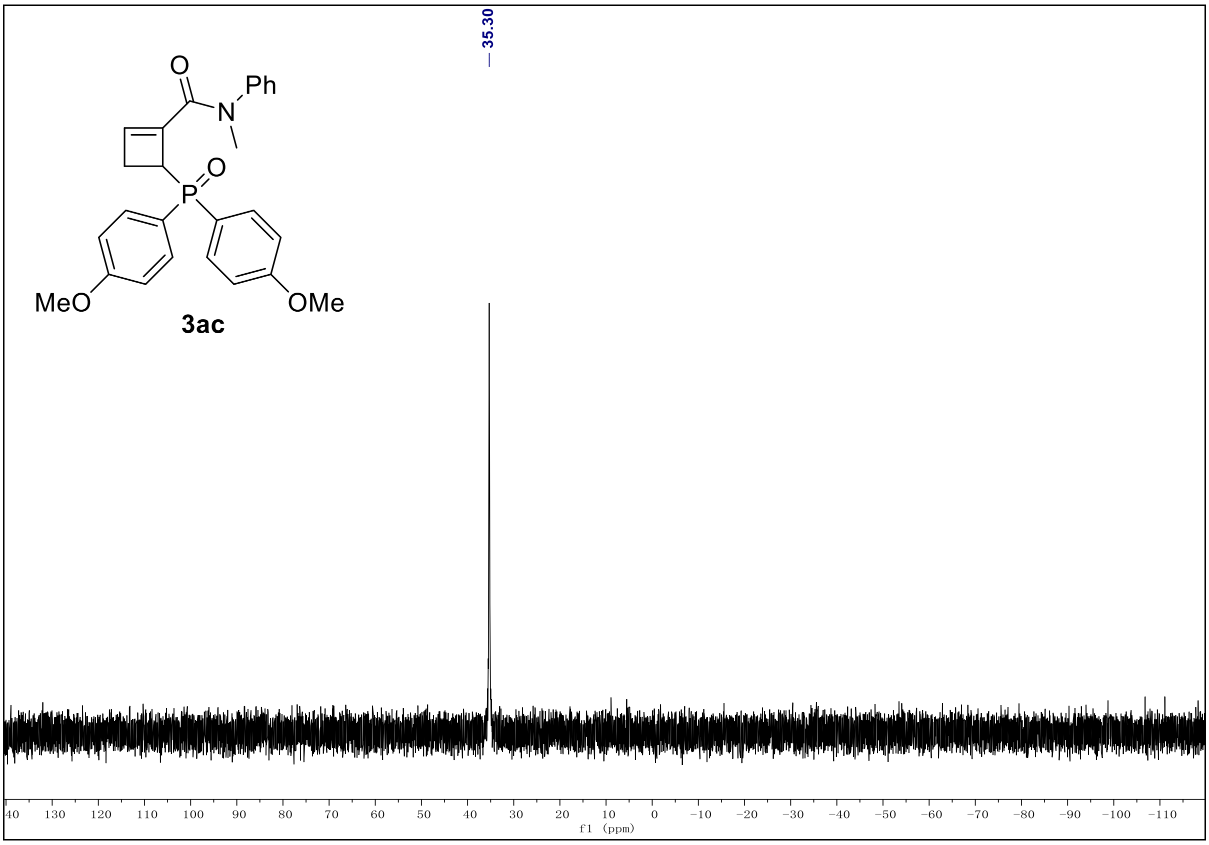

35.30
O
Ph
N
P
O
MeO
OMe
3ac
40
130
120
110
100
90
80
70
60
50
40
30
20
10
0
−10
−20
−30
−40
−50
−60
−70
−80
−90
−100
−110
f1 (ppm)

^{1}H NMR (400 MHz, CDCl$_3$), ^{13}C NMR (100 MHz, CDCl$_3$) and ^{31}P NMR (162 MHz, CDCl$_3$) spectrum of 3ad.

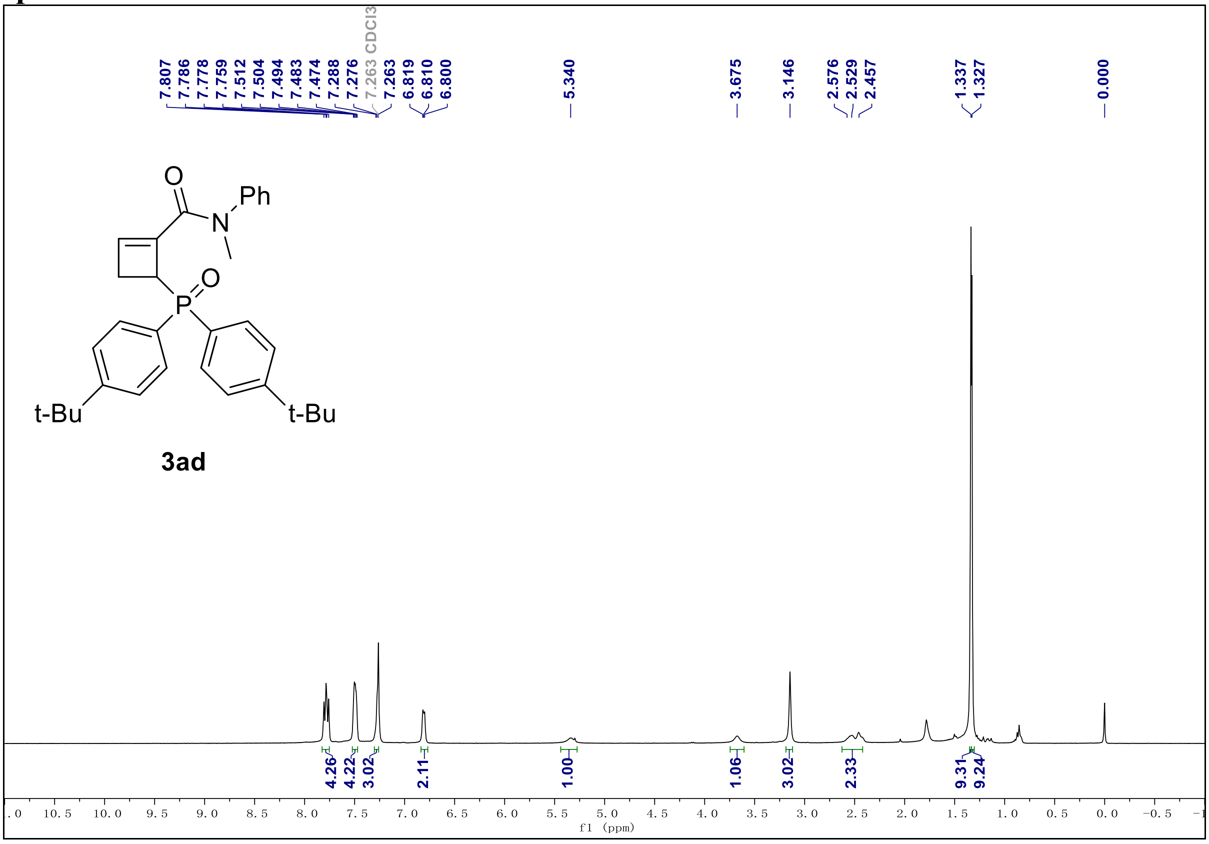

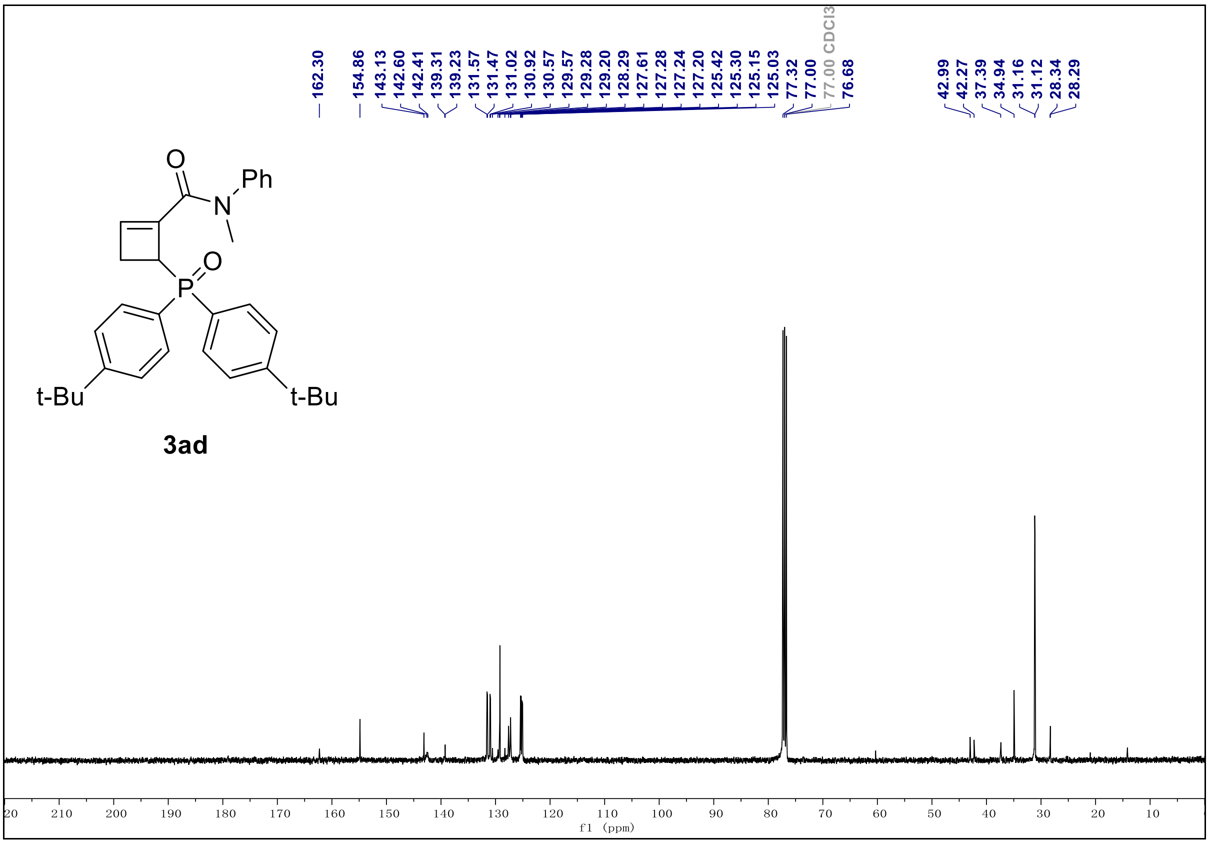

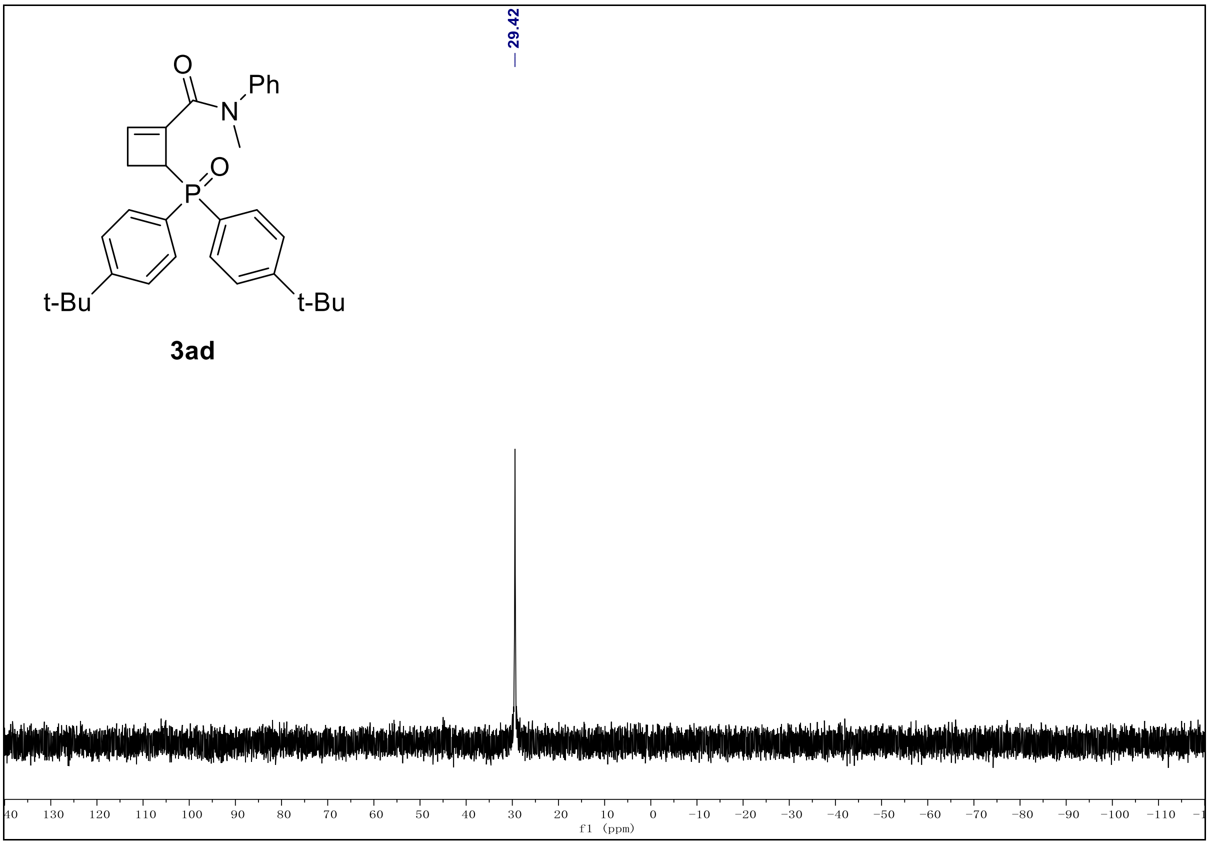

29.42
O
Ph
N
O
P
t-Bu
t-Bu
3ad
40 130 120 110 100 90 80 70 60 50 40 30 20 10 0 −10 −20 −30 −40 −50 −60 −70 −80 −90 −100 −110
f1 (ppm)

^{1}H NMR (400 MHz, $CDCl_3$), ^{13}C NMR (100 MHz, $CDCl_3$) and ^{31}P NMR (162 MHz, $CDCl_3$) spectrum of 3ae.

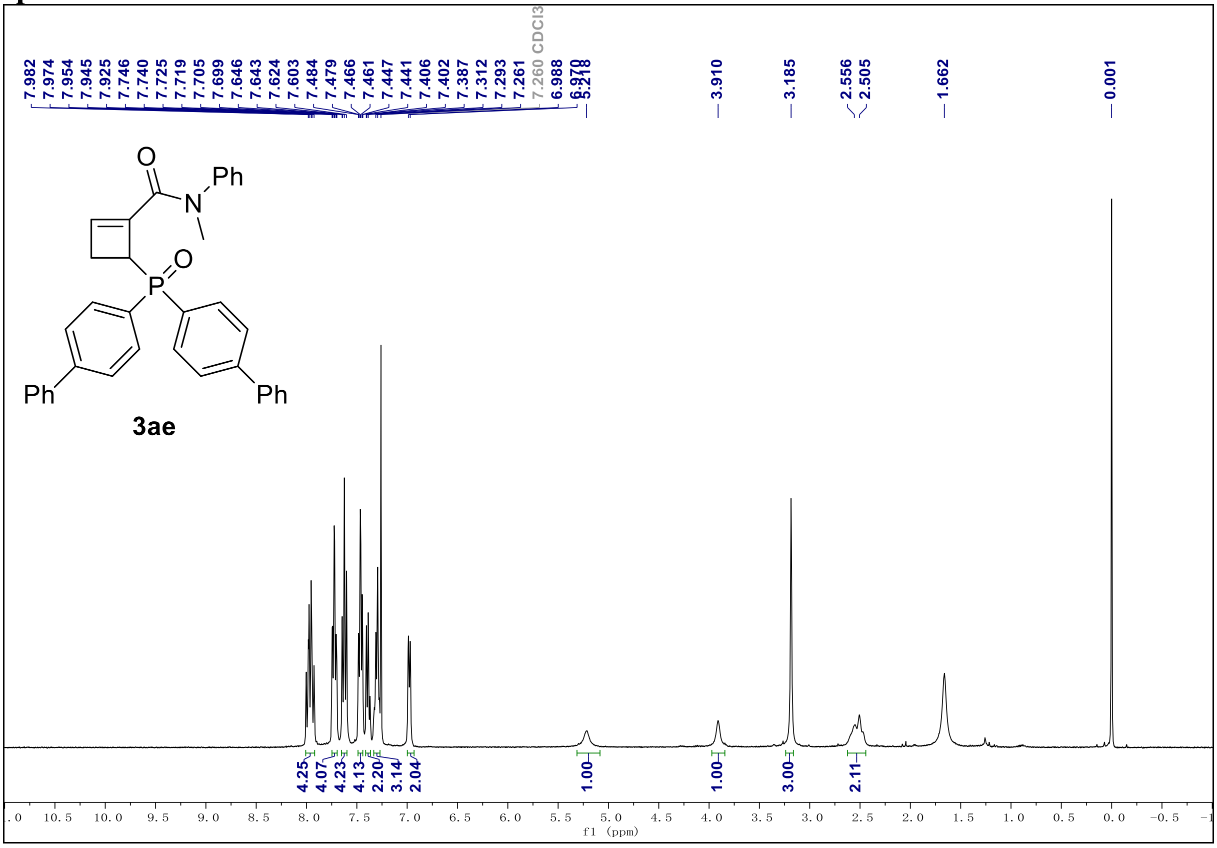

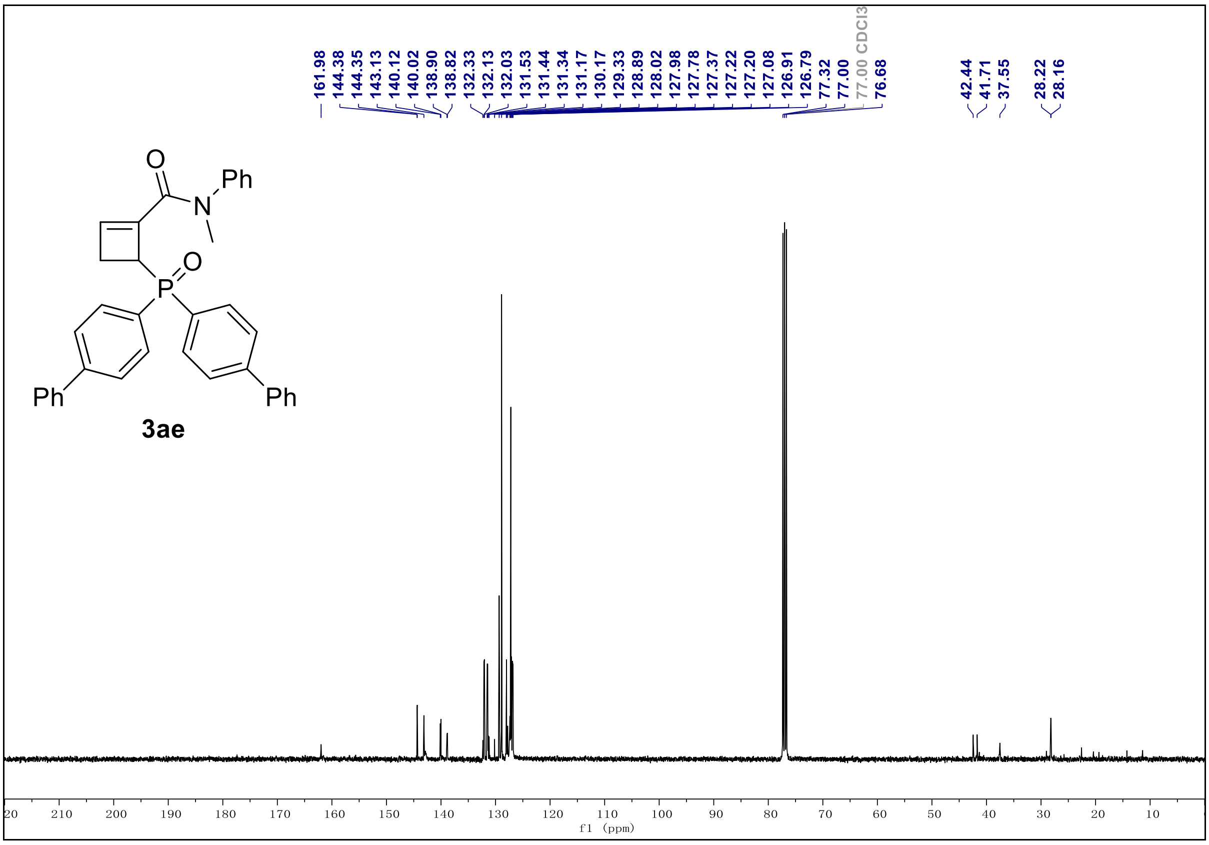

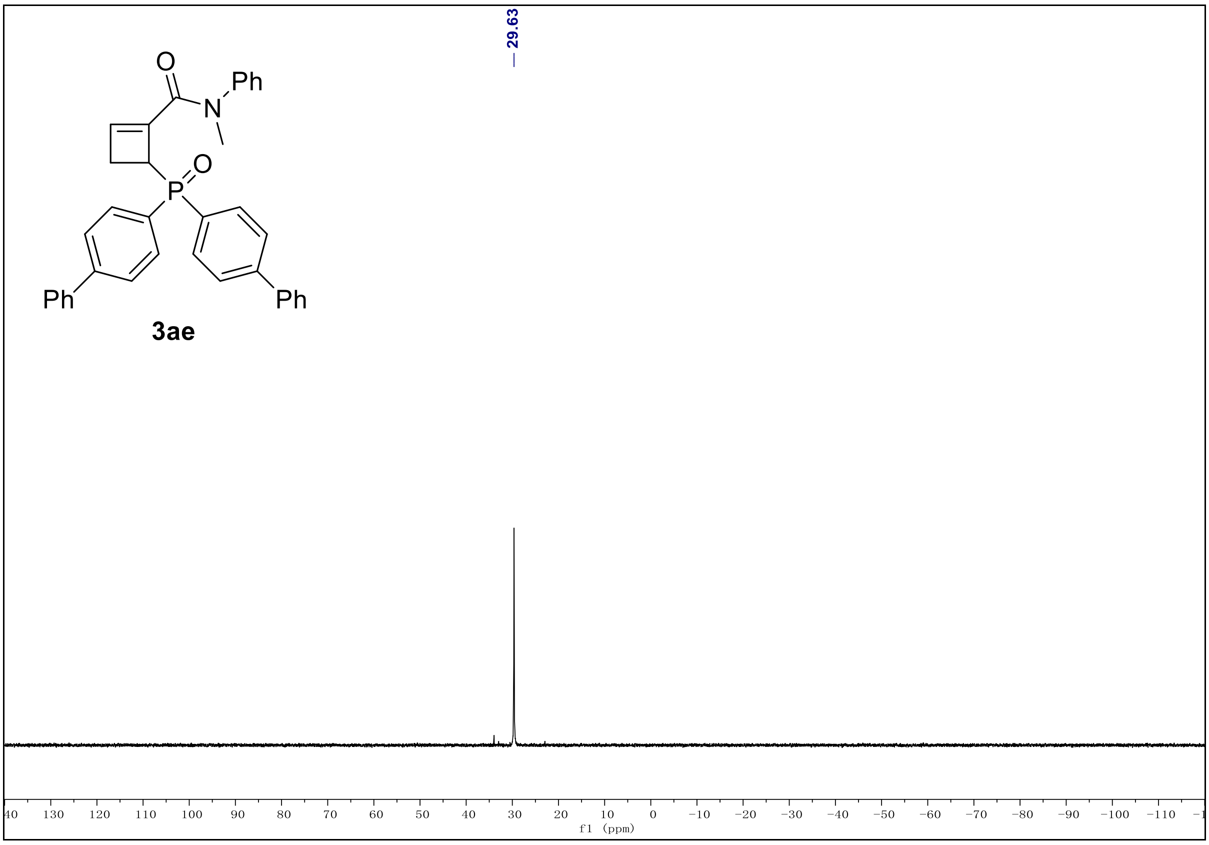
29.63
O
N
Ph
P
O
Ph
Ph
3ae
40
130
120
110
100
90
80
70
60
50
40
30
20
10
0
−10
−20
−30
−40
−50
−60
−70
−80
−90
−100
−110
f1 (ppm)

^{1}H NMR (400 MHz, CDCl3), ^{13}C NMR (100 MHz, CDCl3) and ^{31}P NMR (162 MHz, CDCl3) spectrum of 3af.

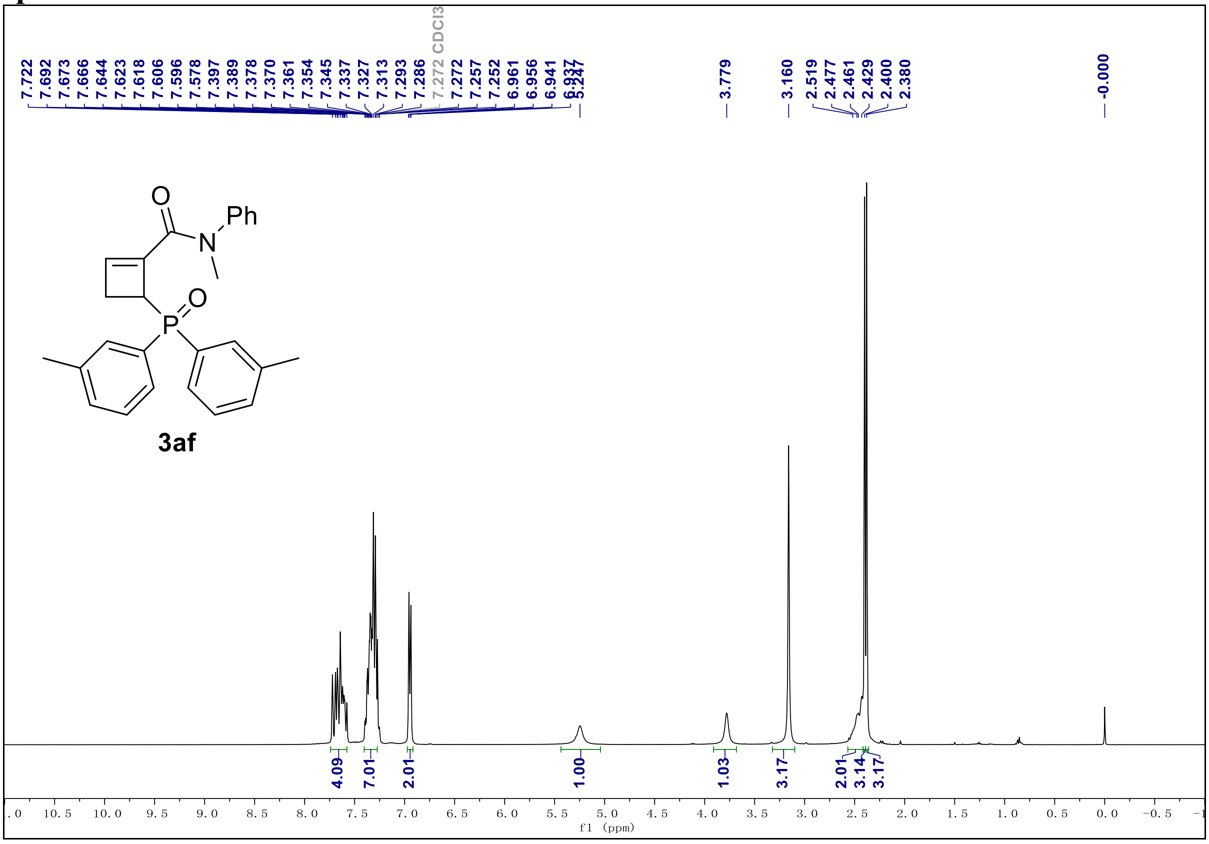

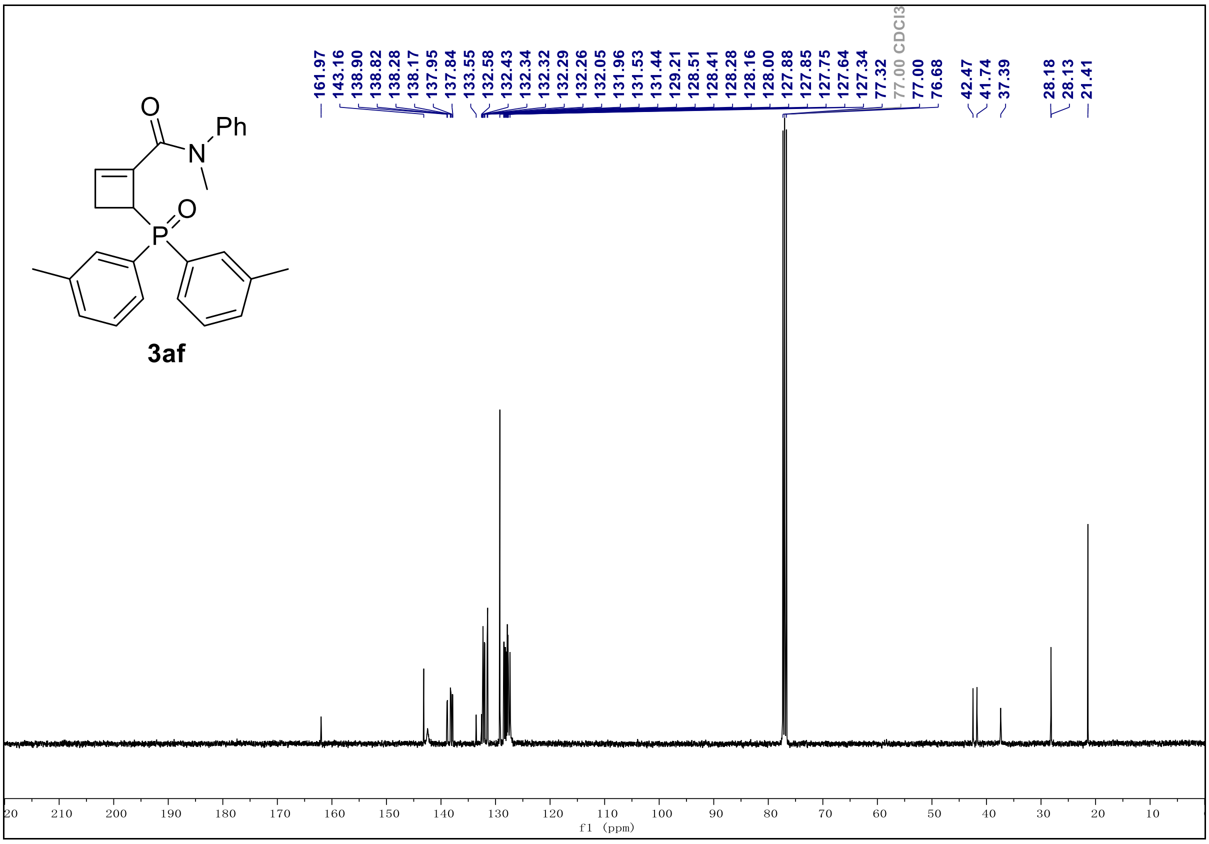

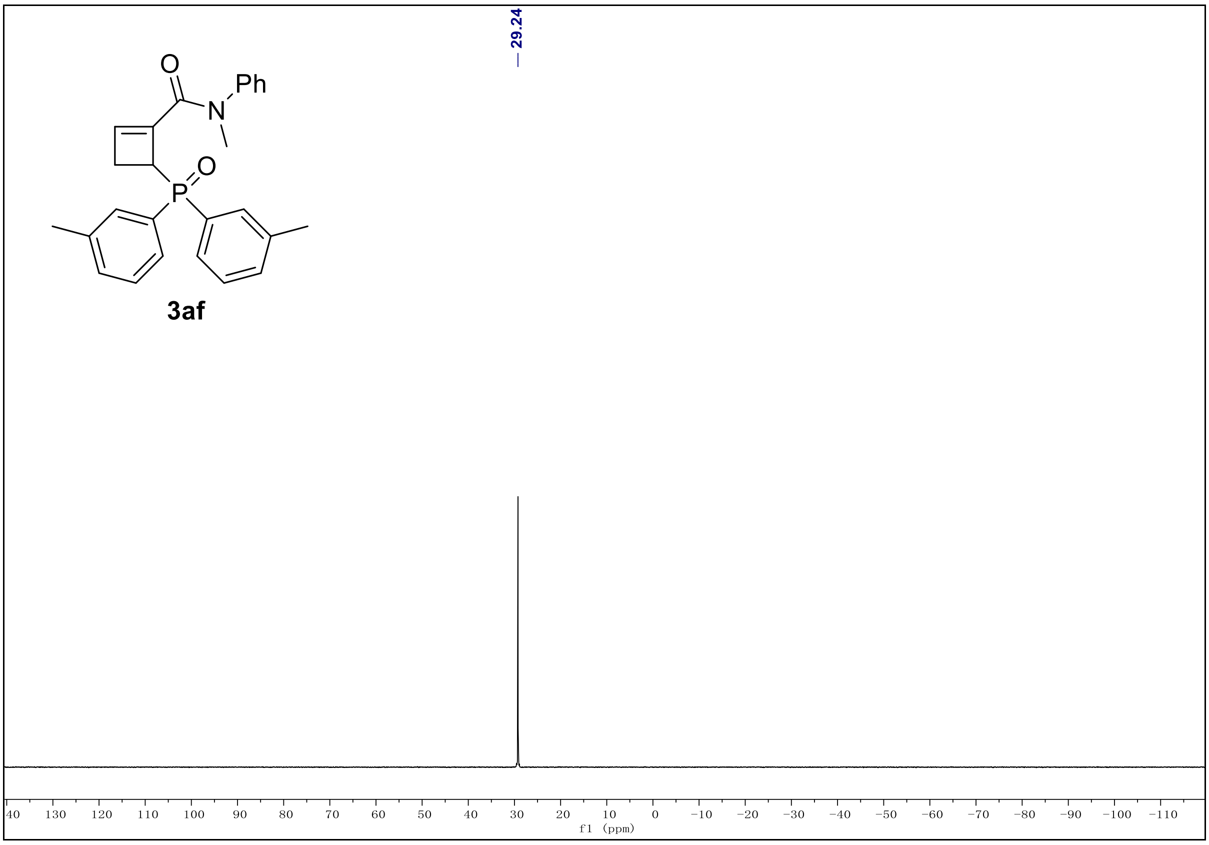

O
N
Ph
P
O
3af
29.24
40
130
120
110
100
90
80
70
60
50
40
30
20
10
0
−10
−20
−30
−40
−50
−60
−70
−80
−90
−100
−110
f1 (ppm)

^{1}H NMR (400 MHz, CDCl3), ^{13}C NMR (100 MHz, CDCl3), ^{19}F NMR (376 MHz, CDCl3) and ^{31}P NMR (162 MHz, CDCl3) spectrum of 3ag.

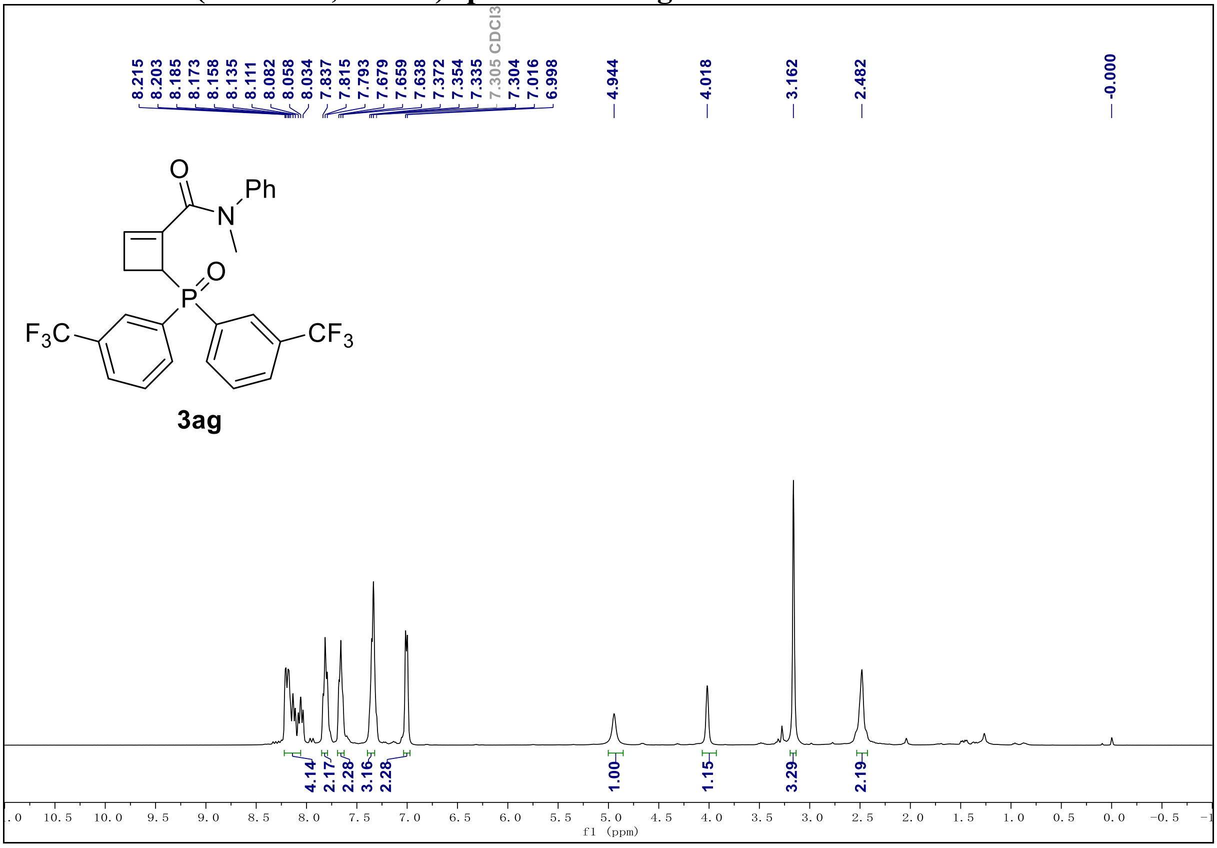

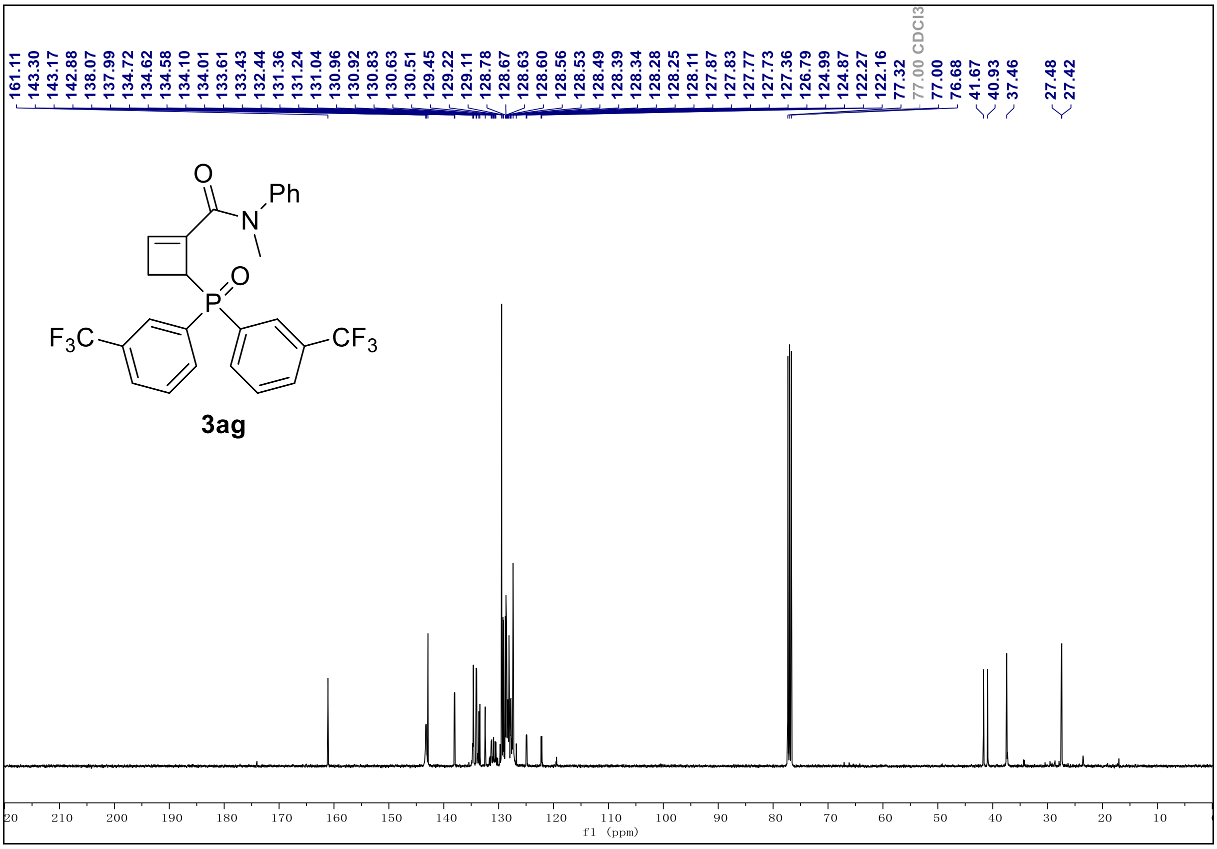

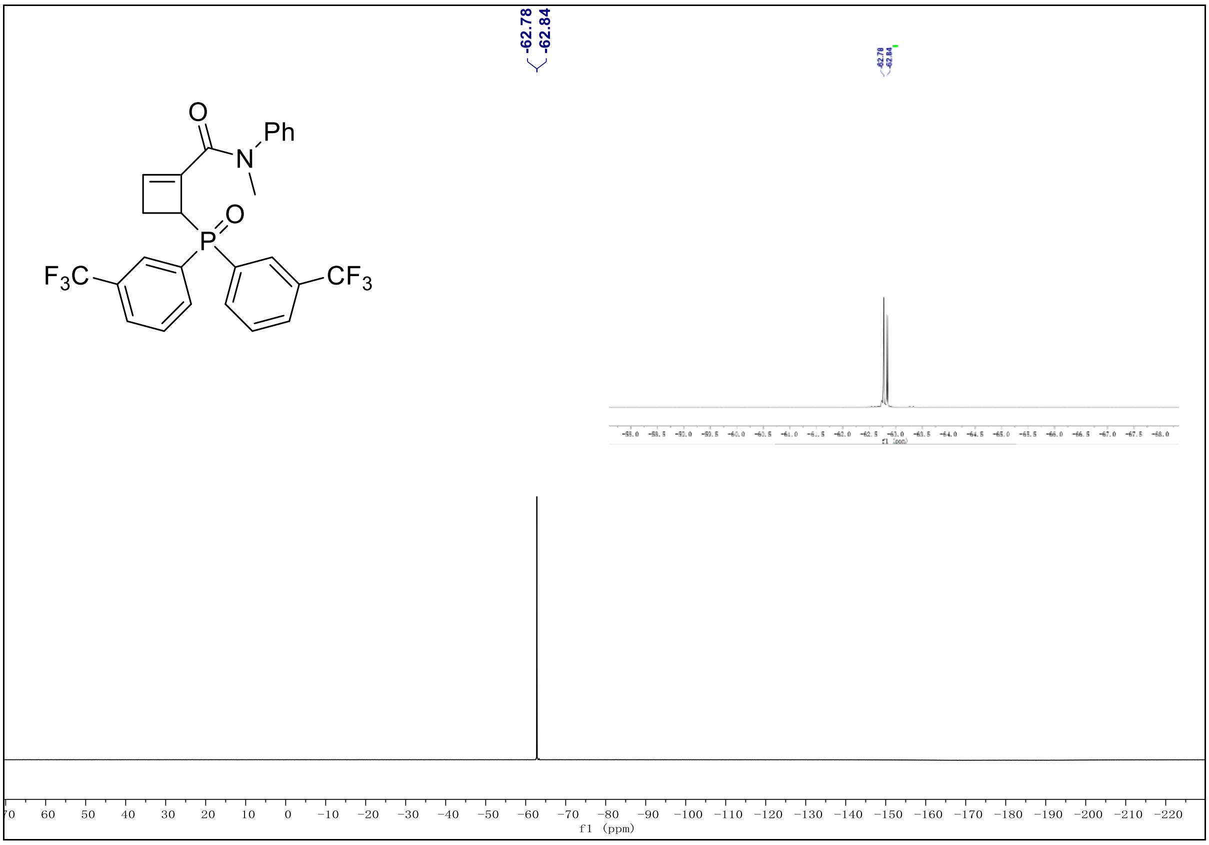

-62.78
-62.84
F3C
CF3
O
N
Ph
P
O
f1 (ppm)

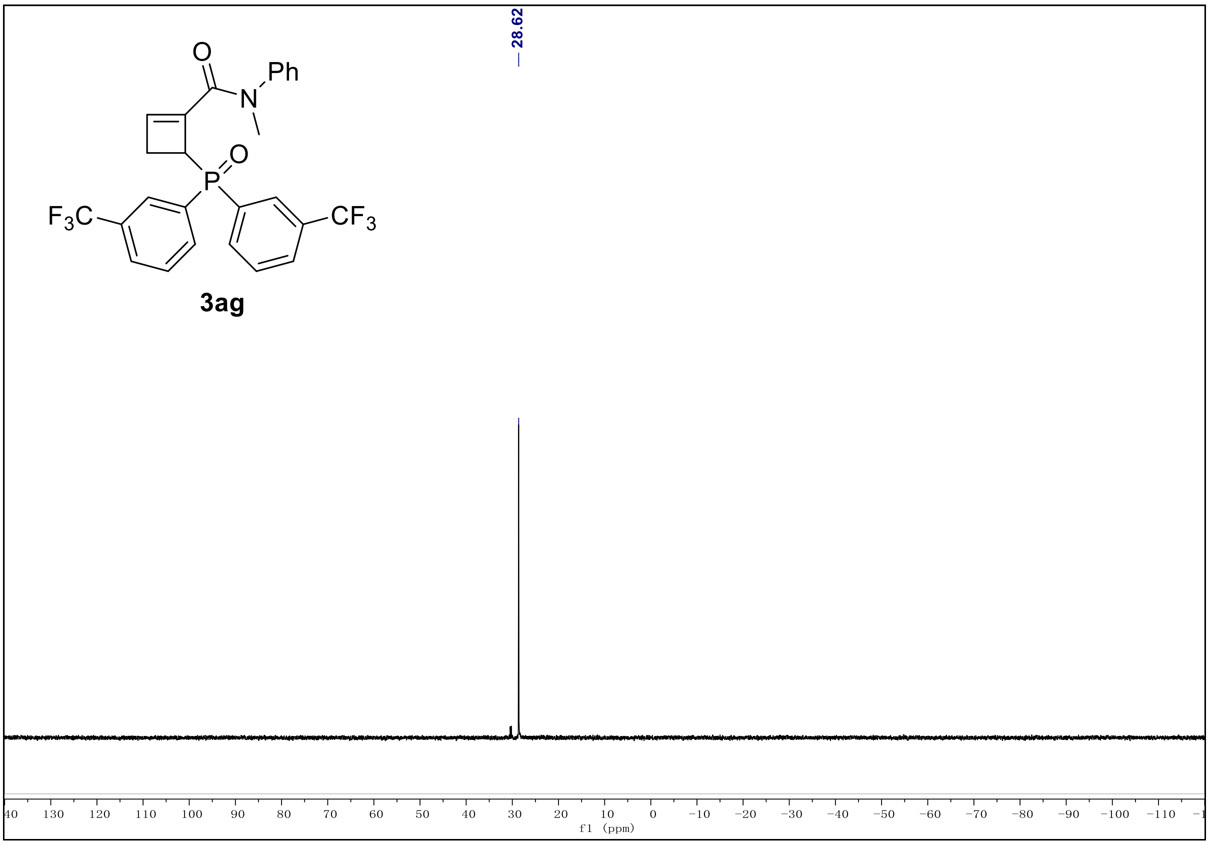

28.62
F3C
CF3
O
N
Ph
P
O
3ag
f1 (ppm)

^{1}H NMR (400 MHz, CDCl₃), ^{13}C NMR (100 MHz, CDCl₃), ^{19}F NMR (376 MHz, CDCl₃) and ^{31}P NMR (162 MHz, CDCl₃) spectrum of 3ah.

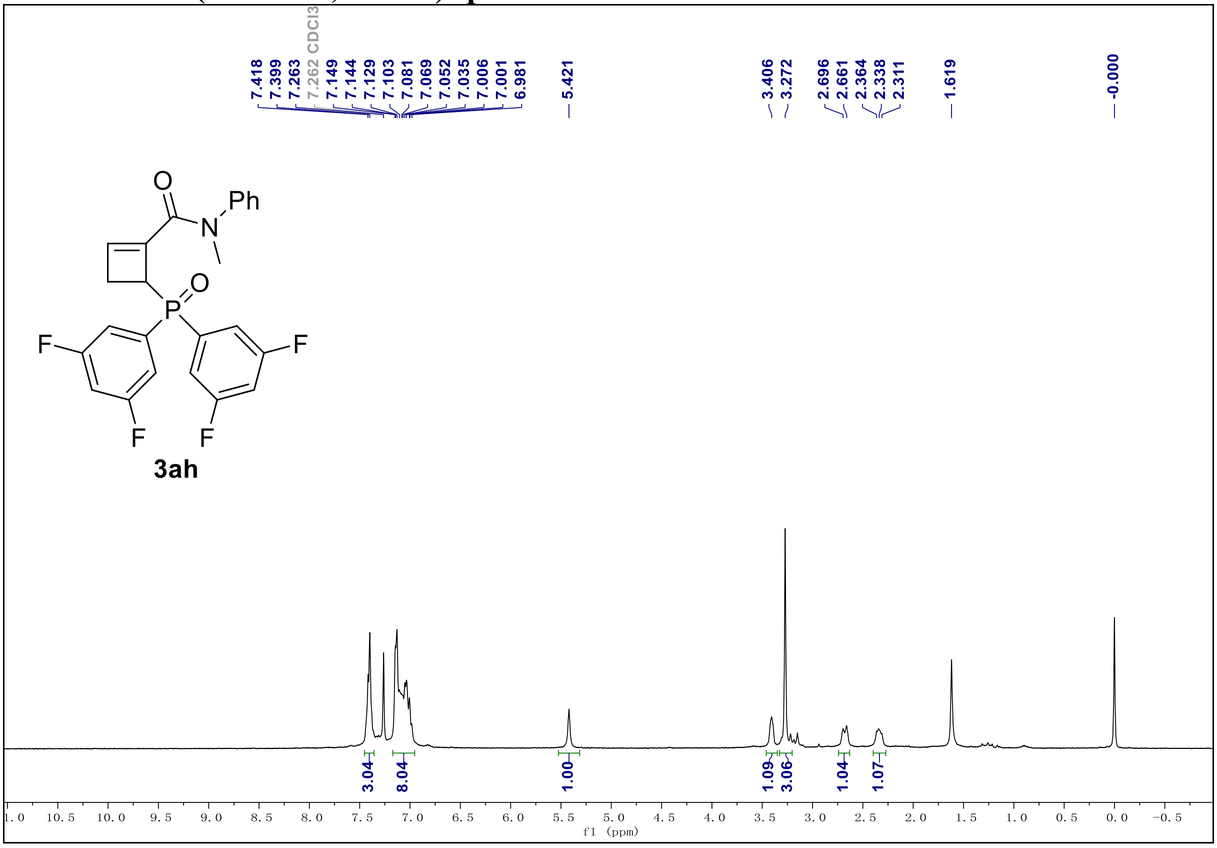

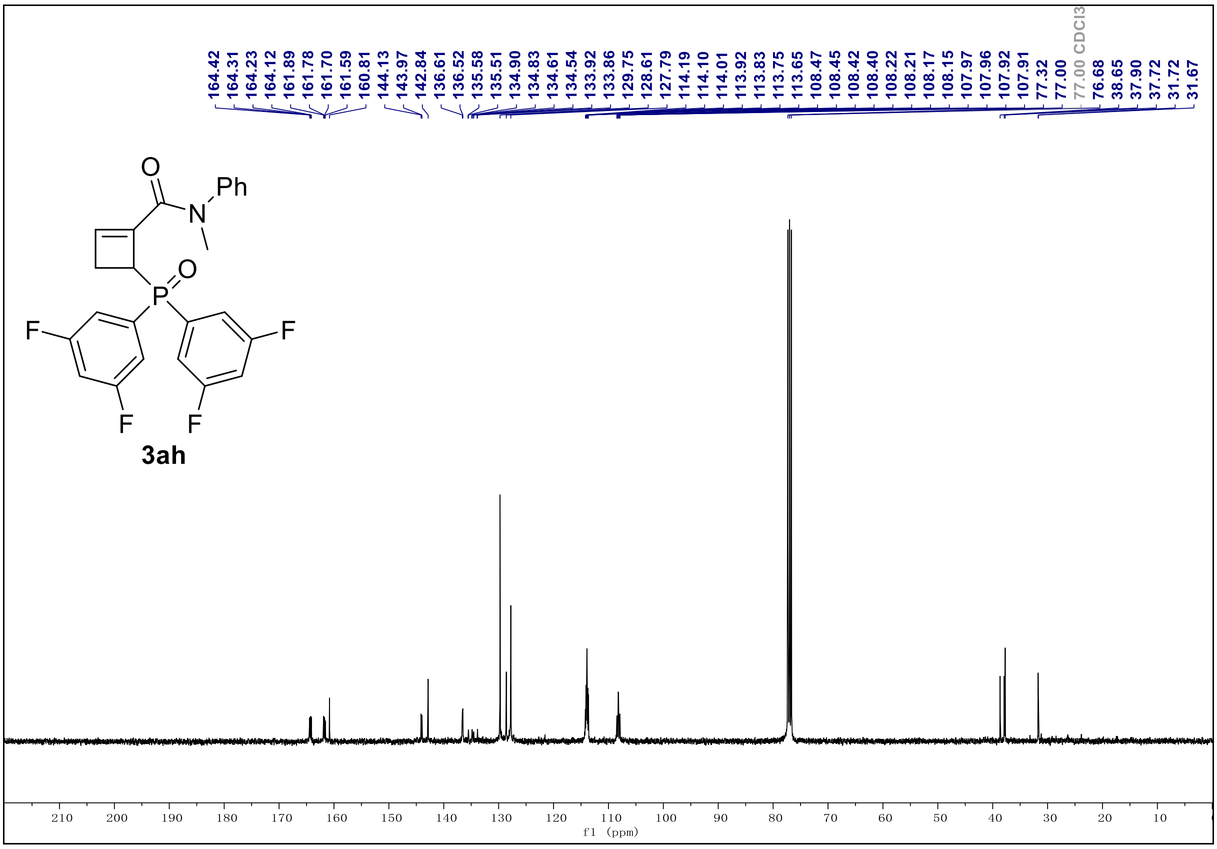

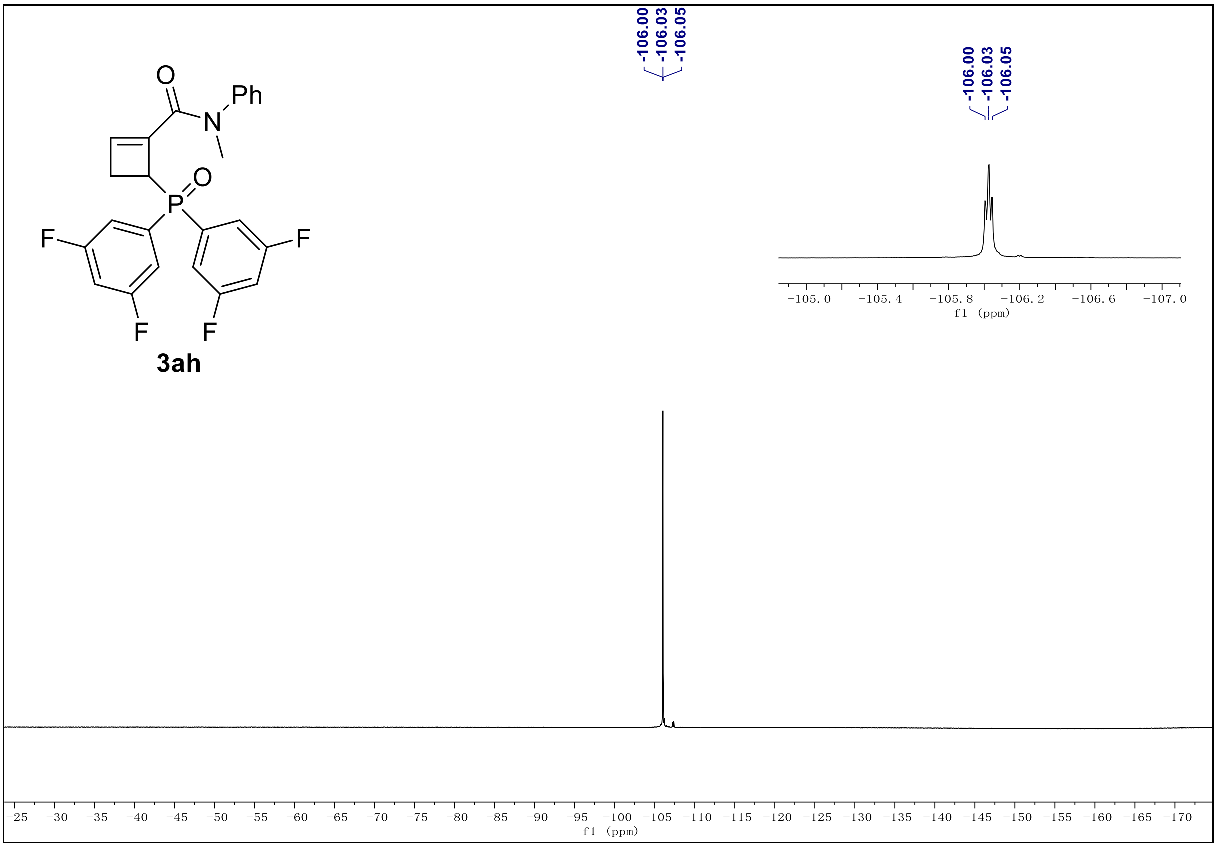

-106.00
-106.03
-106.05
O
N
Ph
P
O
F
F
F
F
3ah
-105.0 -105.4 -105.8 -106.2 -106.6 -107.0
f1 (ppm)
-25 -30 -35 -40 -45 -50 -55 -60 -65 -70 -75 -80 -85 -90 -95 -100 -105 -110 -115 -120 -125 -130 -135 -140 -145 -150 -155 -160 -165 -170
f1 (ppm)

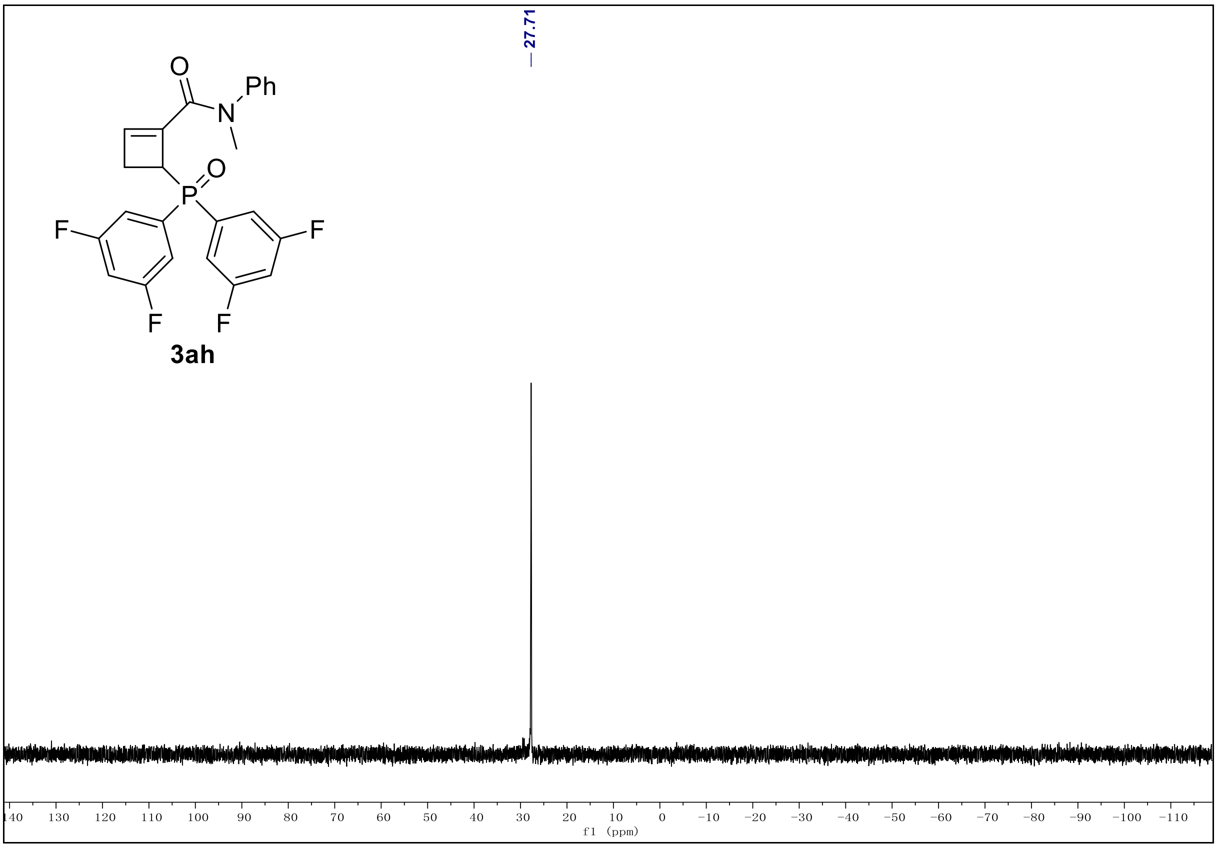

27.71
O
N
Ph
P
O
F
F
F
F
3ah
140 130 120 110 100 90 80 70 60 50 40 30 20 10 0 -10 -20 -30 -40 -50 -60 -70 -80 -90 -100 -110
f1 (ppm)

^{1}H NMR (400 MHz, $CDCl_3$), ^{13}C NMR (100 MHz, $CDCl_3$) and ^{31}P NMR (162 MHz, $CDCl_3$) spectrum of 3ai.

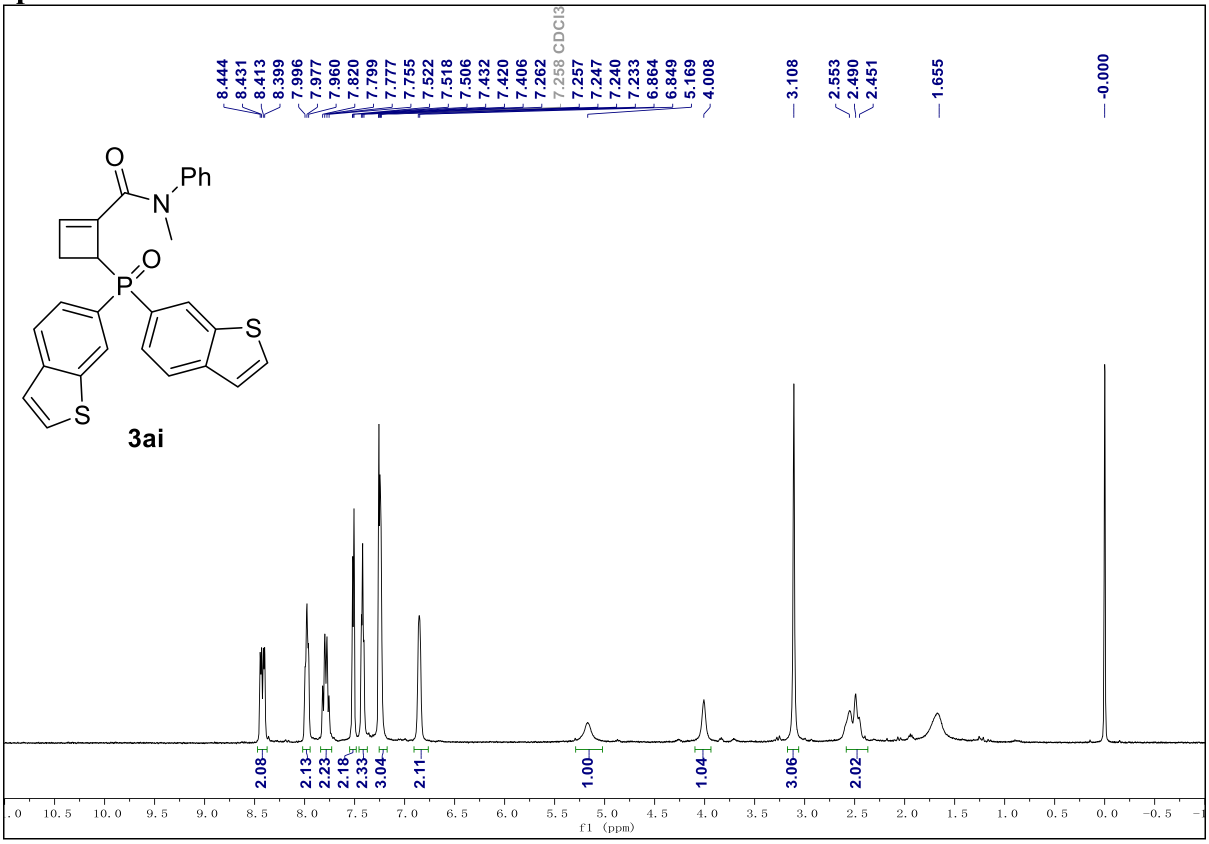

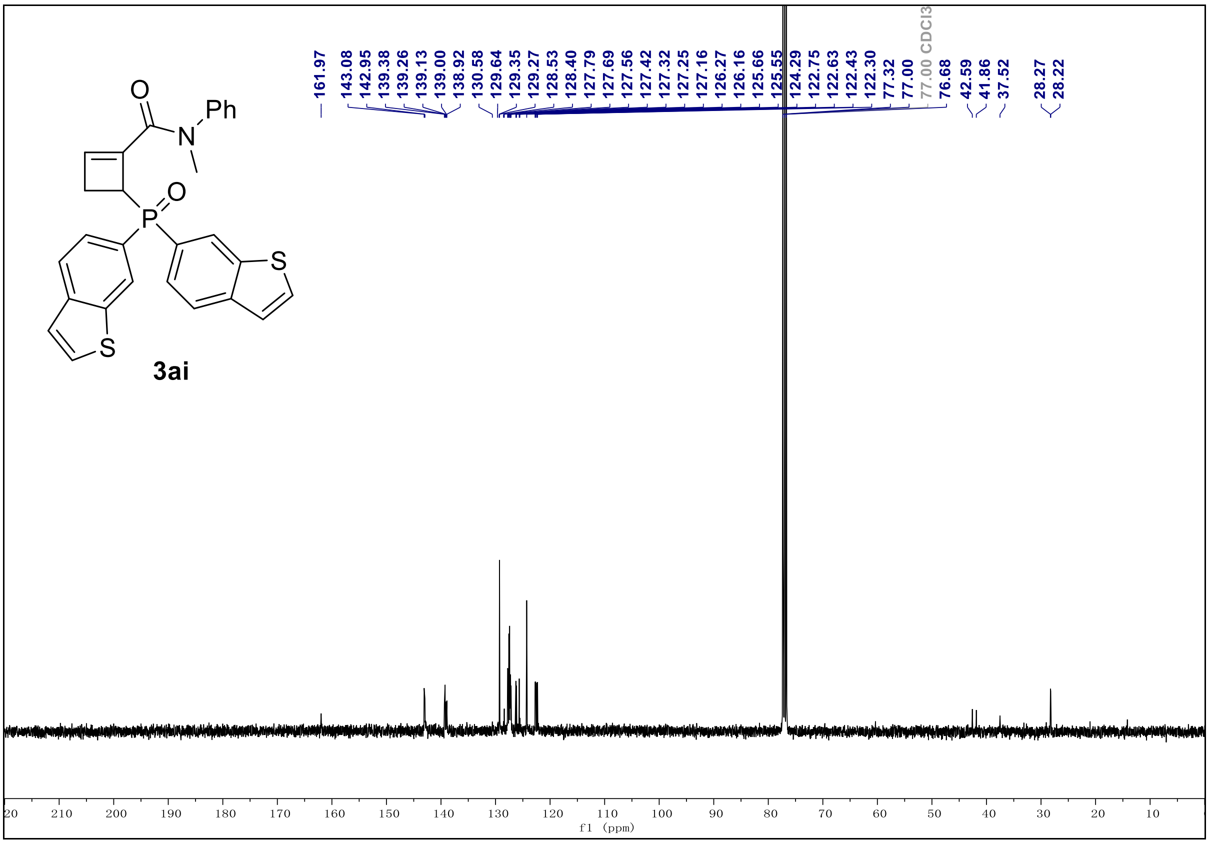

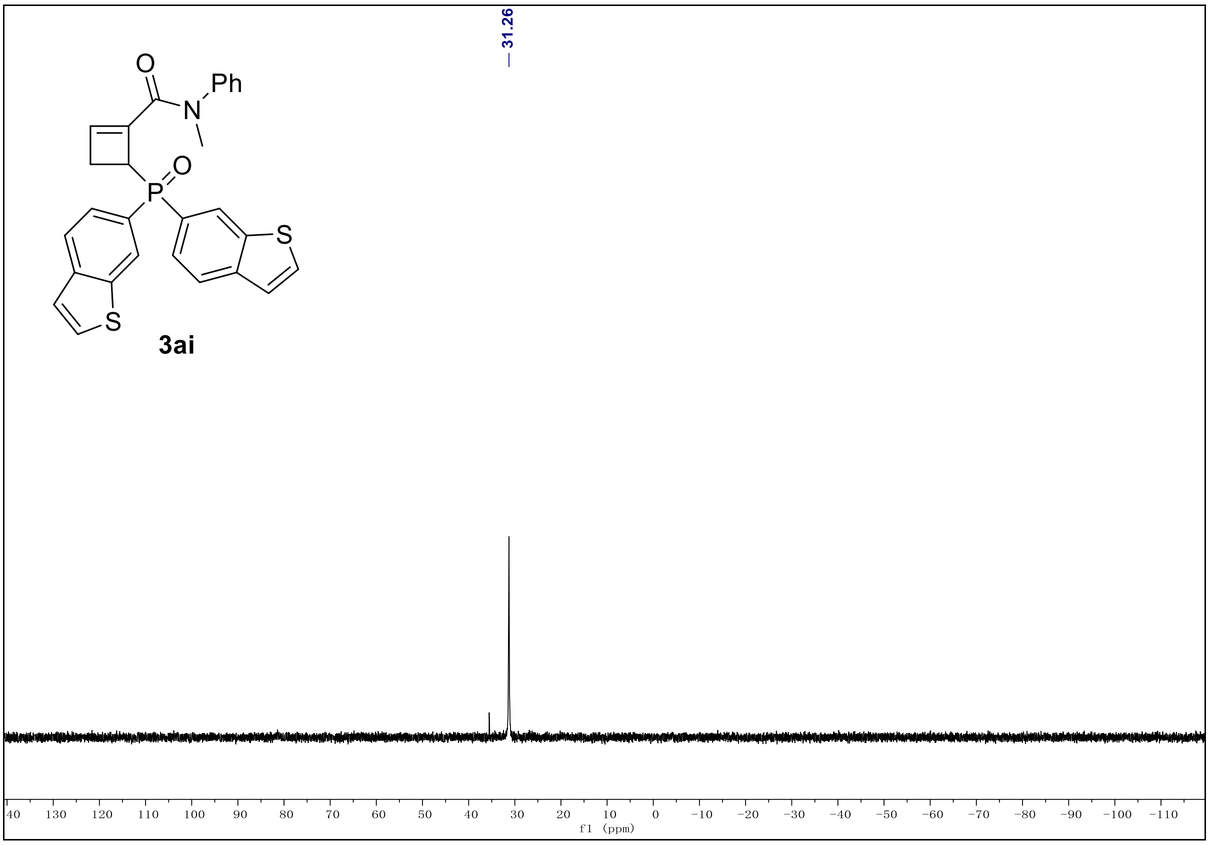
3ai
31.26
f1 (ppm)

^{1}H NMR (400 MHz, $CDCl_3$), ^{13}C NMR (100 MHz, $CDCl_3$) and ^{31}P NMR (162 MHz, $CDCl_3$) spectrum of 3aj.

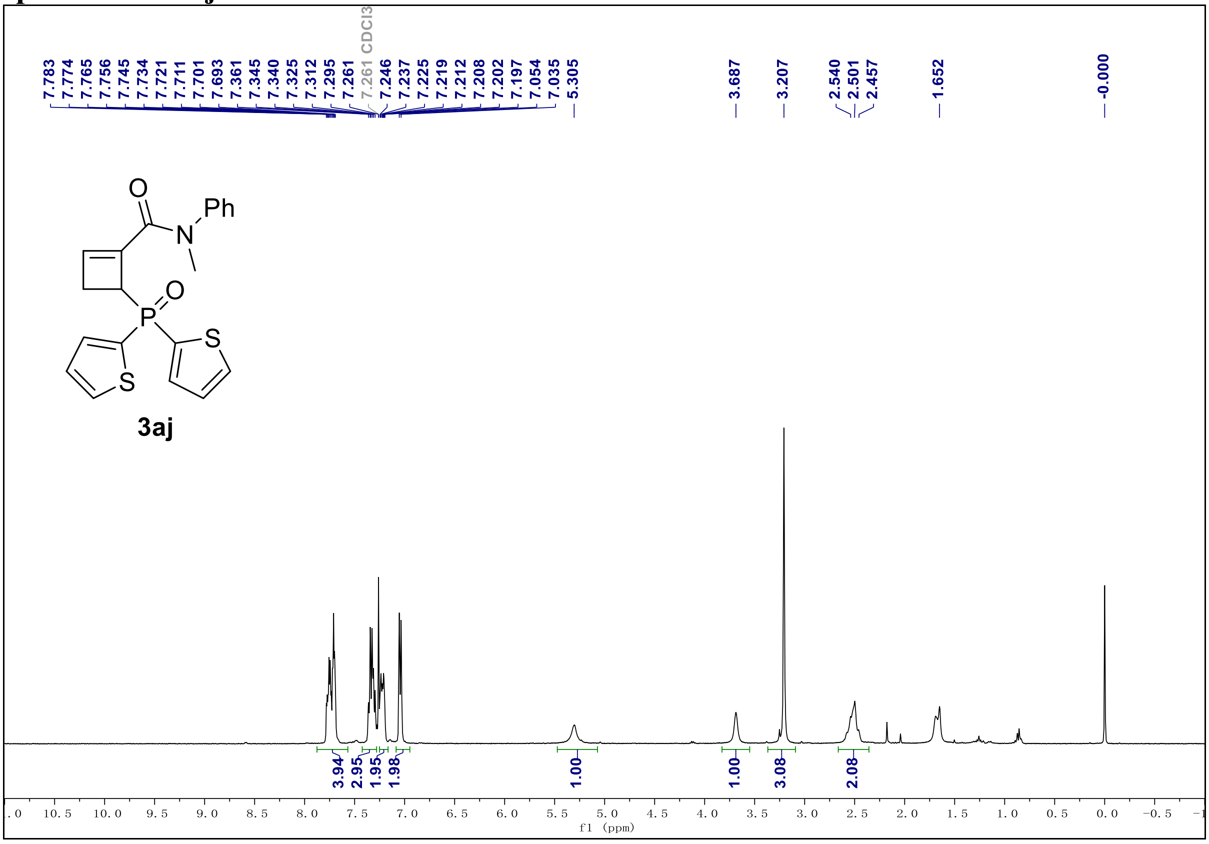

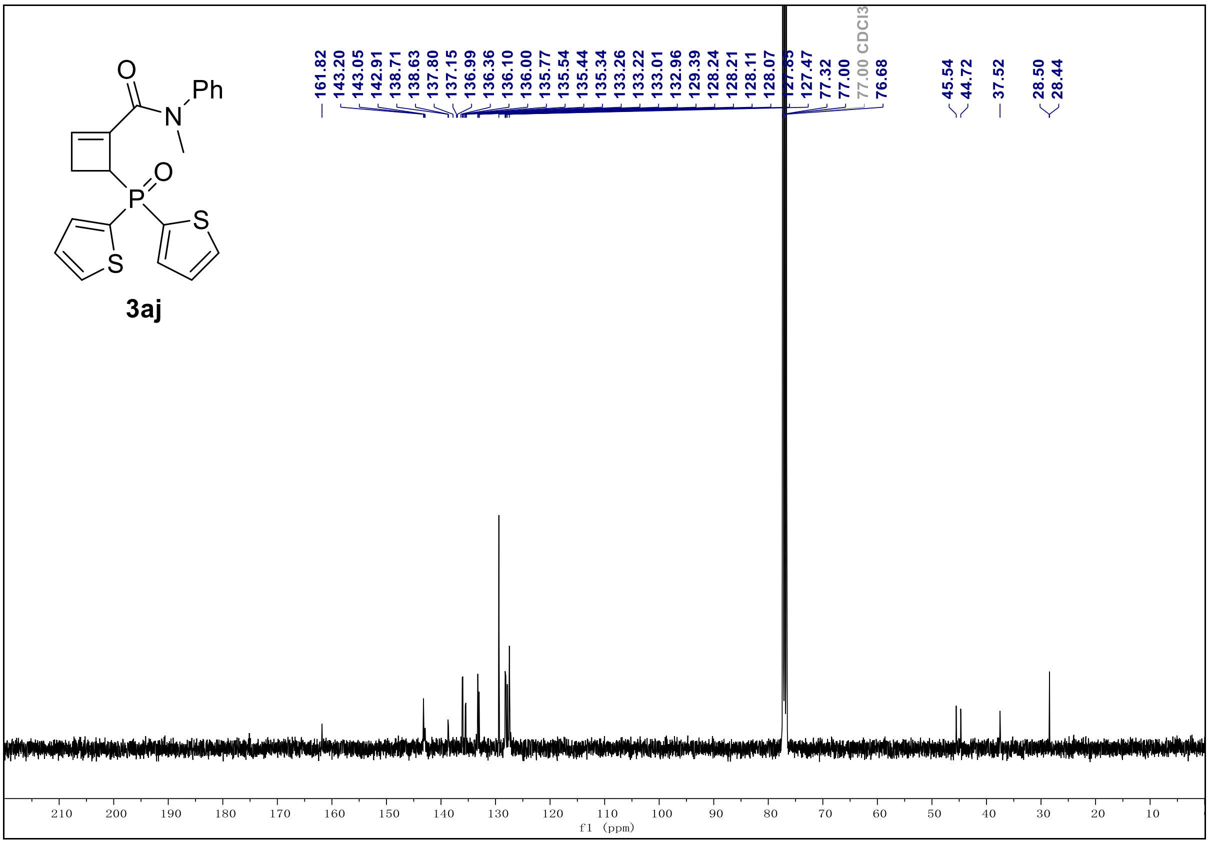

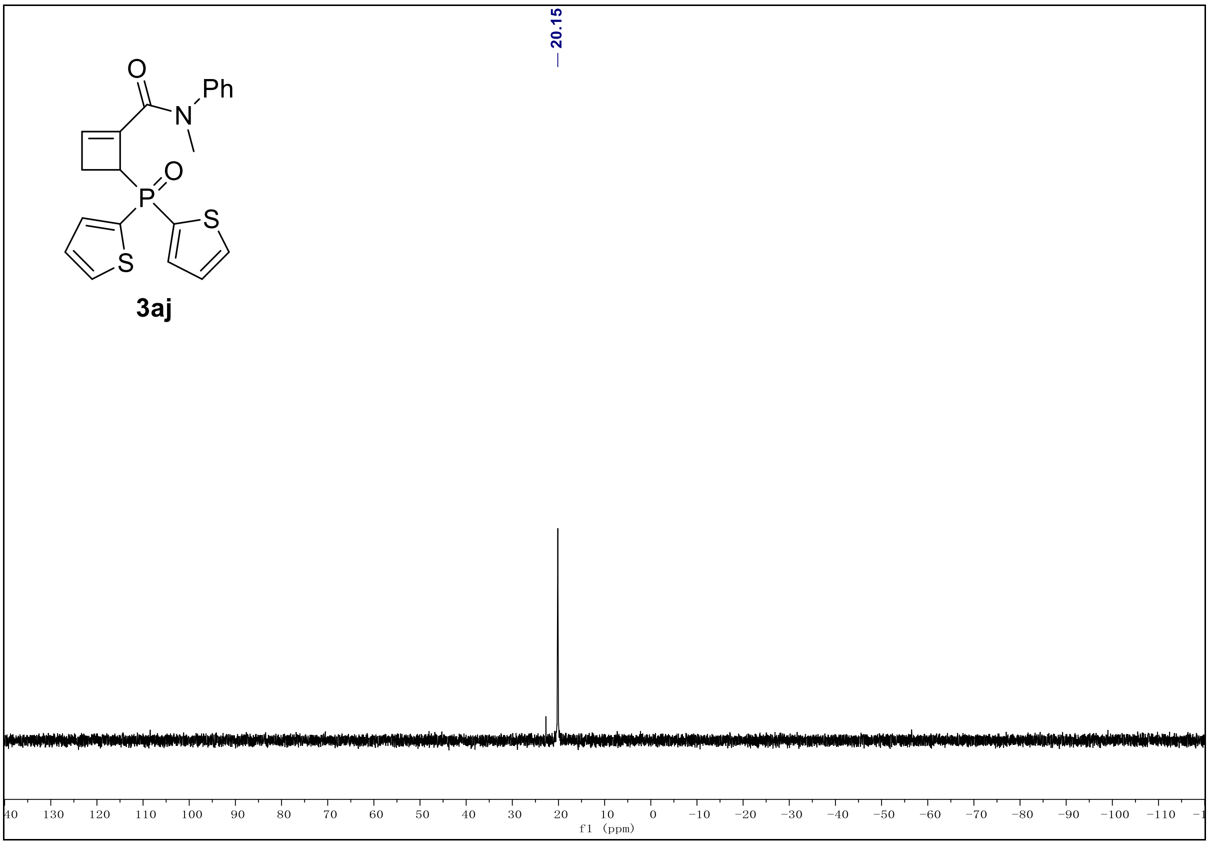

3aj
— 20.15
40
130
120
110
100
90
80
70
60
50
40
30
20
10
0
−10
−20
−30
−40
−50
−60
−70
−80
−90
−100
−110
f1 (ppm)

^{1}H NMR (400 MHz, $CDCl_3$), ^{13}C NMR (100 MHz, $CDCl_3$) and ^{31}P NMR (162 MHz, $CDCl_3$) spectrum of 3ak.

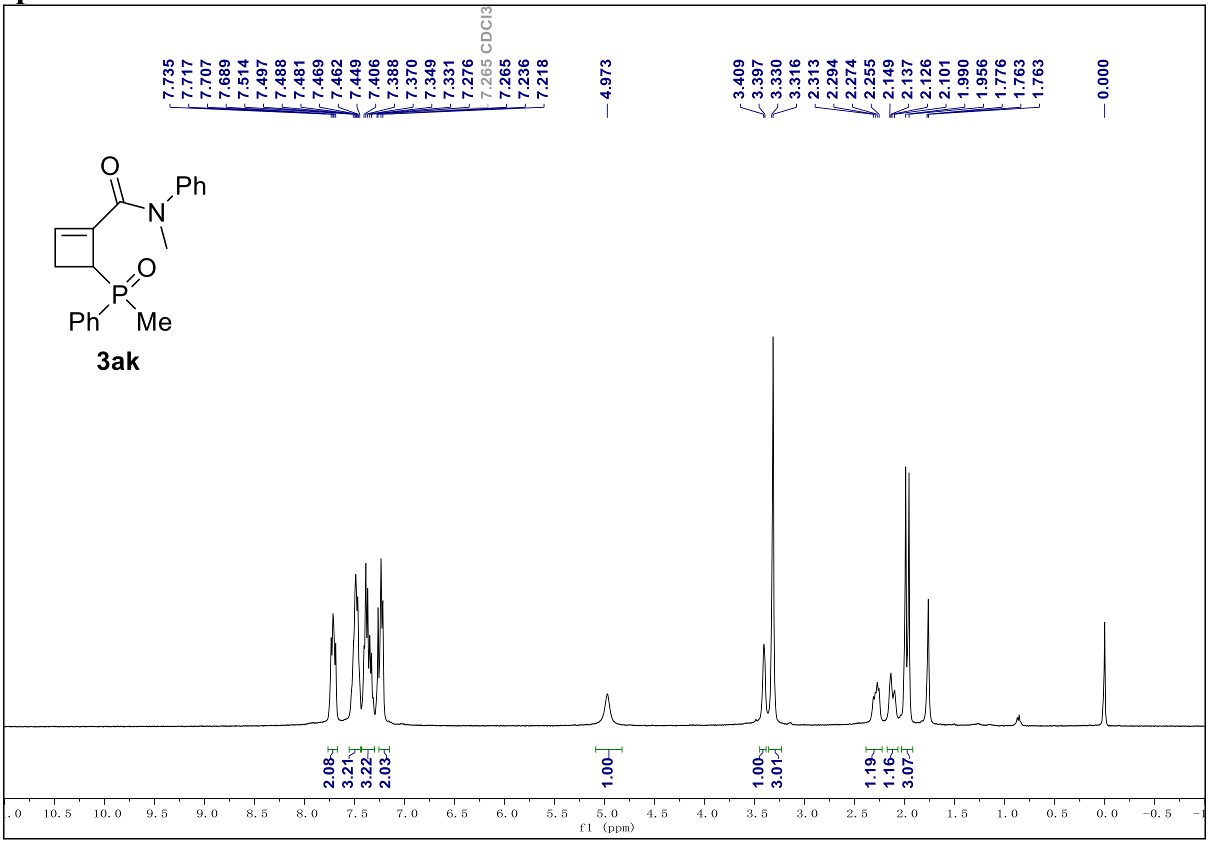

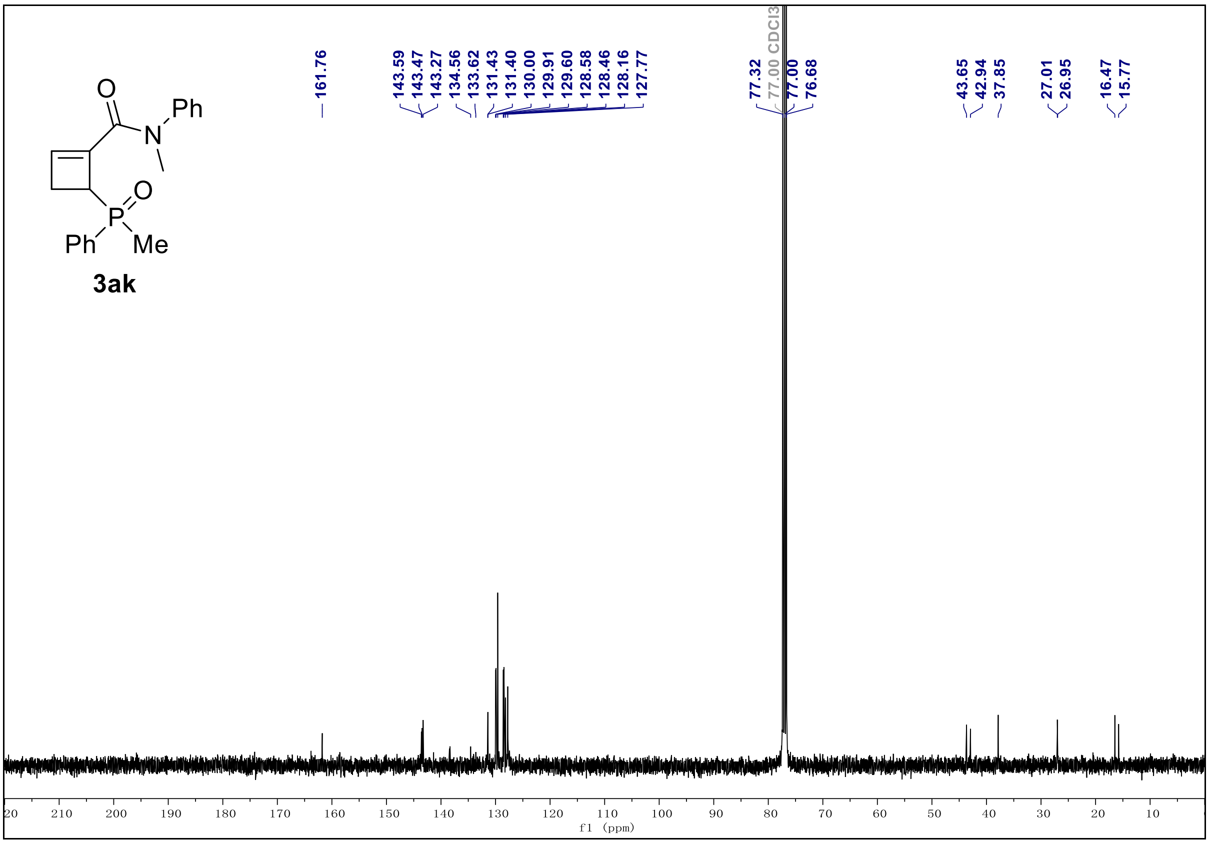

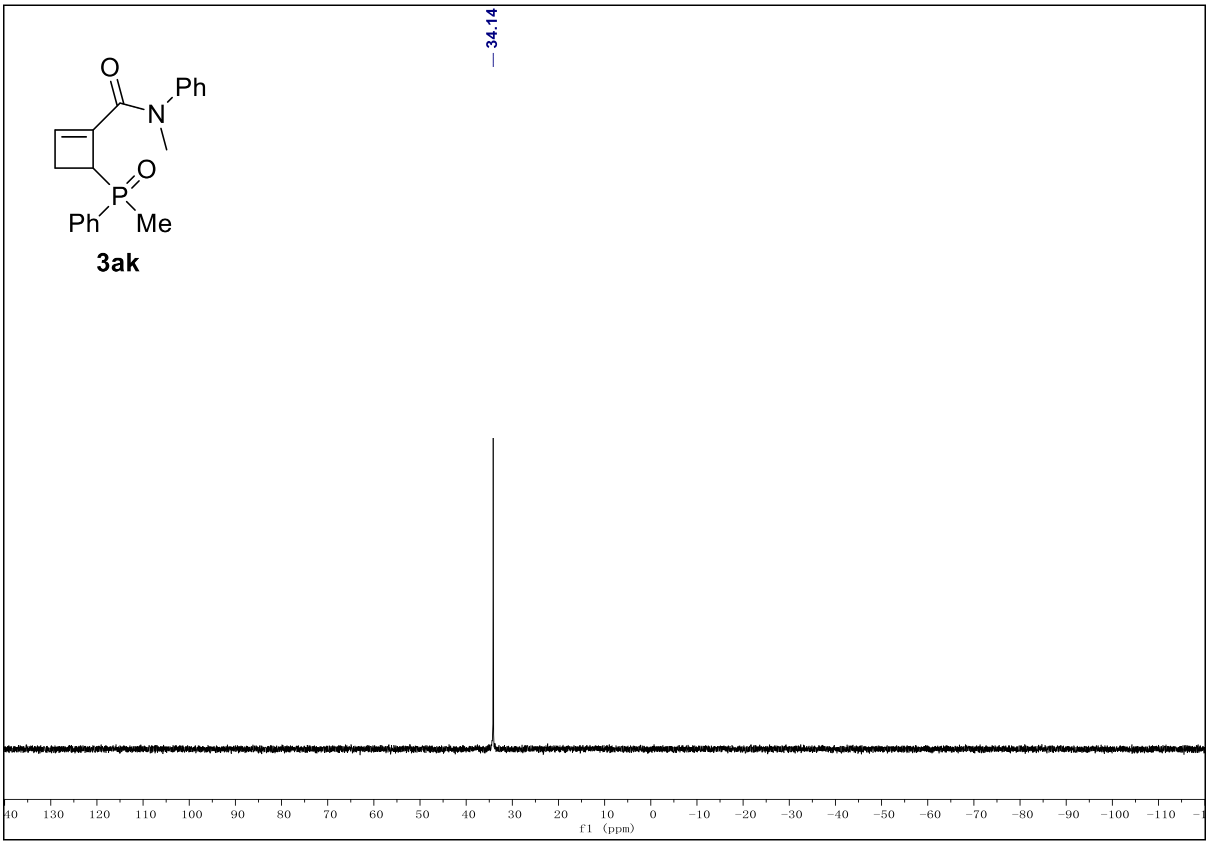
34.14
3ak
f1 (ppm)

^{1}H NMR (400 MHz, $CDCl_3$), ^{13}C NMR (100 MHz, $CDCl_3$) and ^{31}P NMR (162 MHz, $CDCl_3$) spectrum of 3al (major).

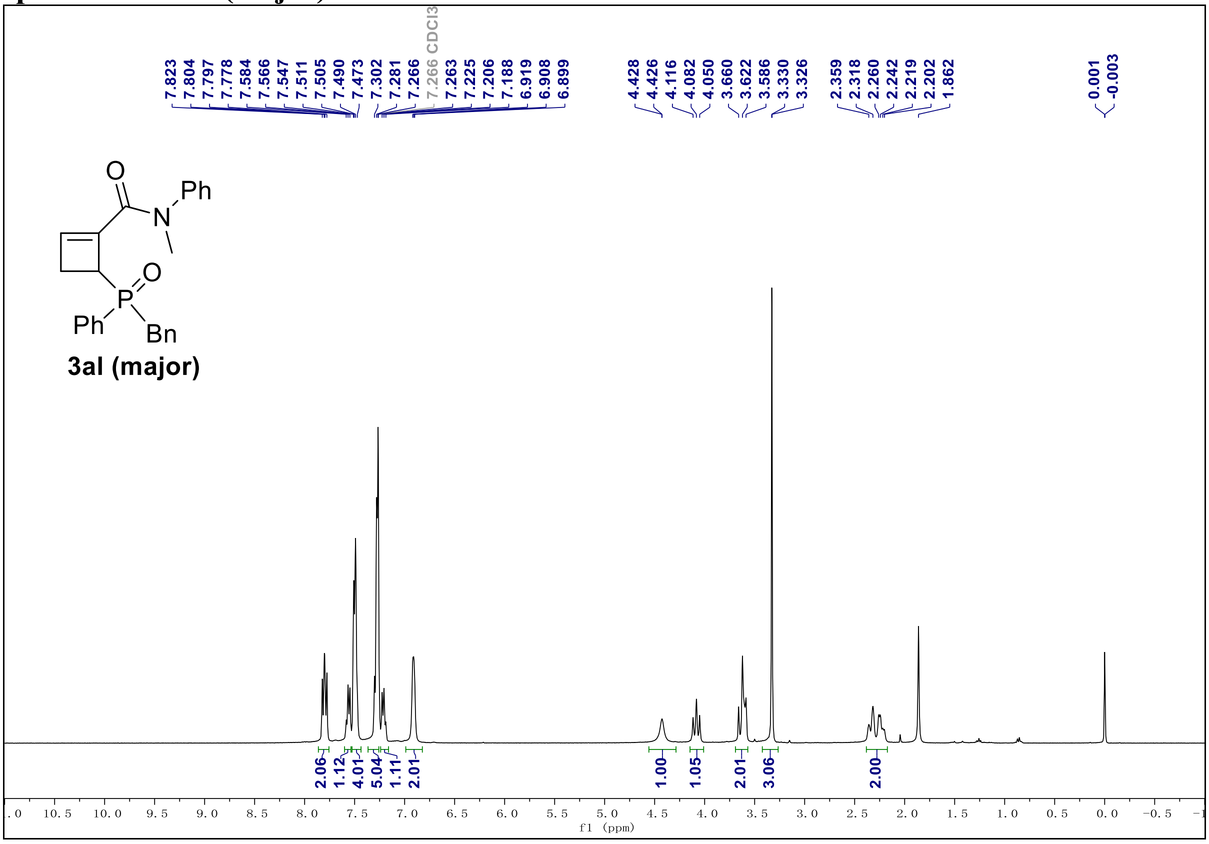

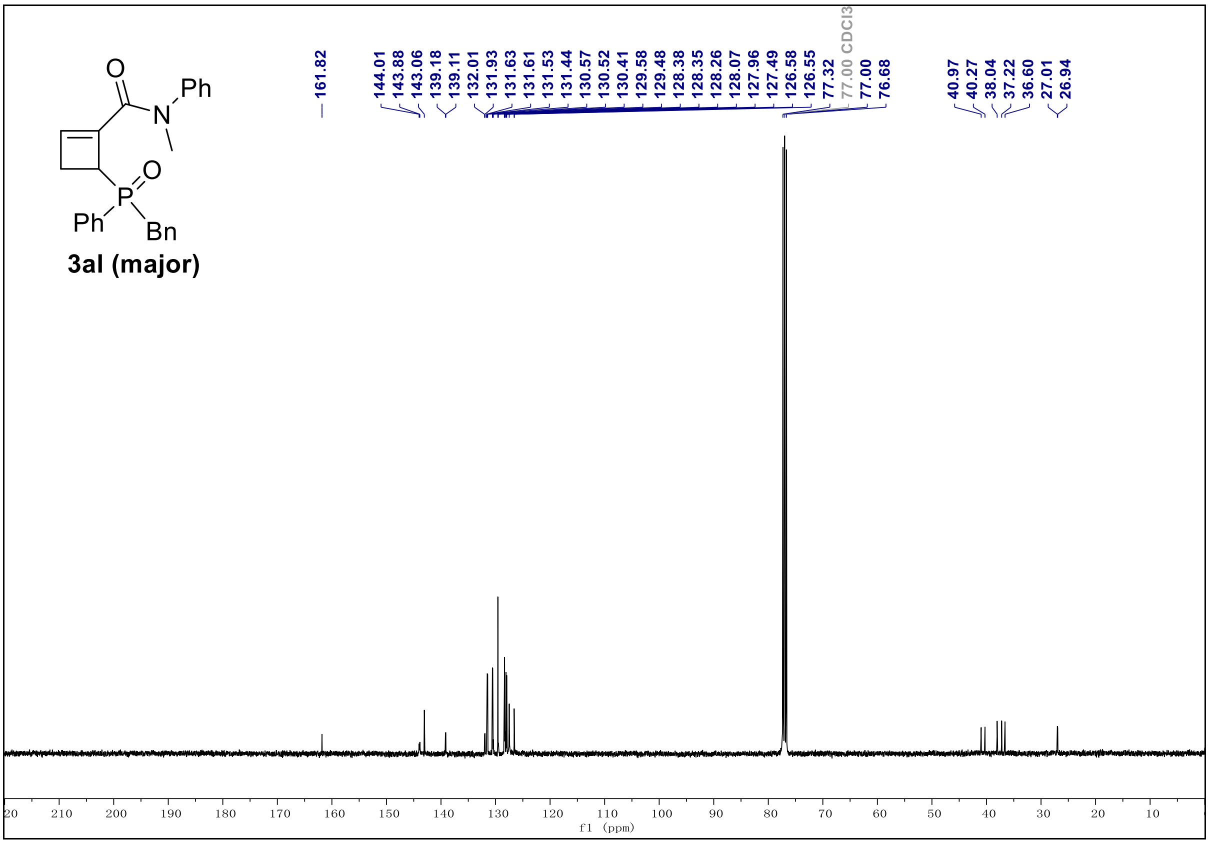

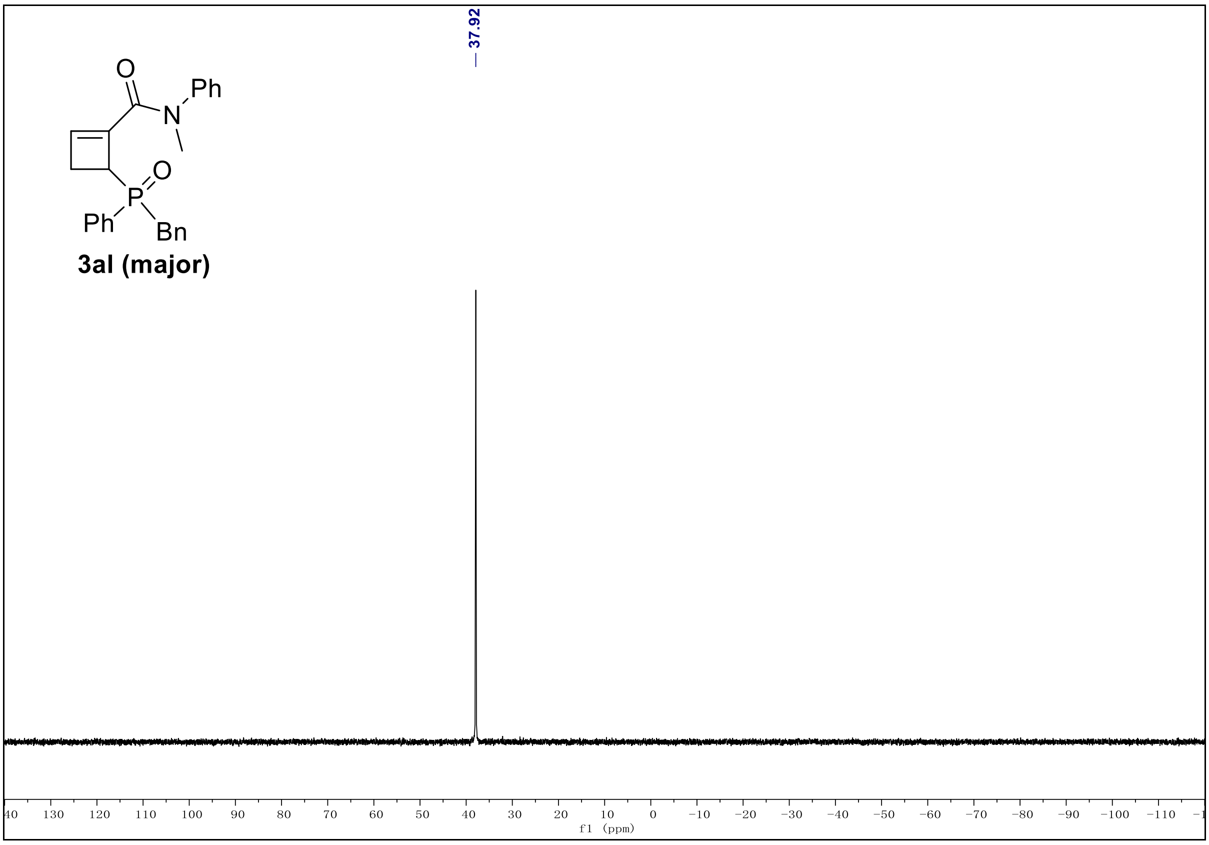
37.92
O
Ph
N
P
O
Ph
Bn
3al (major)
f1 (ppm)

^{1}H NMR (400 MHz, $CDCl_3$), ^{13}C NMR (100 MHz, $CDCl_3$) and ^{31}P NMR (162 MHz, $CDCl_3$) spectrum of 3al (minor).

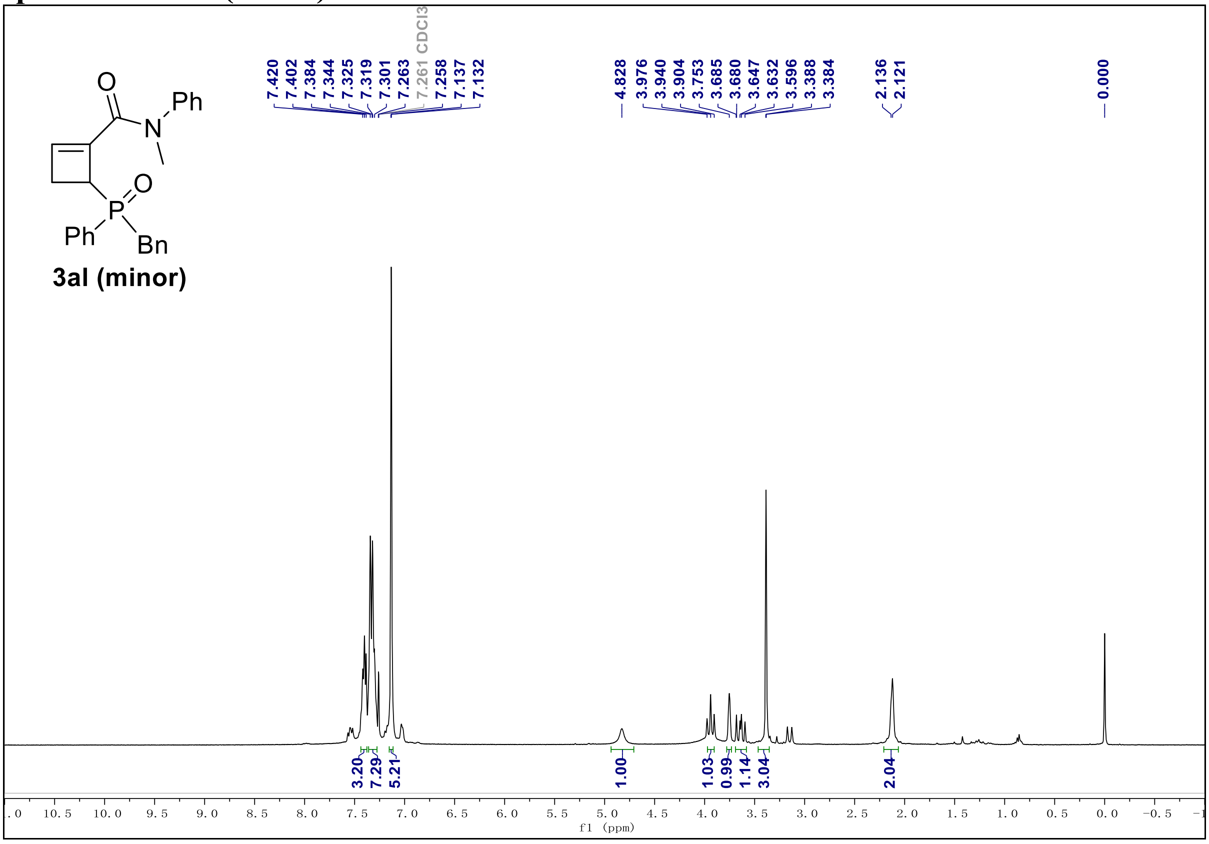

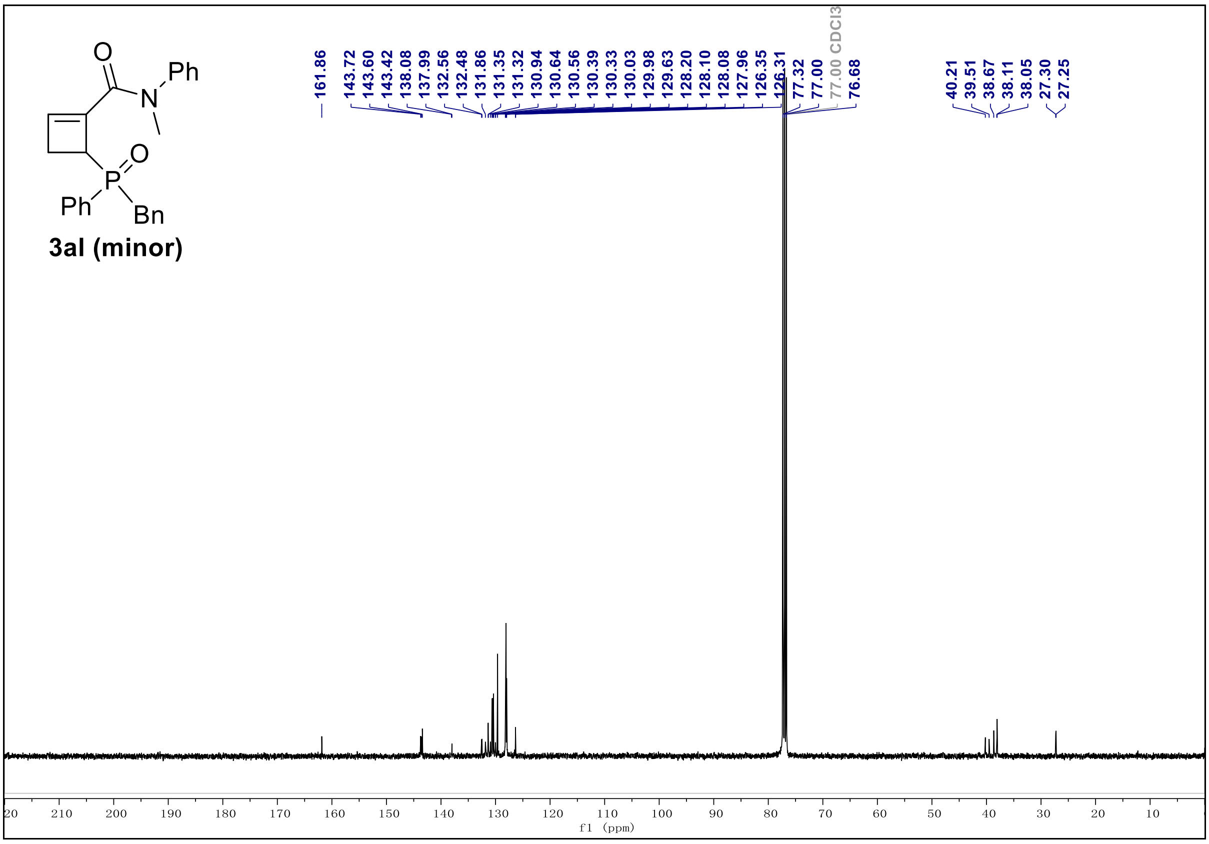

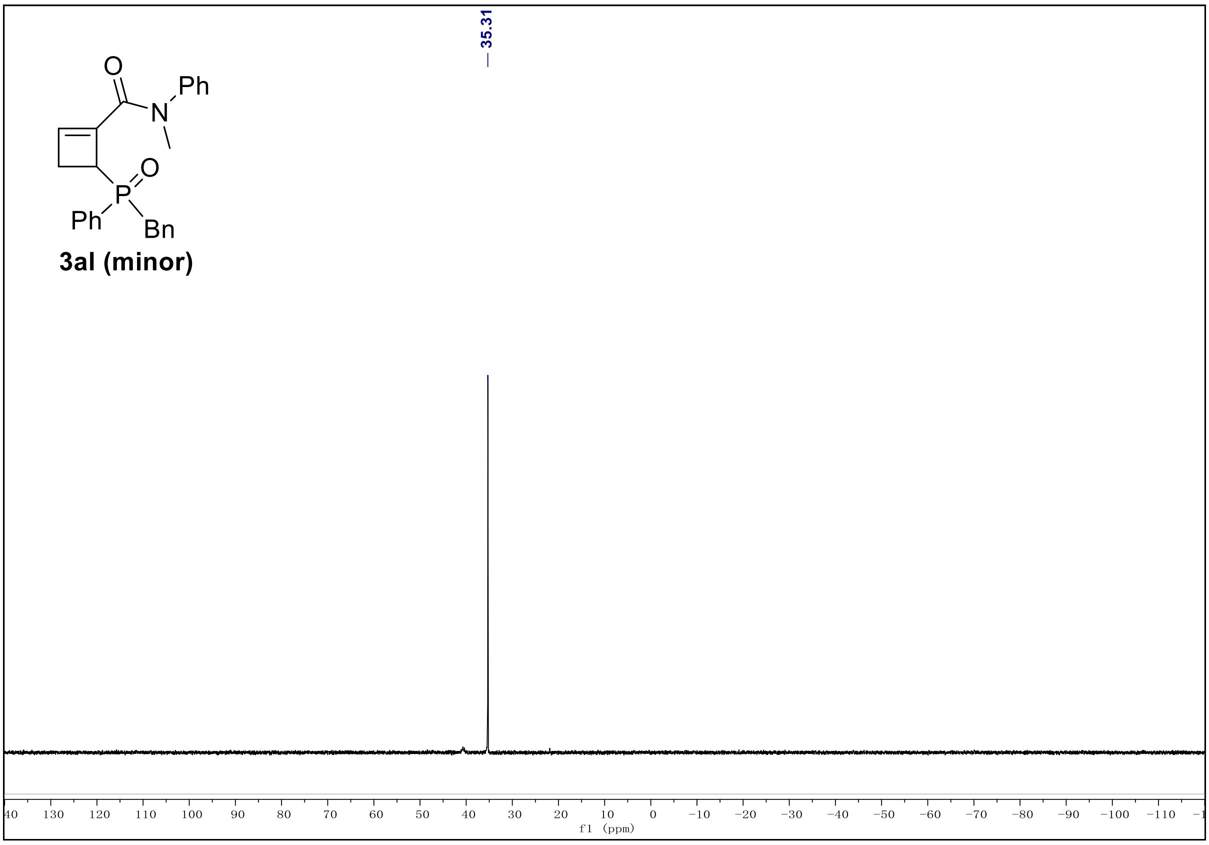

35.31
O
Ph
N
P
O
Ph
Bn
3al (minor)
f1 (ppm)

^{1}H NMR (400 MHz, $CDCl_3$), ^{13}C NMR (100 MHz, $CDCl_3$) and ^{31}P NMR (162 MHz, $CDCl_3$) spectrum of 3ta.

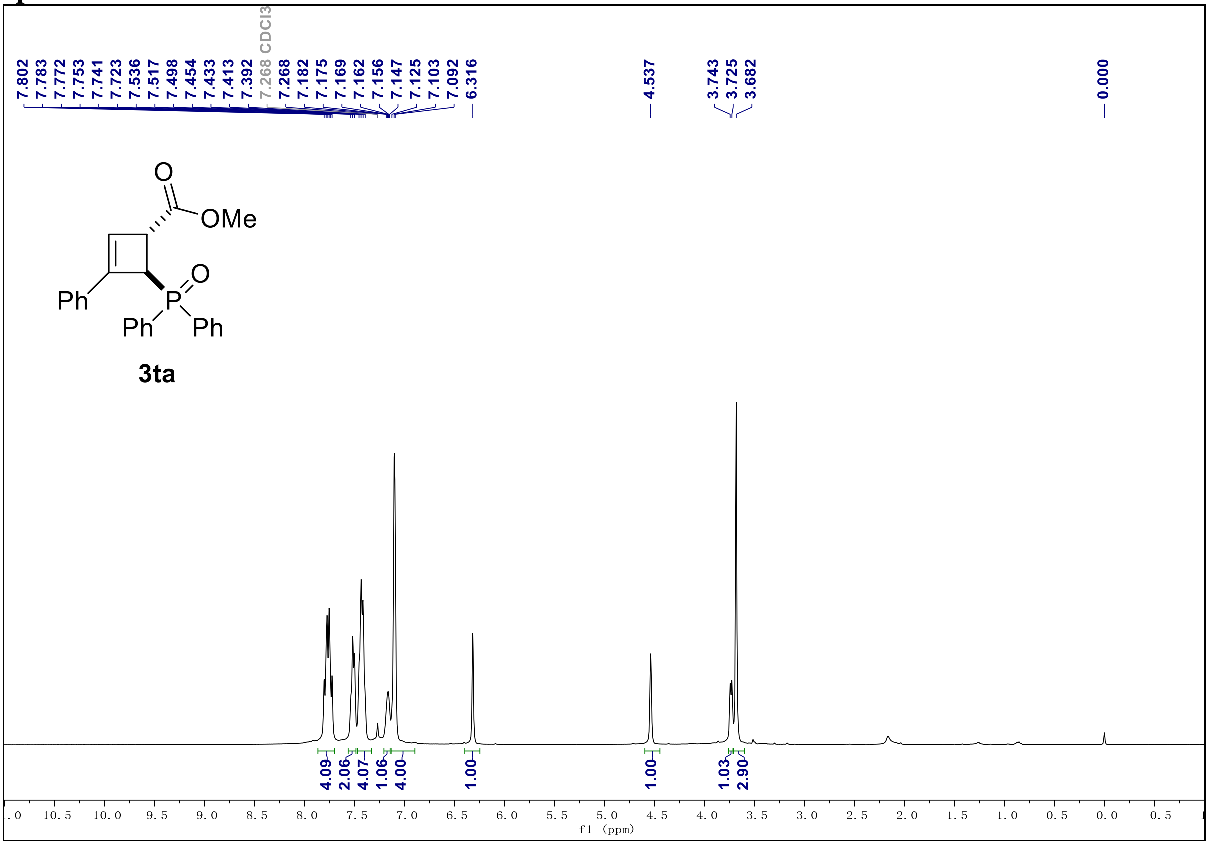

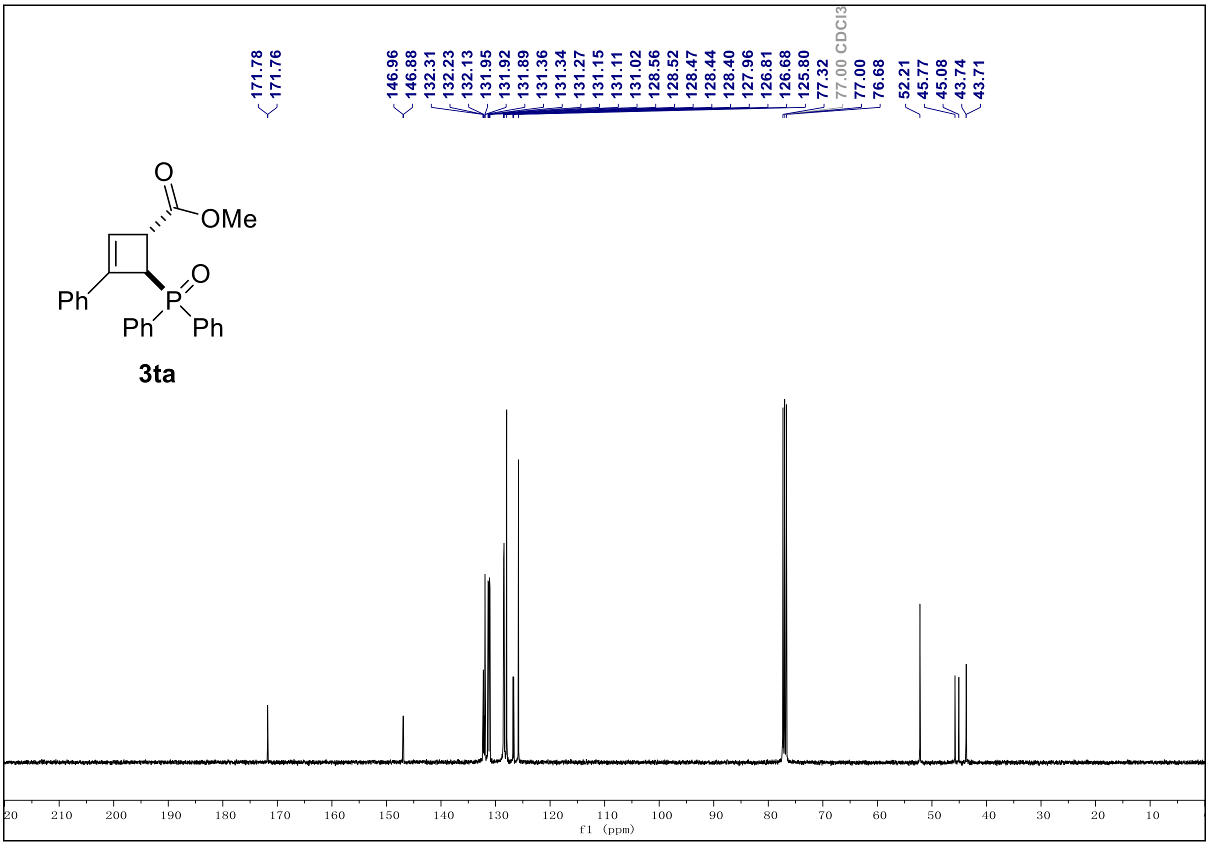

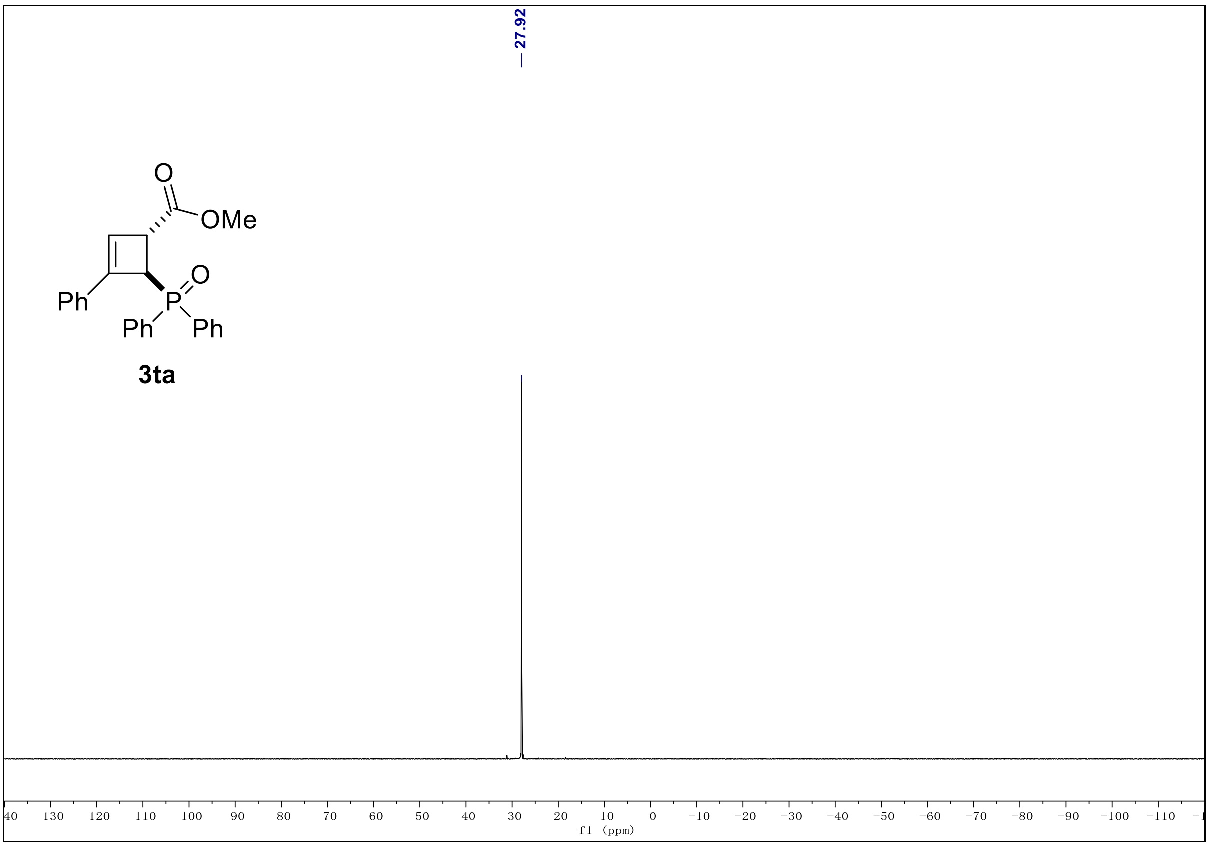

27.92
OMe
O
O
P
Ph
Ph
Ph
3ta
f1 (ppm)

^{1}H NMR (400 MHz, CDCl$_3$) and ^{13}C NMR (100 MHz, CDCl$_3$) spectrum of 4a.

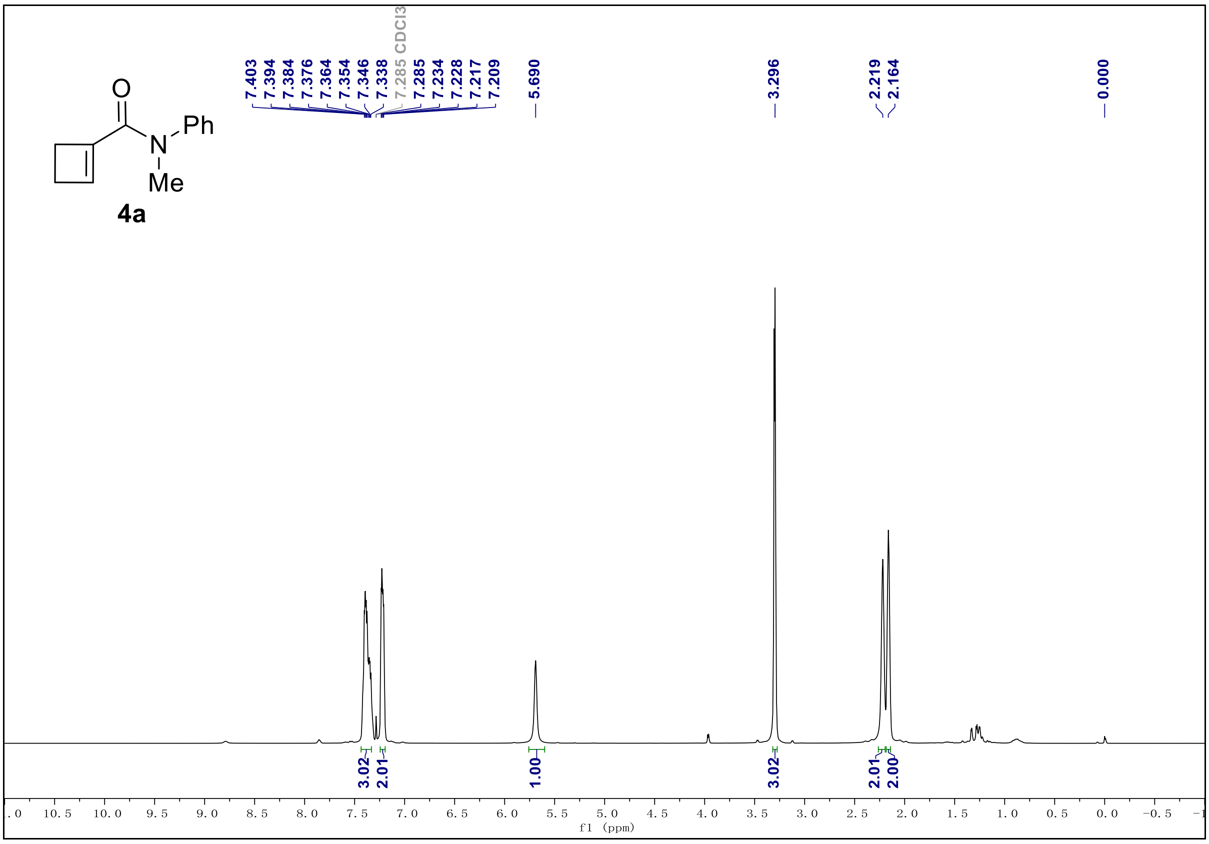

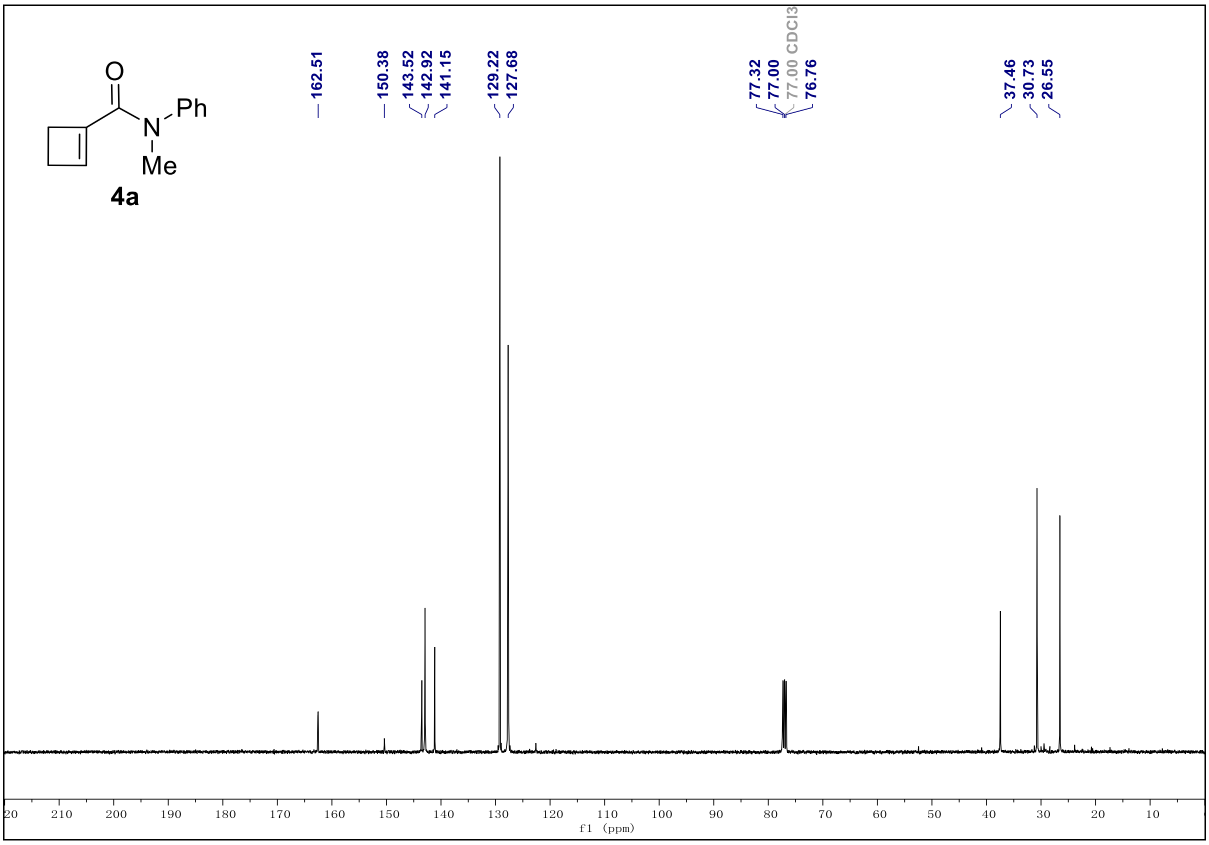

^{1}H NMR (400 MHz, CDCl3), ^{13}C NMR (100 MHz, CDCl3) and ^{31}P NMR (162 MHz, CDCl3) spectrum of 7.

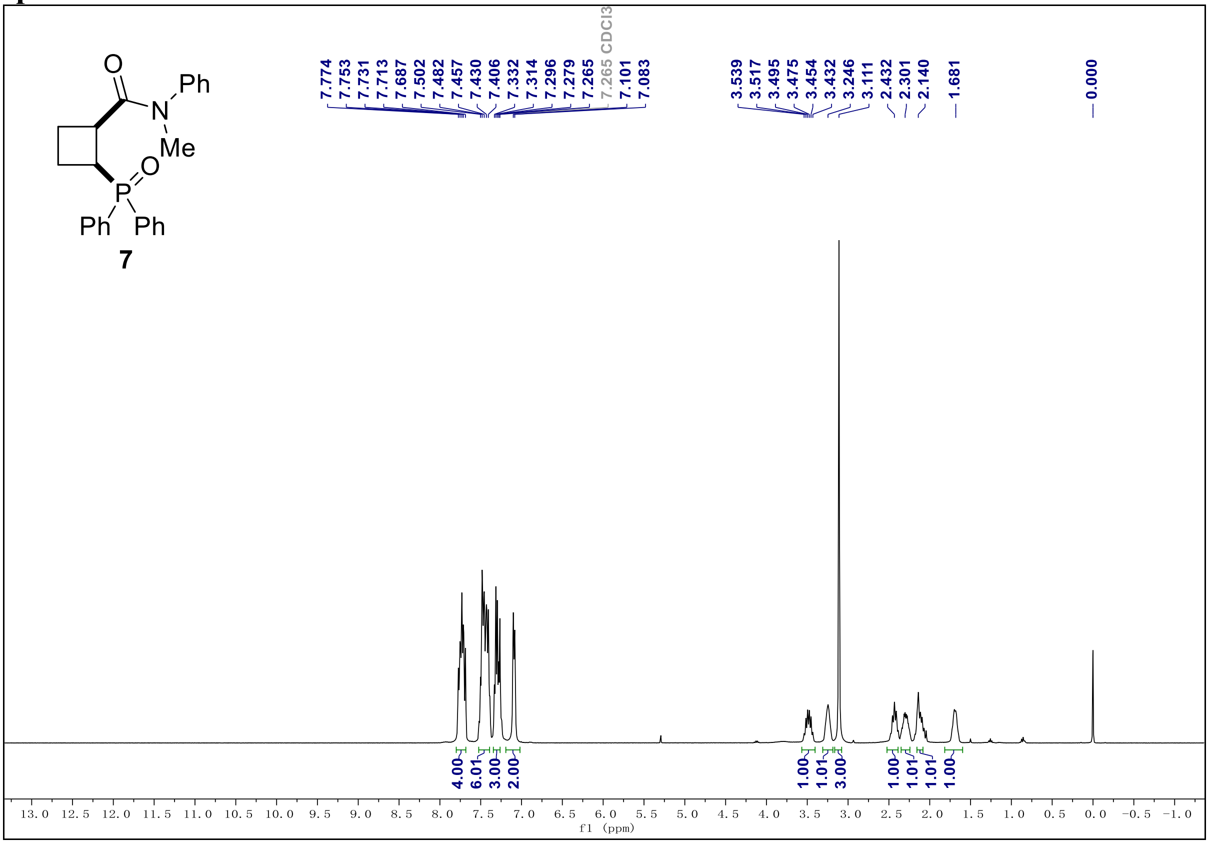

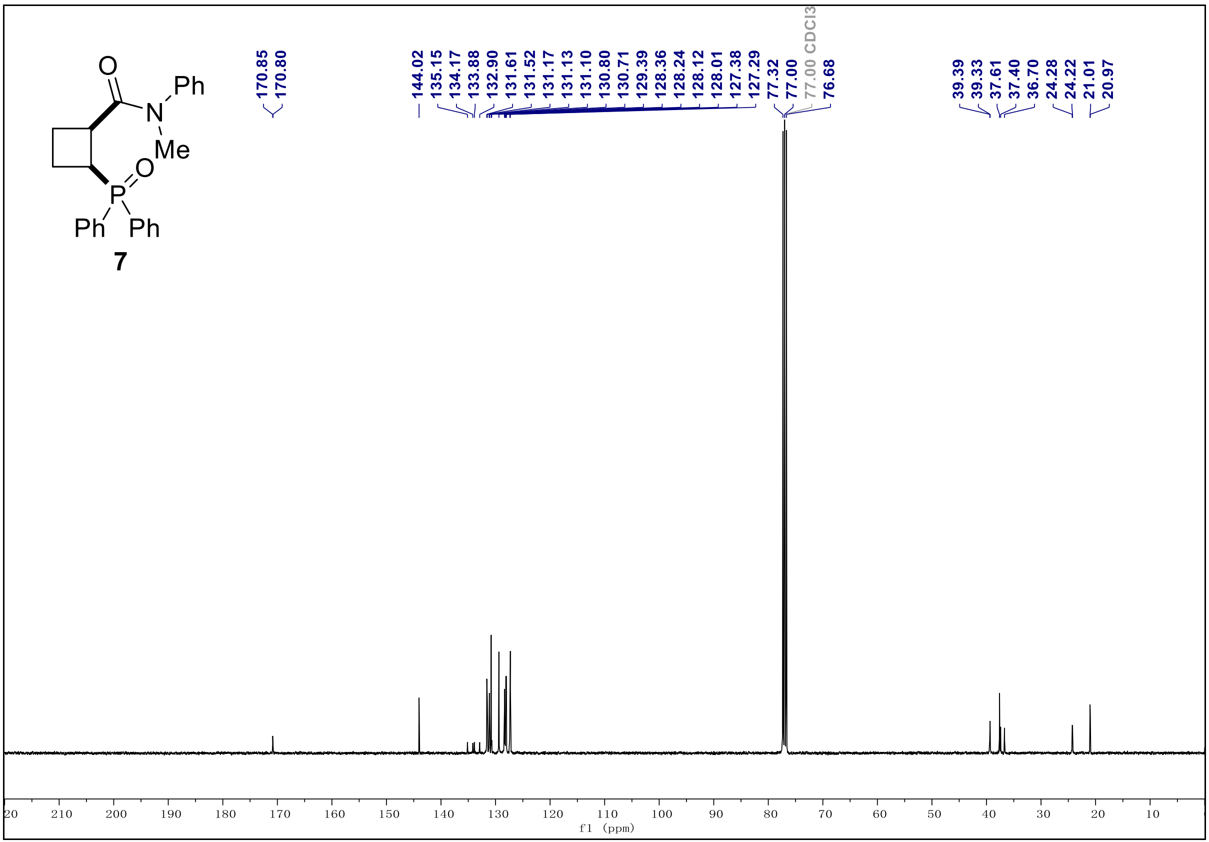

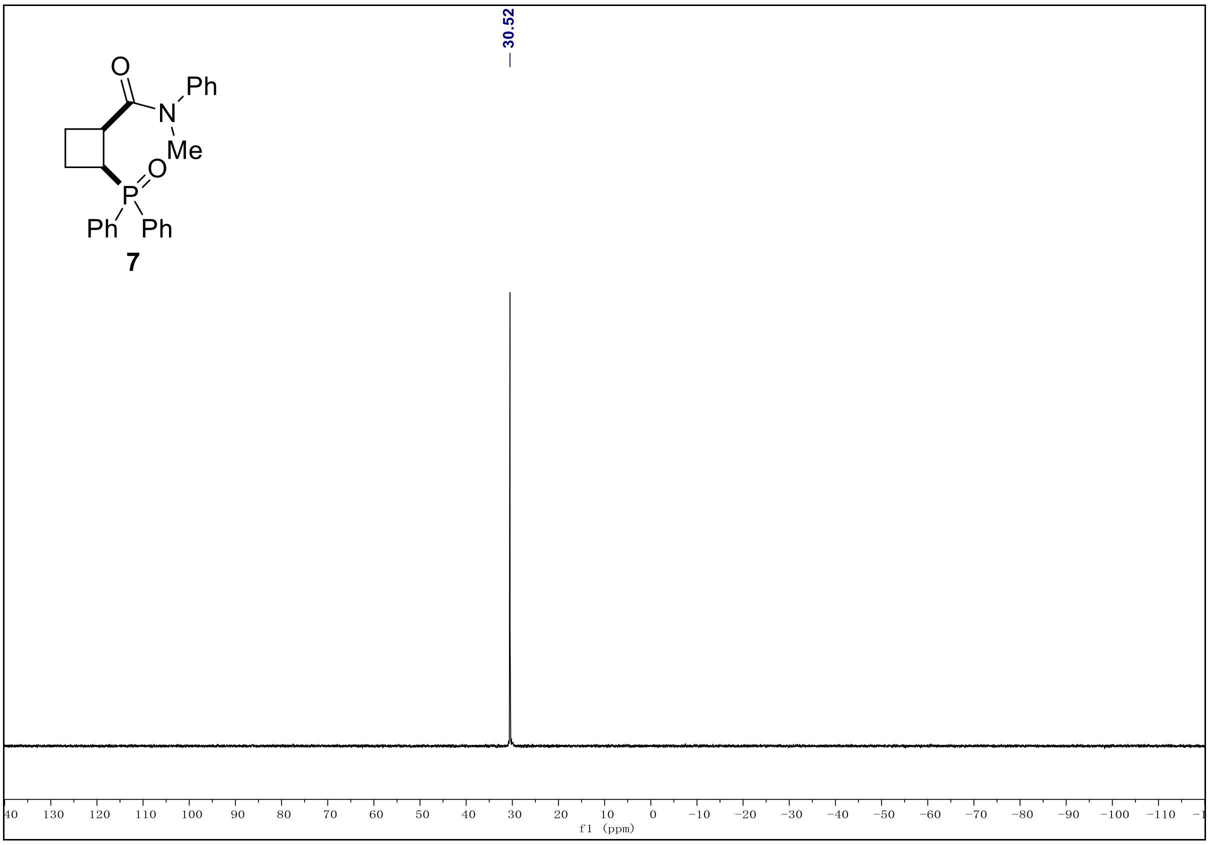

NOESY spectrum of 7

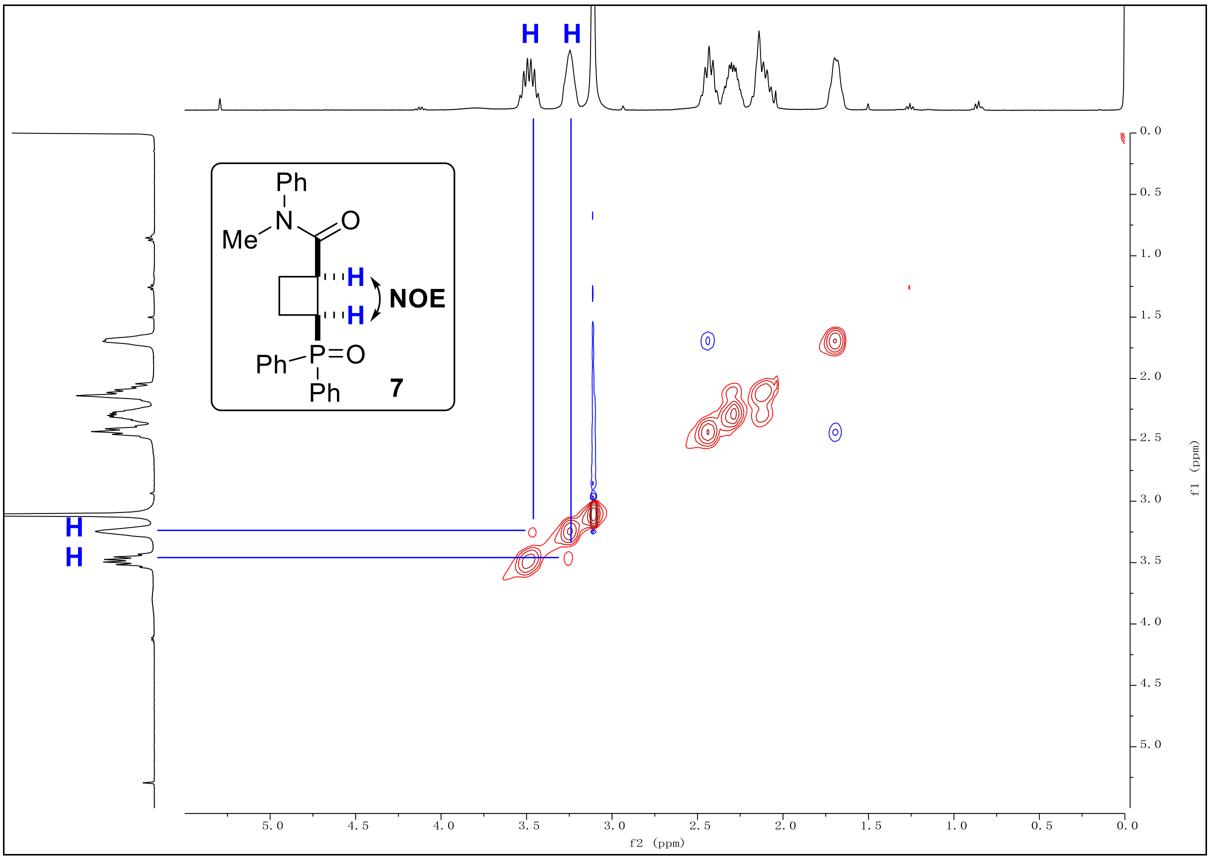

^{1}H NMR (400 MHz, CDCl$_3$), ^{13}C NMR (100 MHz, CDCl$_3$) and ^{31}P NMR (162 MHz, CDCl$_3$) spectrum of 8.

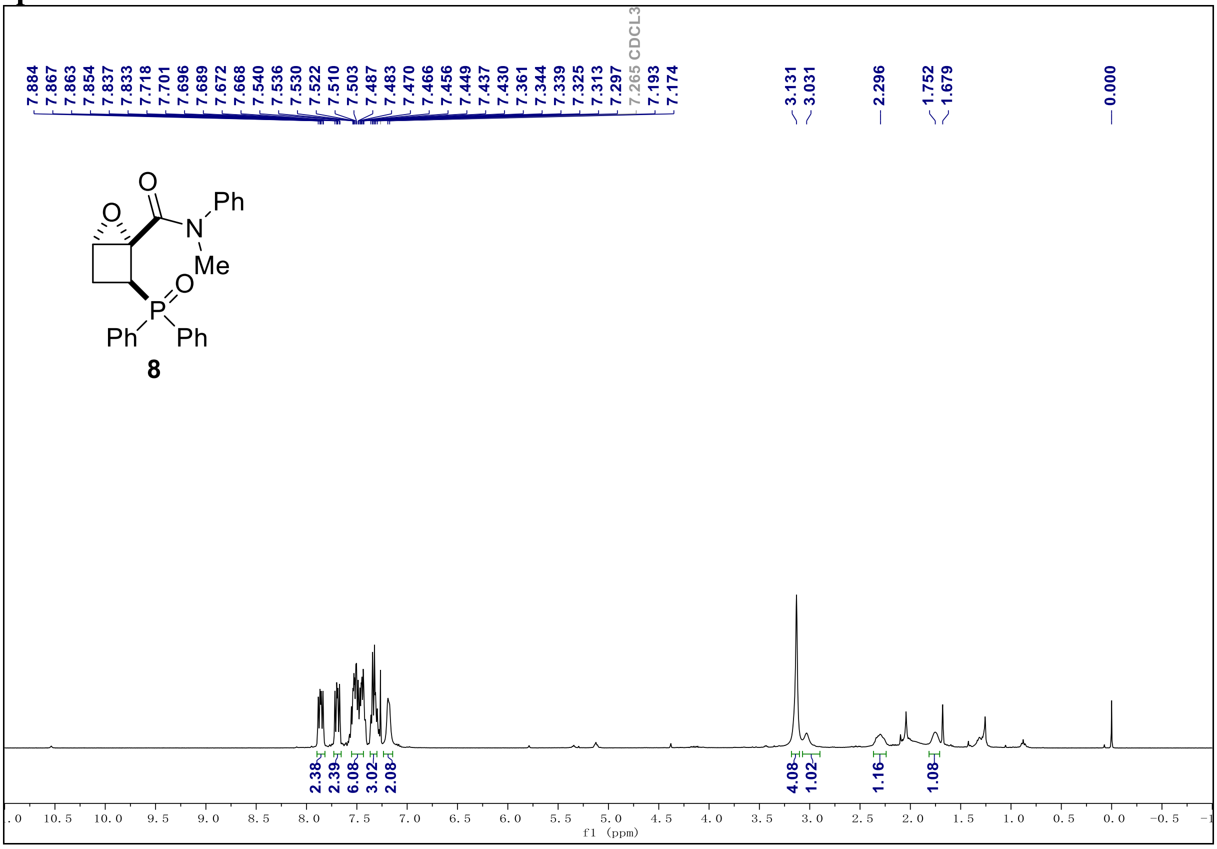

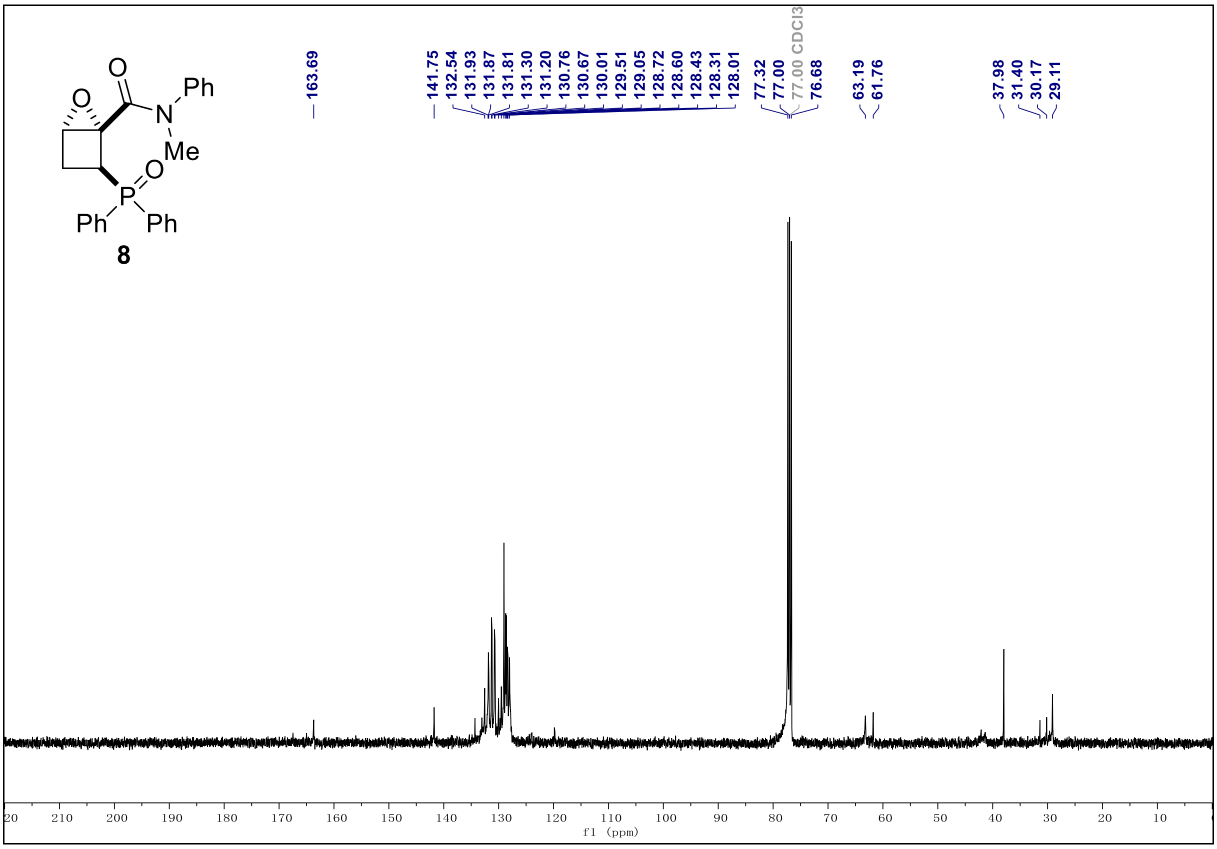

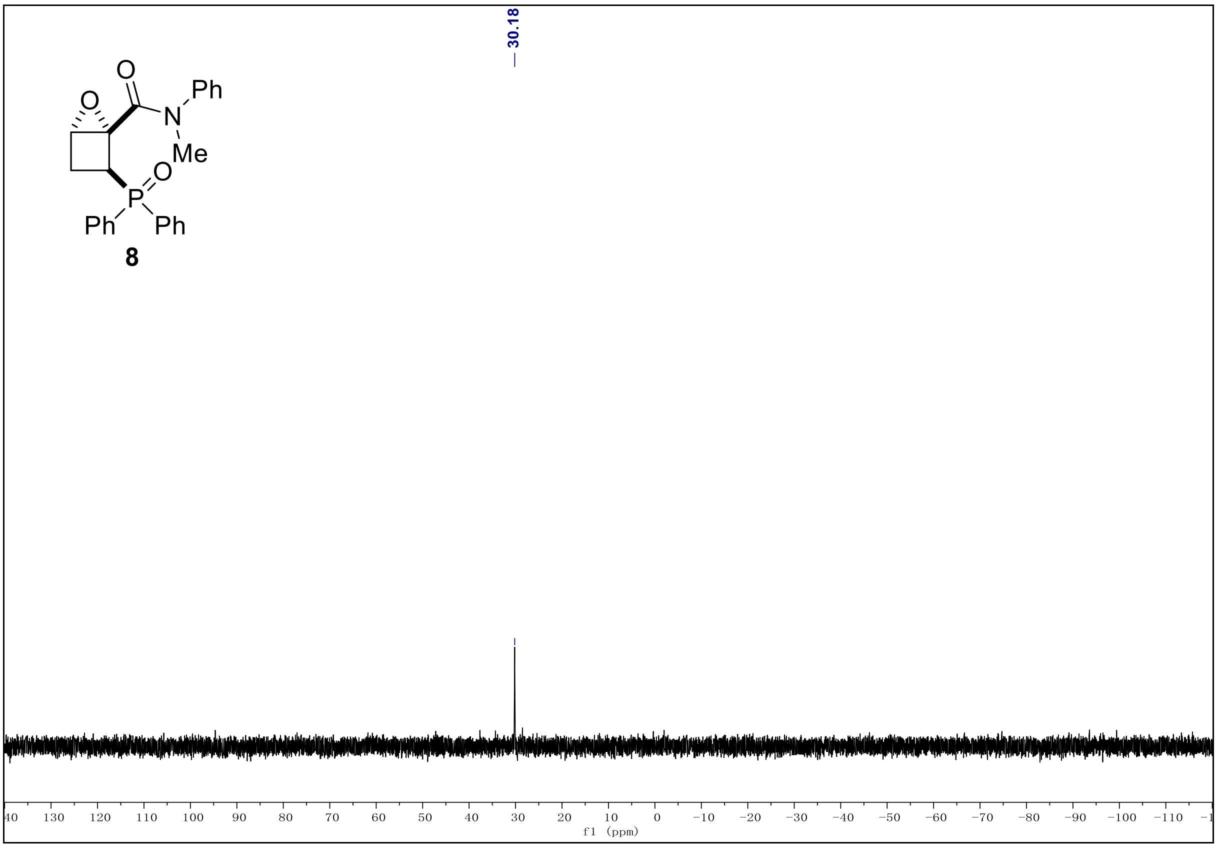
30.18
O
N
Ph
Me
P
Ph
Ph
8
40
130
120
110
100
90
80
70
60
50
40
30
20
10
0
−10
−20
−30
−40
−50
−60
−70
−80
−90
−100
−110
f1 (ppm)

^{1}H NMR (400 MHz, CDCl3), ^{13}C NMR (100 MHz, CDCl3) and ^{31}P NMR (162 MHz, CDCl3) spectrum of 9.

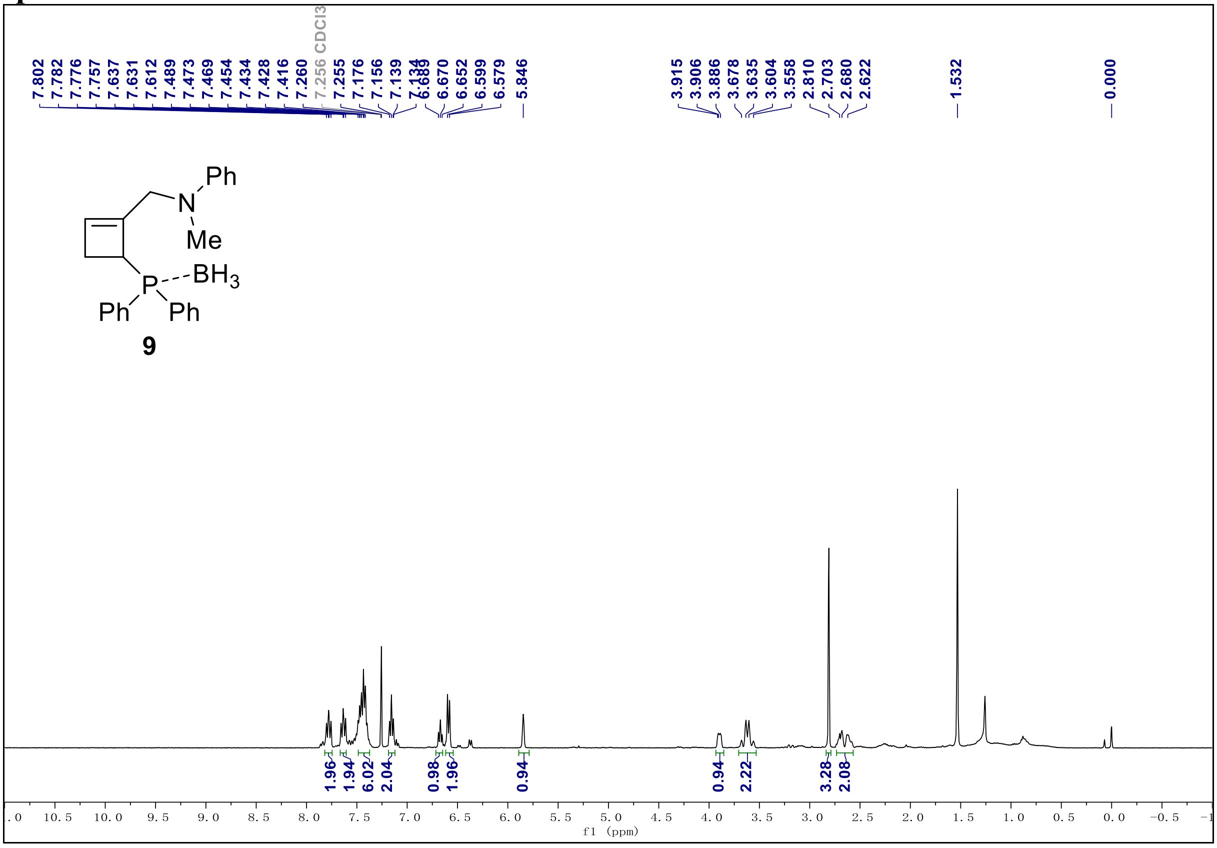

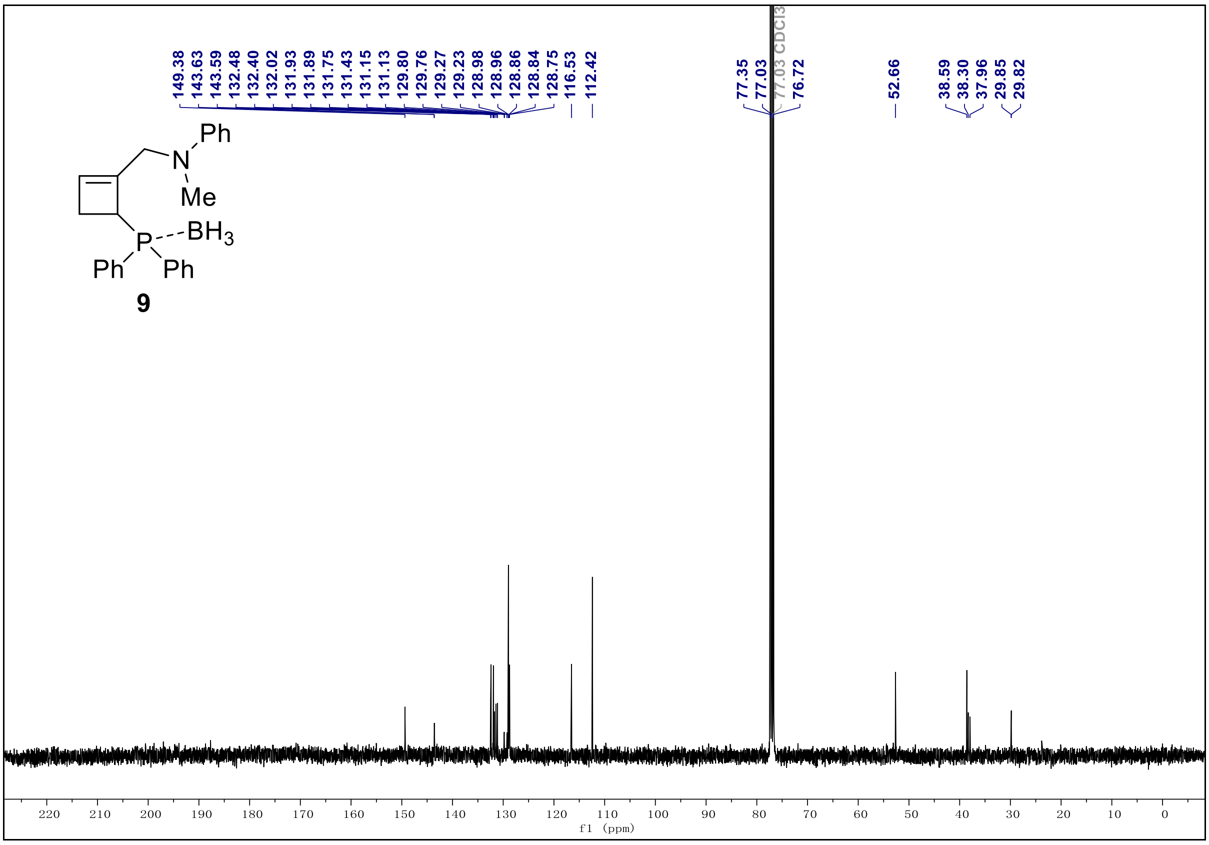

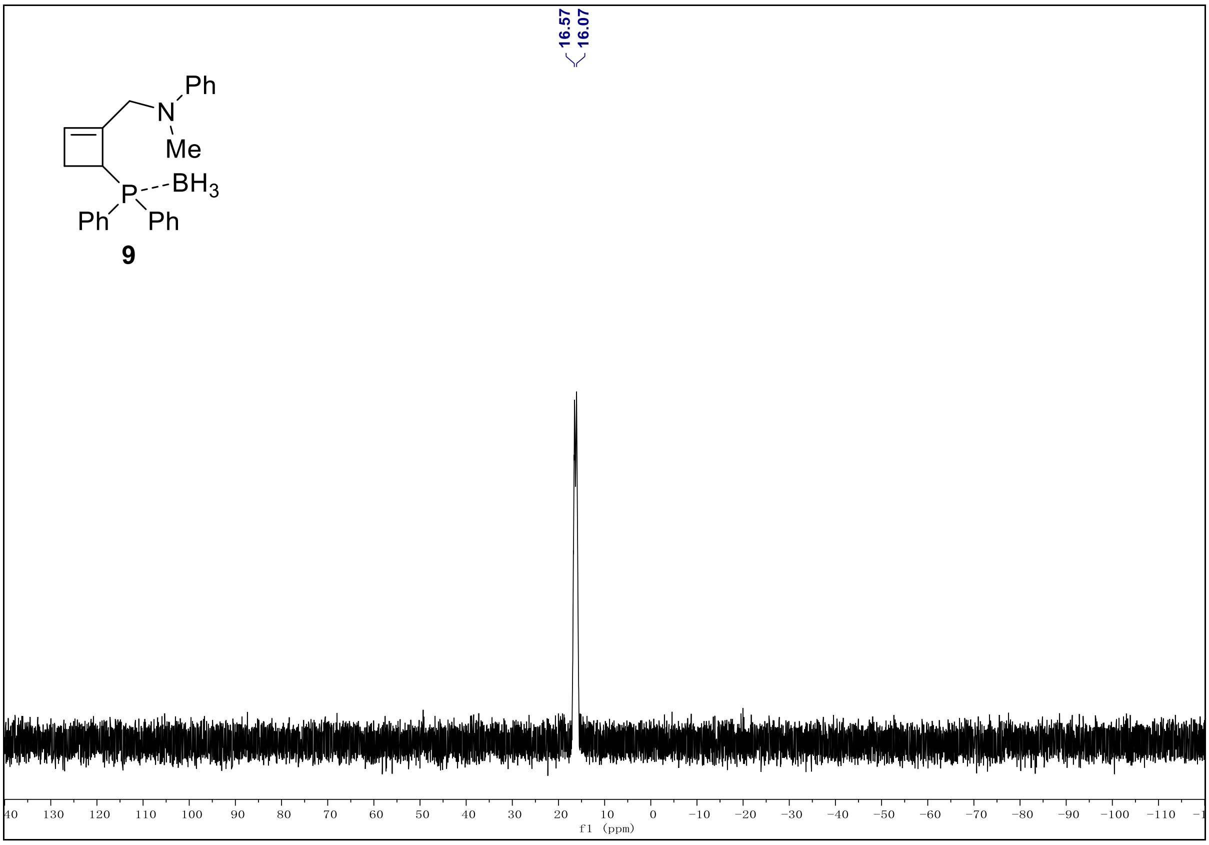
16.57
16.07
Ph
N
Me
BH3
P
Ph
Ph
9
f1 (ppm)

^{1}H NMR (400 MHz, CDCl$_3$), ^{13}C NMR (100 MHz, CDCl$_3$) and ^{31}P NMR (162 MHz, CDCl$_3$) spectrum of 10.

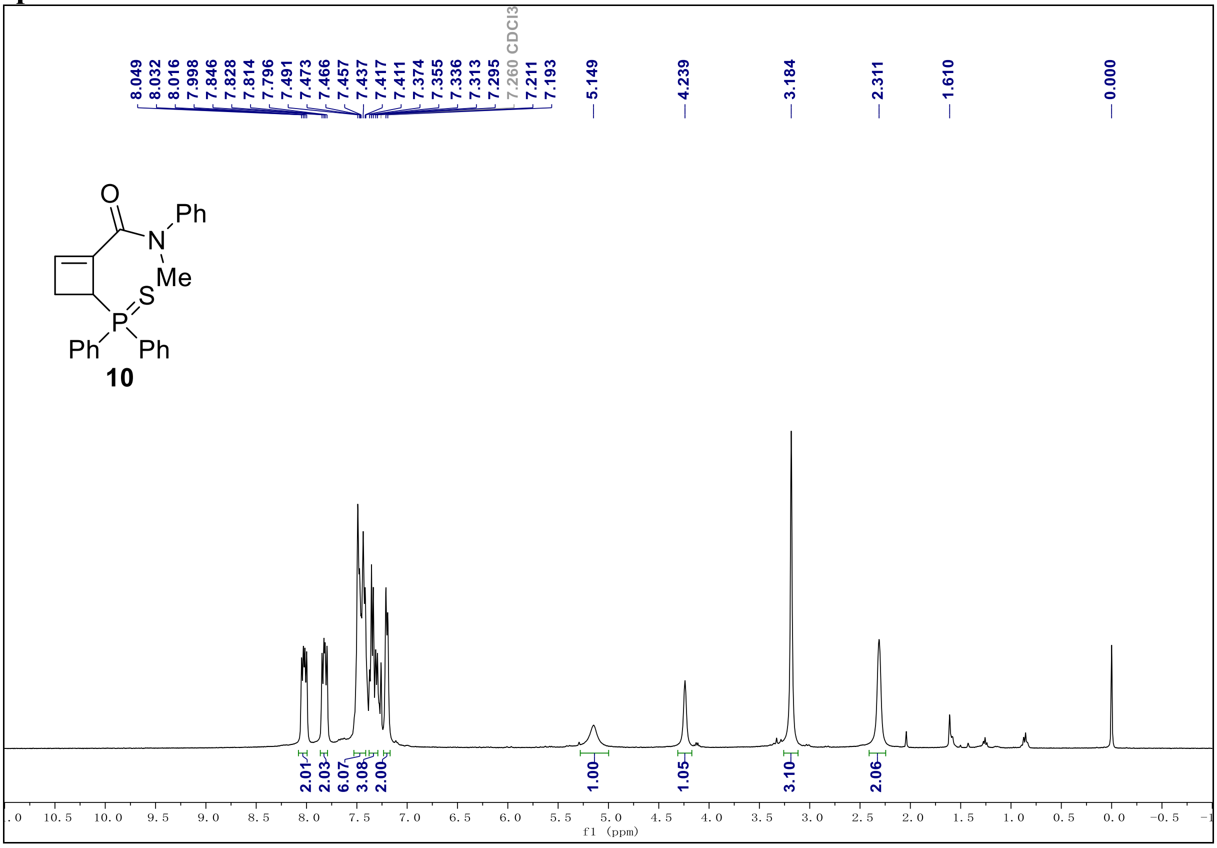

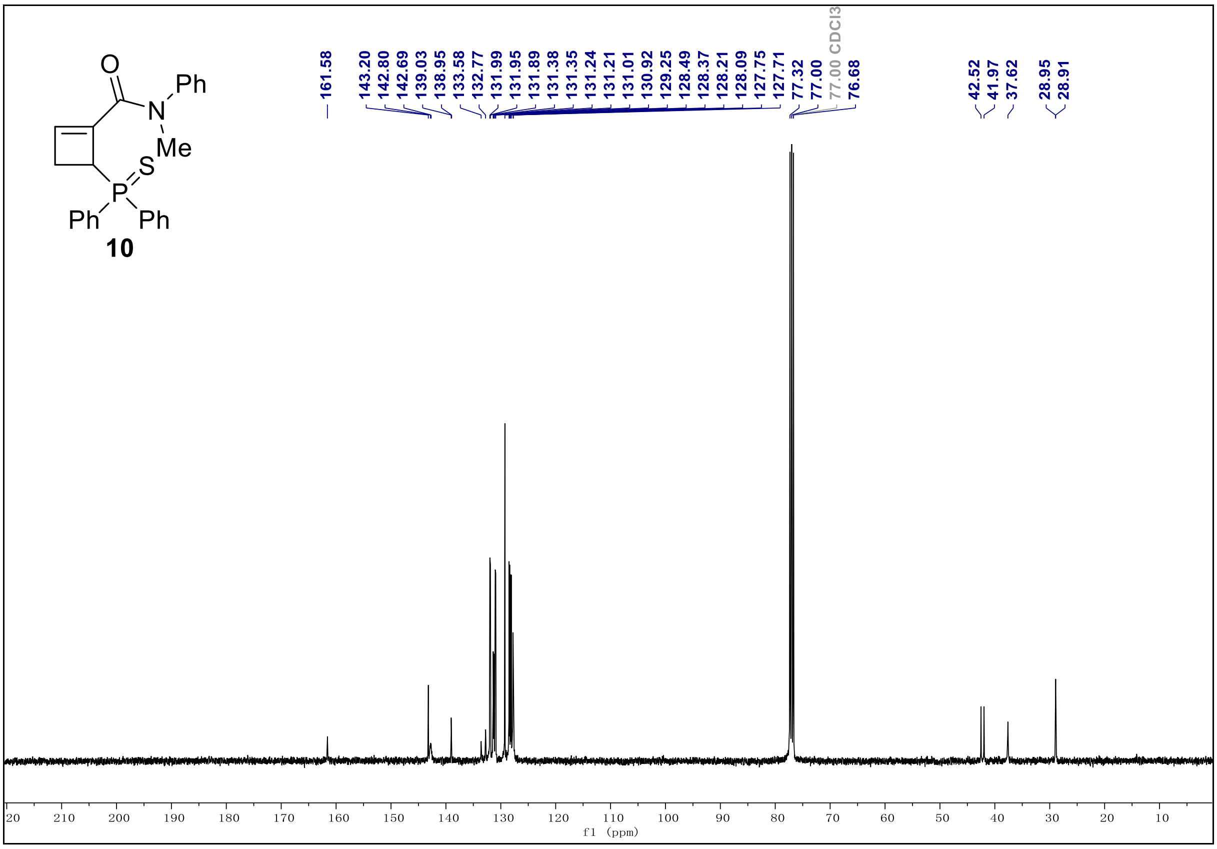

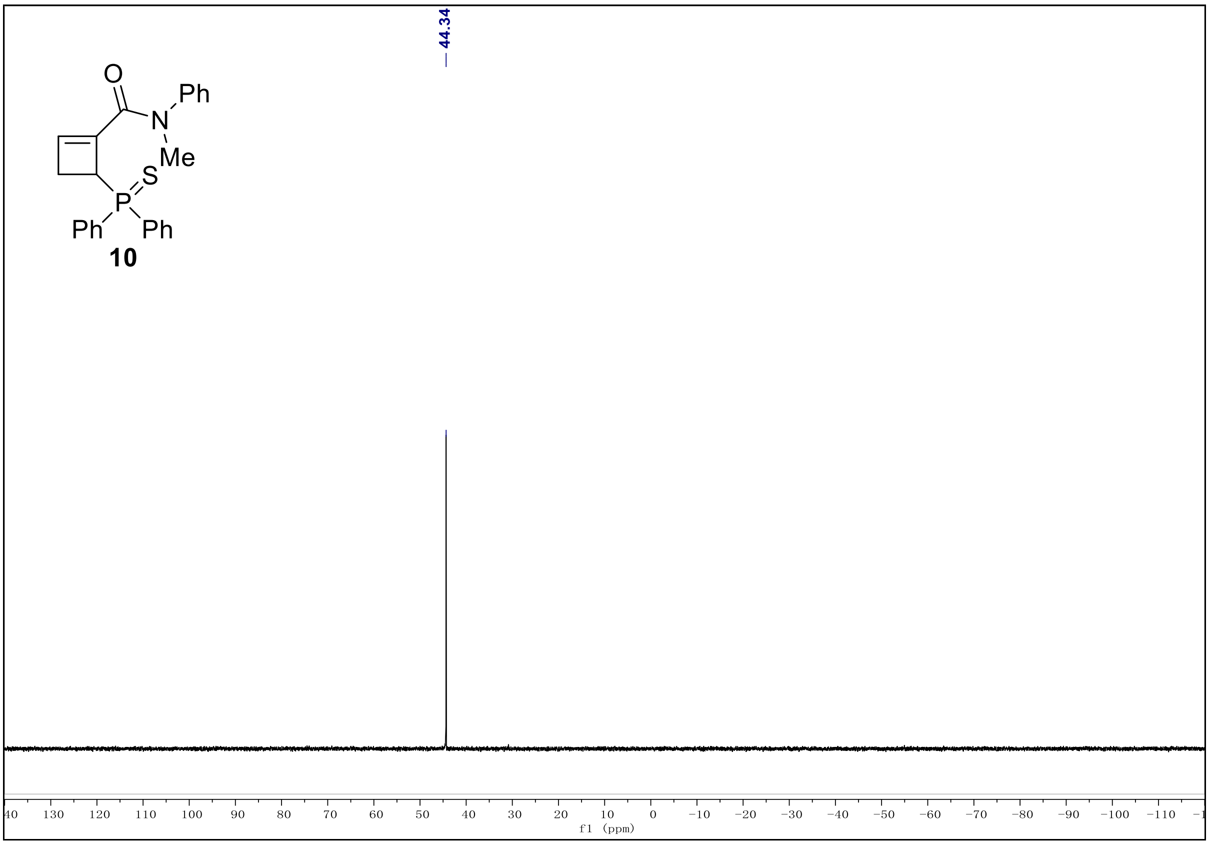

44.34
O
N
Ph
Me
S
P
Ph
Ph
10
40 130 120 110 100 90 80 70 60 50 40 30 20 10 0 −10 −20 −30 −40 −50 −60 −70 −80 −90 −100 −110
f1 (ppm)

^{1}H NMR (400 MHz, $CDCl_3$), ^{13}C NMR (100 MHz, $CDCl_3$) and ^{31}P NMR (162 MHz, $CDCl_3$) spectrum of 11.

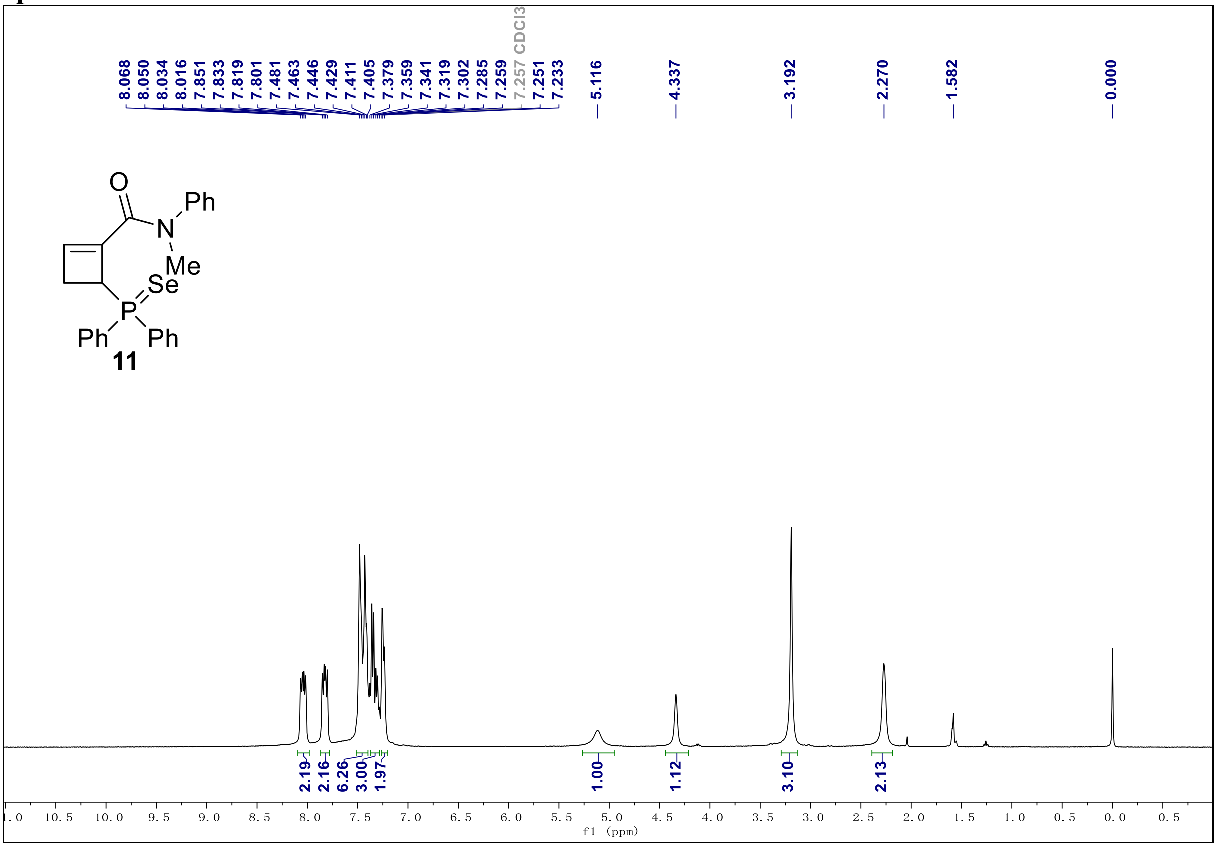

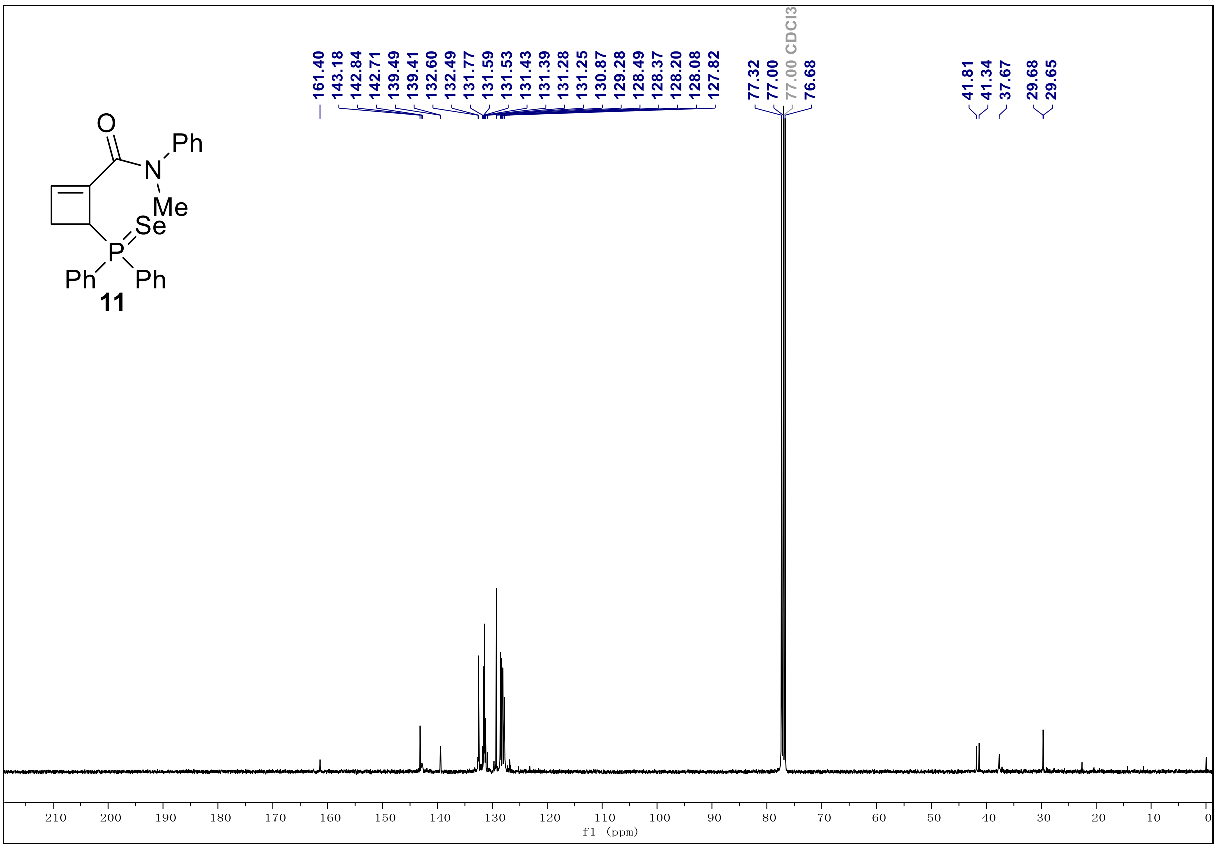

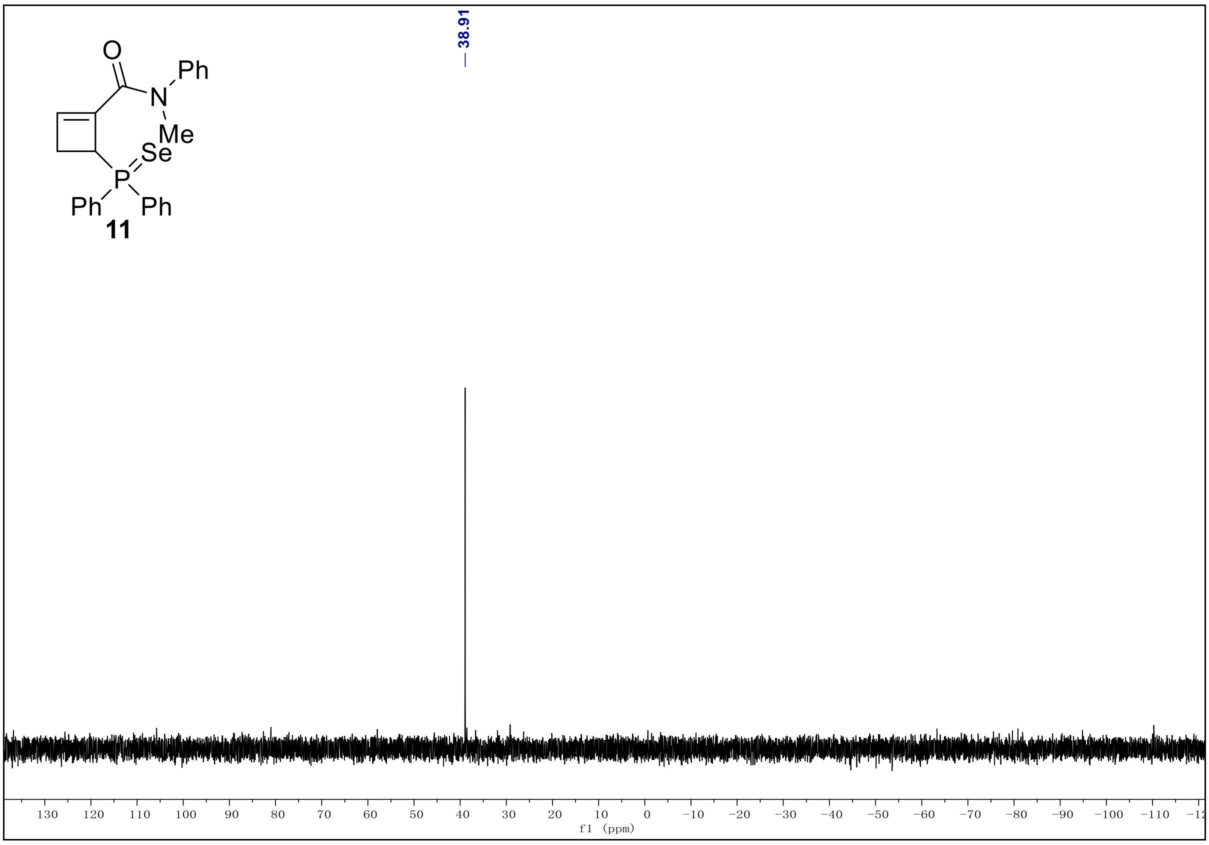

O
Ph
N
Me
Se
P
Ph
Ph
11
38.91
130 120 110 100 90 80 70 60 50 40 30 20 10 0 −10 −20 −30 −40 −50 −60 −70 −80 −90 −100 −110
f1 (ppm)
